# Supplementary material for: Rational design of N-heterocyclic compound classes via regenerative cyclization of diamines
Source: Nat Commun. 2023 Feb 3;14:595. doi: 10.1038/s41467-023-36220-w (PMC9898245; doi:10.1038/s41467-023-36220-w)
Supplement: Supplementary file 1 — Supplementary Information [file 41467_2023_36220_MOESM1_ESM.pdf]

Supplementary information

Rational design of N-heterocyclic compound classes via regenerative cyclization of diamines

Robin Fertig, Felix Leowsky-Künstler, Torsten Irrgang & Rhett Kempe*

Lehrstuhl Anorganische Chemie II – Katalysatordesign, Sustainable Chemistry Centre, Universität Bayreuth, 95440 Bayreuth, Germany

*Address correspondence to kempe@uni-bayreuth.de

Content

Supplementary Methods......3

1. **Materials and Methods**......3
2. **Screenings for the synthesis of 2-aminophenyl-2,3-dihydro-perimidines (A)** 4
3. **Screenings for the synthesis of 7,7a,8,9-tetrahydro-10*H*-imidazo[1,5-*a*]-perimidin-10-ones (C)**......8
4. **Scale up experiments**......10
5. **Synthesis of ligands and complexes**......11
6. **Synthesis of 2-aminobenzyl alcohol derivatives**......11
7. **Synthesis of 1,8-diaminonaphthalene derivatives**......13
8. **Synthesis of 2-aminophenyl-2,3-dihydro-perimidines (A)**......15
9. **Synthesis of fertigines (B)**......16
10. **Synthesis of 7,7a,8,9-tetrahydro-10*H*-imidazo[1,5-*a*]-perimidin-10-ones (C)**......18
11. **Characterization of fertigines (B)**......19
12. **Mechanistic investigations**......22
13. **Isolation and characterization of products**......31
14. **NMR spectra of isolated products**......108
15. **LC-HRMS spectra**......201
16. **Crystallographic data**......213

Supplementary References......219

Supplementary Methods

1. Materials and Methods

All reactions and manipulations with air sensitive compounds being present were performed under dry argon (Ar 5.0) or nitrogen (N_2 5.0), using Schlenk and glove box techniques. Nonhalogenated solvents were dried over sodium benzophenone, 2-methyltetrahydrofuran (2-MeTHF) was dried over calcium hydride, and halogenated solvents were dried over P_2O_5. Deuterated solvents were bought from Cambridge Isotope Laboratories, distilled accordingly, and stored over molecular sieves (3 Å). 1,8-Diaminonaphthalene was sublimated before use. Other chemicals were purchased from commercial vendors and used without further purification. NMR spectra were collected on a Varian INOVA 400 MHz spectrometer or on a Bruker Avance III HD 500 MHz. Chemical shifts (δ) are reported in ppm relative to residual solvent signal ($CDCl_3$: 7.26 ppm (^1H), 77.16 ppm (^{13}C), DMSO-d_6: 2.50 ppm (^1H), 39.51 ppm (^{13}C), C_6D_6: 7.16 ppm (^1H), 128.39 ppm (^{13}C), thf-d_8: 1.72 ppm, 3.58 ppm (^1H), 67.21 ppm, 25.31 ppm (^{13}C), CD_3CN: 1.94 ppm (^1H), 1.32 ppm, 118.26 ppm (^{13}C)). Coupling constants (J) are given in Hz (coupling patterns: s: singlet, d: doublet, t: triplet, q: quartet, m: multiplet). GC analyses were carried out using an Agilent Technologies 6890N system equipped with a Macherey-Nagel (MN) Optima 5 HT column (30 m, 320 µm, 0.25 µm) or an Agilent Technologies 6850 system equipped with a MN Optima 17 column (30 m, 320 µm, 0.25 µm). GC/MS analyses were carried out on an Agilent 7890A/MSD 5975C system equipped with a HP-5MS column (30 m, 320 µm, 0.25 µm). For column chromatography, Alox N (90 Å pore withdraw, 50 – 200 µm particle size) from Macherey-Nagel was used. All organic compounds were characterized by ^1H and ^{13}C NMR analysis. Unknown compounds or compounds with incomplete spectroscopic literature data were further analysed via elemental analysis (Elementar Unicube or LC-HRMS). Hydrogenations were conducted in PARR Instrument stainless steel autoclaves N-MT5 300 mL equipped with heating mantles and temperature controllers. Liquid chromatography-high resolution mass spectra (LC-HRMS) were obtained from a Thermo Fisher scientific Q-Exactive instrument with a hybrid quadrupole orbitrap analyser in ESI+ mode. For liquid chromatography a Luna Omega PS C18 (100x2.1 mm, 1.6 µm) column was used with a solvent gradient from 30:70 MeCN/water to 90:10 MeCN/water. The samples were dissolved in ethanol or DMSO. The obtained single crystals were mounted on a cryoloop (MiTeGen) with a layer of fomblin YR-1800 (CAS: 69991-67-9). All measurements were performed on a STOE STADIVARI [λ(Mo Kα) = 0.71073 Å] equipped with a dectris (Pilatus 200 K – 20 Hz) detector and an Oxford Cryostream low temperature unit. Structure solution and

refinement was accomplished with OlexSys2[1], SHELXL-2014[2], and Mercury 2020.1[3]. Non-hydrogen atoms were anisotropically refined, hydrogen atoms were included in the refinement on calculated positions riding on their carrier atoms.

2. Screenings for the synthesis of 2-aminophenyl-2,3-dihydro-perimidines (A)

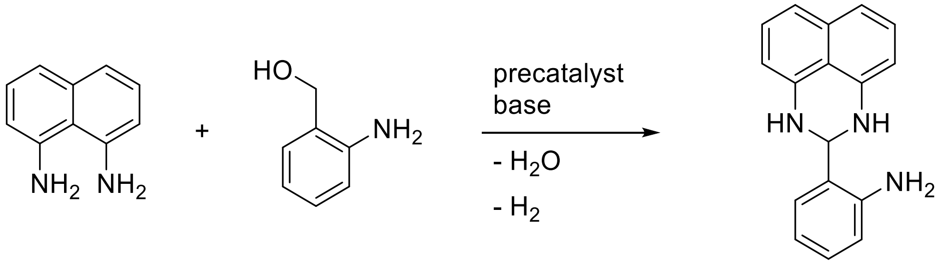

NDA 2-ABA A1

Supplementary Figure 1 Synthesis of **A1** starting from naphthalene-1,8-diamine (**NDA**) and 2-aminobenzyl alcohol (**2-ABA**).

Supplementary Table 1 Precatalyst screening.[a]

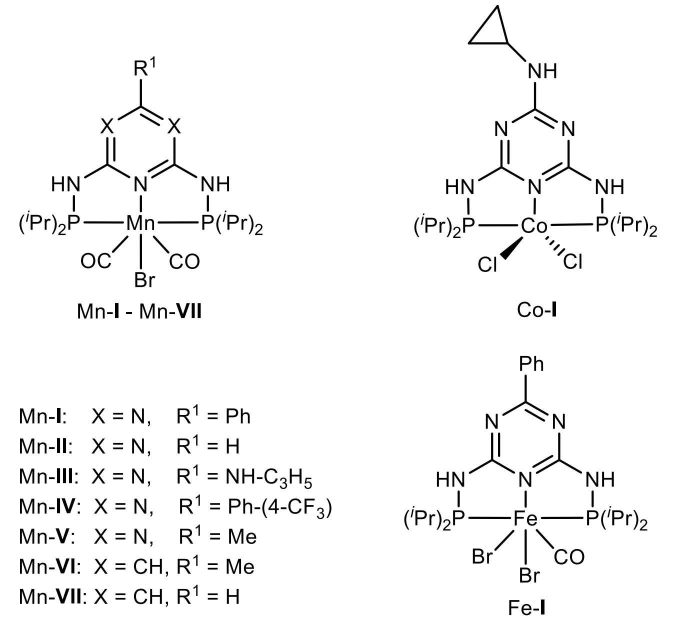

Entry	Precatalyst	**A1** [%][b]
1	Mn-**I**	75
2	Mn-**II**	41
3	Mn-**III**	68
4	Mn-**IV**	37
5	Mn-**V**	64
6	Mn-**VI**	5
7	Mn-**VII**	7
8	Co-**I**	0
9	Fe-**I**	11
10	[MnBr(CO)$_5$]	6
11	no catalyst	0

[a] Reaction conditions: 1 mmol NDA, 1 mmol 2-ABA, 1 mmol KO^{t}Bu, 1 mol% precatalyst, 4 mL 2-MeTHF, 100 °C (oil bath), 1.5 h. [b] Determined by GC with dodecane as an internal standard.

Supplementary Table 2 Base screening.[a]

Entry	Base	**A1** [%][b]
1	KO*t*Bu	78
2	NaO*t*Bu	40
3	KOH	82
4	NaOH	29
5	KH	49
6	NaHMDS	5
7	KHMDS	79
8	Cs_2CO_3	5
9	no base	0

[a] Reaction conditions: 1 mmol NDA, 1 mmol 2-ABA, 1 mmol base, 1 mol% precatalyst Mn-**I**, 4 mL 2-MeTHF, 100 °C (oil bath), 1.5 h. [b] Determined by GC with dodecane as an internal standard.

Supplementary Table 3 Solvent screening.[a]

Entry	Solvent	**A1** [%][b]
1	2-MeTHF	78
2	THF	65
3	Diglyme	0
4	Dioxan	49
5	Toluene	47
6	Pyridine	20
7	*tert*-Amylalcohol	21
8	DME	64

[a] Reaction conditions: 1 mmol NDA, 1 mmol 2-ABA, 1 mmol KO*t*Bu, 1 mol% precatalyst Mn-**I**, 4 mL solvent, 100 °C (oil bath), 1.5 h. [b] Determined by GC with dodecane as an internal standard.

Supplementary Table 4 Temperature screening.[a]

Entry	Temperature [°C]	**A1** [%][b]
1	50	0
2	60	7
3	80	43
4	100	79
5	120	88
6	140	85

[a] Reaction conditions: 1 mmol NDA, 1 mmol 2-ABA, 1 mmol KO^{t}Bu, 1 mol% precatalyst Mn-**I**, 4 mL 2-MeTHF, 1.5 h. [b] Determined by GC with dodecane as an internal standard.

Supplementary Table 5 Base loading screening.[a]

Entry	Amount of KO^{t}Bu [mmol]	**A1** [%][b]
1	0	0
2	0.1	51
3	0.3	80
4	0.5	88
5	0.7	90
6	1	89
7	1.5	100
8	2	100

[a] Reaction conditions: 1 mmol NDA, 1 mmol 2-ABA, KO^{t}Bu, 1 mol% precatalyst Mn-**I**, 4 mL 2-MeTHF, 100 °C (oil bath), 1.5 h. [b] Determined by GC with dodecane as an internal standard.

Supplementary Table 6 Precatalyst Mn-I loading.[a]

Entry	Amount of precatalyst Mn-**I** [mol%]	**A1** [%][b]
1	0	0
2	0.1	41
3	0.2	65
4	0.5	69
5	1	82
6	1.5	96
7	2	100

[a] Reaction conditions: 1 mmol NDA, 1 mmol 2-ABA, 0.3 mmol KO^{t}Bu, precatalyst Mn-**I**, 4 mL 2-MeTHF, 100 °C (oil bath), 1.5 h. [b] Determined by GC with dodecane as an internal standard.

Supplementary Table 7 2-MeTHF amount screening.[a]

Entry	2-MeTHF [mL]	**A1** [%][b]
1	2	88
2	3	96
3	4	79
4	5	73

[a] Reaction conditions: 1 mmol NDA, 1 mmol 2-ABA, 0.3 mmol KO^{t}Bu, 1 mol% precatalyst Mn-**I**, 2-MeTHF, 100 °C (oil bath), 1.5 h. [b] Determined by GC with dodecane as an internal standard.

3. Screenings for the synthesis of 7,7a,8,9-tetrahydro-10*H*-imidazo[1,5-*a*]-perimidin-10-ones (C)

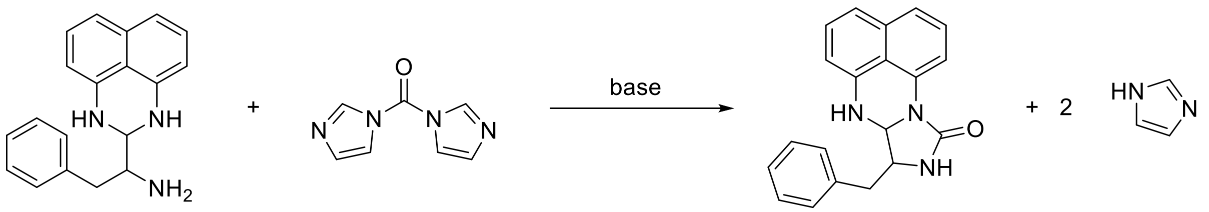

A26 **CDI** **C2**

Supplementary Figure 2 Synthesis of **C2** starting from aliphatic aminoperimidin **A26** and carbonyldiimidazol (**CDI**).

Supplementary Table 8 Base screening.[a]

Entry	Base	**C2** [%][b]
1	KO*t*Bu	91
2	NaO*t*Bu	86
3	KOH	89
4	NaOH	74
5	DBU	64
6	NaHMDS	83
7	K_2CO_3	59
8	no base	0

[a] Reaction conditions: 0.5 mmol **A26**, 0.6 mmol CDI, 0.15 mmol base, 10 mL 1,4-dioxane, 130 °C (oil bath), 16 h, pressure tube, nitrogen atmosphere. [b] Determined by NMR.

Supplementary Table 9 Solvent screening.[a]

Entry	Solvent	**C2** [%][b]
1	1,4-Dioxane	92
2	THF	82
3	2-MeTHF	84
4	Toluol	86
5	*tert*-Amyl alcohol	72
6	Cyclopentyl methyl ether	89

[a] Reaction conditions: 0.5 mmol **A26**, 0.6 mmol CDI, 0.15 mmol KO*t*Bu, 10 mL solvent, 130 °C (oil bath), 16 h, pressure tube, nitrogen atmosphere. [b] Determined by NMR.

Supplementary Table 10 Temperature screening.[a]

Entry	Temperature [°C]	**C2** [%][b]
1	80	81
2	90	81
3	100	85
4	110	87
5	120	93
6	130	95
7	140	94

[a] Reaction conditions: 0.5 mmol **A26**, 0.6 mmol CDI, 0.15 mmol KO*t*Bu, 10 mL 1,4-dioxane, 16 h, pressure tube, nitrogen atmosphere. [b] Determined by NMR.

Supplementary Table 11 Base loading screening.[a]

Entry	Amount of KO*t*Bu [mmol]	**C2** [%][b]
1	0	0
2	0.05	71
3	0.1	86
4	0.15	92
5	0.2	91
6	0.25	92

[a] Reaction conditions: 0.5 mmol **A26**, 0.6 mmol CDI, x mmol KO*t*Bu, 10 mL 1,4-dioxane, 130 °C (oil bath), 16 h, pressure tube, nitrogen atmosphere. [b] Determined by NMR.

Supplementary Table 12 Amount of CDI screening.[a]

Entry	Amount of CDI [mmol]	**C2** [%][b]
1	0.5	68
2	0.525	74
3	0.55	73
4	0.575	77
5	0.6	70
6	0.625	66
7	0.65	66

[a] Reaction conditions: 0.5 mmol **A26**, x mmol CDI, 0.15 mmol KO*t*Bu, 10 mL 1,4-dioxane, 130 °C (oil bath), 30 min, pressure tube, nitrogen atmosphere. [b] Determined by NMR.

Supplementary Table 13 Time screening.[a]

Entry	Time [h]	**C2** [%][b]
1	0.5	76
2	1	85
3	1.5	84
4	2	95
5	3	95
6	4	98
7	5	98
8	6	98

[a] Reaction conditions: 0.5 mmol **A26**, 0.575 mmol CDI, 0.15 mmol base, 10 mL 1,4-dioxane, 130 °C (oil bath), pressure tube, nitrogen atmosphere. [b] Determined by NMR.

4. Scale up experiments

Reaction conditions for upscaling the 2,3-dihydroaminoperimidine synthesis:

In a glovebox, 1,8-naphthalenediamin (15 mmol, 2373 mg) and 2-aminobenzyl alcohol (15 mmol, 1847 mg) are dissolved in 15 mL 2-MeTHF and added to a Schlenk tube. Mn-precatalyst Mn-**I** (0.15 mmol, 90 mg, 1 mol%), KO^{t}Bu (4.5 mmol, 504 mg, 30 mol%) and 30 mL 2-MeTHF are added to the Schlenk tube. The reaction mixture is heated at 100 °C using an open system consisting of a reflux condenser and a bubble counter. After 6 h reaction time, it is cooled down to room temperature, 15 mL H_2O is added, and the product is precipitated with pentane. The product **A1** is obtained in 96 % isolated yield (3.752 g) after filtration with pentane and subsequently drying in vacuo.

Reaction conditions for upscaling the fertigine synthesis:

In a glovebox, 1,8-naphthalenediamin (15 mmol, 2373 mg) and 2-aminobenzyl alcohol (15 mmol, 1847 mg) are dissolved in 15 mL 2-MeTHF and added to a Schlenk tube. Mn-precatalyst Mn-**I** (0.15 mmol, 90 mg, 1 mol%), KO^{t}Bu (4.5 mmol, 504 mg, 30 mol%) and 30 mL 2-MeTHF are added to the Schlenk tube. The reaction mixture is heated at 100 °C using an open system consisting of a reflux condenser and a bubble counter. After 6 h reaction time, benzaldehyde (15 mmol, 1516 µL) is added to the reaction using a syringe via a septum. The reaction is stirred overnight (15 h) at 100 °C, cooled down to room temperature and 10 mL H_2O is added. For

precipitation, pentane is added, the product is filtrated and washed with pentane, obtaining **B1a** in 93 % yield (4.868 g).

5. Synthesis of ligands and complexes

The ligands and precatalysts Mn-**I**/**II**/**III**/**IV**/**V**[4], Mn-**VI**/**VII**[5,6], Co-**I**[7,8] and Fe-**I**[9] were synthesized according to published procedures.

6. Synthesis of 2-aminobenzyl alcohol derivatives

15 mmol of anthranilic acid derivatives are dissolved in THF and cooled with an ice bath to 0 °C. 33 mmol $LiAlH_4$ is added in portions under rigorous stirring. After the addition, the reaction is led to warm up to room temperature and stirred overnight (15 h). The reaction is stopped following the Fieser workup: The reaction is cooled to 0 °C, diluted with diethyl ether and 1.25 mL water and 1.25 mL 15% aqueous NaOH solution are added slowly. Water (3.75 mL) is added, and the reaction is stirred for 15 min. Na_2SO_4 is added and the reaction is filtrated to remove the salts. The organic solvent is removed, and the crude product is purified by sublimation (60 – 100 °C). All 2-aminobenzyl alcohol derivatives were checked by ^1H NMR spectroscopy and GC/MS before use.

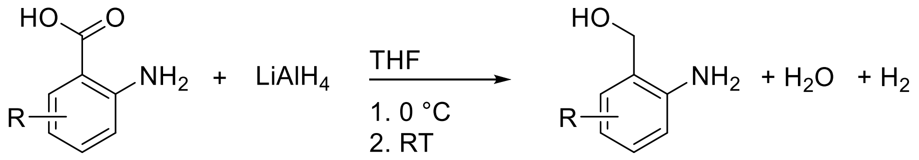

Supplementary Figure 3 General reaction conditions for the synthesis of 2-aminobenzyl alcohols.

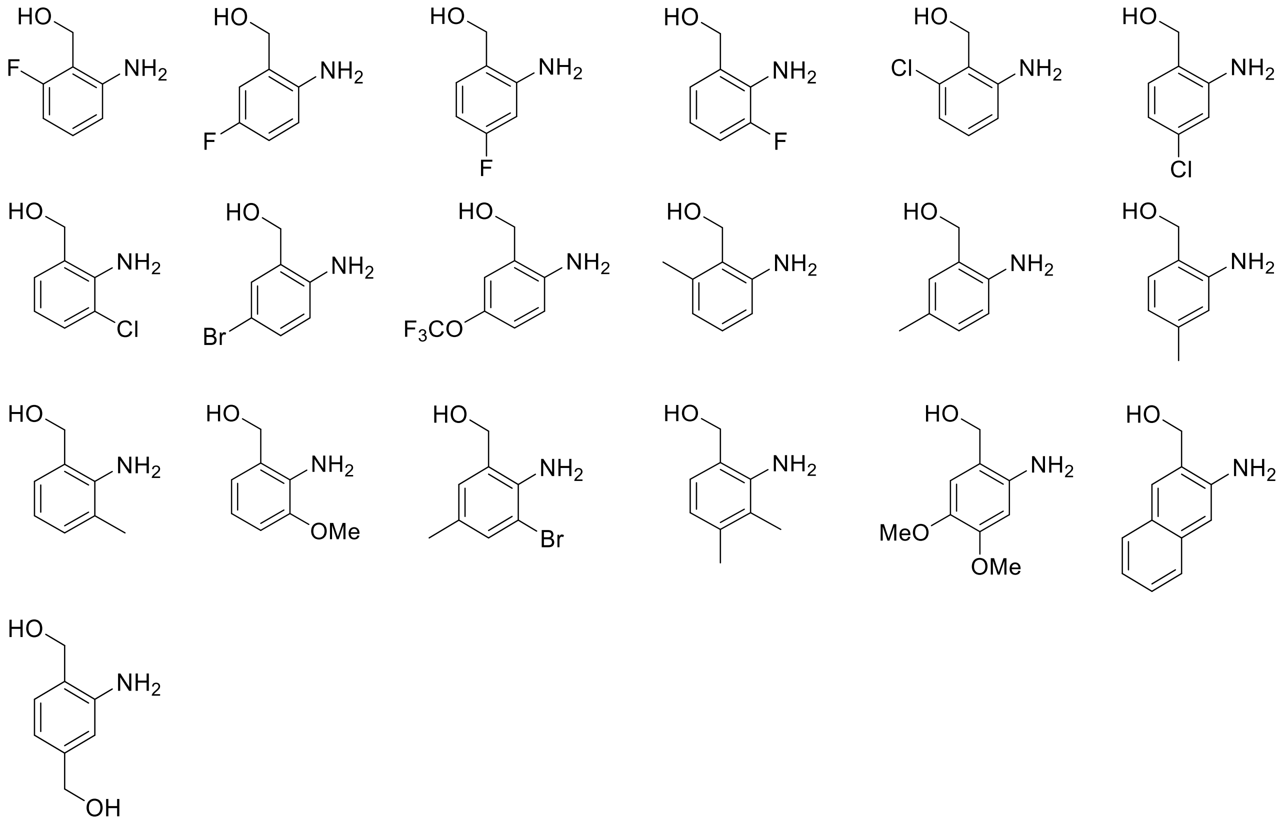

Supplementary Figure 4 Overview of the synthesized 2-aminobenzyl alcohols by reduction with $LiAlH_4$.

Synthesis of (6-amino-1,3-benzodioxol-5-yl)methanol

(6-Nitrobenzo[d][1,3]dioxol-5-yl)methanol (30 mmol) is dissolved in 30 mL methanol and a spade point of Pd@C is added. The hydrogen is stored in a rubber balloon, leading to a hydrogen atmosphere (ca. 1 atm) in the reaction flask. The reaction is stirred at room temperature for 24 h, Na_2SO_4 is added, and the reaction is filtrated. After removing the solvent, the crude product is purified by sublimation at 100 °C.

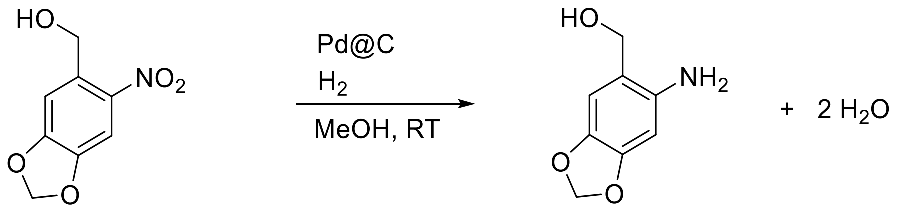

Supplementary Figure 5 Synthesis of (6-amino-1,3-benzodioxol-5-yl)methanol.

7. Synthesis of 1,8-diaminonaphthalene derivatives

Synthesis of 2-chloronaphthalene-1,8-diamine

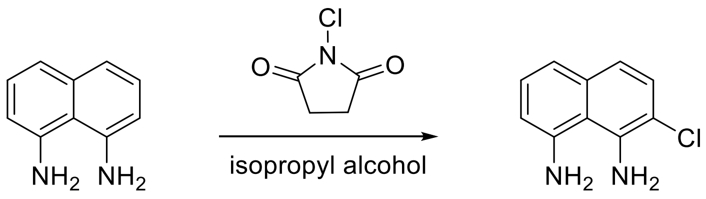

Supplementary Figure 6 Synthesis of 2-chloronaphthalene-1,8-diamine.

1,8-Diaminonaphthalene (9.97 g, 63 mmol) is dissolved in 100 mL isopropyl alcohol and *N*-chlorosuccinimide (8.41 g, 63 mmol) is added in small portions. The reaction is stirred at 80 °C with reflux for 2 h. After cooling down to room temperature, the solvent is removed and the reaction mixture is extracted with diethyl ether and water (3 x 30 mL). The organic layer is dried with Na_2SO_4 and the crude product is purified by column chromatography with Alox N (pentane/ethyl acetate: 4:2 → 2:3) obtaining 3.38 g of a white solid (17.5 mmol, 28 %). The purity is proofed via GC/MS and NMR analysis.

Synthesis of 5,6-diaminoacenaphthene

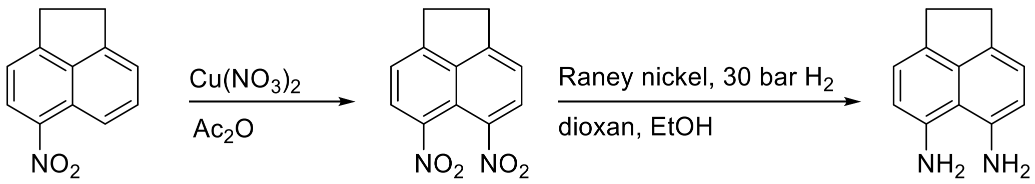

Supplementary Figure 7 Synthesis of 5,6-diaminoacenaphthene.

5-Nitroacenaphthene (6.98 g, 35 mmol) is dissolved in 150 mL Ac_2O and $Cu(NO_3)_2$ (6.56 g, 35 mmol) is added under rigorous stirring in small portions to the solution. The reaction is stirred at room temperature for 15 h, then the Ac_2O is removed in vacuo. 100 mL Water were added, and the mixture is stirred for ca. 30 minutes until the remaining Cu-salts are dissolved. 5,6-Dinitroacenaphthene precipitates, it is filtrated and dried. For further purification, 5,6-dinitroacenaphthene is recrystallized in a 2/1 mixture of dioxane and thf at 70 °C and obtained as white crystals in 38 % yield (3.28 g) after 3 days. The reduction is conducted by dissolving 1 g

of 5,6-dinitroacenaphthene in a 1/1 mixture of dioxan and EtOH and adding 1 mL of a Raney nickel suspension to it. The mixture is stirred at 50 °C and 30 bar H_2 for 15 h. After cooling down to room temperature, the pressure is released, and the mixture is filtrated. Precipitation with HCl in Et_2O, filtration of the HCl-salt and neutralisation with $NaHCO_3$ led to the product in 76 % yield (562 mg, 3.05 mmol). The purity is proofed by GC/MS and NMR analysis.

Synthesis of 3,6-di-*tert*-butylnaphthalene-1,8-diamine

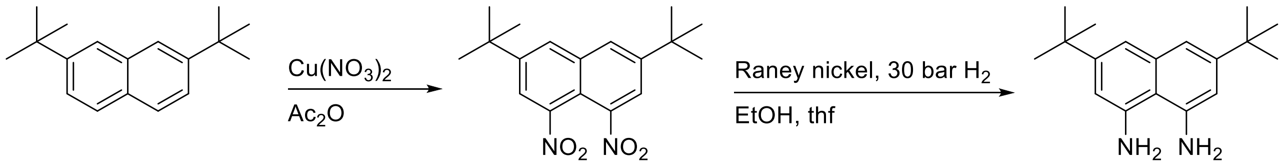

Supplementary Figure 8 Synthesis of 3,6-di-*tert*-butylnaphthalene-1,8-diamine.

2,7-Di-*tert*-butylnaphthalene (5 g, 20.7 mmol) is dissolved in 100 mL Ac_2O and copper(II) nitrate (7.88 g, 42 mmol) is added in small portions at 0 °C within 15 minutes. After stirring the mixture at room temperature for 2 hours, the reaction is stopped by pouring it in 500 mL ice water. The formed precipitate is filtrated, washed with water and dried in vacuo. The obtained yellow solid (5.62 g, 17 mmol) is used without further purification. 3,6-Di-*tert*-butyl-1,8-dinitronaphthalene is dissolved in a 1/1 mixture of EtOH/thf and 1 mL of a Raney nickel suspension is added. The mixture is stirred at 50 °C and 30 bar H_2 for 15 h. After cooling down to room temperature, the pressure is released, and the mixture is filtrated. The crude product is purified via column chromatography over Alox N (pentane/ethyl acetate 5:1 → 3:2) and obtained as a red solid (1244 mg, 4.61 mmol). The purity is proofed by GC/MS and NMR analysis.

8. Synthesis of 2-aminophenyl-2,3-dihydro-perimidines (A)

General reaction conditions for the synthesis 2-aminophenyl-2,3-dihydro-perimidines: In a glovebox, 2 mmol 1,8-naphthalenediamin and 2 mmol 2-aminobenzyl alcohol derivatives are dissolved in 1 mL 2-MeTHF and added to a Schlenk tube. 0.5 mL of a 0.04 mmol/mL stock solution of the Mn-precatalyst Mn-**I** and 0.5 mL of a 1.2 mmol/mL KO^{t}Bu stock solution is added to the Schlenk tube. 1 mL 2-MeTHF is added, and the reaction mixture is heated at 100 °C using an open system consisting of a reflux condenser and a bubble counter. After 2 h, the reaction is stopped by cooling down to room temperature and the addition of 2 mL H_2O. Depending on the product, we performed two different methods for purification: 1.) The mixture is extracted with dichloromethane (3 x 10 mL), the organic layers are dried with Na_2SO_4 and the solvent is removed in vacuo. The crude product is purified by column chromatography using Alox N as stationary phase. 2.) 5 mL H_2O is added, the product is precipitated with pentane, filtrated, and washed with pentane. Finally, it is dried in vacuo overnight.

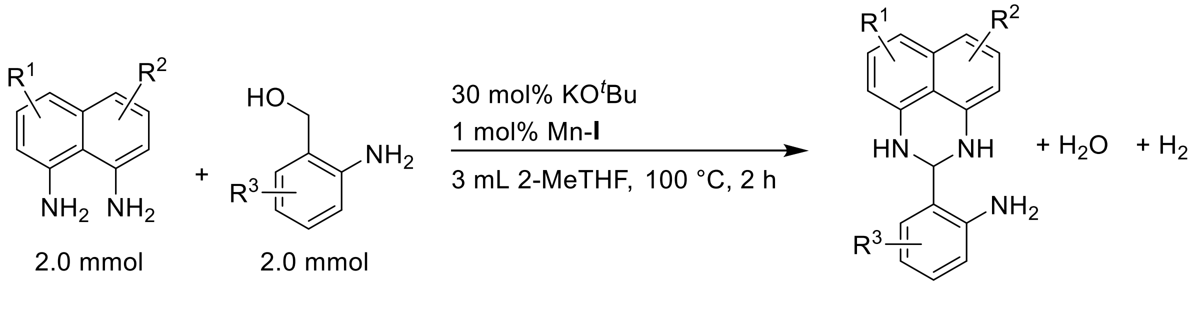

Supplementary Figure 9 Synthesis of 2,3-dihydroaminoperimidines **A1-A24**.

General reaction conditions for the synthesis of 1-(2,3-dihydro-1H-perimidin-2-yl)methanamines: In a glovebox, 1 mol% Mn-precatalyst Mn-**I** (0.02 mmol, dissolved in 1.5 mL 1,4-dioxane), 30 mol% KO^{t}Bu (0.6 mmol, dissolved in 1.5 mL 1,4-dioxane), 2 mmol 1,8-diaminonaphthalene and 2.2 mmol 2-aminopropan-1-ol derivatives are added to a Schlenk tube and dissolved in 9 mL 1,4-dioxane. The reaction mixture is heated at 100 °C using an open system consisting of a reflux condenser and a bubble counter. The mixture is stirred for 4 hours, cooled down to room temperature, the 1,4-dioxane is evaporated under vacuo and 6 mL water are added. The reaction mixture is extracted with ethyl acetate (3 x 50 mL), the organic layers are dried with Na_2SO_4 and the solvent is removed in vacuo. The crude product is purified via gradient column

chromatography using Alox N as stationary phase. To the product are 10 mL of an aqueous saturated solution of $NaHCO_3$ added, the product was extracted with ethyl acetate, dried with Na_2SO_4 and the solution was narrowed. At the end the product is purified via column chromatography over Silica C18 ec with ethyl acetate.

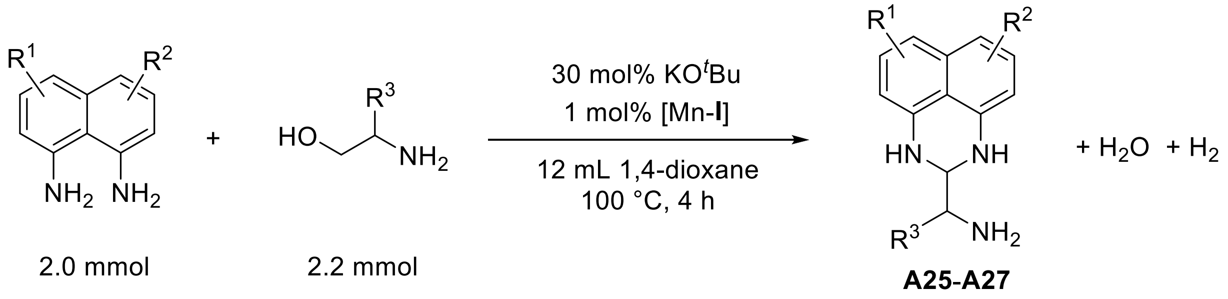

Supplementary Figure 10 Synthesis of 1-(2,3-dihydro-1*H*-perimidin-2-yl)methanamines **A25-A27**.

9. Synthesis of fertigines (B)

General reaction conditions for the synthesis of fertigines B1-B5: In a glovebox, 2 mmol 1,8-diaminonaphthalene and 2 mmol 2-aminobenzyl alcohol derivatives are dissolved in 1 mL 2-MeTHF and added to a Schlenk tube. 0.5 mL of a 0.04 mmol/mL stock solution of Mn-**I** and 0.5 mL of a 1.2 mmol/mL KO^{t}Bu stock solution is added to the Schlenk tube. 1 mL 2-MeTHF is added, and the reaction mixture is heated at 100 °C using an open system consisting of a reflux condenser and a bubble counter. After 2 hours reaction time, 2 mmol of various aldehydes are added to the reaction. For this, the aldehyde is dissolved in at least 0.5 mL 2-MeTHF and added to the reaction mixture through a septum with a syringe. After 15 h, the reaction is stopped by cooling down to room temperature. The work-up depends on the substrates used. Usually, 2 mL H_2O is added, and the reaction mixture is extracted with dichloromethane (3 x 10 mL). The organic layers were dried with Na_2SO_4 and the solvent is removed in vacuo. The crude product is purified by column chromatography using Alox N as stationary phase. Using some substrates, the product precipitates during reaction. If this is the case, 5 mL water is added, the reaction mixture is diluted with pentane and filtrated. After washing the residue with H_2O and cold pentane, it is dried in vacuo at 70 °C to obtain the product.

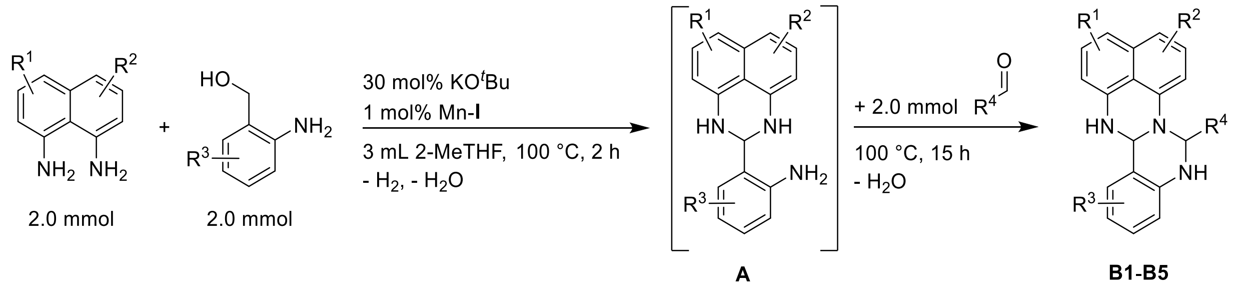

Supplementary Figure 11 Synthesis of fertigines **B1-B5**.

General reaction conditions for the synthesis of amino-fertigine derivatives B6a-B6c: In a glovebox, 1 mol% Mn-precatalyst Mn-**I** (0.02 mmol, dissolved in 0.5 mL 2-MeTHF), 30 mol% KO^{t}Bu (0.6 mmol, dissolved in 0.5 mL 2-MeTHF), 2 mmol 1,8-diaminonaphthalene and 2 mmol 2-aminobenzyl alcohol are added to a Schlenk tube and dissolved in 3 mL 2-MeTHF. The reaction mixture is heated at 100 °C using an open system consisting of a reflux condenser and a bubble counter. After 2 hours reaction time, 2.0 or 2.2 mmol 2-aminobenzyl alcohol derivatives are dissolved in 1.0 mL 2-MeTHF and added to the reaction mixture through a septum with a syringe. After 15 hours, the reaction is stopped by cooling down to room temperature and 4 mL water are added. The reaction mixture is diluted with pentane, the precipitate is filtrated and washed with water and pentane. The dried solid is slurred with ethanol and stirred for 10 min at 100 °C. After filtration, the product is dried in vacuo.

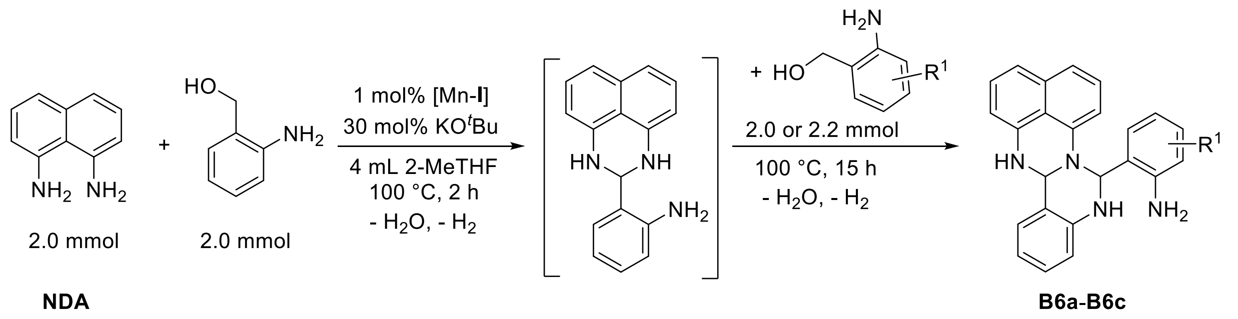

Supplementary Figure 12 Synthesis of amino-fertigines **B6a-B6c**.

10. Synthesis of 7,7a,8,9-tetrahydro-10*H*-imidazo[1,5-*a*]-perimidin-10-ones (C)

General reaction conditions for the synthesis of 7,7a,8,9-tetrahydro-10H-imidazo[1,5-a]-perimidin-10-one derivatives C1-C3: In a glovebox, 2 mmol perimidine derivatives, 30 mol% KO^{t}Bu (0.6 mmol, dissolved in 1.5 mL 1,4-dioxane) and 2.3 mmol carbonyldiimidazol (CDI) are added to a pressure tube and dissolved in 8.5 mL 1,4-dioxane. The sealed pressure tube is heated at 130 °C for 2 hours in an oil bath. After cooling down to room temperature 30 mL water are added and the product is extracted with diethyl ether (4 x 50 mL). The organic layers are dried with Na_2SO_4 and the solvent was removed in vacuo. The crude product is purified via gradient column chromatography using Alox N as stationary phase.

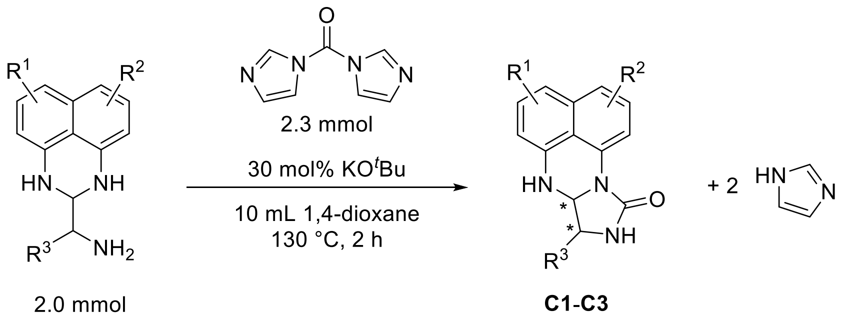

Supplementary Figure 13 Synthesis of 7,7a,8,9-tetrahydro-10*H*-imidazo[1,5-*a*]-perimidin-10-ones **C1-C3**.

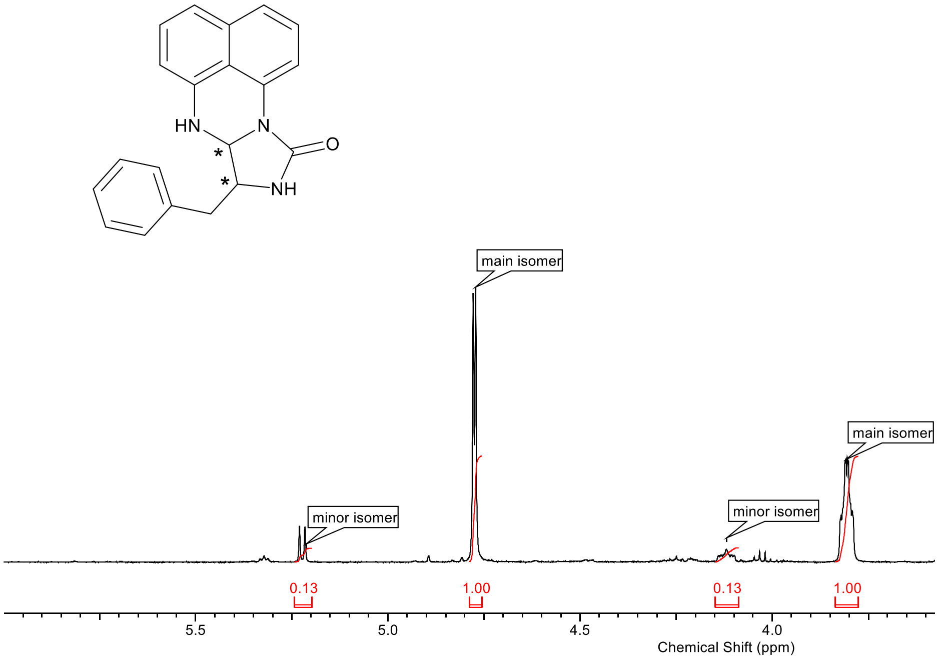

Supplementary Figure 14 Excerpt from ^{1}H NMR-spectrum (500 MHz, 293 K) of the crude **C2** in DMSO-d_6 to determine the diastereomeric ratio based on the integrals of the main and minor isomer.

11. Characterization of fertigines (B)

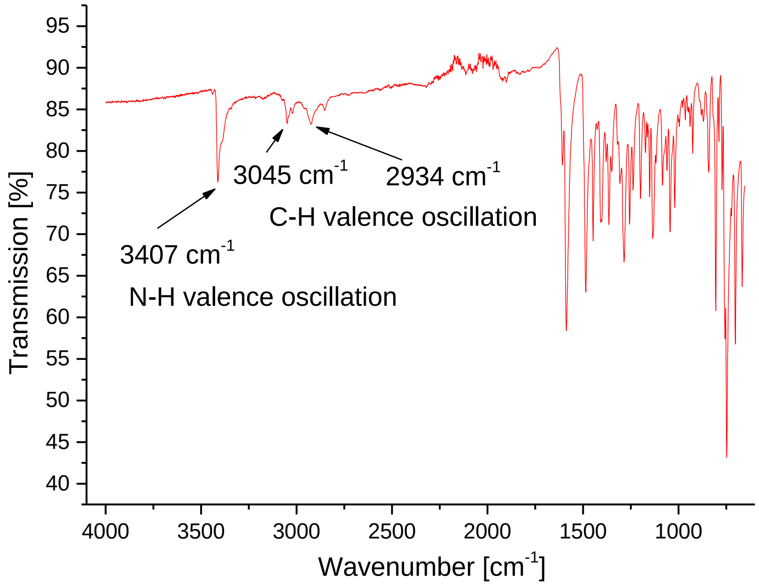

Supplementary Figure 15 IR-spectrum of B1a.

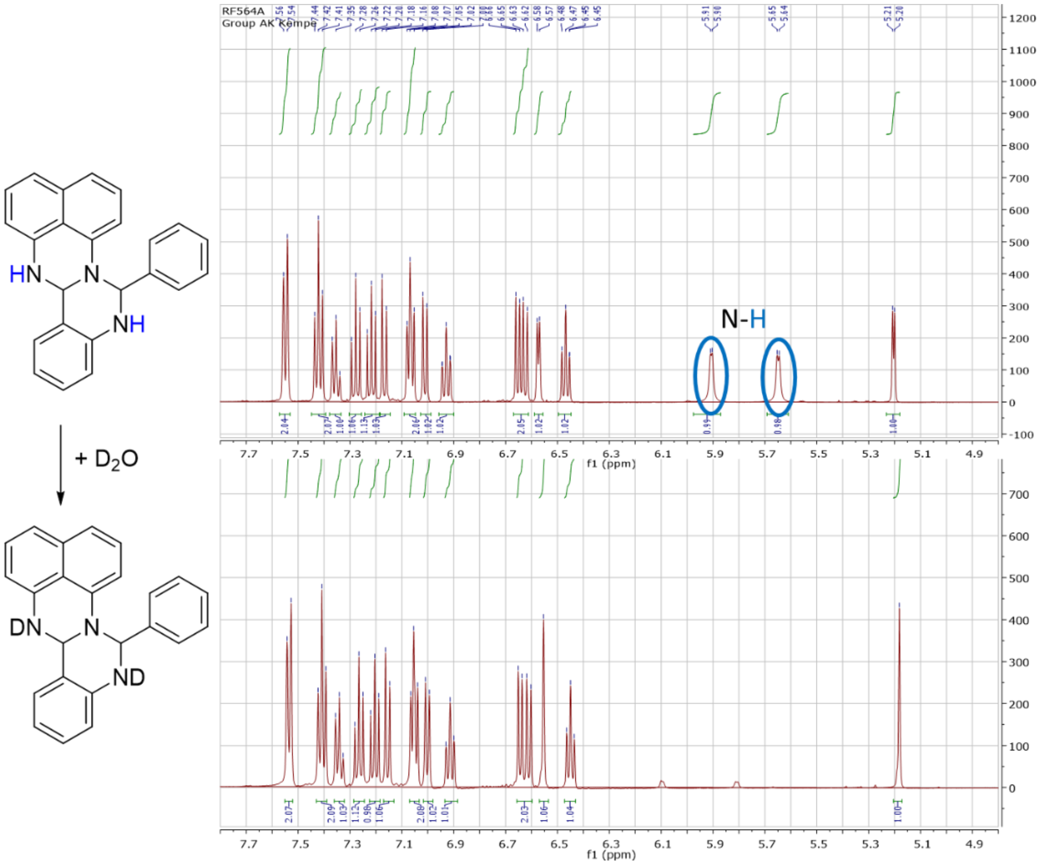

Supplementary Figure 16 ^{1}H NMR (500 MHz, 293 K) of **B1a** in CD_3CN and after the addition of D_2O.

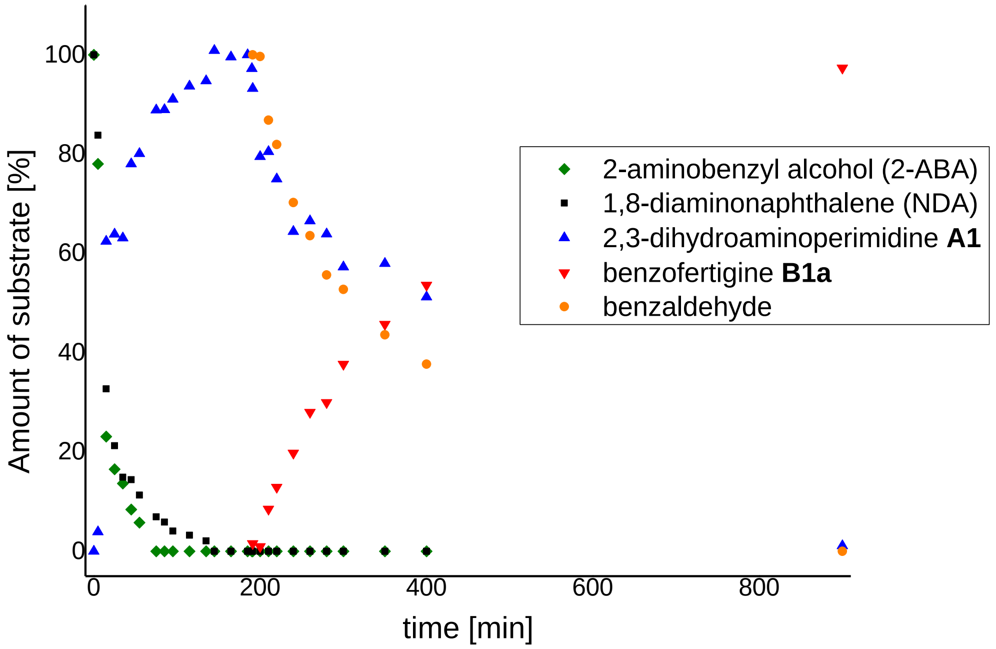

Supplementary Figure 17 Time-conversion plot for the synthesis of fertigine **B1a** (red) over the intermediate product 2,3-dihydroaminoperimidin **A1** (blue)**.** Reaction conditions: 15 mmol NDA (black), 15 mmol 2-aminobenzyl alcohol (green), 4.5 mmol KO^{t}Bu, 0.15 mmol Mn-**I**, 45 mL 2-MeTHF, 100 °C (oil bath). After 190 min. 15 mmol benzaldehyde (orange) is added.

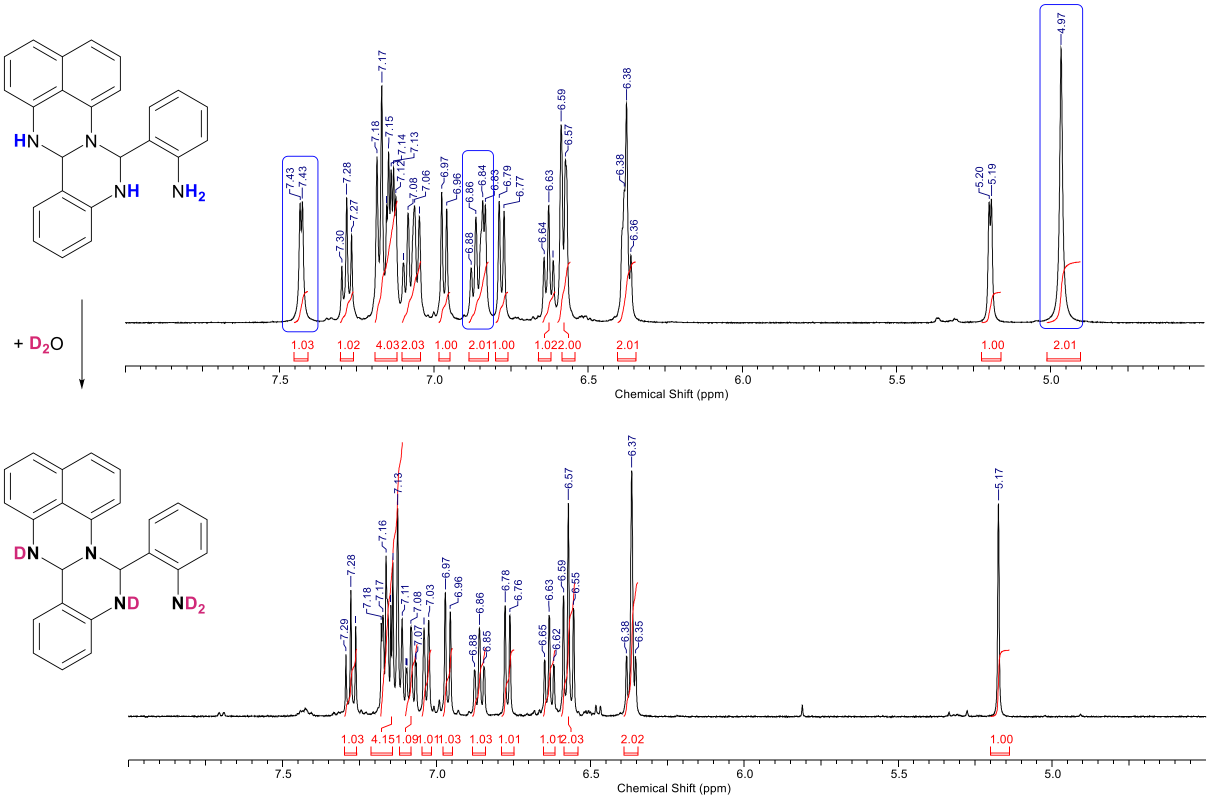

Supplementary Figure 18 ^{1}H NMR (500 MHz, 293 K) of **B6a** in DMSO-d_6 and after the addition of D_2O.

12. Mechanistic investigations

In absence of diaminonaphthalene during catalysis, self-condensation of the aminobenzyl alcohol was observed via ^{1}H NMR analysis. Reaction conditions: 60 µmol 2-aminobenzyl alcohol, 0.6 µmol Mn-**I**, 18 µmol KO^{t}Bu and 700 µL thf-d_8 were heated at 90 °C using an open system for hydrogen release. After 24 h ^{1}H NMR measurement was conducted.

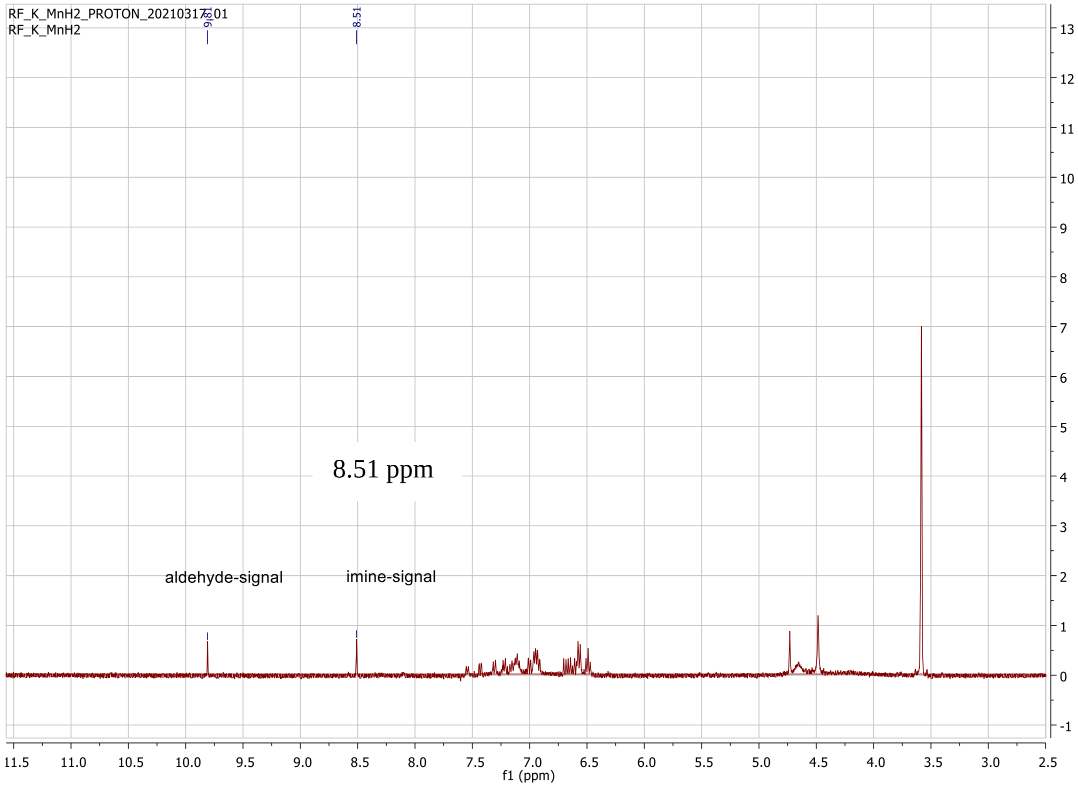

Supplementary Figure 19 ^{1}H NMR spectra showing the self-condensation of the aminobenzyl alcohol in the absence of naphthalene diamine.

Qualitative and quantitative analyses of evolved hydrogen

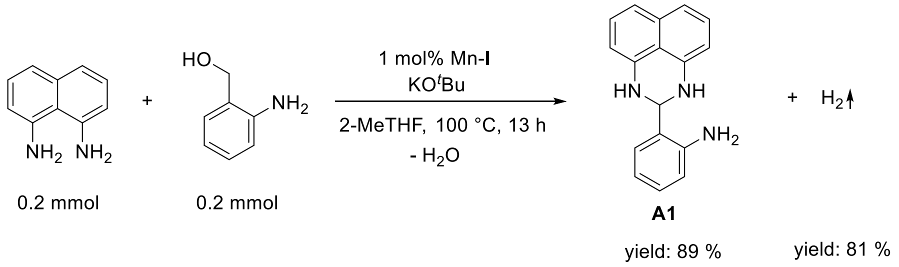

Supplementary Figure 20 Control experiment for the qualitative and quantitative determination of hydrogen.

The release of one equivalent hydrogen was proofed by analyzing the gas mixture with methane as an internal standard in the Schlenk tube after reaction. The gas mixture was analyzed using an Agilent Technologies 6890N equipped with a TCD and an Agilent special plot and molsieve capillary column (30 m, 320 μm, 0.25 μm). Reaction conditions: 0.2 mmol diaminonaphthalene, 0.2 mmol aminobenzyl alcohol, 1 mol% Mn-**I**, 0.06 mmol KO^{t}Bu and 1 ml 2-MeTHF were added to a Schlenk tube (150 mL), closed and heated at 100 °C (oil bath) for 13 h. A yield of 89 % of the perimidine **A1** formed was determined and 81% of hydrogen was detected.

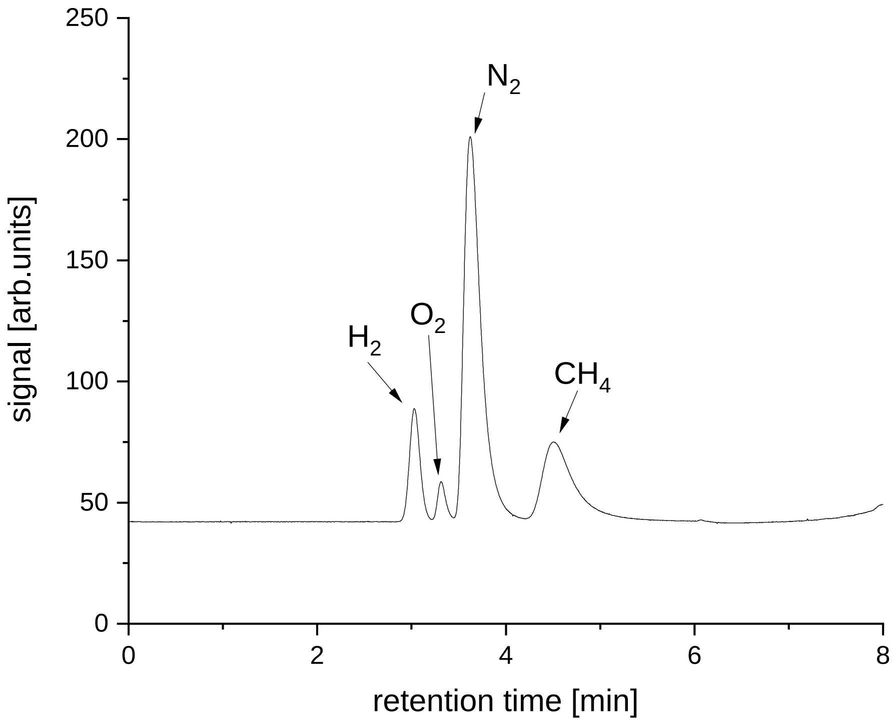

Supplementary Figure 21 Chromatogram of the gas-chromatographic analysis from the upper gas layer over the reaction mixture after 13 h reaction time.

Investigation of the reaction with 2-aminobenzaldehyde via ^{1}H NMR analysis

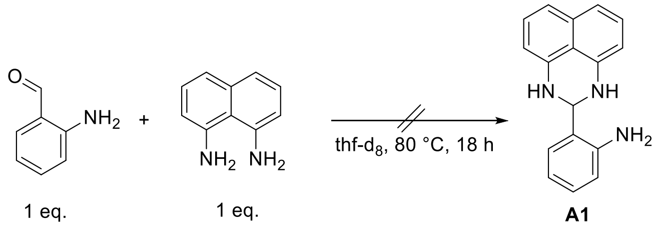

Supplementary Figure 22 Control experiment without KO^{t}Bu.

In absence of KO^{t}Bu no reaction between 2-aminobenzaldehyde and diaminonaphthalene was observed. Reaction conditions: 60 µmol 2-aminobenzaldehyde and 60 µmol diaminonaphthalene were dissolved in 700 µL thf-d_8 and were heated at 80 °C for 18 h. Time-dependant amount of 2-aminobenzaldehyde (referred to the aldehyde signal) and of the diamine (referred to the NH_2-signal) were determined with mesitylene as internal standard.

Control experiment 2: with KO^{t}Bu

Reaction conditions: 120 µmol 2-aminobenzaldehyde, 60 µmol diaminonaphthalene, 9 µmol KO^{t}Bu (15 mol%, stock solution of 30 mg/2 mL thf-d_8), 61 µL stock solution of mesitylene (15 µL / 1 mL thf-d_8), 700 µL thf-d_8 at RT. Time-dependant amount of 2-aminobenzaldehyde (referred to the aldehyde signal) and of the diamine (referred to the NH_2-signal) with mesitylene (2.22 ppm) as internal standard:

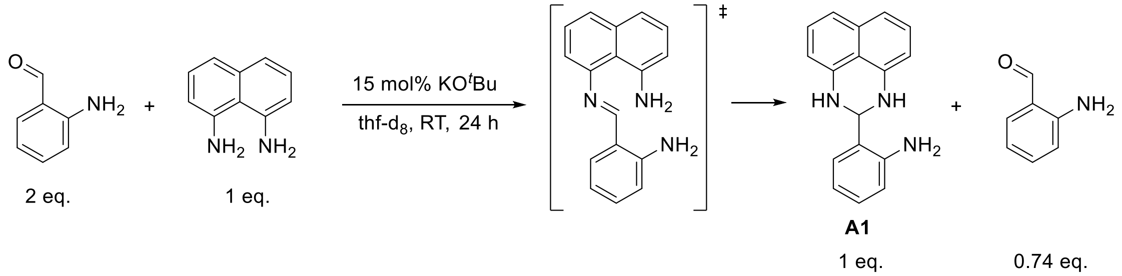

Supplementary Figure 23 Control experiment with KO^{t}Bu.

t = 0 h: 100 % aldehyde (2 eq. compared to 1 eq. diamine), 100 % diamine

t = 0.2 h: 71 % aldehyde (1.4 eq. related to 1 eq. diamine), 38 % diamine

t = 24 h: 37 % aldehyde (0.74 eq. related to 1 eq. diamine), 0 % diamine

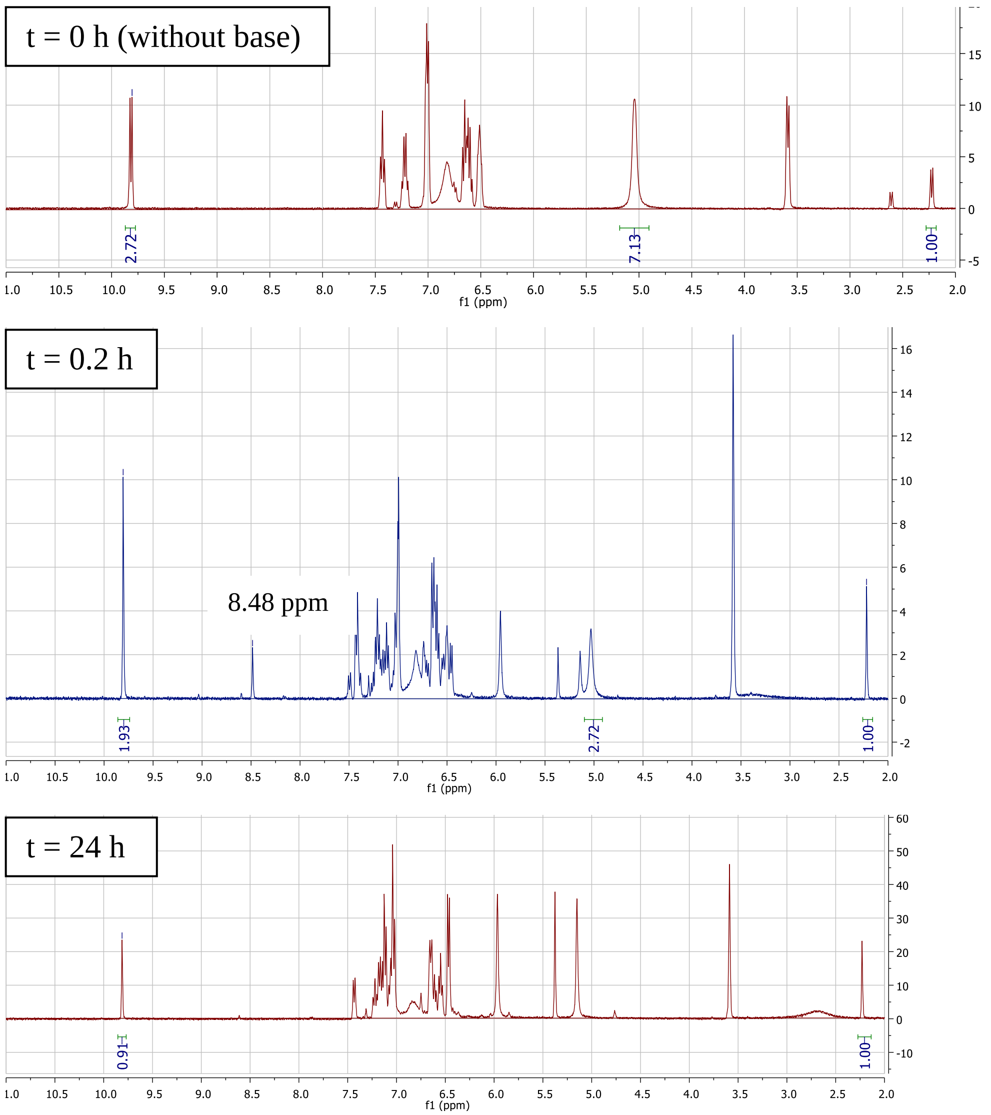

Supplementary Figure 24 Time-dependant ^{1}H NMR studies of the reaction of 2-aminobenzaldehyde with diaminonaphthalene in the presence of KO^{t}Bu (time: before addition of KO^{t}Bu, after 0.2 h and after 24 h).

In addition to the time-dependent consumption of the 2-aminobenzaldehyde, the characteristic imine signal at 8.48 ppm indicates the formation of an imine intermediate, as it differs slightly from the observed imine signal (8.51 ppm) of the self-condensation product of the 2-aminobenzaldehyde in Supplementary Figure 19.

Synthesis and characterisation of A1K

Synthesis of **A1K**: 5 mmol **A1** (1306.7 mg) is dissolved in 30 mL dry thf in a Schlenk tube, 5 mL of a 1 M solution of KO^{t}Bu (5 mmol) in thf is added to the Schlenk tube under argon. A yellow solid precipitate. After 30 min, the thf is filtrated, the yellow solid washed with thf and again filtrated. After drying in vacuo over night the solid is used for further studies.

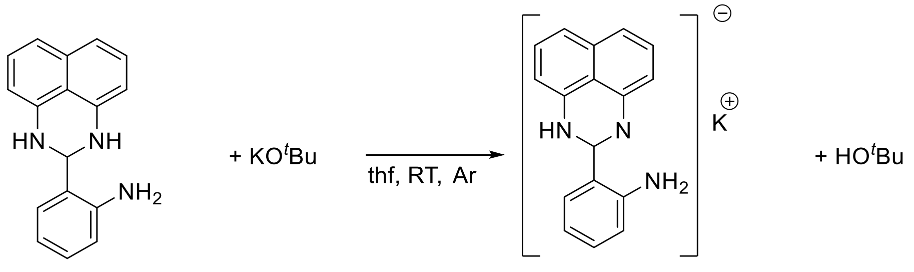

A1 1 eq. | 1 eq. | **A1K**

Supplementary Figure 25 Synthesis of **A1K**.

Characterisation of **A1K**:

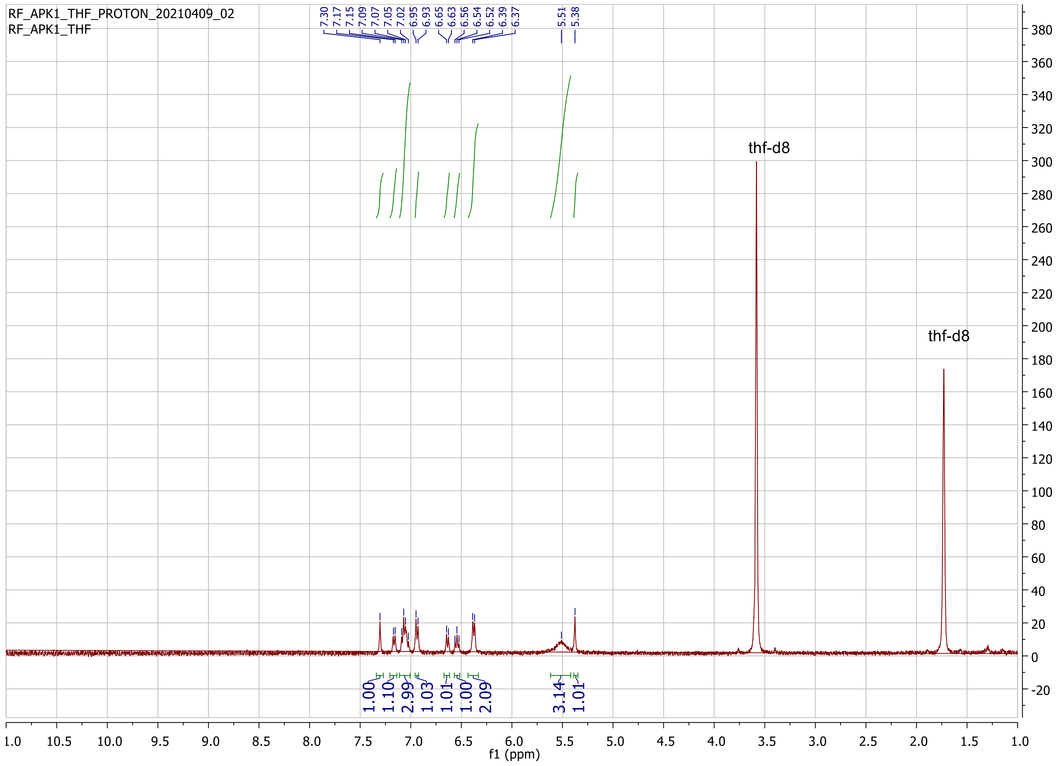

Supplementary Figure 26 ^{1}H NMR of **A1K** (thf-d$_8$, 400 MHz, 293 K): δ = 7.30 (s, 1H), 7.16 (d, J = 7.0 Hz, 1H), 7.06 (dd, J = 16.8, 9.7 Hz, 3H), 6.94 (d, J = 7.8 Hz, 1H), 6.64 (d, J = 7.3 Hz, 1H), 6.54 (t, J = 8.0 Hz, 1H), 6.38 (d, J = 7.6 Hz, 2H), 5.51 (s, 3H), 5.38 (s, 1H) ppm.

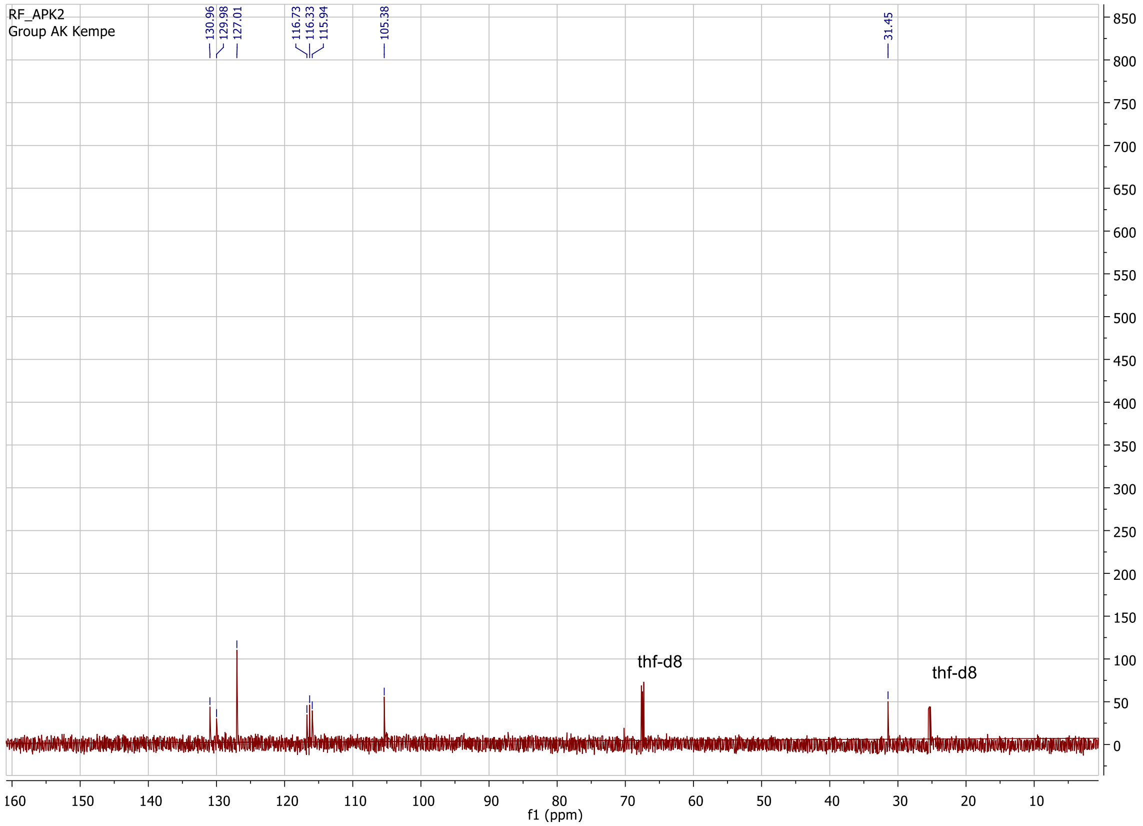

Supplementary Figure 27 ^{13}C NMR of **A1K** (thf-d_8, 125 MHz, 293 K): δ = 130.96, 129.98, 127.01, 116.73, 116.33, 115.94, 105.38, 31.45 ppm.

Elemental analysis calculated (**A1K** + 2 thf): C 68.09, H 7.25, N 9.16

Elemental analysis found: C 68.34, H 6.95, N 9.49

Control experiments with A1K

Addition of D_2O to **A1K** in thf-d_8 in the NMR-tube led to the back reaction of **A1K** to **A1**.

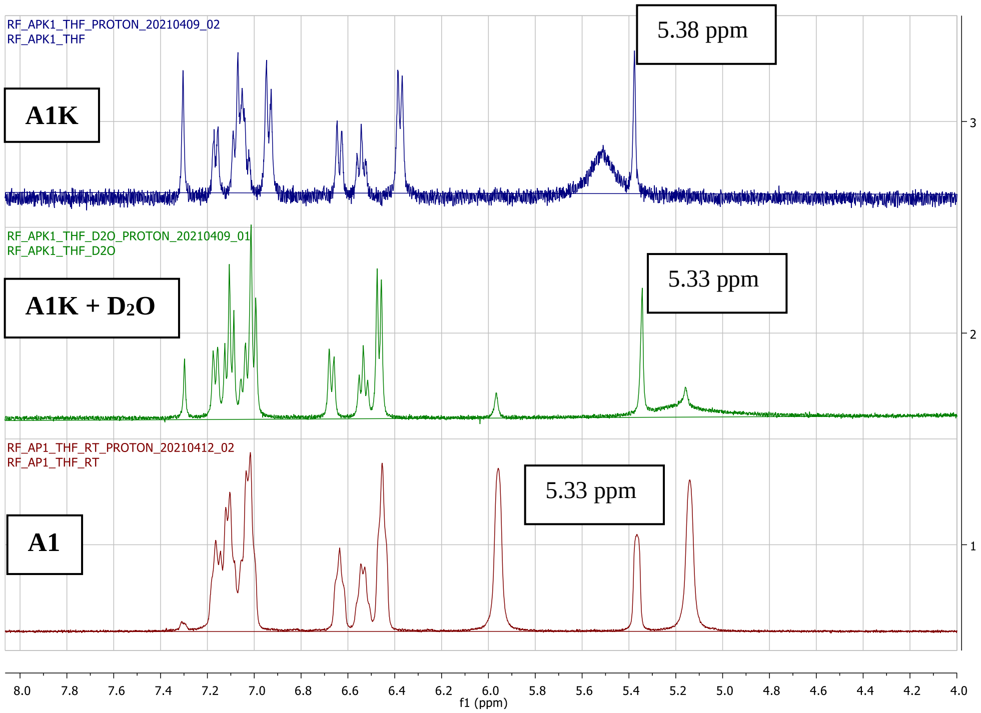

Supplementary Figure 28 Addition of D_2O (green) to **A1K** (blue); the bottom spectra show **A1** (red) as reference.

Additionally, the extraction of **A1K** with ethyl acetate/water led to the isolation of **A1** in the organic phase, while a pH-change of the water phase from 7 to 14 is observed. The use of sodium tetraphenylborate for analyzing the potassium amount in the aqueous phase proofed the formation of 1 eq. KOH per 1 eq. **A1K**. Reaction conditions: 0.2 mmol **A1K** (63 mg) is extracted with ethyl acetate/water. To the combined water phases is added an excess of a solution of $NaB(Ph)_4$. After centrifugation, decantation, and drying in vacuo, 81 mg (0.22 mmol) of a white precipitation of $KB(Ph)_4$ was obtained.

^{1}H NMR control experiments show the reaction of **A1K** to **B1a** after the addition of benzaldehyde:

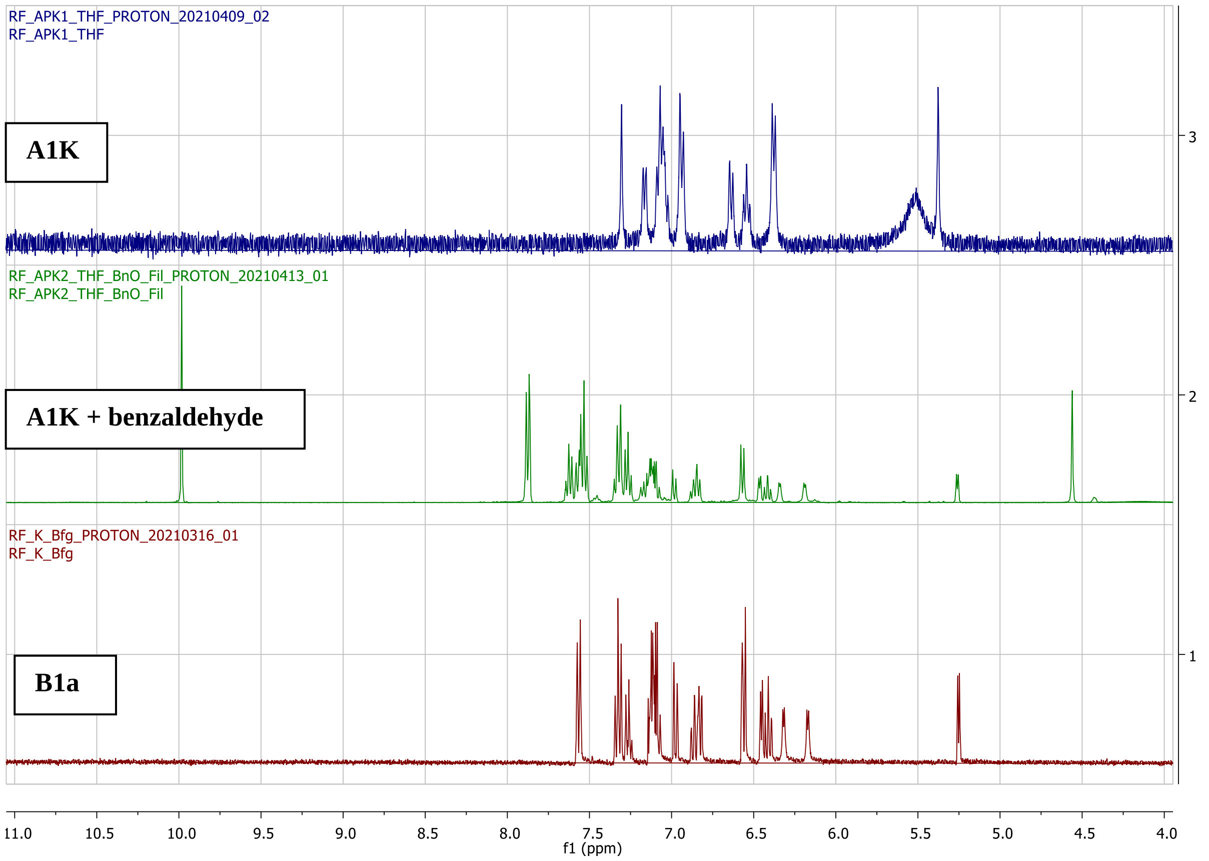

Supplementary Figure 29 ^{1}H NMR of the formation of **B1a** after the addition of benzaldehyde to **A1K** (green). Reaction conditions: To a suspension of 10 mg **A1K** (ca. 31 µmol) in 700 µL thf-d_8 is added 40 µmol benzaldehyde at room temperature. ^{1}H NMR of **A1K** (blue) and **B1a** (red) for reference.

Investigation of the condensation of A1 with benzaldehyde via ^{1}H NMR analysis

Reaction conditions: 60 µmol **A**1, 60 µmol benzaldehyde, 6 µmol KO^{t}Bu (10 mol%, stock solution 30 mg/3 mL thf-d_8), 61 µL stock solution of mesitylene (15 µL / 1 mL thf-d_8), 700 µL thf-d_8 at RT. Without base no reaction is observed (t = 0 h), after addition of base an instant (< 30 s at RT) consumption of the benzaldehyde to 59 % (9.98 ppm), **A1** to 67 % (5.40 ppm) and formation of **B1a** (5.29 ppm) is observed. Mesitylene (2.22 ppm) is used as internal standard.

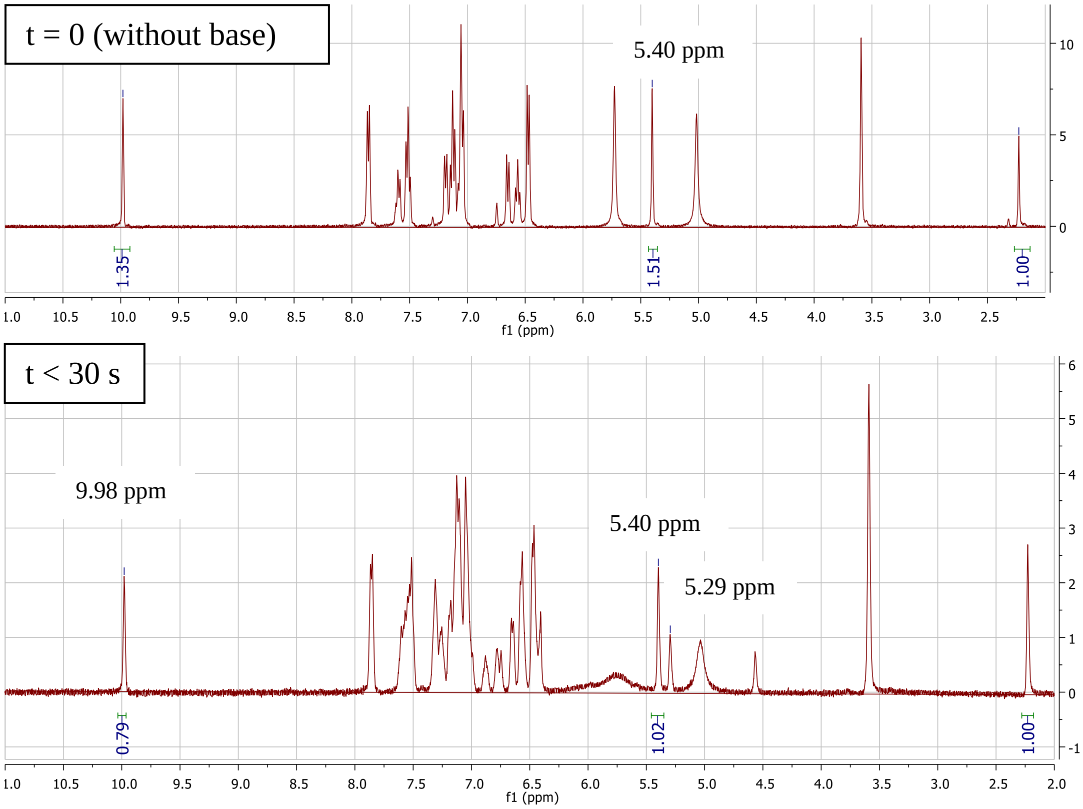

Supplementary Figure 30 ^{1}H NMR spectra showing the instant formation of **B1a** after addition of KO^{t}Bu to a solution of **A1** and benzaldehyde.

13. Isolation and characterization of products

Synthesis of **A1**

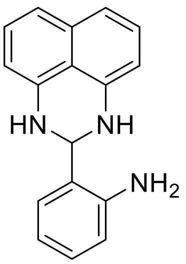

Chemical Formula: $C_{17}H_{15}N_3$

In a glovebox, 1,8-diaminonaphthalene (2 mmol, 316 mg), 2-aminobenzyl alcohol (2 mmol, 247 mg), KO^{t}Bu (0.6 mmol, 67 mg, 30 mol%), Mn-**I** (0.02 mmol, 12 mg, 1 mol%) and 3 mL 2-MeTHF are added to a Schlenk tube. The reaction mixture is heated at 100 °C using an open system consisting of a reflux condenser and a bubble counter. After stirring for 2 h, the mixture is cooled down to room temperature and 2 mL H_2O is added. The aqueous phase is extracted with dichloromethane (3 x 10 mL), the organic layers were dried with Na_2SO_4 and the solvent was removed in vacuo. The crude product was purified via column chromatography over Alox N (pentane/ethyl acetate: 5:1) and obtained as a white solid (470 mg, 1.8 mmol, 90 %).

^{1}H NMR (DMSO-d$_6$, 500 MHz, 293 K): δ = 7.27 (d, J = 7.4 Hz, 1H), 7.15 (t, J = 7.7 Hz, 2H), 7.09 (t, J = 7.6 Hz, 1H), 7.00 (d, J = 7.0 Hz, 2H), 6.72 (d, J = 6.4 Hz, 1H), 6.64 (s, 2H), 6.58 (t, J = 7.2 Hz, 1H), 6.53 (d, J = 5.5 Hz, 2H) ppm.

^{13}C NMR (DMSO-d$_6$, 125 MHz, 293 K): δ = 147.66, 143.93, 134.47, 130.08, 129.19, 126.75, 122.30, 115.75, 115.46, 115.34, 112.70, 104.69, 66.45, 39.52 ppm.

Elemental analysis calculated: C 78.13, H 5.79, N 16.08

Elemental analysis found: C 78.03, H 5.78, N 15.94

Synthesis of **A2**

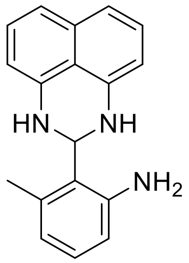

Chemical Formula: $C_{18}H_{17}N_3$

In a glovebox, 1,8-diaminonaphthalene (2 mmol, 316 mg), 2-amino-6-methylbenzyl alcohol (2 mmol, 275 mg), KO^{t}Bu (0.6 mmol, 67 mg, 30 mol%), Mn-**I** (0.02 mmol, 12 mg, 1 mol%) and 3 mL 2-MeTHF are added to a Schlenk tube. The reaction mixture is heated at 100 °C using an open system consisting of a reflux condenser and a bubble counter. After stirring for 2 h, the mixture is cooled down to room temperature and 2 mL H_2O is added. The aqueous phase is extracted with dichloromethane (3 x 10 mL), the organic layers were dried with Na_2SO_4 and the solvent was removed in vacuo. The crude product was purified via column chromatography over Alox N (pentane/ethyl acetate: 5:1) as a grey solid (512 mg, 1.86 mmol, 93 %).

^{1}H NMR (DMSO-d$_6$, 500 MHz, 293 K): δ = 7.15 (t, J = 7.8 Hz, 2H), 7.01 (d, J = 8.1 Hz, 2H), 6.95 (t, J = 7.7 Hz, 1H), 6.64 (s, 2H), 6.55 (d, J = 8.0 Hz, 1H), 6.52 (d, J = 7.4 Hz, 2H), 6.40 (d, J = 7.4 Hz, 1H), 5.65 (s, 1H), 5.50 (s, 2H), 2.31 (s, 3H) ppm.

^{13}C NMR (DMSO-d$_6$, 125 MHz, 293 K): δ = 148.87, 144.34, 134.54, 128.83, 126.67, 119.17, 118.05, 115.41, 114.44, 112.74, 104.93, 63.48, 20.25 ppm.

Elemental analysis calculated: C 78.52, H 6.22, N 15.26

Elemental analysis found: C 77.84, H 5.96, N 15.93

Synthesis of **A3**

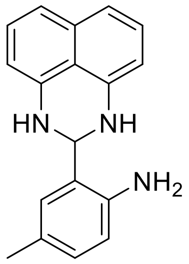

Chemical Formula: $C_{18}H_{17}N_3$

In a glovebox, 1,8-diaminonaphthalene (2 mmol, 316 mg), 2-amino-5-methylbenzyl alcohol (2 mmol, 275 mg), KO^{t}Bu (0.6 mmol, 67 mg, 30 mol%), Mn-**I** (0.02 mmol, 12 mg, 1 mol%) and 3 mL 2-MeTHF are added to a Schlenk tube. The reaction mixture is heated at 100 °C using an open system consisting of a reflux condenser and a bubble counter. After stirring for 2 h, the mixture is cooled down to room temperature and 2 mL H_2O is added. The aqueous phase is extracted with dichloromethane (3 x 10 mL), the organic layers were dried with Na_2SO_4 and the solvent was removed in vacuo. The crude product was purified via column chromatography over Alox N (pentane/ethyl acetate: 5:1) as a grey solid (479 mg, 1.74 mmol, 87 %).

^{1}H NMR (DMSO-d$_6$, 500 MHz, 293 K): δ = 7.14 (t, J = 7.8 Hz, 1H), 7.10 (s, 1H), 6.99 (d, J = 8.1 Hz, 1H), 6.91 (d, J = 8.1 Hz, 1H), 6.62 (d, J = 8.1 Hz, 1H), 6.59 (s, 1H), 6.51 (d, J = 7.4 Hz, 1H), 5.34 (s, 1H), 5.14 (s, 1H), 2.18 (s, 1H) ppm.

^{13}C NMR (DMSO-d$_6$, 125 MHz, 293 K): δ =145.11, 143.97, 134.46, 130.38, 129.63, 126.73, 123.85, 122.39, 116.01, 115.29, 112.67, 104.63, 66.16, 20.10 ppm.

Elemental analysis calculated: C 78.52, H 6.22, N 15.26
Elemental analysis found: C 78.52, H 6.15, N 15.16

Synthesis of **A4**

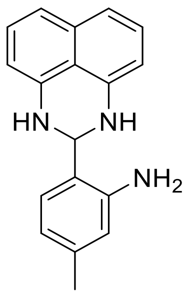

Chemical Formula: $C_{18}H_{17}N_3$

In a glovebox, 1,8-diaminonaphthalene (2 mmol, 316 mg), 2-amino-4-methylbenzyl alcohol (2 mmol, 275 mg), KO^{t}Bu (0.6 mmol, 67 mg, 30 mol%), Mn-**I** (0.02 mmol, 12 mg, 1 mol%) and 3 mL 2-MeTHF are added to a Schlenk tube. The reaction mixture is heated at 100 °C using an open system consisting of a reflux condenser and a bubble counter. After stirring for 2 h, the mixture is cooled down to room temperature and 2 mL H_2O is added. The aqueous phase is extracted with dichloromethane (3 x 10 mL), the organic layers were dried with Na_2SO_4 and the solvent was removed in vacuo. The crude product was purified via column chromatography over Alox N (pentane/ethyl acetate: 5:1) as a grey solid (424 mg, 1.54 mmol, 77 %).

^{1}H NMR (DMSO-d$_6$, 500 MHz, 293 K): δ = 7.14 (t, J = 7.6 Hz, 3H), 6.98 (d, J = 8.1 Hz, 2H), 6.56 (s, 2H), 6.55 – 6.48 (m, 3H), 6.40 (d, J = 7.7 Hz, 1H), 5.33 (s, 1H), 5.27 (s, 2H), 2.19 (s, 3H) ppm.

^{13}C NMR (DMSO-d$_6$, 125 MHz, 293 K): δ = 147.46, 144.00, 138.29, 134.47, 130.05, 126.73, 119.71, 116.37, 116.13, 115.27, 112.70, 104.63, 66.27, 21.00 ppm.

Elemental analysis calculated: C 78.52, H 6.22, N 15.26

Elemental analysis found: C 78.12, H 6.05, N 14.88

Synthesis of **A5**

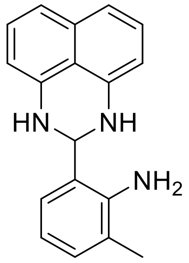

In a glovebox, 1,8-diaminonaphthalene (2 mmol, 316 mg), 2-amino-3-methylbenzyl alcohol (2 mmol, 275 mg), KO^{t}Bu (0.6 mmol, 67 mg, 30 mol%), Mn-**I** (0.02 mmol, 12 mg, 1 mol%) and 3 mL 2-MeTHF are added to a Schlenk tube. The reaction mixture is heated at 100 °C using an open system consisting of a reflux condenser and a bubble counter. After stirring for 2 h, the mixture is cooled down to room temperature and 2 mL H_2O is added. The aqueous phase is extracted with dichloromethane (3 x 10 mL), the organic layers were dried with Na_2SO_4 and the solvent was removed in vacuo. The crude product was purified via column chromatography over Alox N (pentane/ethyl acetate: 5:1) as a grey solid (534 mg, 1.94 mmol, 97 %).

^{1}H NMR (DMSO-d$_6$, 500 MHz, 293 K): δ = 7.14 (dd, J = 15.3, 7.4 Hz, 3H), 7.02 (dd, J = 13.8, 7.8 Hz, 3H), 6.68 (s, 2H), 6.53 (dd, J = 15.2, 7.5 Hz, 3H), 5.37 (s, 1H), 5.19 (s, 2H), 2.12 (s, 3H) ppm.

^{13}C NMR (DMSO-d$_6$, 125 MHz, 293 K): δ = 145.65, 143.96, 134.48, 130.33, 128.37, 126.75, 122.51, 121.74, 115.40, 115.33, 112.73, 104.72, 67.79, 17.72 ppm.

Elemental analysis calculated: C 78.52, H 6.22, N 15.26
Elemental analysis found: C 77.94, H 6.07, N 15.08

Synthesis of **A6**

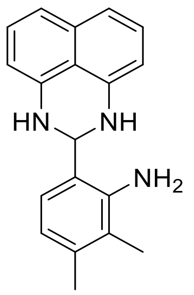

Chemical Formula: $C_{19}H_{19}N_3$

In a glovebox, 1,8-diaminonaphthalene (2 mmol, 316 mg), 2-amino-3,4-dimethylbenzyl alcohol (2 mmol, 303 mg), KO^{t}Bu (0.6 mmol, 67 mg, 30 mol%), Mn-**I** (0.02 mmol, 12 mg, 1 mol%) and 3 mL 2-MeTHF are added to a Schlenk tube. The reaction mixture is heated at 100 °C using an open system consisting of a reflux condenser and a bubble counter. After stirring for 2 h, the mixture is cooled down to room temperature and 2 mL H_2O is added. The aqueous phase is extracted with dichloromethane (3 x 10 mL), the organic layers were dried with Na_2SO_4 and the solvent was removed in vacuo. The crude product was purified via column chromatography over Alox N (pentane/ethyl acetate: 5:1) as a white solid (544 mg, 1.88 mmol, 94 %).

^{1}H NMR (DMSO-d$_6$, 500 MHz, 293 K): δ = 7.15 (t, J = 7.8 Hz, 2H), 7.06 – 6.97 (m, 3H), 6.63 (s, 2H), 6.51 (d, J = 7.4 Hz, 2H), 6.47 (d, J = 7.6 Hz, 1H), 5.32 (s, 1H), 5.15 (s, 2H), 2.22 (s, 3H), 2.02 (s, 3H) ppm.

^{13}C NMR (DMSO-d$_6$, 125 MHz, 293 K): δ = 145.54, 144.00, 136.59, 134.49, 127.56, 126.74, 120.70, 119.93, 117.44, 115.37, 112.74, 104.69, 68.12, 20.46, 12.91 ppm.

Elemental analysis calculated: C 78.86, H 6.62, N 14.52

Elemental analysis found: C 78.17, H 6.32, N 14.19

Synthesis of **A7**

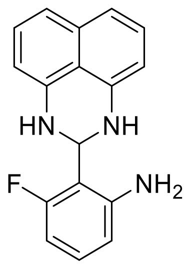

Chemical Formula: $C_{17}H_{14}FN_3$

In a glovebox, 1,8-diaminonaphthalene (2 mmol, 316 mg), 2-amino-6-fluorobenzyl alcohol (2 mmol, 282 mg), KO^{t}Bu (0.6 mmol, 67 mg, 30 mol%), Mn-**I** (0.02 mmol, 12 mg, 1 mol%) and 3 mL 2-MeTHF are added to a Schlenk tube. The reaction mixture is heated at 100 °C using an open system consisting of a reflux condenser and a bubble counter. After stirring for 2 h, the mixture is cooled down to room temperature and 2 mL H_2O is added. The aqueous phase is extracted with dichloromethane (3 x 10 mL), the organic layers were dried with Na_2SO_4 and the solvent was removed in vacuo. The crude product was purified via column chromatography over Alox N (pentane/ethyl acetate: 5:1) as a white solid (530 mg, 1.90 mmol, 95 %).

^{1}H NMR (DMSO-d$_6$, 500 MHz, 293 K): δ = 7.17 (t, J = 7.8 Hz, 1H), 7.09 (dd, J = 14.8, 8.1 Hz, 1H), 7.04 (d, J = 8.1 Hz, 1H), 6.73 (s, 1H), 6.57 – 6.49 (m, 2H), 6.35 (dd, J = 10.4, 8.4 Hz, 1H), 5.81 (s, 1H), 5.70 (s, 1H) ppm.

^{13}C NMR (DMSO-d$_6$, 125 MHz, 293 K): δ = 162.79, 160.87, 150.53, 150.49, 143.95, 134.48, 130.24, 130.14, 126.73, 115.78, 112.76, 111.58, 108.24, 108.14, 105.10, 101.51, 101.32, 59.66, 59.58, 39.52 ppm.

^{19}F NMR (DMSO-d$_6$, 376 MHz, 293 K): δ = -120.53 (dd, J_1 = 10.6 Hz, J_2 = 6.6 Hz) ppm.

Elemental analysis calculated: C 73.10, H 5.05, N 15.04

Elemental analysis found: C 73.19, H 5.15, N 15.04

Synthesis of **A8**

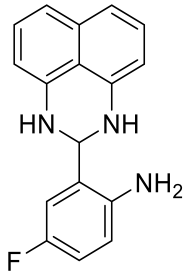

Chemical Formula: $C_{17}H_{14}FN_3$

In a glovebox, 1,8-diaminonaphthalene (2 mmol, 316 mg), 2-amino-5-fluorobenzyl alcohol (2 mmol, 282 mg), KO^{t}Bu (0.6 mmol, 67 mg, 30 mol%), Mn-**I** (0.02 mmol, 12 mg, 1 mol%) and 3 mL 2-MeTHF are added to a Schlenk tube. The reaction mixture is heated at 100 °C using an open system consisting of a reflux condenser and a bubble counter. After stirring for 2 h, the mixture is cooled down to room temperature and 7 mL H_2O is added. After adding 10 mL pentane the precipitation is filtered and washed with pentane. The crude product was purified by filtration with dichloromethane over an Alox N plug and obtained as a white solid (469 mg, 1.68 mmol, 84 %).

^{1}H NMR (DMSO-d$_6$, 500 MHz, 293 K): δ = 7.21 – 7.09 (m, 1H), 7.01 (d, J = 7.2 Hz, 1H), 6.95 (s, 1H), 6.71 (s, 1H), 6.65 (s, 1H), 6.52 (d, J = 6.3 Hz, 1H), 5.41 (s, 1H), 5.23 (s, 1H) ppm.

^{13}C NMR (DMSO-d$_6$, 125 MHz, 293 K): δ = 154.91, 153.08, 143.88, 143.53, 134.41, 126.78, 123.96, 123.91, 116.71, 116.65, 115.69, 115.62, 115.49, 112.62, 104.78, 64.56 ppm.

^{19}F NMR (DMSO-d$_6$, 376 MHz, 293 K): δ = -129.58 (m) ppm.

Elemental analysis calculated: C 73.10, H 5.05, N 15.04

Elemental analysis found: C 73.26, H 5.01, N 14.66

Synthesis of **A9**

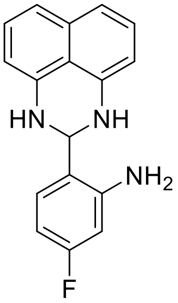

Chemical Formula: $C_{17}H_{14}FN_3$

In a glovebox, 1,8-diaminonaphthalene (2 mmol, 316 mg), 2-amino-4-fluorobenzyl alcohol (2 mmol, 282 mg), KO^{t}Bu (0.6 mmol, 67 mg, 30 mol%), Mn-**I** (0.02 mmol, 12 mg, 1 mol%) and 3 mL 2-MeTHF are added to a Schlenk tube. The reaction mixture is heated at 100 °C using an open system consisting of a reflux condenser and a bubble counter. After stirring for 2 h, the mixture is cooled down to room temperature 7 mL H_2O is added. After adding 10 mL pentane the precipitation is filtered and washed with pentane. The crude product was purified by filtration with dichloromethane over an Alox N plug and obtained as a white solid (491 mg, 1.76 mmol, 88 %).

^{1}H NMR (DMSO-d_6, 500 MHz, 293 K): δ = 7.28 (t, J = 7.6 Hz, 1H), 7.15 (t, J = 7.8 Hz, 2H), 7.00 (d, J = 8.1 Hz, 2H), 6.62 (s, 2H), 6.52 (d, J = 7.3 Hz, 2H), 6.48 (d, J = 11.8 Hz, 1H), 6.35 (t, J = 8.4 Hz, 1H), 5.70 (s, 2H), 5.38 (s, 1H) ppm.

^{13}C NMR (DMSO-d_6, 125 MHz, 293 K): δ = 164.27, 162.35, 149.76, 149.66, 143.87, 134.45, 131.93, 131.84, 126.75, 118.63, 115.44, 112.70, 104.75, 101.60, 101.43, 101.30, 101.11, 65.73 ppm.

^{19}F NMR (DMSO-d_6, 376 MHz, 293 K): δ = -114.25 (dt, J_1 = 11.9 Hz, J_2 = 7.9 Hz) ppm.

Elemental analysis calculated: C 73.10, H 5.05, N 15.04

Elemental analysis found: C 72.99, H 5.07, N 15.12

Synthesis of **A10**

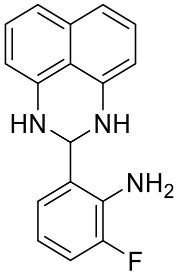

Chemical Formula: $C_{17}H_{14}FN_3$

In a glovebox, 1,8-diaminonaphthalene (2 mmol, 316 mg), 2-amino-3-fluorobenzyl alcohol (2 mmol, 282 mg), KO^{t}Bu (0.6 mmol, 67 mg, 30 mol%), Mn-**I** (0.02 mmol, 12 mg, 1 mol%) and 3 mL 2-MeTHF are added to a Schlenk tube. The reaction mixture is heated at 100 °C using an open system consisting of a reflux condenser and a bubble counter. After stirring for 2 h, the mixture is cooled down to room temperature and 2 mL H_2O is added. The aqueous phase is extracted with dichloromethane (3 x 10 mL), the organic layers were dried with Na_2SO_4 and the solvent was removed in vacuo. The crude product was purified via column chromatography over Alox N (pentane/ethyl acetate: 5:1) as a white solid (508 mg, 1.82 mmol, 91 %).

^{1}H NMR (DMSO-d$_6$, 500 MHz, 293 K): δ = 7.15 (dd, J = 14.0, 6.2 Hz, 3H), 7.08 (dd, J = 11.4, 8.1 Hz, 1H), 7.02 (d, J = 8.2 Hz, 2H), 6.71 (s, 2H), 6.64 – 6.55 (m, 1H), 6.52 (d, J = 7.4 Hz, 2H), 5.45 (s, 1H), 5.33 (s, 2H) ppm.

^{13}C NMR (DMSO-d$_6$, 125 MHz, 293 K): δ = 152.24, 150.36, 143.59, 135.75, 135.64, 134.42, 126.77, 125.63, 125.13, 125.10, 115.55, 115.00, 114.94, 114.87, 114.72, 112.67, 104.81, 65.92 ppm.

^{19}F NMR (DMSO-d$_6$, 376 MHz, 293 K): δ = -135.02 (dd, J_1 = 11.9 Hz, J_2 = 5.3 Hz) ppm.

Elemental analysis calculated: C 73.10, H 5.05, N 15.04

Elemental analysis found: C 72.97, H 5.09, N 14.55

Synthesis of **A11**

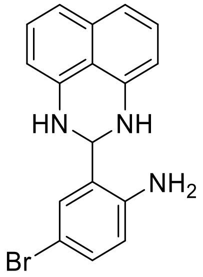

Chemical Formula: $C_{17}H_{14}BrN_3$

In a glovebox, 1,8-diaminonaphthalene (2 mmol, 316 mg), 2-amino-5-bromobenzyl alcohol (2 mmol, 404 mg), KO^{t}Bu (0.6 mmol, 67 mg, 30 mol%), Mn-**I** (0.02 mmol, 12 mg, 1 mol%) and 3 mL 2-MeTHF are added to a Schlenk tube. The reaction mixture is heated at 100 °C using an open system consisting of a reflux condenser and a bubble counter. After stirring for 2 h, the mixture is cooled down to room temperature and 2 mL H_2O is added. The aqueous phase is extracted with dichloromethane (3 x 10 mL), the organic layers were dried with Na_2SO_4 and the solvent was removed in vacuo. The crude product was purified via column chromatography over Alox N (pentane/ethyl acetate: 5:1) as a white solid (640 mg, 1.88 mmol, 94 %).

^{1}H NMR (DMSO-d$_6$, 500 MHz, 293 K): δ = 7.42 (d, J = 2.3 Hz, 1H), 7.22 (dd, J = 8.6, 2.4 Hz, 1H), 7.16 (t, J = 7.8 Hz, 2H), 7.01 (d, J = 8.2 Hz, 2H), 6.71 – 6.63 (m, 3H), 6.52 (d, J = 7.4 Hz, 2H), 5.55 (s, 2H), 5.39 (s, 1H) ppm.

^{13}C NMR (DMSO-d$_6$, 125 MHz, 293 K): δ = 146.85, 143.58, 134.40, 132.04, 131.46, 126.77, 124.61, 117.63, 115.52, 112.61, 105.95, 104.79, 64.77 ppm.

Elemental analysis calculated: C 60.02, H 4.15, N 12.35
Elemental analysis found: C 59.79, H 4.03, N 12.09

Synthesis of **A12**

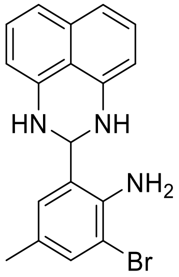

Chemical Formula: $C_{18}H_{16}BrN_3$

In a glovebox, 1,8-diaminonaphthalene (2 mmol, 316 mg), 2-amino-3-bromo-5-methylbenzyl alcohol (2 mmol, 432 mg), KO^{t}Bu (0.6 mmol, 67 mg, 30 mol%), Mn-**I** (0.02 mmol, 12 mg, 1 mol%) and 3 mL 2-MeTHF are added to a Schlenk tube. The reaction mixture is heated at 100 °C using an open system consisting of a reflux condenser and a bubble counter. After stirring for 2 h, the mixture is cooled down to room temperature and 2 mL H_2O is added. The aqueous phase is extracted with dichloromethane (3x10 mL), the organic layers were dried with Na_2SO_4 and the solvent was removed in vacuo. The crude product was purified via column chromatography over Alox N (pentane/ethyl acetate: 5:1) as a yellow solid (503 mg, 1.42 mmol, 71 %).

^{1}H NMR (DMSO-d$_6$, 500 MHz, 293 K): δ = 7.32 (s, 1H), 7.22 – 7.12 (m, 3H), 7.04 (d, J = 8.1 Hz, 2H), 6.74 (s, 2H), 6.54 (d, J = 7.4 Hz, 2H), 5.38 (s, 1H), 5.36 (s, 1H), 2.20 (s, 3H) ppm.

^{13}C NMR (DMSO-d$_6$, 125 MHz, 293 K): δ = 143.56, 142.07, 134.43, 132.60, 130.68, 126.79, 125.64, 124.09, 115.67, 112.70, 109.51, 104.91, 67.38, 19.55 ppm.

Elemental analysis calculated: C 61.03, H 4.55, N 11.86

Elemental analysis found: C 61.01, H 4.55, N 11.53

Synthesis of **A13**

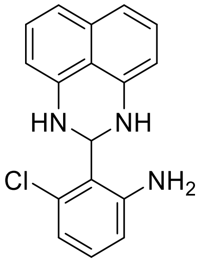

Chemical Formula: $C_{17}H_{14}ClN_3$

In a glovebox, 1,8-diaminonaphthalene (2 mmol, 316 mg), 2-amino-6-chlorobenzyl alcohol (2 mmol, 315 mg), KO^{t}Bu (0.6 mmol, 67 mg, 30 mol%), Mn-**I** (0.02 mmol, 12 mg, 1 mol%) and 3 mL 2-MeTHF are added to a Schlenk tube. The reaction mixture is heated at 100 °C using an open system consisting of a reflux condenser and a bubble counter. After stirring for 2 h, the mixture is cooled down to room temperature and 2 mL H_2O is added. The aqueous phase is extracted with dichloromethane (3 x 10 mL), the organic layers were dried with Na_2SO_4 and the solvent was removed in vacuo. The crude product was purified via column chromatography over Alox N (pentane/ethyl acetate: 5:1→5:3) as a white solid (479 mg, 1.62 mmol, 81 %).

^{1}H NMR (DMSO-d$_6$, 500 MHz, 293 K): δ = 7.17 (t, J = 7.8 Hz, 2H), 7.05 (dd, J = 10.6, 8.2 Hz, 3H), 6.78 (s, 2H), 6.67 (d, J = 8.2 Hz, 1H), 6.62 (d, J = 7.8 Hz, 1H), 6.54 (d, J = 7.3 Hz, 2H), 5.97 – 5.86 (m, 3H) ppm

^{13}C NMR (DMSO-d$_6$, 125 MHz, 293 K): δ = 150.60, 143.87, 134.47, 134.27, 130.17, 126.73, 117.16, 115.97, 115.77, 114.91, 112.69, 105.16, 64.29 ppm.

Elemental analysis calculated: C 69.04, H 4.77, N 14.21

Elemental analysis found: C 68.93, H 4.63 N 13.91

Synthesis of **A14**

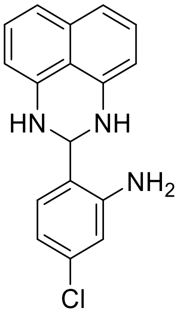

Chemical Formula: $C_{17}H_{14}ClN_3$

In a glovebox, 1,8-diaminonaphthalene (2 mmol, 316 mg), 2-amino-4-chlorobenzyl alcohol (2 mmol, 315 mg), KO^{t}Bu (0.6 mmol, 67 mg, 30 mol%), Mn-**I** (0.02 mmol, 12 mg, 1 mol%) and 3 mL 2-MeTHF are added to a Schlenk tube. The reaction mixture is heated at 100 °C using an open system consisting of a reflux condenser and a bubble counter. After stirring for 2 h, the mixture is cooled down to room temperature 7 mL H_2O is added. After adding 10 mL pentane the precipitation is filtered and washed with pentane. The crude product was purified by filtration with dichloromethane over an Alox N plug and obtained as a white solid (479 mg, 1.62 mmol, 81 %).

^{1}H NMR (DMSO-d$_6$, 500 MHz, 293 K): δ = 7.27 (d, J = 8.0 Hz, 1H), 7.15 (t, J = 7.6 Hz, 2H), 7.01 (d, J = 8.0 Hz, 2H), 6.75 (s, 1H), 6.63 (s, 2H), 6.58 (d, J = 7.6 Hz, 1H), 6.52 (d, J = 7.2 Hz, 2H), 5.68 (s, 2H), 5.38 (s, 1H) ppm.

^{13}C NMR (DMSO-d$_6$, 125 MHz, 293 K): δ = 149.12, 143.71, 134.43, 133.58, 131.66, 126.76, 121.18, 115.49, 114.75, 114.44, 112.69, 104.79, 65.44 ppm.

Elemental analysis calculated: C 69.04, H 4.77, N 14.21

Elemental analysis found: C 68.73, H 4.45, N 13.79

Synthesis of **A15**

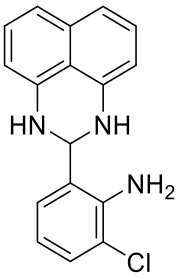

Chemical Formula: $C_{17}H_{14}ClN_3$

In a glovebox, 1,8-diaminonaphthalene (2 mmol, 316 mg), 2-amino-3-chlorobenzyl alcohol (2 mmol, 315 mg), KO^{t}Bu (0.6 mmol, 67 mg, 30 mol%), Mn-**I** (0.02 mmol, 12 mg, 1 mol%) and 3 mL 2-MeTHF are added to a Schlenk tube. The reaction mixture is heated at 100 °C using an open system consisting of a reflux condenser and a bubble counter. After stirring for 2 h, the mixture is cooled down to room temperature and 2 mL H_2O is added. The aqueous phase is extracted with dichloromethane (3 x 10 mL), the organic layers were dried with Na_2SO_4 and the solvent was removed in vacuo. The crude product was purified via column chromatography over Alox N (pentane/ethyl acetate: 5:1) as a white solid (509 mg, 1.72 mmol, 86 %).

^{1}H NMR (DMSO-d$_6$, 500 MHz, 293 K): δ = 7.29 (dd, J = 16.7, 7.7 Hz, 2H), 7.17 (t, J = 7.8 Hz, 2H), 7.03 (d, J = 8.1 Hz, 2H), 6.75 (s, 2H), 6.63 (t, J = 7.7 Hz, 1H), 6.53 (d, J = 7.3 Hz, 2H), 5.59 (s, 2H), 5.43 (s, 1H) ppm.

^{13}C NMR (DMSO-d$_6$, 125 MHz, 293 K): δ = 143.53, 134.42, 129.34, 129.30, 126.78, 124.07, 118.59, 116.03, 115.66, 112.69, 104.91, 67.10, 39.52 ppm.

Elemental analysis calculated: C 69.04, H 4.77, N 14.21

Elemental analysis found: C 68.93, H 4.63, N 13.91

Synthesis of **A16**

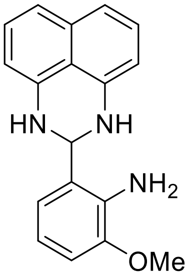

Chemical Formula: $C_{18}H_{17}N_3O$

In a glovebox, 1,8-diaminonaphthalene (2 mmol, 316 mg), 2-amino-3-methoxybenzyl alcohol (2 mmol, 306 mg), KO^{t}Bu (0.6 mmol, 67 mg, 30 mol%), Mn-**I** (0.02 mmol, 12 mg, 1 mol%) and 3 mL 2-MeTHF are added to a Schlenk tube. The reaction mixture is heated at 100 °C using an open system consisting of a reflux condenser and a bubble counter. After stirring for 2 h, the mixture is cooled down to room temperature and 2 mL H_2O is added. The aqueous phase is extracted with dichloromethane (3 x 10 mL), the organic layers were dried with Na_2SO_4 and the solvent was removed in vacuo. The crude product was purified via column chromatography over Alox N (pentane/ethyl acetate: 5:1) as a yellow solid (472 mg, 1.62 mmol, 81 %).

^{1}H NMR (DMSO-d$_6$, 500 MHz, 293 K): δ = 7.14 (t, J = 7.8 Hz, 2H), 6.99 (d, J = 8.1 Hz, 2H), 6.94 (d, J = 7.5 Hz, 1H), 6.89 (d, J = 7.9 Hz, 1H), 6.64 (s, 2H), 6.59 (t, J = 7.8 Hz, 1H), 6.51 (d, J = 7.4 Hz, 2H), 5.40 (s, 1H), 4.99 (s, 2H), 3.81 (s, 3H) ppm.

^{13}C NMR (DMSO-d$_6$, 125 MHz, 293 K): δ = 147.02, 143.83, 136.82, 134.46, 126.74, 122.49, 122.24, 115.36, 115.21, 112.68, 110.75, 104.68, 66.39, 55.75 ppm.

Elemental analysis calculated: C 74.20, H 5.88, N 14.42

Elemental analysis found: C 73.69, H 5.72, N 14.09

Synthesis of **A17**

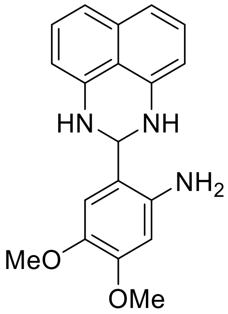

Chemical Formula: $C_{19}H_{19}N_3O_2$

In a glovebox, 1,8-diaminonaphthalene (2 mmol, 316 mg), 2-amino-4,5-dimethoxybenzyl alcohol (2 mmol, 367 mg), KO^{t}Bu (0.6 mmol, 67 mg, 30 mol%), Mn-**I** (0.02 mmol, 12 mg, 1 mol%) and 3 mL 2-MeTHF are added to a Schlenk tube. The reaction mixture is heated at 100 °C using an open system consisting of a reflux condenser and a bubble counter. After stirring for 2 h, the mixture is cooled down to room temperature and 2 mL H_2O is added. The aqueous phase is extracted with dichloromethane (3 x 10 mL), the organic layers were dried with Na_2SO_4 and the solvent was removed in vacuo. The crude product was purified via column chromatography over Alox N (pentane/ethyl acetate: 5:1→5:4) as a white solid (463 mg, 1.44 mmol, 72 %).

^{1}H NMR (DMSO-d$_6$, 500 MHz, 293 K): δ = 7.14 (t, J = 7.8 Hz, 1H), 6.98 (d, J = 8.1 Hz, 1H), 6.94 (s, 1H), 6.51 (d, J = 9.4 Hz, 1H), 6.40 (s, 1H), 5.34 (s, 1H), 4.98 (s, 1H), 3.71 (s, 1H), 3.64 (s, 1H) ppm.

^{13}C NMR (DMSO-d$_6$, 125 MHz, 293 K): δ = 150.17, 144.10, 142.10, 139.79, 134.47, 126.72, 115.42, 115.24, 114.02, 112.69, 104.59, 101.05, 65.06, 56.66, 55.41 ppm.

Elemental analysis calculated: C 71.01, H 5.96, N 13.08
Elemental analysis found: C 70.71, H 5.66, N 12.83

Synthesis of **A18**

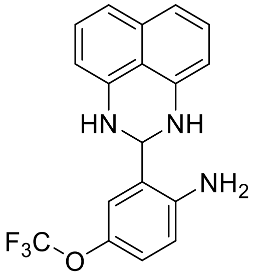

Chemical Formula: $C_{18}H_{14}F_3N_3O$

In a glovebox, 1,8-diaminonaphthalene (2 mmol, 316 mg), 2-amino-5-(trifluoromethoxy)benzyl alcohol (2 mmol, 414 mg), KO^{t}Bu (0.6 mmol, 67 mg, 30 mol%), Mn-**I** (0.02 mmol, 12 mg, 1 mol%) and 3 mL 2-MeTHF are added to a Schlenk tube. The reaction mixture is heated at 100 °C using an open system consisting of a reflux condenser and a bubble counter. After stirring for 2 h, the mixture is cooled down to room temperature and 7 mL H_2O is added. After adding 10 mL pentane the precipitation is filtered and washed with pentane. The crude product was purified by filtration with dichloromethane over an Alox N plug and obtained as a white solid (593 mg, 1.72 mmol, 86 %).

^{1}H NMR (DMSO-d$_6$, 500 MHz, 293 K): δ = 7.28 (d, J = 2.5 Hz, 1H), 7.16 (t, J = 7.8 Hz, 2H), 7.09 (d, J = 8.7 Hz, 1H), 7.01 (d, J = 8.0 Hz, 2H), 6.75 (d, J = 8.8 Hz, 1H), 6.68 (s, 2H), 6.52 (d, J = 7.3 Hz, 2H), 5.59 (s, 2H), 5.42 (s, 1H) ppm.

^{13}C NMR (DMSO-d$_6$, 125 MHz, 293 K): δ = 146.94, 143.61, 143.59, 138.07, 134.40, 126.81, 126.76, 123.01, 122.72, 122.31, 121.43, 119.41, 116.15, 115.56, 112.64, 104.84, 64.87 ppm.

^{19}F NMR (DMSO-d$_6$, 376 MHz, 293 K): δ = -57.24 (s) ppm.

Elemental analysis calculated: C 62.61, H 4.09, N 12.17

Elemental analysis found: C 62.23, H 3.75, N 12.48

Synthesis of **A19**

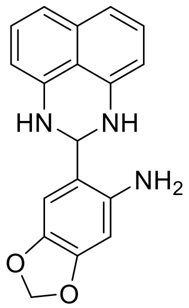

Chemical Formula: $C_{18}H_{15}N_3O_2$

In a glovebox, 1,8-diaminonaphthalene (2 mmol, 316 mg), (6-aminobenzo[d][1,3]dioxol-5-yl)methanol (2 mmol, 335 mg), KO^{t}Bu (0.6 mmol, 67 mg, 30 mol%), Mn-**I** (0.02 mmol, 12 mg, 1 mol%) and 3 mL 2-MeTHF are added to a Schlenk tube. The reaction mixture is heated at 100 °C using an open system consisting of a reflux condenser and a bubble counter. After stirring for 2 h, the mixture is cooled down to room temperature and 7 mL H_2O is added. After adding 10 mL pentane the precipitation is filtered and washed with pentane. The crude product was purified by filtration with dichloromethane over an Alox N plug and obtained as an orange solid (420 mg, 1.38 mmol, 69 %).

^{1}H NMR (DMSO-d$_6$, 500 MHz, 293 K): δ = 7.14 (t, J = 7.8 Hz, 2H), 6.98 (d, J = 8.2 Hz, 2H), 6.87 (s, 1H), 6.54 – 6.48 (m, 4H), 6.37 (s, 1H), 5.85 (s, 2H), 5.33 (s, 1H), 5.09 (s, 2H) ppm.

^{13}C NMR (DMSO-d$_6$, 125 MHz, 293 K): δ = 147.73, 143.96, 142.87, 137.97, 134.45, 126.74, 115.28, 114.69, 112.62, 109.35, 104.62, 100.13, 97.48, 64.85 ppm.

Elemental analysis calculated: C 70.81, H 4.95, N 13.76
Elemental analysis found: C 70.31, H 5.04, N 13.33

Synthesis of **A20**

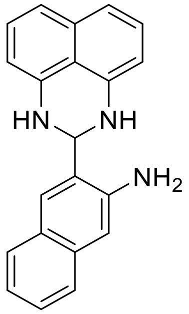

Chemical Formula: $C_{21}H_{17}N_3$

In a glovebox, 1,8-diaminonaphthalene (2 mmol, 316 mg), (3-aminonaphthalen-2-yl)methanol (2 mmol, 346 mg), KO^{t}Bu (0.6 mmol, 67 mg, 30 mol%), Mn-**I** (0.02 mmol, 12 mg, 1 mol%) and 3 mL 2-MeTHF are added to a Schlenk tube. The reaction mixture is heated at 100 °C using an open system consisting of a reflux condenser and a bubble counter. After stirring for 2 h, the mixture is cooled down to room temperature and 7 mL H_2O is added. After adding 10 mL pentane the precipitation is filtered and washed with pentane. The crude product was purified by filtration with dichloromethane over an Alox N plug and obtained as a grey solid (479 mg, 1.54 mmol, 77 %).

^{1}H NMR (DMSO-d$_6$, 500 MHz, 293 K): δ = 7.87 (s, 1H), 7.69 (d, J = 8.1 Hz, 1H), 7.55 (d, J = 8.3 Hz, 1H), 7.32 (t, J = 7.5 Hz, 1H), 7.18 (t, J = 7.8 Hz, 2H), 7.13 (t, J = 7.4 Hz, 1H), 7.03 (d, J = 8.1 Hz, 2H), 7.00 (s, 1H), 6.77 (s, 2H), 6.55 (d, J = 7.3 Hz, 2H), 5.63 (s, 2H), 5.58 (s, 1H) ppm.

^{13}C NMR (DMSO-d$_6$, 125 MHz, 293 K): δ = 145.76, 143.55, 134.80, 134.46, 129.58, 127.76, 126.81, 126.37, 125.93, 124.68, 121.31, 115.55, 112.73, 108.19, 104.84, 66.72 ppm.

Elemental analysis calculated: C 81.00, H 5.50, N 13.49

Elemental analysis found: C 80.94, H 5.42, N 13.13

Synthesis of **A21**

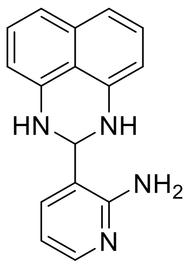

Chemical Formula: $C_{16}H_{14}N_4$

In a glovebox, 1,8-diaminonaphthalene (2 mmol, 316 mg), (2-aminopyridin-3-yl)methanol (2 mmol, 248 mg), KO^{t}Bu (0.6 mmol, 67 mg, 30 mol%), Mn-**I** (0.02 mmol, 12 mg, 1 mol%) and 3 mL 2-MeTHF are added to a Schlenk tube. The reaction mixture is heated at 100 °C using an open system consisting of a reflux condenser and a bubble counter. After stirring for 2 h, the mixture is cooled down to room temperature and 2 mL H_2O is added. The aqueous phase is extracted with dichloromethane (3 x 10 mL), the organic layers were dried with Na_2SO_4 and the solvent was removed in vacuo. The crude product was purified via column chromatography over Alox N (pentane/ethyl acetate: 5:1 →5:3) as a white solid (503 mg, 1.92 mmol, 96 %).

^{1}H NMR (DMSO-d$_6$, 500 MHz, 293 K): δ = 8.01 (s, 1H), 7.60 (d, J = 7.2 Hz, 1H), 7.18 (t, J = 7.7 Hz, 2H), 7.04 (d, J = 8.1 Hz, 2H), 6.72 (s, 2H), 6.64 – 6.58 (m, 1H), 6.55 (d, J = 7.3 Hz, 2H), 6.11 (s, 2H), 5.38 (s, 1H) ppm.

^{13}C NMR (DMSO-d$_6$, 125 MHz, 293 K): δ = 158.08, 148.06, 143.54, 137.79, 134.42, 126.79, 117.43, 115.65, 112.68, 111.93, 104.90, 65.72 ppm.

Elemental analysis calculated: C 73.26, H 5.38, N 21.36

Elemental analysis found: C 73.07, H 5.44, N 21.30

Synthesis of **A22**

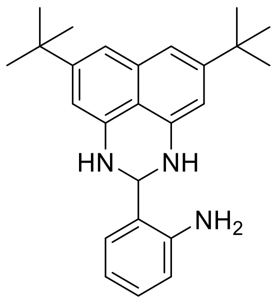

Chemical Formula: $C_{25}H_{31}N_3$

In a glovebox, 1,8-diamino-4,6-di-*tert*-butyl-naphthalene (1 mmol, 270.5 mg), 2-aminobenzyl alcohol (1 mmol, 123 mg), KO^{t}Bu (0.3 mmol, 3 mg, 30 mol%), Mn-**I** (0.01 mmol, 12 mg, 1 mol%) and 3 mL 2-MeTHF are added to a Schlenk tube. The reaction mixture is heated at 100 °C using an open system consisting of a reflux condenser and a bubble counter. After stirring for 6 h, the mixture is cooled down to room temperature and 2 mL H_2O is added. The aqueous phase is extracted with ethyl acetate (3 x 10 mL), the organic layers were dried with Na_2SO_4 and the solvent was removed in vacuo. The crude product was purified via column chromatography over Alox N (pentane/ethyl acetate: 5:1 →5:3) and obtained as a white solid (283 mg, 0.76 mmol, 76 %).

^{1}H NMR (DMSO-d_6, 500 MHz, 293 K): δ = 7.24 (d, J = 6.6 Hz, 1H), 7.07 (t, J = 7.6 Hz, 1H), 6.94 (d, J = 1.4 Hz, 1H), 6.70 (d, J = 7.9 Hz, 1H), 6.59 (d, J = 1.4 Hz, 1H), 6.38 (s, 1H), 5.36 (s, 1H), 5.35 (s, 1H), 1.29 (s, 1H) ppm.

^{13}C NMR (DMSO-d_6, 125 MHz, 293 K): δ = 149.00, 147.71, 143.20, 134.12, 130.09, 129.11, 122.39, 115.72, 115.40, 111.32, 109.94, 102.88, 34.48, 31.23 ppm.

Elemental analysis calculated: C 80.39, H 8.37 N 11.25

Elemental analysis found: C 79.99, H 8.31, N 11.50

Synthesis of **A23**

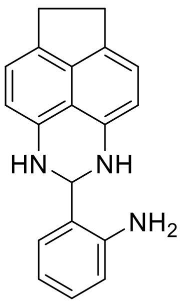

Chemical Formula: $C_{19}H_{17}N_3$

In a glovebox, 5,6-acenaphthenediamine (1 mmol, 184.3 mg), 2-aminobenzyl alcohol (1 mmol, 123 mg), KO*t*Bu (0.3 mmol, 3 mg, 30 mol%), Mn-**I** (0.01 mmol, 12 mg, 1 mol%) and 3 mL 2-MeTHF are added to a Schlenk tube. The reaction mixture is heated at 100 °C using an open system consisting of a reflux condenser and a bubble counter. After stirring for 6 h, the mixture is cooled down to room temperature and 2 mL H_2O is added. The aqueous phase is extracted with ethyl acetate (3 x 10 mL), the organic layers were dried with Na_2SO_4 and the solvent was removed in vacuo. The crude product was purified via column chromatography over Alox N (pentane/ethyl acetate: 5:3) and obtained as a yellow solid (247 mg, 0.86 mmol, 86 %).

^{1}H NMR (DMSO-d_6, 500 MHz, 293 K): δ = 7.24 (d, J = 7.5 Hz, 1H), 7.08 (t, J = 7.7 Hz, 1H), 6.95 (d, J = 7.3 Hz, 1H), 6.70 (d, J = 8.1 Hz, 1H), 6.56 (t, J = 7.4 Hz, 1H), 6.42 (d, J = 7.2 Hz, 1H), 6.38 (s, 1H), 5.34 (s, 1H), 5.30 (s, 1H), 3.20 (s, 1H) ppm.

^{13}C NMR (DMSO-d_6, 125 MHz, 293 K): δ = 147.64, 140.59, 139.82, 132.13, 130.04, 129.06, 122.59, 119.58, 115.71, 115.43, 111.47, 105.29, 67.77, 29.96 ppm.

Elemental analysis calculated (product + 1 ethyl acetate): C 73.57, H 6.71, N 11.19
Elemental analysis found: C 73.86, H 6.32, N 11.11

Synthesis of **A24**

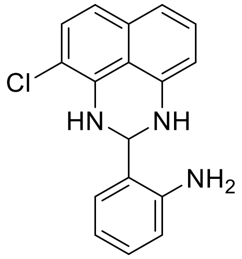

Chemical Formula: $C_{17}H_{14}ClN_3$

In a glovebox, 2-choro-1,8-diamino-naphthalene (1 mmol, 192.6 mg), 2-aminobenzyl alcohol (1 mmol, 123 mg), KO^{t}Bu (0.3 mmol, 3 mg, 30 mol%), Mn-**I** (0.01 mmol, 12 mg, 1 mol%) and 3 mL 2-MeTHF are added to a Schlenk tube. The reaction mixture is heated at 100 °C using an open system consisting of a reflux condenser and a bubble counter. After stirring for 6 h, the mixture is cooled down to room temperature and 2 mL H_2O is added. The aqueous phase is extracted with ethyl acetate (3 x 10 mL), the organic layers were dried with Na_2SO_4 and the solvent was removed in vacuo. The crude product was purified via column chromatography over Alox N (pentane/ethyl acetate: 5:2) and obtained as a grey solid (269 mg, 0.91 mmol, 91%).

^{1}H NMR (DMSO-d$_6$, 500 MHz, 293 K): δ = 7.24 (dd, J = 17.3, 8.3 Hz, 2H), 7.07 (dd, J = 14.2, 8.5 Hz, 2H), 6.91 (s, 1H), 6.72 (dd, J = 8.0, 0.9 Hz, 1H), 6.67 – 6.62 (m, 1H), 6.56 (td, J = 7.5, 1.0 Hz, 1H), 5.81 (s, 1H), 5.55 (s, 1H), 5.32 (s, 1H) ppm.

^{13}C NMR (DMSO-d$_6$, 125 MHz, 293 K): δ = 147.06, 142.82, 138.39, 132.90, 129.07, 128.87, 127.47, 127.01, 122.89, 116.97, 115.92, 115.81, 115.53, 112.85, 107.98, 105.97, 64.31 ppm.

Elemental analysis calculated: C 69.04, H 4.77, N 14.21
Elemental analysis found: C 69.12, H 4.52, N 14.34

Synthesis of **A25**

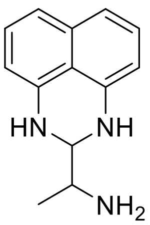

Chemical Formula: $C_{13}H_{15}N_3$

In a glovebox, Mn-precatalyst Mn-**I** (0.02 mmol, 12.3 mg, dissolved in 1.5 mL 1,4-dioxane), KO^tBu (0.6 mmol, 67 mg, dissolved in 1.5 mL 1,4-dioxane), 1,8-diaminonaphthalene (2.0 mmol, 316 mg) and L-alaninol (2.2 mmol, 165 mg, 172 µL) are added to a Schlenk tube and dissolved in 9 mL 1,4-dioxane. The reaction mixture is heated at 100 °C under light argon counter flow using an open system consisting of a reflux condenser and a bubble counter. The mixture is stirred for 4 h, cooled down to room temperature and the 1,4-dioxane is evaporated under vacuo. 6 mL water are added and the organic compounds were extracted with ethyl acetate (3 x 50 mL). The combined organic layers were dried with Na_2SO_4 and the solvent was removed in vacuo. The crude product was purified via gradient column chromatography over Alox N using solvent mixtures beginning with ethyl acetate/pentane 1:1 and switching to ethanol/pentane 1:2. To the product 10 mL of an aqueous saturated solution of $NaHCO_3$ were added, the product was extracted with ethyl acetate and after drying with Na_2SO_4 the solution was narrowed. At the end the product was purified via column chromatography over Silica C18 ec with ethyl acetate and obtained as brown viscous oil (392 mg, 1.84 mmol, 92 %).

^{1}H NMR (DMSO-d_6, 500 MHz, 293 K): δ = 7.11 (t, J = 7.6 Hz, 2H), 6.91 (d, J = 8.2 Hz, 2H), 6.47 (dd, J_1 = 7.5 Hz, J_2 = 0.8 Hz, 1H), 6.45 (dd, J_1 = 7.4 Hz, J_2 = 0.7 Hz, 1H), 6.32 (s, 1H), 6.21 (s, 1H), 4.09 (d, J = 4.4 Hz, 1H), 2.94 – 2.89 (m, 1H), 1.79 (s, broad, 2H), 1.09 (d, J = 6.6 Hz, 3H) ppm.

^{13}C NMR (DMSO-d_6, 125 MHz, 293 K): δ = 143.02, 142.91, 134.40, 126.89, 114.76, 114.71, 112.46, 104.01, 68.85, 49.64, 17.85 ppm.

LC-HRMS (ESI+) *m/z* calculated for $[C_{13}H_{16}N_3]^+$: 214.13387, found: 214.13420.

Synthesis of **A26**

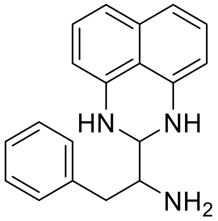

Chemical Formula: $C_{19}H_{19}N_3$

In a glovebox, Mn-precatalyst Mn-**I** (0.02 mmol, 12.3 mg, dissolved in 1.5 mL 1,4-dioxane), KO^tBu (0.6 mmol, 67 mg, dissolved in 1.5 mL 1,4-dioxane), 1,8-diaminonaphthalene (2.0 mmol, 316 mg) and L-phenylalaninol (2.2 mmol, 333 mg) are added to a Schlenk tube and dissolved in 9 mL 1,4-dioxane. The reaction mixture is heated at 100 °C under light argon counter flow using an open system consisting of a reflux condenser and a bubble counter. The mixture is stirred for 4 h, cooled down to room temperature and the 1,4-dioxane is evaporated under vacuo. 6 mL water are added and the organic compounds were extracted with ethyl acetate (3 x 50 mL). The combined organic layers were dried with Na_2SO_4 and the solvent was removed in vacuo. The crude product was purified via gradient column chromatography over Alox N using solvent mixtures beginning with ethyl acetate/pentane 1:1 and switching to ethanol/pentane 1:2. To the product 10 mL of an aqueous saturated solution of $NaHCO_3$ were added, the product was extracted with ethyl acetate and after drying with Na_2SO_4 the solution was narrowed. At the end the product was purified via column chromatography over Silica C18 ec with ethyl acetate and obtained as yellowish brown viscous oil (544 mg, 1.88 mmol, 94 %).

^{1}H NMR (DMSO-d_6, 500 MHz, 293 K): δ = 7.31 – 7.27 (m, 4H), 7.21 – 7.17 (m, 1H), 7.14 (td, J_1 = 7.8 Hz, J_2 = 1.5 Hz, 2H), 6.94 (d, J = 8.2 Hz, 2 H), 6.51 (m, 2H), 6,44 (s, 1H), 6.35 (s, 1H), 4.24 (d, J = 3.8 Hz, 1H), 3.11 – 3.02 (m, 2H), 2.56 – 2.51 (m, 1H), 1.53 (s, broad, 2H) ppm.

^{13}C NMR (DMSO-d_6, 125 MHz, 293 K): δ = 143.05, 142.98, 140.38, 134.43, 129.25, 128.19, 126.91, 125.80, 114.92, 112.51, 104.24, 104.16, 67.74, 55.90, 37.60 ppm.

LC-HRMS (ESI+) *m/z* calculated for $[C_{19}H_{20}N_3]^+$: 290.16517, found: 290.16551.

Synthesis of **A27**

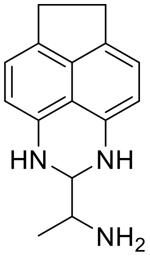

Chemical Formula: $C_{15}H_{17}N_3$

In a glovebox, Mn-precatalyst Mn-**I** (0.02 mmol, 12.3 mg, dissolved in 1.5 mL 1,4-dioxane), KO^{t}Bu (0.6 mmol, 67 mg, dissolved in 1.5 mL 1,4-dioxane), 5,6-diaminoacenaphthene (2.0 mmol, 369 mg) and L-alaninol (2.2 mmol, 165 mg, 172 µL) are added to a Schlenk tube and dissolved in 9 mL 1,4-dioxane. The reaction mixture is heated at 100 °C under light argon counter flow using an open system consisting of a reflux condenser and a bubble counter. The mixture is stirred for 4 h, cooled down to room temperature and the 1,4-dioxane is evaporated under vacuo. 6 mL water are added and the organic compounds were extracted with ethyl acetate (3 x 50 mL). The combined organic layers were dried with Na_2SO_4 and the solvent was removed in vacuo. The crude product was purified via column chromatography over Alox N using solvent mixtures beginning with ethyl acetate/pentane 1:1 and switching to ethanol/pentane 1:2. To the product 10 mL of an aqueous saturated solution of $NaHCO_3$ were added, the product was extracted with ethyl acetate and after drying with Na_2SO_4 the solution was narrowed. At the end the product was purified via column chromatography over Silica C18 ec with ethyl acetate and obtained as brown viscous oil (435 mg, 1.82 mmol, 91 %).

^{1}H NMR (DMSO-d$_6$, 500 MHz, 293 K): δ = 6.92 (d, J = 7.2 Hz, 2H), 6.39 (d, J = 7.3 Hz, 1H), 6.37 (d, J = 7.2 Hz, 1H), 6.07 (s, 1H), 5.97 (s, 1H), 4.05 (d, J = 4.3 Hz, 1H), 3.16 (s, 4H), 2.93 (m, 1H), 1.68 (s, broad, 2H), 1.10 (d, J = 6.4 Hz, 3H) ppm.

^{13}C NMR (DMSO-d$_6$, 125 MHz, 293 K): δ = 139.83, 139.78, 139.72, 131.60, 131.55, 119.69, 111.45, 104.71, 70.20, 49.58, 29.90, 18.09 ppm.

LC-HRMS (ESI+) *m/z* calculated for $[C_{15}H_{18}N_3]^+$: 240.14952, found: 240.14922.

Synthesis of **B1a**

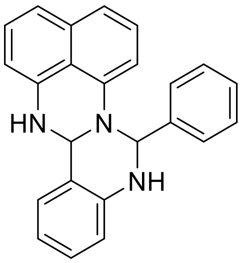

Chemical Formula: $C_{24}H_{19}N_3$

In a glovebox, 1,8-diaminonaphthalene (2 mmol, 316 mg), 2-aminobenzyl alcohol (2 mmol, 247 mg), KO^{t}Bu (0.6 mmol, 67 mg, 30 mol%), Mn-**I** (0.02 mmol, 12 mg, 1 mol%) and 3 mL 2-MeTHF are added to a Schlenk tube. The reaction mixture is heated at 100 °C using an open system consisting of a reflux condenser and a bubble counter. After 2 hours reaction time, benzaldehyde (2 mmol, 203 µl) is diluted in 0.5 mL 2-MeTHF and added with a syringe to the reaction mixture via a septum. After stirring for 15 h, the mixture is cooled down to room temperature and 2 mL H_2O is added. The aqueous phase is extracted with dichloromethane (3 x 10 mL), the organic layers were dried with Na_2SO_4 and the solvent was removed in vacuo. The crude product was purified via column chromatography over Alox N (pentane/ethyl acetate: 5:1) and obtained as a white solid (648 mg, 1.86 mmol, 93 %).

^{1}H NMR (DMSO-d$_6$, 500 MHz, 293 K): δ = 7.50 (d, J = 7.6 Hz, 2H), 7.43 (t, J = 7.5 Hz, 2H), 7.36 – 7.31 (m, 2H), 7.24 (t, J = 7.9 Hz, 1H), 7.16 (t, J = 7.7 Hz, 1H), 7.10 (d, J = 8.1 Hz, 1H), 7.06 (dd, J = 7.3, 4.0 Hz, 2H), 6.98 – 6.94 (m, 2H), 6.87 (t, J = 7.6 Hz, 1H), 6.64 – 6.55 (m, 3H), 6.38 (t, J = 7.4 Hz, 1H), 5.09 (d, J = 3.4 Hz, 1H) ppm.

^{13}C NMR (DMSO-d$_6$, 125 MHz, 293 K): δ = 143.20, 142.38, 141.15, 139.97, 134.28, 128.60, 127.95, 127.74, 126.94, 126.90, 126.62, 125.37, 121.62, 117.87, 115.46, 115.33, 113.78, 113.33, 105.59, 105.30, 65.48, 60.00 ppm.

Elemental analysis calculated: C 82.49, H 5.48, N 12.03

Elemental analysis found: C 82.68, H 5.39, N 11.99

Synthesis of **B1b**

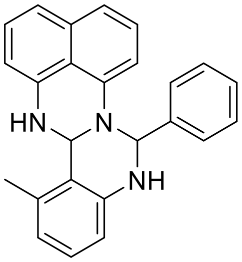

Chemical Formula: $C_{25}H_{21}N_3$

In a glovebox, 1,8-diaminonaphthalene (2 mmol, 316 mg), 2-amino-6-methylbenzyl alcohol (2 mmol, 275 mg), KO^{t}Bu (0.6 mmol, 67 mg, 30 mol%), Mn-**I** (0.02 mmol, 12 mg, 1 mol%) and 3 mL 2-MeTHF are added to a Schlenk tube. The reaction mixture is heated at 100 °C using an open system consisting of a reflux condenser and a bubble counter. After 2 hours reaction time, benzaldehyde (2 mmol, 203 µL) is diluted in 0.5 mL 2-MeTHF and added with a syringe to the reaction mixture via a septum. After stirring for 15 h, the mixture is cooled down to room temperature and 2 mL H_2O is added. The aqueous phase is extracted with dichloromethane (3 x 10 mL), the organic layers were dried with Na_2SO_4 and the solvent was removed in vacuo. The crude product was purified via column chromatography over Alox N (pentane/ethyl acetate: 5:0.5) and obtained as a yellow solid (669 mg, 1.84 mmol, 92 %).

^{1}H NMR (DMSO-d$_6$, 500 MHz, 293 K): δ = 7.31 – 7.19 (m, 7H), 7.08 (d, J = 8.0 Hz, 1H), 7.01 – 6.91 (m, 2H), 6.76 (s, 1H), 6.73 (s, 1H), 6.68 (d, J = 7.4 Hz, 1H), 6.59 (d, J = 8.0 Hz, 1H), 6.46 (d, J = 7.4 Hz, 1H), 6.03 (d, J = 7.4 Hz, 1H), 5.63 (s, 1H), 5.47 (s, 1H), 2.31 (s, 3H) ppm.

^{13}C NMR (DMSO-d$_6$, 125 MHz, 293 K): δ = 143.51, 142.45, 141.59, 141.15, 135.69, 134.41, 128.65, 128.38, 128.26, 128.03, 126.72, 125.51, 120.74, 118.64, 117.44, 117.06, 115.59, 114.64, 112.24, 105.12, 68.11, 64.12, 18.40 ppm.

Elemental analysis calculated: C 82.61, H 5.82, N 11.56

Elemental analysis found: C 82.11, H 5.81, N 11.45

Synthesis of **B1c**

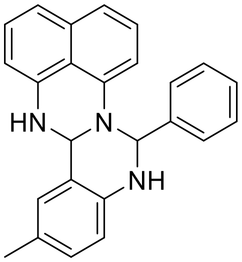

Chemical Formula: $C_{25}H_{21}N_3$

In a glovebox, 1,8-diaminonaphthalene (2 mmol, 316 mg), 2-amino-5-methylbenzyl alcohol (2 mmol, 275 mg), KO^{t}Bu (0.6 mmol, 67 mg, 30 mol%), Mn-**I** (0.02 mmol, 12 mg, 1 mol%) and 3 mL 2-MeTHF are added to a Schlenk tube. The reaction mixture is heated at 100 °C using an open system consisting of a reflux condenser and a bubble counter. After 2 hours reaction time, benzaldehyde (2 mmol, 203 µL) is diluted in 0.5 mL 2-MeTHF and added with a syringe to the reaction mixture via a septum. After stirring for 15 h, the mixture is cooled down to room temperature and 2 mL H_2O is added. The aqueous phase is extracted with dichloromethane (3 x 10 mL), the organic layers were dried with Na_2SO_4 and the solvent was removed in vacuo. The crude product was purified via column chromatography over Alox N (pentane/ethyl acetate: 5:0.5) and obtained as a yellow solid (603 mg, 1.66 mmol, 83 %).

^{1}H NMR (DMSO-d$_6$, 500 MHz, 293 K): δ = 7.49 (d, J = 7.6 Hz, 2H), 7.41 (t, J = 7.6 Hz, 2H), 7.33 (t, J = 7.3 Hz, 1H), 7.29 (d, J = 3.6 Hz, 1H), 7.24 (t, J = 7.9 Hz, 1H), 7.17 (t, J = 7.8 Hz, 1H), 7.10 (d, J = 8.2 Hz, 1H), 7.03 (d, J = 7.7 Hz, 1H), 6.96 (d, J = 8.1 Hz, 1H), 6.89 (s, 1H), 6.76 (d, J = 4.5 Hz, 1H), 6.70 (d, J = 8.0 Hz, 1H), 6.57 (d, J = 7.4 Hz, 1H), 6.55 – 6.50 (m, 2H), 5.07 (d, J = 3.6 Hz, 1H), 2.01 (s, 3H) ppm.

^{13}C NMR (DMSO-d$_6$, 125 MHz, 293 K): δ = 142.40, 141.22, 140.81, 140.03, 134.29, 128.60, 128.55, 127.67, 126.95, 126.63, 125.67, 123.89, 121.71, 117.74, 115.25, 113.65, 105.52, 105.20, 65.48, 60.03, 20.35 ppm.

Elemental analysis calculated: C 82.61, H 5.82, N 11.56
Elemental analysis found: C 82.83 H 5.83, N 11.47

Synthesis of **B1d**

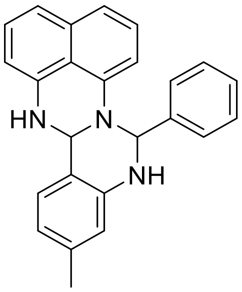

Chemical Formula: $C_{25}H_{21}N_3$

In a glovebox, 1,8-diaminonaphthalene (2 mmol, 316 mg), 2-amino-4-methylbenzyl alcohol (2 mmol, 275 mg), KO^{t}Bu (0.6 mmol, 67 mg, 30 mol%), Mn-**I** (0.02 mmol, 12 mg, 1 mol%) and 3 mL 2-MeTHF are added to a Schlenk tube. The reaction mixture is heated at 100 °C using an open system consisting of a reflux condenser and a bubble counter. After 2 hours reaction time, benzaldehyde (2 mmol, 203 µL) is diluted in 0.5 mL 2-MeTHF and added with a syringe to the reaction mixture via a septum. After stirring for 15 h, the mixture is cooled down to room temperature and 2 mL H_2O is added. The aqueous phase is extracted with dichloromethane (3 x 10 mL), the organic layers were dried with Na_2SO_4 and the solvent was removed in vacuo. The crude product was purified via column chromatography over Alox N (pentane/ethyl acetate: 5:0.5) and obtained as a yellow solid (596 mg, 1.64 mmol, 82 %).

^{1}H NMR (DMSO-d$_6$, 500 MHz, 293 K): δ = 7.50 (d, J = 7.6 Hz, 2H), 7.42 (t, J = 7.5 Hz, 2H), 7.34 (t, J = 7.2 Hz, 1H), 7.30 – 7.21 (m, 2H), 7.15 (t, J = 7.7 Hz, 1H), 7.10 (d, J = 8.2 Hz, 1H), 7.05 (d, J = 7.8 Hz, 1H), 6.95 (t, J = 7.7 Hz, 2H), 6.86 (d, J = 4.3 Hz, 1H), 6.59 – 6.52 (m, 2H), 6.41 (s, 1H), 6.19 (d, J = 7.7 Hz, 1H), 5.06 (d, J = 3.2 Hz, 1H), 2.05 (s, 3H) ppm.

^{13}C NMR (DMSO-d$_6$, 125 MHz, 293 K): δ = 143.00, 142.36, 141.23, 140.02, 137.02, 134.29, 128.58, 127.70, 126.96, 126.87, 126.60, 125.36, 118.95, 117.81, 116.53, 115.28, 113.82, 113.76, 105.60, 105.27, 65.50, 59.95, 20.92 ppm.

Elemental analysis calculated: C 82.61, H 5.82, N 11.56
Elemental analysis found: C 82.96 H 5.82, N 11.70

Synthesis of **B1e**

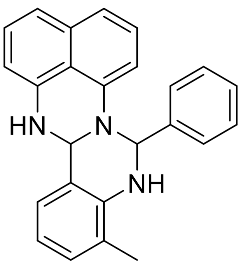

Chemical Formula: $C_{25}H_{21}N_3$

In a glovebox, 1,8-diaminonaphthalene (2 mmol, 316 mg), 2-amino-3-methylbenzyl alcohol (2 mmol, 275 mg), KO^{t}Bu (0.6 mmol, 67 mg, 30 mol%), Mn-**I** (0.02 mmol, 12 mg, 1 mol%) and 3 mL 2-MeTHF are added to a Schlenk tube. The reaction mixture is heated at 100 °C using an open system consisting of a reflux condenser and a bubble counter. After 2 hours reaction time, benzaldehyde (2 mmol, 203 µL) is diluted in 0.5 mL 2-MeTHF and added with a syringe to the reaction mixture via a septum. After stirring for 15 h, the mixture is cooled down to room temperature and 2 mL H_2O is added. The aqueous phase is extracted with dichloromethane (3 x 10 mL), the organic layers were dried with Na_2SO_4 and the solvent was removed in vacuo. The crude product was purified via column chromatography over Alox N (pentane/ethyl acetate: 5:0.5) and obtained as a yellow solid (567 mg, 1.56 mmol, 78 %).

^{1}H NMR (DMSO-d$_6$, 500 MHz, 293 K): δ = 7.51 (d, J = 7.6 Hz, 2H), 7.43 (t, J = 7.6 Hz, 2H), 7.34 (t, J = 6.1 Hz, 2H), 7.25 (t, J = 7.9 Hz, 1H), 7.16 (t, J = 7.8 Hz, 1H), 7.10 (d, J = 8.1 Hz, 1H), 7.06 (d, J = 7.7 Hz, 1H), 6.96 (t, J = 8.4 Hz, 2H), 6.78 (d, J = 7.3 Hz, 1H), 6.60 (d, J = 5.0 Hz, 1H), 6.57 (d, J = 7.3 Hz, 1H), 6.34 (dd, J = 14.4, 6.6 Hz, 2H), 5.12 (d, J = 3.7 Hz, 1H), 2.10 (s, 3H) ppm.

^{13}C NMR (DMSO-d$_6$, 125 MHz, 293 K): δ = 142.26, 141.16, 141.00, 139.97, 134.28, 128.99, 128.61, 127.73, 126.94, 126.91, 126.68, 123.15, 121.55, 121.03, 117.81, 115.33, 115.23, 113.72, 105.29, 105.23, 65.48, 60.02, 17.22 ppm.

Elemental analysis calculated: C 82.61, H 5.82, N 11.56
Elemental analysis found: C 81.99 H 5.81, N 11.22

Synthesis of **B1f**

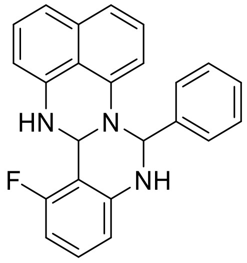

Chemical Formula: $C_{24}H_{18}FN_3$

In a glovebox, 1,8-diaminonaphthalene (2 mmol, 316 mg), 2-amino-6-fluorobenzyl alcohol (2 mmol, 282 mg), KO^{t}Bu (0.6 mmol, 67 mg, 30 mol%), Mn-**I** (0.02 mmol, 12 mg, 1 mol%) and 3 mL 2-MeTHF are added to a Schlenk tube. The reaction mixture is heated at 100 °C using an open system consisting of a reflux condenser and a bubble counter. After 2 hours reaction time, benzaldehyde (2 mmol, 203 µL) is diluted in 0.5 mL 2-MeTHF and added with a syringe to the reaction mixture via a septum. After stirring for 15 h, the mixture is cooled down to room temperature and 2 mL H_2O is added. The aqueous phase is extracted with dichloromethane (3 x 10 mL), the organic layers were dried with Na_2SO_4 and the solvent was removed in vacuo. The crude product was purified via column chromatography over Alox N (pentane/ethyl acetate: 5:0.5) and obtained as a yellow solid (639 mg, 1.73 mmol, 87 %).

^{1}H NMR (DMSO-d$_6$, 500 MHz, 293 K): δ = 7.41 – 7.29 (m, 6H), 7.20 (t, J = 7.2 Hz, 2H), 7.13 (t, J = 7.8 Hz, 1H), 7.04 (d, J = 8.1 Hz, 1H), 6.97 (dd, J = 14.6, 7.8 Hz, 1H), 6.82 (s, 1H), 6.61 (t, J = 7.7 Hz, 2H), 6.51 (d, J = 8.1 Hz, 1H), 6.30 – 6.21 (m, 1H), 6.20 (d, J = 2.5 Hz, 1H), 5.43 (s, 1H) ppm.

^{13}C NMR (DMSO-d$_6$, 125 MHz, 293 K): δ = 161.66, 159.73, 145.53, 145.47, 141.06, 141.00, 140.83, 134.22, 129.36, 129.27, 128.48, 128.15, 127.63, 126.90, 126.15, 119.46, 115.61, 114.92, 109.82, 109.74, 107.48, 107.35, 105.18, 102.61, 102.43, 66.43, 60.08 ppm.

^{19}F NMR (DMSO-d$_6$, 376 MHz, 293 K): δ = -119.89 (s) ppm.

Elemental analysis calculated: C 78.45, H 4.94, N 11.44

Elemental analysis found: C 77.82, H 5.01, N 11.55

Synthesis of **B1g**

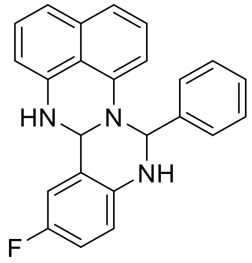

Chemical Formula: $C_{24}H_{18}FN_3$

In a glovebox, 1,8-diaminonaphthalene (2 mmol, 316 mg), 2-amino-5-fluorobenzyl alcohol (2 mmol, 282 mg), KO^{t}Bu (0.6 mmol, 67 mg, 30 mol%), Mn-**I** (0.02 mmol, 12 mg, 1 mol%) and 3 mL 2-MeTHF are added to a Schlenk tube. The reaction mixture is heated at 100 °C using an open system consisting of a reflux condenser and a bubble counter. After 2 hours reaction time, benzaldehyde (2 mmol, 203 µL) is diluted in 0.5 mL 2-MeTHF and added with a syringe to the reaction mixture via a septum. After stirring for 15 h, the mixture is cooled down to room temperature and 2 mL H_2O is added. The aqueous phase is extracted with dichloromethane (3 x 10 mL), the organic layers were dried with Na_2SO_4 and the solvent was removed in vacuo. The crude product was purified via column chromatography over Alox N (pentane/ethyl acetate: 5:0.5) and obtained as a yellow solid (602 mg, 1.64 mmol, 82 %).

^{1}H NMR (DMSO-d$_6$, 500 MHz, 293 K): δ = 7.50 (d, J = 7.5 Hz, 2H), 7.43 (t, J = 7.6 Hz, 2H), 7.39 – 7.32 (m, 2H), 7.26 (t, J = 7.9 Hz, 1H), 7.18 (t, J = 7.8 Hz, 1H), 7.13 (d, J = 8.1 Hz, 1H), 7.09 (d, J = 7.7 Hz, 1H), 6.99 (d, J = 8.0 Hz, 1H), 6.95 (d, J = 4.6 Hz, 1H), 6.91 (dd, J = 9.4, 2.8 Hz, 1H), 6.74 (td, J = 8.7, 2.9 Hz, 1H), 6.62 (dd, J = 9.1, 5.6 Hz, 3H), 5.06 (d, J = 3.7 Hz, 1H) ppm.

^{13}C NMR (DMSO-d$_6$, 125 MHz, 293 K): δ = 154.85, 153.01, 142.18, 140.84, 139.73, 139.60, 134.25, 128.67, 127.79, 126.94, 126.91, 126.68, 122.99, 122.95, 118.05, 115.64, 114.89, 114.71, 114.46, 114.41, 113.71, 111.94, 111.76, 105.82, 105.49, 65.52, 59.83 ppm.

^{19}F NMR (DMSO-d$_6$, 376 MHz, 293 K): δ = -128.59 (m) ppm.

Elemental analysis calculated: C 78.45, H 4.94, N 11.44
Elemental analysis found: C 78.81, H 4.47, N 11.30

Synthesis of **B1h**

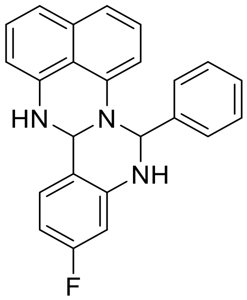

Chemical Formula: $C_{24}H_{18}FN_3$

In a glovebox, 1,8-diaminonaphthalene (2 mmol, 316 mg), 2-amino-4-fluorobenzyl alcohol (2 mmol, 282 mg), KO^{t}Bu (0.6 mmol, 67 mg, 30 mol%), Mn-**I** (0.02 mmol, 12 mg, 1 mol%) and 3 mL 2-MeTHF are added to a Schlenk tube. The reaction mixture is heated at 100 °C using an open system consisting of a reflux condenser and a bubble counter. After 2 hours reaction time, benzaldehyde (2 mmol, 203 µL) is diluted in 0.5 mL 2-MeTHF and added with a syringe to the reaction mixture via a septum. After stirring for 15 h, the mixture is cooled down to room temperature and 2 mL H_2O is added. The aqueous phase is extracted with dichloromethane (3 x 10 mL), the organic layers were dried with Na_2SO_4 and the solvent was removed in vacuo. The crude product was purified via column chromatography over Alox N (pentane/ethyl acetate: 5:0.5) and obtained as a yellow solid (625 mg, 1.70 mmol, 85 %).

^{1}H NMR (DMSO-d$_6$, 500 MHz, 293 K): δ = 7.49 (d, J = 7.5 Hz, 2H), 7.44 (t, J = 7.6 Hz, 2H), 7.39 – 7.30 (m, 2H), 7.26 (t, J = 7.9 Hz, 2H), 7.20 – 7.11 (m, 2H), 7.08 (d, J = 7.7 Hz, 1H), 7.07 – 7.02 (m, 1H), 6.98 (d, J = 8.1 Hz, 1H), 6.64 (d, J = 4.0 Hz, 1H), 6.57 (d, J = 7.3 Hz, 1H), 6.39 (dd, J = 11.3, 2.4 Hz, 1H), 6.16 (td, J = 8.7, 2.4 Hz, 1H), 5.05 (d, J = 3.6 Hz, 1H) ppm.

^{13}C NMR (DMSO-d$_6$, 125 MHz, 293 K): δ = 163.30, 161.39, 144.86, 144.77, 141.97, 140.88, 139.67, 134.27, 128.68, 127.87, 127.14, 127.06, 126.94, 126.88, 126.62, 118.15, 117.71, 115.52, 113.77, 105.79, 105.47, 101.86, 101.68, 99.29, 99.09, 65.49, 59.73 ppm.

^{19}F NMR (DMSO-d$_6$, 376 MHz, 293 K): δ = -114.85 (m) ppm.

Elemental analysis calculated: C 78.45, H 4.94, N 11.44

Elemental analysis found: C 77.88, H 4.99, N 11.28

Synthesis of **B1i**

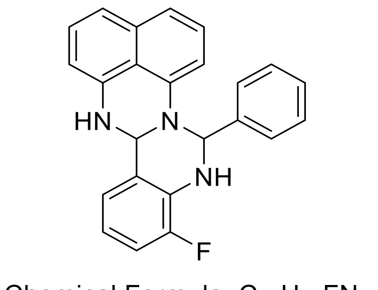

Chemical Formula: $C_{24}H_{18}FN_3$

In a glovebox, 1,8-diaminonaphthalene (2 mmol, 316 mg), 2-amino-3-fluorobenzyl alcohol (2 mmol, 282 mg), KO^{t}Bu (0.6 mmol, 67 mg, 30 mol%), Mn-**I** (0.02 mmol, 12 mg, 1 mol%) and 3 mL 2-MeTHF are added to a Schlenk tube. The reaction mixture is heated at 100 °C using an open system consisting of a reflux condenser and a bubble counter. After 2 hours reaction time, benzaldehyde (2 mmol, 203 µL) is diluted in 0.5 mL 2-MeTHF and added with a syringe to the reaction mixture via a septum. After stirring for 15 h, the mixture is cooled down to room temperature and 2 mL H_2O is added. The aqueous phase is extracted with dichloromethane (3 x 10 mL), the organic layers were dried with Na_2SO_4 and the solvent was removed in vacuo. The crude product was purified via column chromatography over Alox N (pentane/ethyl acetate: 5:0.5) and obtained as a yellow solid (580 mg, 1.58 mmol, 79 %).

^{1}H NMR (DMSO-d$_6$, 500 MHz, 293 K): δ = 7.51 (d, J = 7.5 Hz, 2H), 7.45 (t, J = 7.6 Hz, 2H), 7.40 (d, J = 3.9 Hz, 1H), 7.36 (t, J = 7.2 Hz, 1H), 7.27 (t, J = 7.9 Hz, 1H), 7.20 – 7.11 (m, 2H), 7.07 (d, J = 7.7 Hz, 1H), 6.98 (d, J = 8.1 Hz, 1H), 6.92 (d, J = 7.2 Hz, 2H), 6.84 (t, 1H), 6.63 (d, J = 4.7 Hz, 1H), 6.58 (d, J = 7.3 Hz, 1H), 6.37 (dd, J = 12.9, 7.9 Hz, 1H), 5.11 (d, J = 3.8 Hz, 1H) ppm.

^{13}C NMR (DMSO-d$_6$, 125 MHz, 293 K): δ = 150.57, 148.67, 141.74, 140.71, 139.56, 134.25, 131.33, 131.23, 128.72, 127.90, 126.98, 126.86, 126.65, 124.64, 121.10, 118.23, 115.53, 114.56, 114.51, 113.73, 113.66, 113.59, 105.57, 105.51, 65.10, 59.58 ppm.

^{19}F NMR (DMSO-d$_6$, 376 MHz, 293 K): δ = -136.37 (dd, J_1 = 11.9 Hz, J_2 = 5.3 Hz) ppm.

Elemental analysis calculated: C 78.45, H 4.94, N 11.44

Elemental analysis found: C 77.99, H 5.06, N 11.39

Synthesis of **B1j**

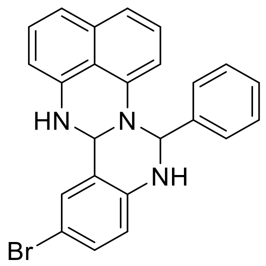

Chemical Formula: $C_{24}H_{18}BrN_3$

In a glovebox, 1,8-diaminonaphthalene (2 mmol, 316 mg), 2-amino-5-bromobenzyl alcohol (2 mmol, 404 mg), KO^{t}Bu (0.6 mmol, 67 mg, 30 mol%), Mn-**I** (0.02 mmol, 12 mg, 1 mol%) and 3 mL 2-MeTHF are added to a Schlenk tube. The reaction mixture is heated at 100 °C using an open system consisting of a reflux condenser and a bubble counter. After 2 hours reaction time, benzaldehyde (2 mmol, 203 µL) is diluted in 0.5 mL 2-MeTHF and added with a syringe to the reaction mixture via a septum. After stirring for 15 h, the mixture is cooled down to room temperature and 2 mL H_2O is added. The aqueous phase is extracted with dichloromethane (3 x 10 mL), the organic layers were dried with Na_2SO_4 and the solvent was removed in vacuo. The crude product was purified via column chromatography over Alox N (pentane/ethyl acetate: 5:1) and obtained as an orange solid (745 mg, 1.74 mmol, 87 %).

^{1}H NMR (DMSO-d$_6$, 500 MHz, 293 K): δ = 7.48 (d, J = 7.5 Hz, 2H), 7.44 (t, J = 7.5 Hz, 2H), 7.36 (d, J = 4.6 Hz, 2H), 7.27 (t, J = 7.9 Hz, 1H), 7.20 (dd, J = 12.7, 5.2 Hz, 3H), 7.15 (d, J = 8.1 Hz, 1H), 7.10 (d, J = 7.7 Hz, 1H), 7.04 – 6.97 (m, 2H), 6.64 (d, J = 3.9 Hz, 1H), 6.62 – 6.56 (m, 2H), 5.05 (d, J = 3.6 Hz, 1H) ppm.

^{13}C NMR (DMSO-d$_6$, 125 MHz, 293 K): δ = 142.55, 141.97, 140.69, 139.42, 134.26, 130.61, 128.71, 127.89, 127.79, 127.02, 126.84, 126.66, 123.82, 118.24, 115.68, 115.35, 113.65, 106.24, 105.84, 105.52, 65.41, 59.62 ppm.

Elemental analysis calculated: C 67.30, H 4.24, N 9.81

Elemental analysis found: C 67.17, H 4.34, N 9.84

Synthesis of **B1k**

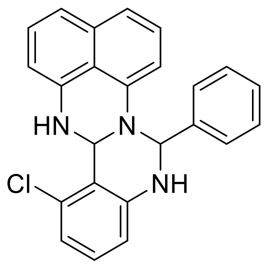

Chemical Formula: $C_{24}H_{18}ClN_3$

In a glovebox, 1,8-diaminonaphthalene (2 mmol, 316 mg), 2-amino-6-chlorobenzyl alcohol (2 mmol, 315 mg), KO^{t}Bu (0.6 mmol, 67 mg, 30 mol%), Mn-**I** (0.02 mmol, 12 mg, 1 mol%) and 3 mL 2-MeTHF are added to a Schlenk tube. The reaction mixture is heated at 100 °C using an open system consisting of a reflux condenser and a bubble counter. After 2 hours reaction time, benzaldehyde (2 mmol, 203 µL) is diluted in 0.5 mL 2-MeTHF and added with a syringe to the reaction mixture via a septum. After stirring for 15 h, the mixture is cooled down to room temperature and 2 mL H_2O is added. The aqueous phase is extracted with dichloromethane (3 x 10 mL), the organic layers were dried with Na_2SO_4 and the solvent was removed in vacuo. The crude product was purified via column chromatography over Alox N (pentane/ethyl acetate: 5:1) and obtained as a yellow solid (683 mg, 1.78 mmol, 89 %).

^{1}H NMR (DMSO-d$_6$, 500 MHz, 293 K): δ = 7.35 – 7.26 (m, 4H), 7.24 (dd, J = 9.3, 6.1 Hz, 4H), 7.11 (t, J = 8.5 Hz, 2H), 6.93 (t, J = 7.8 Hz, 1H), 6.78 – 6.70 (m, 3H), 6.68 (d, J = 7.8 Hz, 1H), 6.03 (d, J = 7.4 Hz, 1H), 5.60 (s, 1H), 5.48 (s, 1H) ppm.

^{13}C NMR (DMSO-d$_6$, 125 MHz, 293 K): δ = 145.31, 141.98, 140.80, 140.35, 134.26, 132.13, 129.91, 128.96, 128.63, 128.09, 126.75, 125.36, 121.89, 117.38, 116.75, 116.40, 115.99, 115.66, 112.81, 105.63, 68.39, 64.66 ppm.

Elemental analysis calculated: C 75.09, H 4.73, N 10.95

Elemental analysis found: C 75.28, H 4.78, N 11.37

Synthesis of **B1l**

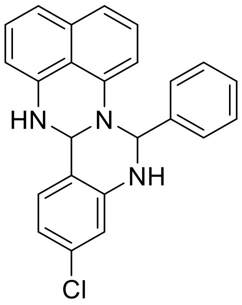

Chemical Formula: $C_{24}H_{18}ClN_3$

In a glovebox, 1,8-diaminonaphthalene (2 mmol, 316 mg), 2-amino-4-chlorobenzyl alcohol (2 mmol, 315 mg), KO^{t}Bu (0.6 mmol, 67 mg, 30 mol%), Mn-**I** (0.02 mmol, 12 mg, 1 mol%) and 3 mL 2-MeTHF are added to a Schlenk tube. The reaction mixture is heated at 100 °C using an open system consisting of a reflux condenser and a bubble counter. After 2 hours reaction time, benzaldehyde (2 mmol, 203 µL) is diluted in 0.5 mL 2-MeTHF and added with a syringe to the reaction mixture via a septum. After stirring for 15 h, the mixture is cooled down to room temperature and 2 mL H_2O is added. The aqueous phase is extracted with dichloromethane (3 x 10 mL), the organic layers were dried with Na_2SO_4 and the solvent was removed in vacuo. The crude product was purified via column chromatography over Alox N (pentane/ethyl acetate: 5:1) and obtained as a yellow solid (683 mg, 1.78 mmol, 89 %).

^{1}H NMR (DMSO-d$_6$, 500 MHz, 293 K): δ = 7.49 (d, J = 7.5 Hz, 2H), 7.44 (t, J = 7.6 Hz, 2H), 7.38 – 7.31 (m, 2H), 7.29 – 7.22 (m, 2H), 7.20 – 7.08 (m, 3H), 7.03 (d, J = 8.1 Hz, 1H), 6.98 (d, J = 8.0 Hz, 1H), 6.65 (dd, J = 8.1, 3.0 Hz, 2H), 6.57 (d, J = 7.3 Hz, 1H), 6.39 (d, J = 8.1 Hz, 1H), 5.04 (d, J = 3.9 Hz, 1H) ppm.

^{13}C NMR (DMSO-d$_6$, 125 MHz, 293 K): δ = 144.59, 141.92, 140.78, 139.54, 134.26, 132.35, 128.71, 127.90, 127.14, 126.93, 126.86, 126.62, 120.40, 118.22, 115.60, 114.88, 113.74, 112.20, 105.83, 105.53, 65.47, 59.67 ppm.

Elemental analysis calculated: C 75.09, H 4.73, N 10.95
Elemental analysis found: C 75.19, H 4.95, N 10.99

Synthesis of **B1m**

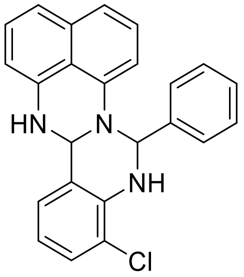

Chemical Formula: $C_{24}H_{18}ClN_3$

In a glovebox, 1,8-diaminonaphthalene (2 mmol, 316 mg), 2-amino-3-chlorobenzyl alcohol (2 mmol, 315 mg), KO^{t}Bu (0.6 mmol, 67 mg, 30 mol%), Mn-**I** (0.02 mmol, 12 mg, 1 mol%) and 3 mL 2-MeTHF are added to a Schlenk tube. The reaction mixture is heated at 100 °C using an open system consisting of a reflux condenser and a bubble counter. After 2 hours reaction time, benzaldehyde (2 mmol, 203 µL) is diluted in 0.5 mL 2-MeTHF and added with a syringe to the reaction mixture via a septum. After stirring for 15 h, the mixture is cooled down to room temperature and 2 mL H_2O is added. The aqueous phase is extracted with dichloromethane (3 x 10 mL), the organic layers were dried with Na_2SO_4 and the solvent was removed in vacuo. The crude product is purified by recrystallization in ethyl acetate/pentane and obtained as orange crystals (575 mg, 1.50 mmol, 75 %).

^{1}H NMR (DMSO-d$_6$, 500 MHz, 293 K): δ = 7.51 – 7.40 (m, 5H), 7.37 (d, J = 6.7 Hz, 1H), 7.28 (t, J = 7.8 Hz, 1H), 7.23 – 7.10 (m, 2H), 7.07 (dd, J = 14.1, 7.4 Hz, 3H), 6.98 (d, J = 8.0 Hz, 1H), 6.84 (d, J = 4.4 Hz, 1H), 6.68 (d, J = 4.0 Hz, 1H), 6.59 (d, J = 7.2 Hz, 1H), 6.42 (t, J = 7.7 Hz, 1H), 5.10 (s, 1H) ppm.

^{13}C NMR (DMSO-d$_6$, 125 MHz, 293 K): δ = 141.64, 140.60, 139.41, 139.16, 134.25, 128.77, 128.05, 127.94, 127.02, 126.75, 126.69, 124.29, 123.84, 118.31, 116.71, 115.77, 115.56, 113.65, 105.56, 105.49, 65.30, 59.75 ppm.

Elemental analysis calculated: C 75.09, H 4.73, N 10.95
Elemental analysis found: C 74.86, H 4.79, N 11.20

Synthesis of **B1n**

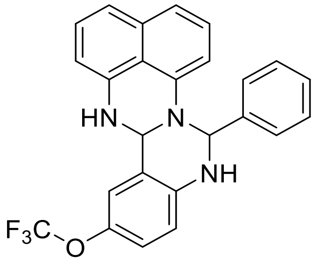

Chemical Formula: $C_{25}H_{18}F_3N_3O$

In a glovebox, 1,8-diaminonaphthalene (2 mmol, 316 mg), 2-amino-5-(trifluormethoxy)benzyl alcohol (2 mmol, 414 mg), KO^{t}Bu (0.6 mmol, 67 mg, 30 mol%), Mn-**I** (0.02 mmol, 12 mg, 1 mol%) and 3 mL 2-MeTHF are added to a Schlenk tube. The reaction mixture is heated at 100 °C using an open system consisting of a reflux condenser and a bubble counter. After 2 hours reaction time, benzaldehyde (2 mmol, 203 µL) is diluted in 0.5 mL 2-MeTHF and added with a syringe to the reaction mixture via a septum. After stirring for 15 h, the mixture is cooled down to room temperature and 7 mL H_2O is added. After adding 10 mL pentane the precipitation is filtered and washed with pentane. The product is obtained as a yellow solid after drying in vacuo overnight (606 mg, 1.40 mmol, 70 %).

^{1}H NMR (DMSO-d$_6$, 500 MHz, 293 K): δ = 7.50 (d, J = 7.5 Hz, 1H), 7.45 (t, J = 7.6 Hz, 1H), 7.41 – 7.32 (m, 1H), 7.27 (dd, J = 9.6, 6.2 Hz, 1H), 7.19 (t, J = 7.8 Hz, 1H), 7.13 (dd, J = 11.9, 8.0 Hz, 1H), 7.04 (s, 1H), 6.99 (d, J = 8.1 Hz, 1H), 6.87 (dd, J = 8.7, 2.3 Hz, 1H), 6.70 – 6.64 (m, 1H), 6.60 (d, J = 7.3 Hz, 1H), 5.06 (d, J = 3.8 Hz, 1H) ppm.

^{13}C NMR (DMSO-d$_6$, 125 MHz, 293 K): δ = 142.48, 141.99, 140.67, 139.36, 138.09, 134.24, 128.75, 127.89, 126.97, 126.84, 126.65, 122.45, 121.35, 118.59, 118.25, 115.73, 113.92, 113.70, 105.85, 105.57, 65.46, 59.67 ppm.

^{19}F NMR (DMSO-d$_6$, 376 MHz, 293 K): δ = -57.31 (s) ppm.

Elemental analysis calculated: C 69.28, H 4.19, N 9.69

Elemental analysis found: C 69.62, H 4.23, N 10.02

Synthesis of **B1o**

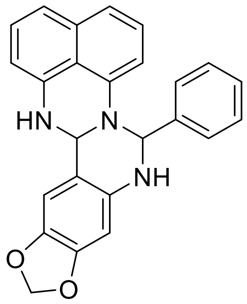

Chemical Formula: $C_{25}H_{19}N_3O_2$

In a glovebox, 1,8-diaminonaphthalene (2 mmol, 316 mg), (6-aminobenzo[d][1,3]dioxol-5-yl)methanol (2 mmol, 334 mg), KO^{t}Bu (0.6 mmol, 67 mg, 30 mol%), Mn-**I** (0.02 mmol, 12 mg, 1 mol%) and 3 mL 2-MeTHF are added to a Schlenk tube. The reaction mixture is heated at 100 °C using an open system consisting of a reflux condenser and a bubble counter. After 2 hours reaction time, benzaldehyde (2 mmol, 203 µL) is diluted in 0.5 mL 2-MeTHF and added with a syringe to the reaction mixture via a septum. After stirring for 15 h, the mixture is cooled down to room temperature and 2 mL H_2O is added. The aqueous phase is extracted with dichloromethane (3 x 10 mL), the organic layers were dried with Na_2SO_4 and the solvent was removed in vacuo. The crude product was purified via column chromatography over Alox N (pentane/ethyl acetate: 5:2) and obtained as a yellow solid (621 mg, 1.58 mmol, 79 %).

^{1}H NMR (DMSO-d$_6$, 500 MHz, 293 K): δ = 7.48 (d, J = 7.6 Hz, 2H), 7.42 (t, J = 7.6 Hz, 2H), 7.33 (t, J = 7.2 Hz, 1H), 7.28 – 7.20 (m, 2H), 7.17 (t, J = 7.8 Hz, 1H), 7.12 (d, J = 8.1 Hz, 1H), 7.05 (d, J = 7.8 Hz, 1H), 6.98 (d, J = 8.1 Hz, 1H), 6.67 (s, 2H), 6.56 (d, J = 7.3 Hz, 1H), 6.52 (d, J = 3.7 Hz, 1H), 6.26 (s, 1H), 5.74 (s, 1H), 5.68 (s, 1H), 4.99 (d, J = 3.4 Hz, 1H) ppm.

^{13}C NMR (DMSO-d$_6$, 125 MHz, 293 K): δ = 146.91, 142.18, 141.13, 139.86, 138.15, 138.13, 134.26, 128.58, 127.68, 126.95, 126.87, 126.66, 117.80, 115.44, 113.84, 113.74, 105.73, 105.61, 105.28, 99.89, 95.69, 65.61, 60.03 ppm.

Elemental analysis calculated: C 76.32, H 4.87, N 10.68
Elemental analysis found: C 75.96 H 4.87 N 10.39

Synthesis of **B1p**

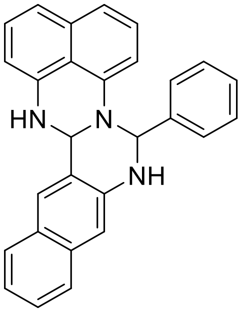

Chemical Formula: $C_{28}H_{21}N_3$

In a glovebox, 1,8-diaminonaphthalene (2 mmol, 316 mg), (3-aminonaphthalen-2-yl)methanol (2 mmol, 347 mg), KO^{t}Bu (0.6 mmol, 67 mg, 30 mol%), Mn-**I** (0.02 mmol, 12 mg, 1 mol%) and 3 mL 2-MeTHF are added to a Schlenk tube. The reaction mixture is heated at 100 °C using an open system consisting of a reflux condenser and a bubble counter. After 2 hours reaction time, benzaldehyde (2 mmol, 203 µL) is diluted in 0.5 mL 2-MeTHF and added with a syringe to the reaction mixture via a septum. After stirring for 15 h, the mixture is cooled down to room temperature. The product precipitates during reaction. For purification, it was filtrated, washed with H_2O and cold diethyl ether and dried at 70 °C in vacuo. The product is obtained as a colourless solid (583 mg, 1.46 mmol, 73 %).

^{1}H NMR (DMSO-d$_6$, 500 MHz, 293 K): δ = 7.60 – 7.54 (m, 3H), 7.53 (d, J = 3.9 Hz, 1H), 7.44 (dd, J = 14.1, 7.2 Hz, 4H), 7.35 (dd, J = 9.7, 5.7 Hz, 2H), 7.27 – 7.15 (m, 3H), 7.11 (dd, J = 13.9, 8.0 Hz, 2H), 7.01 (t, J = 7.5 Hz, 1H), 6.95 (d, J = 8.1 Hz, 1H), 6.91 (s, 1H), 6.70 (d, J = 4.0 Hz, 1H), 6.67 (d, J = 7.4 Hz, 1H), 5.27 (d, J = 3.6 Hz, 1H) ppm.

^{13}C NMR (DMSO-d$_6$, 125 MHz, 293 K): δ = 142.34, 141.90, 140.83, 139.80, 134.26, 134.14, 128.64, 127.84, 127.34, 126.99, 126.61, 125.89, 125.64, 125.44, 124.76, 124.51, 121.30, 118.08, 115.51, 113.79, 105.78, 105.57, 65.58, 60.19 ppm.

Elemental analysis calculated: C 84.18, H 5.30, N 10.52
Elemental analysis found: C 83.78, H 5.02, N 10.34

Synthesis of **B1q**

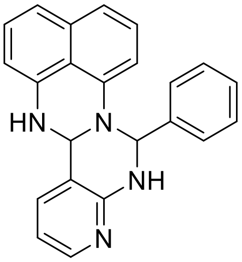

Chemical Formula: $C_{23}H_{18}N_4$

In a glovebox, 1,8-diaminonaphthalene (2 mmol, 316 mg), 2-amino-3-pyridinecarboxaldehyde (2 mmol, 225 mg), KO^{t}Bu (0.6 mmol, 67 mg, 30 mol%), Mn-**I** (0.02 mmol, 12 mg, 1 mol%) and 3 mL 2-MeTHF are added to a Schlenk tube. The reaction mixture is heated at 100 °C using an open system consisting of a reflux condenser and a bubble counter. After 2 hours reaction time, benzaldehyde (2 mmol, 203 µL) is diluted in 0.5 mL 2-MeTHF and added with a syringe to the reaction mixture via a septum. After stirring for 15 h, the mixture is cooled down to room temperature. The product precipitates during reaction. For purification, it was filtrated, washed with H_2O and cold diethyl ether and dried at 70 °C in vacuo. The product is obtained as a light pink solid (603 mg, 1.72 mmol, 86 %).

^{1}H NMR (DMSO-d_6, 500 MHz, 293 K): δ = 7.76 (d, J = 4.2 Hz, 1H), 7.69 (d, J = 4.4 Hz, 1H), 7.47 (dt, J = 20.0, 7.6 Hz, 5H), 7.36 (dd, J = 16.0, 7.5 Hz, 2H), 7.27 (d, J = 7.9 Hz, 1H), 7.22 – 7.12 (m, 2H), 7.10 (d, J = 7.7 Hz, 1H), 6.99 (d, J = 8.1 Hz, 1H), 6.66 (d, J = 4.3 Hz, 1H), 6.61 (d, J = 7.3 Hz, 1H), 6.43 – 6.37 (m, 1H), 5.06 (d, J = 3.7 Hz, 1H) ppm.

^{13}C NMR (DMSO-d_6, 125 MHz, 293 K): δ = 154.30, 147.20, 141.73, 140.46, 139.35, 134.25, 132.94, 128.74, 127.95, 126.99, 126.85, 126.66, 118.41, 116.96, 115.68, 113.58, 112.17, 105.74, 64.95, 59.61 ppm.

Elemental analysis calculated: C 78.83, H 5.18, N 15.99

Elemental analysis found: C 78.52, H 5.41, N 16.01

Synthesis of **B2a**

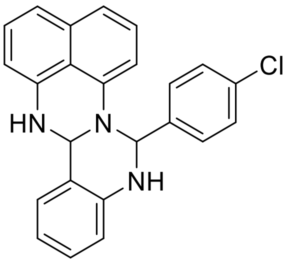

Chemical Formula: $C_{24}H_{18}ClN_3$

In a glovebox, 1,8-diaminonaphthalene (2 mmol, 316 mg), 2-aminobenzyl alcohol (2 mmol, 247 mg), KO^{t}Bu (0.6 mmol, 67 mg, 30 mol%), Mn-**I** (0.02 mmol, 12 mg, 1 mol%) and 3 mL 2-MeTHF are added to a Schlenk tube. The reaction mixture is heated at 100 °C using an open system consisting of a reflux condenser and a bubble counter. After 2 hours reaction time, 4-chlorobenzaldehyde (2 mmol, 281 mg) is diluted in 0.7 mL 2-MeTHF and added with a syringe to the reaction mixture via a septum. After stirring for 15 h, the mixture is cooled down to room temperature and 2 mL H_2O is added. The aqueous phase is extracted with dichloromethane (3 x 10 mL), the organic layers were dried with Na_2SO_4 and the solvent was removed in vacuo. The crude product was purified by recrystallization in ethyl acetate and obtained as yellow crystals (537 mg, 1.40 mmol, 70 %).

^{1}H NMR (CD_3CN, 500 MHz, 293 K): δ = 7.54 (d, J = 8.3 Hz, 2H), 7.54 (d, J = 8.3 Hz, 2H), 7.42 (d, J = 8.5 Hz, 2H), 7.42 (d, J = 8.5 Hz, 2H), 7.27 (t, J = 7.9 Hz, 1H), 7.27 (t, J = 7.9 Hz, 1H), 7.22 (t, J = 7.8 Hz, 1H), 7.22 (t, J = 7.8 Hz, 1H), 7.17 (d, J = 8.2 Hz, 1H), 7.17 (d, J = 8.2 Hz, 1H), 7.07 (t, J = 6.5 Hz, 2H), 6.99 (d, J = 7.7 Hz, 1H), 6.93 (t, J = 7.7 Hz, 1H), 6.67 (d, J = 7.4 Hz, 1H), 6.67 (d, J = 7.4 Hz, 1H), 6.62 (d, J = 8.0 Hz, 1H), 6.62 (d, J = 8.0 Hz, 1H), 6.54 (d, J = 4.8 Hz, 1H), 6.48 (t, J = 7.5 Hz, 1H), 6.48 (t, J = 7.5 Hz, 1H), 5.91 (d, J = 3.2 Hz, 1H), 5.65 (d, J = 4.4 Hz, 1H), 5.19 (d, J = 3.9 Hz, 1H) ppm.

^{13}C NMR (CD_3CN, 125 MHz, 293 K): δ = 143.87, 142.28, 142.08, 140.71, 135.85, 134.29, 130.04, 129.79, 129.54, 128.11, 127.78, 126.59, 123.21, 119.85, 117.89, 117.75, 115.58, 115.11, 107.48, 66.85, 61.77 ppm

Elemental analysis calculated: C 75.09, H 4.73, N 10.95

Elemental analysis found: C 74.82, H 5.04, N 10.80

Synthesis of **B2b**

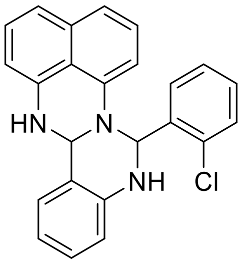

Chemical Formula: $C_{24}H_{18}ClN_3$

In a glovebox, 1,8-diaminonaphthalene (2 mmol, 316 mg), 2-aminobenzyl alcohol (2 mmol, 247 mg), KO^{t}Bu (0.6 mmol, 67 mg, 30 mol%), Mn-**I** (0.02 mmol, 12 mg, 1 mol%) and 3 mL 2-MeTHF are added to a Schlenk tube. The reaction mixture is heated at 100 °C using an open system consisting of a reflux condenser and a bubble counter. After 2 hours reaction time, 2-chlorobenzaldehyde (2 mmol, 225 µL) is diluted in 0.7 mL 2-MeTHF and added with a syringe to the reaction mixture via a septum. After stirring for 15 h, the mixture is cooled down to room temperature and 2 mL H_2O is added. The aqueous phase is extracted with dichloromethane (3 x 10 mL), the organic layers were dried with Na_2SO_4 and the solvent was removed in vacuo. The crude product was purified via column chromatography over Alox N (pentane/ethyl acetate: 5:1) and obtained as an orange solid (575 mg, 1.50 mmol, 75 %).

^{1}H NMR (DMSO-d$_6$, 500 MHz, 293 K): δ = 7.58 – 7.49 (m, 2H), 7.46 – 7.39 (m, 2H), 7.36 (d, J = 3.9 Hz, 1H), 7.27 (t, J = 7.9 Hz, 1H), 7.17 (t, J = 7.8 Hz, 1H), 7.11 (d, J = 8.0 Hz, 2H), 7.06 (d, J = 7.8 Hz, 1H), 6.96 (d, J = 8.1 Hz, 1H), 6.90 (t, J = 7.6 Hz, 1H), 6.81 (d, J = 4.6 Hz, 1H), 6.74 (d, J = 4.5 Hz, 1H), 6.59 (dd, J = 10.5, 7.8 Hz, 2H), 6.42 (t, J = 7.4 Hz, 1H), 5.14 (d, J = 3.8 Hz, 1H) ppm.

^{13}C NMR (DMSO-d$_6$, 125 MHz, 293 K): δ = 143.32, 140.36, 139.69, 138.73, 134.36, 131.99, 130.14, 129.82, 129.77, 128.15, 126.96, 126.71, 125.31, 121.47, 117.90, 115.69, 115.44, 113.72, 113.06, 105.49, 105.23, 63.94, 59.58 ppm.

Elemental analysis calculated: C 75.09, H 4.73, N 10.95

Elemental analysis found: C 75.58, H 4.51, N 11.02

Synthesis of **B2c**

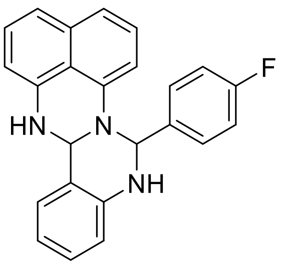

Chemical Formula: $C_{24}H_{18}FN_3$

In a glovebox, 1,8-diaminonaphthalene (2 mmol, 316 mg), 2-aminobenzyl alcohol (2 mmol, 247 mg), KO^{t}Bu (0.6 mmol, 67 mg, 30 mol%), Mn-**I** (0.02 mmol, 12 mg, 1 mol%) and 3 mL 2-MeTHF are added to a Schlenk tube. The reaction mixture is heated at 100 °C using an open system consisting of a reflux condenser and a bubble counter. After 2 hours reaction time, 4-fluorobenzaldehyde (2 mmol, 215 µL) is diluted in 0.5 mL 2-MeTHF and added with a syringe to the reaction mixture via a septum. After stirring for 15 h, the mixture is cooled down to room temperature and 2 mL H_2O is added. The aqueous phase is extracted with dichloromethane (3 x 10 mL), the organic layers were dried with Na_2SO_4 and the solvent was removed in vacuo. The crude product was purified via column chromatography over Alox N (pentane/ethyl acetate: 5:1) and obtained as a yellow solid (683 mg, 1.85 mmol, 92 %).

^{1}H NMR (DMSO-d$_6$, 500 MHz, 293 K): δ = 7.96 (dd, J = 8.5, 5.6 Hz, 2H), 7.77 (d, J = 3.7 Hz, 1H), 7.72 – 7.65 (m, 3H), 7.61 (t, J = 7.8 Hz, 1H), 7.58 – 7.46 (m, 3H), 7.45 – 7.38 (m, 2H), 7.32 (t, J = 7.6 Hz, 1H), 7.09 – 7.00 (m, 3H), 6.83 (t, J = 7.4 Hz, 1H), 5.52 (d, J = 3.1 Hz, 1H) ppm.

^{13}C NMR (DMSO-d$_6$, 125 MHz, 293 K): δ = 143.47, 141.45, 140.39, 138.97, 134.73, 129.53, 129.46, 128.45, 127.37, 127.05, 125.86, 122.05, 118.46, 116.07, 115.92, 115.84, 115.75, 114.25, 113.88, 106.20, 105.84, 65.47, 60.41 ppm.

^{19}F NMR (DMSO-d$_6$, 376 MHz, 293 K): δ = -115.19 (m) ppm.

Elemental analysis calculated: C 78.45, H 4.94, N 11.44

Elemental analysis found: C 77.87, H 5.15, N 11.41

Synthesis of **B2d**

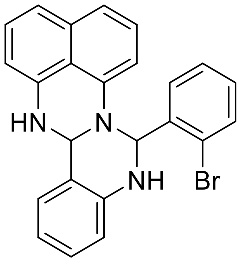

Chemical Formula: $C_{24}H_{18}BrN_3$

In a glovebox, 1,8-diaminonaphthalene (2 mmol, 316 mg), 2-aminobenzyl alcohol (2 mmol, 247 mg), KO^{t}Bu (0.6 mmol, 67 mg, 30 mol%), Mn-**I** (0.02 mmol, 12 mg, 1 mol%) and 3 mL 2-MeTHF are added to a Schlenk tube. The reaction mixture is heated at 100 °C using an open system consisting of a reflux condenser and a bubble counter. After 2 hours reaction time, 2-bromobenzaldehyde (2 mmol, 234 µL) is diluted in 0.5 mL 2-MeTHF and added with a syringe to the reaction mixture via a septum. After stirring for 15 h, the mixture is cooled down to room temperature and 2 mL H_2O is added. The aqueous phase is extracted with dichloromethane (3 x 10 mL), the organic layers were dried with Na_2SO_4 and the solvent was removed in vacuo. The crude product was purified via recrystallization in ethyl acetate at -4 °C and obtained as orange crystals (719 mg, 1.67 mmol, 84 %).

^{1}H NMR (DMSO-d$_6$, 500 MHz, 293 K): δ = 7.70 (d, J = 7.8 Hz, 1H), 7.51 (d, J = 7.6 Hz, 1H), 7.45 (t, J = 7.5 Hz, 1H), 7.38 – 7.30 (m, 2H), 7.27 (t, J = 8.0 Hz, 1H), 7.17 (t, J = 7.8 Hz, 1H), 7.11 (d, J = 8.0 Hz, 2H), 7.07 (d, J = 7.8 Hz, 1H), 6.96 (d, J = 8.1 Hz, 1H), 6.90 (t, J = 7.4 Hz, 1H), 6.83 (d, J = 4.7 Hz, 1H), 6.63 (d, J = 4.5 Hz, 1H), 6.59 (t, J = 8.1 Hz, 2H), 6.42 (s, 1H), 5.13 (d, J = 3.9 Hz, 1H) ppm.

^{13}C NMR (DMSO-d$_6$, 125 MHz, 293 K): δ = 143.31, 140.30, 140.10, 139.67, 134.36, 133.45, 130.10, 129.98, 128.17, 127.51, 126.94, 126.71, 125.33, 122.17, 121.53, 117.92, 115.70, 115.44, 113.76, 113.02, 105.49, 105.37, 66.08, 59.50 ppm.

Elemental analysis calculated: C 67.30, H 4.24, N 9.81

Elemental analysis found: C 67.68, H 3.97, N 9.83

Synthesis of **B2e**

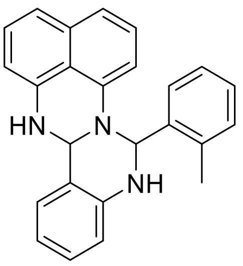

Chemical Formula: $C_{25}H_{21}N_3$

In a glovebox, 1,8-diaminonaphthalene (2 mmol, 316 mg), 2-aminobenzyl alcohol (2 mmol, 247 mg), KO^{t}Bu (0.6 mmol, 67 mg, 30 mol%), Mn-**I** (0.02 mmol, 12 mg, 1 mol%) and 3 mL 2-MeTHF are added to a Schlenk tube. The reaction mixture is heated at 100 °C using an open system consisting of a reflux condenser and a bubble counter. After 2 hours reaction time, 2-methylbenzaldehyde (2 mmol, 233 µL) is diluted in 0.5 mL 2-MeTHF and added with a syringe to the reaction mixture via a septum. After stirring for 15 h, the mixture is cooled down to room temperature and 2 mL H_2O is added. The aqueous phase is extracted with dichloromethane (3 x 10 mL), the organic layers were dried with Na_2SO_4 and the solvent was removed in vacuo. The crude product was purified via column chromatography over Alox N (pentane/ethyl acetate: 5:1) and obtained as a yellow solid (660 mg, 1.82 mmol, 91 %).

^{1}H NMR (DMSO-d$_6$, 500 MHz, 293 K): δ = 7.39 (d, J = 6.3 Hz, 1H), 7.34 (s, 1H), 7.25 (s, 3H), 7.19 – 7.03 (m, 4H), 6.95 (d, J = 7.6 Hz, 1H), 6.91 – 6.84 (m, 1H), 6.78 (s, 1H), 6.65 (s, 1H), 6.60 (d, J = 7.4 Hz, 1H), 6.56 (d, J = 6.6 Hz, 1H), 6.41 – 6.34 (m, 1H), 5.08 (s, 1H), 2.35 (s, 3H) ppm.

^{13}C NMR (DMSO-d$_6$, 125 MHz, 293 K): δ = 143.71, 140.68, 139.86, 139.51, 135.80, 134.38, 130.92, 128.02, 127.83, 127.60, 126.93, 126.75, 125.50, 125.24, 121.39, 117.72, 115.33, 115.27, 113.79, 112.92, 105.34, 105.14, 63.91, 59.70, 18.30 ppm.

Elemental analysis calculated: C 82.61, H 5.82, N 11.56

Elemental analysis found: C 82.32, H 5.87, N 11.70

Synthesis of **B2f**

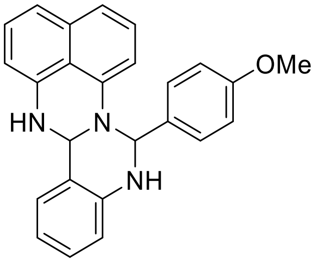

Chemical Formula: $C_{25}H_{21}N_3O$

In a glovebox, 1,8-diaminonaphthalene (2 mmol, 316 mg), 2-aminobenzyl alcohol (2 mmol, 247 mg), KO^{t}Bu (0.6 mmol, 67 mg, 30 mol%), Mn-**I** (0.02 mmol, 12 mg, 1 mol%) and 3 mL 2-MeTHF are added to a Schlenk tube. The reaction mixture is heated at 100 °C using an open system consisting of a reflux condenser and a bubble counter. After 2 hours reaction time, *p*-anisaldehyde (2 mmol, 244 µL) is diluted in 0.5 mL 2-MeTHF and added with a syringe to the reaction mixture via a septum. After stirring for 15 h, the mixture is cooled down to room temperature and 2 mL H_2O is added. The aqueous phase is extracted with dichloromethane (3 x 10 mL), the organic layers were dried with Na_2SO_4 and the solvent was removed in vacuo. The crude product was purified via recrystallization in ethyl acetate at -4 °C and obtained as yellow crystals (592 mg, 1.56 mmol, 78 %).

^{1}H NMR (DMSO-d$_6$, 500 MHz, 293 K): δ = 7.40 (d, J = 8.5 Hz, 1H), 7.32 (d, J = 3.4 Hz, 1H), 7.23 (t, J = 7.9 Hz, 1H), 7.16 (t, J = 7.8 Hz, 1H), 7.08 (dd, J = 14.5, 7.9 Hz, 1H), 7.03 (d, J = 7.7 Hz, 1H), 6.97 (dd, J = 13.5, 8.4 Hz, 1H), 6.91 (d, J = 4.0 Hz, 1H), 6.86 (t, J = 7.5 Hz, 1H), 6.58 (t, J = 8.2 Hz, 1H), 6.52 (d, J = 3.7 Hz, 1H), 6.38 (t, J = 7.3 Hz, 1H), 5.10 (d, J = 3.2 Hz, 1H), 3.76 (s, 1H) ppm.

^{13}C NMR (DMSO-d$_6$, 125 MHz, 293 K): δ = 158.83, 143.23, 141.19, 140.05, 134.29, 134.14, 128.13, 127.92, 126.88, 126.62, 125.36, 121.63, 117.80, l115.39, 115.30, 113.94, 113.82, 113.31, 105.63, 105.26, 65.08, 59.89, 55.12 ppm.

Elemental analysis calculated: C 79.13, H 5.58, N 11.07

Elemental analysis found: C 78.70, H 5.58, N 10.89

Synthesis of **B2g**

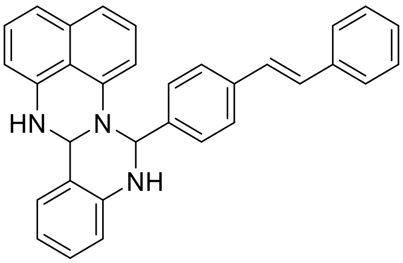

Chemical Formula: $C_{32}H_{25}N_3$

In a glovebox, 1,8-diaminonaphthalene (2 mmol, 316 mg), 2-aminobenzyl alcohol (2 mmol, 247 mg), KO^{t}Bu (0.6 mmol, 67 mg, 30 mol%), Mn-**I** (0.02 mmol, 12 mg, 1 mol%) and 3 mL 2-MeTHF are added to a Schlenk tube. The reaction mixture is heated at 100 °C using an open system consisting of a reflux condenser and a bubble counter. After 2 hours reaction time, *trans*-4-stilbenecarboxyaldehyde (2 mmol, 416 mg) is diluted in 2 mL 2-MeTHF and added with a syringe to the reaction mixture via a septum. After stirring for 15 h, the mixture is cooled down to room temperature. The product precipitates during reaction. For purification it was filtrated, washed with H_2O and cold diethyl ether and dried at 70 °C in vacuo. The product is obtained as a white solid (614 mg, 1.36 mmol, 68 %).

^{1}H NMR (DMSO-d$_6$, 500 MHz, 293 K): δ = 7.67 (d, J = 8.0 Hz, 1H), 7.61 (d, J = 7.5 Hz, 1H), 7.51 (d, J = 7.9 Hz, 1H), 7.41 – 7.34 (m, 2H), 7.31 – 7.23 (m, 2H), 7.17 (t, J = 7.7 Hz, 1H), 7.13 – 7.04 (m, 2H), 6.97 (t, J = 6.7 Hz, 1H), 6.88 (t, J = 7.5 Hz, 1H), 6.65 – 6.57 (m, 2H), 6.39 (t, J = 7.3 Hz, 1H), 5.14 (d, J = 3.0 Hz, 1H) ppm.

^{13}C NMR (DMSO-d$_6$, 125 MHz, 293 K): δ = 143.18, 141.79, 141.11, 140.00, 137.01, 136.58, 134.30, 128.75, 128.60, 128.02, 127.97, 127.71, 127.36, 126.91, 126.73, 126.62, 126.50, 125.40, 121.66, 117.90, 115.51, 115.35, 113.80, 113.38, 105.64, 105.35, 65.40, 60.09 ppm.

Elemental analysis calculated: C 85.11, H 5.58, N 9.31

Elemental analysis found: C 84.77, H 5.37, N 9.03

Synthesis of **B2h**

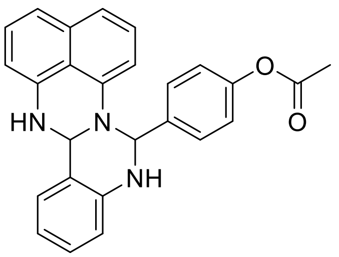

Chemical Formula: $C_{26}H_{21}N_3O_2$

In a glovebox, 1,8-diaminonaphthalene (2 mmol, 316 mg), 2-aminobenzyl alcohol (2 mmol, 247 mg), KO^{t}Bu (0.6 mmol, 67 mg, 30 mol%), Mn-**I** (0.02 mmol, 12 mg, 1 mol%) and 3 mL 2-MeTHF are added to a Schlenk tube. The reaction mixture is heated at 100 °C using an open system consisting of a reflux condenser and a bubble counter. After 2 hours reaction time, 4-(acetyloxy)-benzaldehyde (2 mmol, 278 µL) is added with a syringe to the reaction mixture via a septum. After stirring for 15 h, the mixture is cooled down to room temperature and 2 mL H_2O is added. The aqueous phase is extracted with ethyl acetate (3 x 10 mL), the organic layers were dried with Na_2SO_4 and the solvent was removed in vacuo. The crude product was purified via column chromatography over Alox N (pentane/ethyl acetate: 5:1) and obtained as an orange-brown solid (562 mg, 1.38 mmol, 69 %).

^{1}H NMR (DMSO-d$_6$, 500 MHz, 293 K): δ = 7.52 (d, J = 8.4 Hz, 1H), 7.35 (d, J = 3.5 Hz, 1H), 7.25 (t, J = 7.9 Hz, 1H), 7.21 – 7.14 (m, 1H), 7.11 (d, J = 8.1 Hz, 1H), 7.06 (t, J = 9.0 Hz, 1H), 6.97 (t, J = 6.3 Hz, 1H), 6.87 (t, J = 7.4 Hz, 1H), 6.59 (dd, J = 14.4, 8.1 Hz, 1H), 6.39 (t, J = 7.3 Hz, 1H), 5.10 (d, J = 3.2 Hz, 1H), 2.27 (s, 1H) ppm.

^{13}C NMR (DMSO-d$_6$, 125 MHz, 293 K): δ = 169.27, 149.96, 143.05, 141.03, 139.93, 139.82, 134.28, 128.07, 127.98, 126.92, 126.60, 125.41, 121.99, 121.59, 117.96, 115.58, 115.36, 113.78, 113.41, 105.66, 105.34, 65.15, 59.98, 20.87 ppm.

Elemental analysis calculated: C 76.64, H 5.19, N 10.31
Elemental analysis found: C 76.76, H 5.36, N 9.95

Synthesis of **B2i**

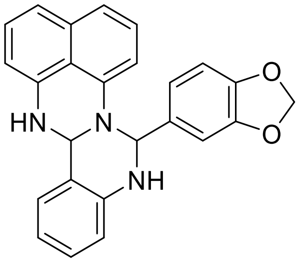

Chemical Formula: $C_{25}H_{19}N_3O_2$

In a glovebox, 1,8-diaminonaphthalene (2 mmol, 316 mg), 2-aminobenzyl alcohol (2 mmol, 247 mg), KO^{t}Bu (0.6 mmol, 67 mg, 30 mol%), Mn-**I** (0.02 mmol, 12 mg, 1 mol%) and 3 mL 2-MeTHF are added to a Schlenk tube. The reaction mixture is heated at 100 °C using an open system consisting of a reflux condenser and a bubble counter. After 2 hours reaction time, piperonal (2 mmol, 301 mg) is diluted in 0.7 mL 2-MeTHF and added with a syringe to the reaction mixture via a septum. After stirring for 15 h, the mixture is cooled down to room temperature and 2 mL H_2O is added. The aqueous phase is extracted with dichloromethane (3 x 10 mL), the organic layers were dried with Na_2SO_4 and the solvent was removed in vacuo. The crude product was purified via recrystallization in ethyl acetate at -4 °C and obtained as yellow crystals (621 mg, 1.58 mmol, 79 %).

^{1}H NMR (DMSO-d$_6$, 500 MHz, 293 K): δ = 7.32 (d, J = 3.8 Hz, 1H), 7.23 (t, J = 7.9 Hz, 1H), 7.16 (t, J = 7.8 Hz, 1H), 7.08 (dd, J = 13.7, 7.9 Hz, 2H), 7.01 (d, J = 7.8 Hz, 1H), 6.98 – 6.92 (m, 4H), 6.86 (t, J = 7.5 Hz, 1H), 6.58 (d, J = 7.7 Hz, 2H), 6.47 (d, J = 4.3 Hz, 1H), 6.38 (t, J = 7.4 Hz, 1H), 6.03 (d, J = 5.2 Hz, 2H), 5.13 (d, J = 3.7 Hz, 1H) ppm.

^{13}C NMR (DMSO-d$_6$, 125 MHz, 293 K): δ = 147.62, 146.82, 143.05, 141.07, 140.00, 136.25, 134.26, 127.94, 126.88, 126.59, 125.37, 121.63, 120.35, 117.86, 115.50, 115.35, 113.81, 113.30, 108.11, 106.95, 105.65, 105.34, 101.15, 65.28, 59.94 ppm.

Elemental analysis calculated: C 76.32, H 4.87, N 10.68

Elemental analysis found: C 76.01, H 4.82, N 10.60

Synthesis of **B2j**

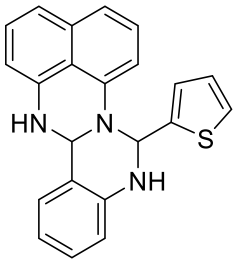

Chemical Formula: $C_{22}H_{17}N_3S$

In a glovebox, 1,8-diaminonaphthalene (2 mmol, 316 mg), 2-aminobenzyl alcohol (2 mmol, 247 mg), KO^{t}Bu (0.6 mmol, 67 mg, 30 mol%), Mn-**I** (0.02 mmol, 12 mg, 1 mol%) and 3 mL 2-MeTHF are added to a Schlenk tube. The reaction mixture is heated at 100 °C using an open system consisting of a reflux condenser and a bubble counter. After 2 hours reaction time, 2-thiophenecarboxaldehyde (2 mmol, 187 µL) is diluted in 0.5 mL 2-MeTHF and added with a syringe to the reaction mixture via a septum. After stirring for 15 h, the mixture is cooled down to room temperature and 2 mL H_2O is added. The aqueous phase is extracted with dichloromethane (3 x 10 mL), the organic layers were dried with Na_2SO_4 and the solvent was removed in vacuo. The crude product was purified via column chromatography over Alox N (pentane/ethyl acetate: 5:0.5) and obtained as a yellow solid (547 mg, 1.54 mmol, 77 %).

^{1}H NMR (DMSO-d$_6$, 400 MHz, 293 K): δ = 7.51 (d, J = 4.9 Hz, 1H), 7.37 (d, J = 3.6 Hz, 1H), 7.24 (t, J = 7.9 Hz, 1H), 7.19 (t, J = 7.8 Hz, 1H), 7.15 – 7.08 (m, 4H), 7.07 – 6.99 (m, 2H), 6.98 (d, J = 8.1 Hz, 1H), 6.89 (t, J = 7.5 Hz, 1H), 6.78 (d, J = 4.4 Hz, 1H), 6.62 (dd, J = 10.0, 7.9 Hz, 2H), 6.44 (t, J = 7.4 Hz, 1H), 5.36 (d, J = 3.5 Hz, 1H) ppm.

^{13}C NMR (DMSO-d$_6$, 101 MHz, 293 K): δ = 147.26, 142.65, 140.32, 140.17, 134.32, 127.98, 127.19, 126.96, 126.52, 126.34, 125.65, 125.38, 121.42, 118.18, 116.08, 115.46, 113.84, 113.76, 105.73, 105.56, 62.98, 60.58 ppm.

Elemental analysis calculated: C 74.34, H 4.82, N 11.82

Elemental analysis found: C 73.93, H 4.88, N 11.36

Synthesis of **B2k**

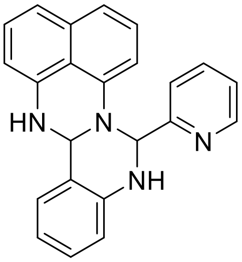

Chemical Formula: $C_{23}H_{18}N_4$

In a glovebox, 1,8-diaminonaphthalene (2 mmol, 316 mg), 2-aminobenzyl alcohol (2 mmol, 247 mg), KO^{t}Bu (0.6 mmol, 67 mg, 30 mol%), Mn-**I** (0.02 mmol, 12 mg, 1 mol%) and 3 mL 2-MeTHF are added to a Schlenk tube. The reaction mixture is heated at 100 °C using an open system consisting of a reflux condenser and a bubble counter. After 2 hours reaction time, 2-pyridinecarboxaldehyde (2 mmol, 191 µL) is diluted in 0.5 mL 2-MeTHF and added with a syringe to the reaction mixture via a septum. After stirring for 15 h, the mixture is cooled down to room temperature. The product precipitates during reaction. For purification it was filtrated, washed with H_2O and cold diethyl ether and dried at 70 °C in vacuo. The product is obtained as a light pink solid (470 mg, 1.34 mmol, 67 %).

^{1}H NMR (DMSO-d$_6$, 500 MHz, 293 K): δ = 8.59 (d, J = 4.1 Hz, 1H), 7.84 (t, J = 7.2 Hz, 1H), 7.57 (d, J = 7.8 Hz, 1H), 7.34 (dd, J = 7.0, 5.0 Hz, 1H), 7.28 – 7.15 (m, 4H), 7.12 (d, J = 8.1 Hz, 1H), 7.00 (d, J = 8.1 Hz, 1H), 6.98 – 6.88 (m, 3H), 6.64 (d, J = 7.0 Hz, 2H), 6.53 – 6.41 (m, 2H), 5.32 (d, J = 2.7 Hz, 1H) ppm.

^{13}C NMR (DMSO-d$_6$, 125 MHz, 293 K): δ = 160.39, 149.37, 143.07, 141.23, 140.43, 137.00, 134.34, 128.03, 126.91, 126.59, 125.51, 123.02, 122.09, 121.44, 117.74, 115.77, 115.53, 113.81, 113.61, 105.52, 105.26, 67.17, 60.79 ppm.

Elemental analysis calculated: C 78.83, H 5.18, N 15.99

Elemental analysis found: C 78.35, H 5.11, N 15.70

Synthesis of **B2l**

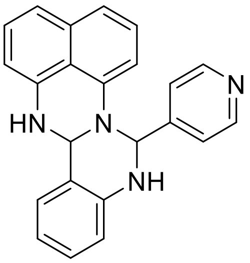

Chemical Formula: $C_{23}H_{18}N_4$

In a glovebox, 1,8-diaminonaphthalene (2 mmol, 316 mg), 2-aminobenzyl alcohol (2 mmol, 247 mg), KO^{t}Bu (0.6 mmol, 67 mg, 30 mol%), Mn-**I** (0.02 mmol, 12 mg, 1 mol%) and 3 mL 2-MeTHF are added to a Schlenk tube. The reaction mixture is heated at 100 °C using an open system consisting of a reflux condenser and a bubble counter. After 2 hours reaction time, 4-pyridinecarboxaldehyde (2 mmol, 189 µL) is diluted in 0.5 mL 2-MeTHF and added with a syringe to the reaction mixture via a septum. After stirring for 15 h, the mixture is cooled down to room temperature. The product precipitates during reaction. For purification it was filtrated, washed with H_2O and cold diethyl ether and dried at 70 °C in vacuo. The product is obtained as a light pink solid (554 mg, 1.58 mmol, 79 %).

^{1}H NMR (DMSO-d$_6$, 500 MHz, 293 K): δ = 8.63 (d, J = 4.1 Hz, 2H), 7.51 (d, J = 4.4 Hz, 2H), 7.34 (d, J = 3.1 Hz, 1H), 7.25 (t, J = 7.9 Hz, 1H), 7.18 (t, J = 7.7 Hz, 1H), 7.13 (d, J = 8.1 Hz, 1H), 7.07 (t, J = 6.8 Hz, 2H), 7.02 (d, J = 4.2 Hz, 1H), 6.98 (d, J = 8.0 Hz, 1H), 6.89 (t, J = 7.4 Hz, 1H), 6.66 – 6.57 (m, 3H), 6.41 (t, J = 7.3 Hz, 1H), 5.04 (d, J = 2.8 Hz, 1H) ppm.

^{13}C NMR (DMSO-d$_6$, 125 MHz, 293 K): δ = 151.42, 150.12, 142.79, 140.76, 139.83, 134.28, 128.08, 126.97, 126.56, 125.44, 122.18, 121.53, 118.20, 115.94, 115.50, 113.73, 113.64, 105.66, 105.57, 64.88, 60.36 ppm.

Elemental analysis calculated: C 78.83, H 5.18, N 15.99

Elemental analysis found: C 78.79, H 5.10, N 15.53

Synthesis of **B2m**

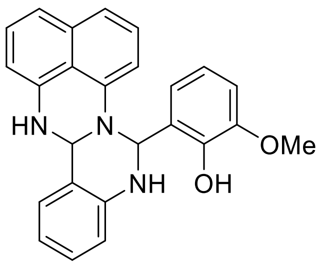

Chemical Formula: $C_{25}H_{21}N_3O_2$

In a glovebox, 1,8-diaminonaphthalene (2 mmol, 316 mg), 2-aminobenzyl alcohol (2 mmol, 247 mg), KO^{t}Bu (0.6 mmol, 67 mg, 30 mol%), Mn-**I** (0.02 mmol, 12 mg, 1 mol%) and 3 mL 2-MeTHF are added to a Schlenk tube. The reaction mixture is heated at 100 °C using an open system consisting of a reflux condenser and a bubble counter. After 2 hours reaction time, *o*-vanilin (2 mmol, 304 mg) is diluted in 1 mL 2-MeTHF and added with a syringe to the reaction mixture via a septum. After stirring for 15 h, the mixture is cooled down to room temperature and 2 mL H_2O is added. The aqueous phase is extracted with dichloromethane (3 x 10 mL), the organic layers were dried with Na_2SO_4 and the solvent was removed in vacuo. The crude product is purified by recrystallization in ethyl acetate at -4 °C. The product is obtained as yellow crystals (554 mg, 1.40 mmol, 70 %).

^{1}H NMR (DMSO-d$_6$, 500 MHz, 293 K): δ = 8.84 (s, 1H), 7.32 (d, J = 3.8 Hz, 1H), 7.23 (d, J = 7.9 Hz, 1H), 7.15 (t, J = 7.8 Hz, 1H), 7.08 (dd, J = 14.3, 7.9 Hz, 2H), 7.01 – 6.91 (m, 3H), 6.86 (d, J = 7.5 Hz, 2H), 6.78 (t, J = 7.9 Hz, 1H), 6.72 (s, 2H), 6.55 (dd, J = 14.2, 7.6 Hz, 2H), 6.39 (s, 1H), 5.32 (d, J = 3.7 Hz, 1H), 3.81 (s, 3H) ppm.

^{13}C NMR (DMSO-d$_6$, 125 MHz, 293 K): δ = 147.60, 143.68, 143.37, 140.89, 140.06, 134.33, 128.30, 127.99, 126.85, 126.64, 125.31, 121.34, 120.23, 118.19, 117.48, 115.31, 115.27, 113.77, 112.91, 111.50, 105.41, 105.25, 61.54, 59.90, 55.94 ppm.

Elemental analysis calculated: C 75.93, H 5.35, N 10.63

Elemental analysis found: C 75.79, H 5.05, N 10.55

Synthesis of **B2n**

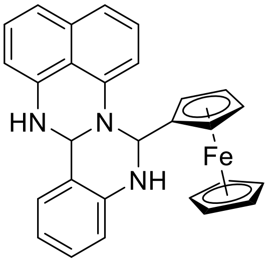

Chemical Formula: $C_{28}H_{23}FeN_3$

In a glovebox, 1,8-diaminonaphthalene (2 mmol, 316 mg), 2-aminobenzyl alcohol (2 mmol, 247 mg), KO^{t}Bu (0.6 mmol, 67 mg, 30 mol%), Mn-**I** (0.02 mmol, 12 mg, 1 mol%) and 3 mL 2-MeTHF are added to a Schlenk tube. The reaction mixture is heated at 100 °C using an open system consisting of a reflux condenser and a bubble counter. After 2 hours reaction time, ferrocenecarboxaldehyde (2 mmol, 428 mg) is diluted in 1.5 mL 2-MeTHF and added with a syringe to the reaction mixture via a septum. After stirring for 15 h, the mixture is cooled down to room temperature. The product precipitates during reaction. For purification it was filtrated, washed with H_2O and cold diethyl ether and dried at 70 °C in vacuo. The product is obtained as a pink solid (659 mg, 1.44 mmol, 72 %).

^{1}H NMR (DMSO-d$_6$, 500 MHz, 293 K): δ = 7.30 (d, J = 3.6 Hz, 1H), 7.25 (t, J = 7.9 Hz, 1H), 7.14 (t, J = 7.8 Hz, 1H), 7.07 (t, J = 7.0 Hz, 1H), 7.02 (d, J = 7.8 Hz, 1H), 6.93 (d, J = 8.1 Hz, 1H), 6.86 (t, J = 7.5 Hz, 1H), 6.79 (d, J = 4.2 Hz, 1H), 6.60 (d, J = 8.0 Hz, 1H), 6.55 (d, J = 7.3 Hz, 1H), 6.44 (d, J = 4.1 Hz, 1H), 6.36 (t, J = 7.4 Hz, 1H), 5.30 (d, J = 3.4 Hz, 1H), 4.35 (s, 3H), 4.21 (s, 1H), 4.13 (s, 1H) ppm.

^{13}C NMR (DMSO-d$_6$, 125 MHz, 293 K): δ = 143.34, 140.53, 140.02, 134.29, 127.86, 126.84, 126.52, 125.25, 121.15, 117.63, 115.22, 115.15, 113.61, 113.10, 105.45, 105.20, 89.74, 68.99, 68.72, 68.14, 66.83, 66.20, 63.43, 59.66 ppm.

Elemental analysis calculated: C 73.53, H 5.07, N 9.19

Elemental analysis found: C 72.85, H 5.07, N 8.99

Synthesis of **B2o**

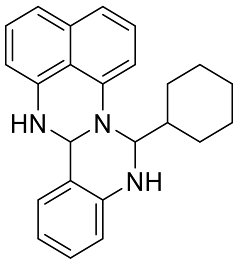

Chemical Formula: $C_{24}H_{25}N_3$

In a glovebox, 1,8-diaminonaphthalene (2 mmol, 316 mg), 2-aminobenzyl alcohol (2 mmol, 247 mg), KO^{t}Bu (0.6 mmol, 67 mg, 30 mol%), Mn-**I** (0.02 mmol, 12 mg, 1 mol%) and 3 mL 2-MeTHF are added to a Schlenk tube. The reaction mixture is heated at 100 °C using an open system consisting of a reflux condenser and a bubble counter. After 2 hours reaction time, cyclohexanecarboxaldehyde (2 mmol, 242 µL) is diluted in 0.5 mL 2-MeTHF and added with a syringe to the reaction mixture via a septum. After stirring for 15 h, the mixture is cooled down to room temperature and 2 mL H_2O is added. The aqueous phase is extracted with dichloromethane (3 x 10 mL), the organic layers were dried with Na_2SO_4 and the solvent was removed in vacuo. The crude product was purified via column chromatography over Alox N (pentane/ethyl acetate: 5:0.1) and obtained as a yellow solid (675 mg, 1.90 mmol, 95 %).

^{1}H NMR (DMSO-d$_6$, 500 MHz, 293 K): δ = 7.45 (d, J = 3.5 Hz, 1H), 7.21 – 7.09 (m, 2H), 7.00 (d, J = 8.1 Hz, 1H), 6.91 (d, J = 8.1 Hz, 1H), 6.78 (dd, J = 16.1, 7.7 Hz, 2H), 6.62 (d, J = 7.3 Hz, 1H), 6.58 (d, J = 4.2 Hz, 1H), 6.42 (d, J = 7.9 Hz, 1H), 6.33 (t, J = 7.3 Hz, 1H), 5.54 (d, J = 3.5 Hz, 1H), 4.86 (dd, J = 9.2, 4.2 Hz, 1H), 2.09 (d, J = 11.7 Hz, 1H), 1.91 (d, J = 12.8 Hz, 1H), 1.83 – 1.60 (m, 4H), 1.30 – 1.06 (m, 4H), 1.02 – 0.92 (m, 1H) ppm.

^{13}C NMR (DMSO-d$_6$, 125 MHz, 293 K): δ = 143.20, 141.54, 139.98, 134.32, 127.65, 126.79, 126.62, 125.17, 121.39, 116.98, 115.22, 114.64, 113.79, 113.02, 105.20, 104.58, 68.05, 59.65, 41.33, 29.29, 27.51, 26.13, 25.38, 25.29 ppm.

Elemental analysis calculated: C 81.09, H 7.09, N 11.82

Elemental analysis found: C 80.49, H 7.16, N 10.99

Synthesis of **B2p**

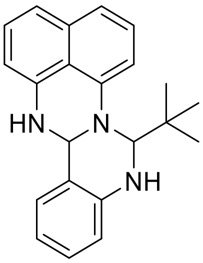

Chemical Formula: $C_{22}H_{23}N_3$

In a glovebox, 1,8-diaminonaphthalene (2 mmol, 316 mg), 2-aminobenzyl alcohol (2 mmol, 247 mg), KO^{t}Bu (0.6 mmol, 67 mg, 30 mol%), Mn-**I** (0.02 mmol, 12 mg, 1 mol%) and 3 mL 2-MeTHF are added to a Schlenk tube. The reaction mixture is heated at 100 °C using an open system consisting of a reflux condenser and a bubble counter. After 2 hours reaction time, pivalaldehyde (2 mmol, 125 µL) is diluted in 0.5 mL 2-MeTHF and added with a syringe to the reaction mixture via a septum. After stirring for 15 h, the mixture is cooled down to room temperature and 2 mL H_2O is added. The aqueous phase is extracted with dichloromethane (3 x 10 mL), the organic layers were dried with Na_2SO_4 and the solvent was removed in vacuo. The crude product is purified by recrystallization in dichloromethane/pentane at -4 °C. The product is obtained as green crystals (451 mg, 1.37 mmol, 68 %).

^{1}H NMR (DMSO-d_6, 500 MHz, 293 K): δ = 7.41 (d, J = 3.7 Hz, 1H), 7.21 (t, J = 7.8 Hz, 1H), 7.12 (t, J = 8.0 Hz, 1H), 7.01 – 6.91 (m, 3H), 6.87 (t, J = 7.5 Hz, 1H), 6.75 (d, J = 7.9 Hz, 1H), 6.70 (d, J = 7.4 Hz, 1H), 6.66 (d, J = 8.0 Hz, 1H), 6.41 (t, J = 7.4 Hz, 1H), 6.10 (d, J = 4.2 Hz, 1H), 5.29 (d, J = 3.6 Hz, 1H), 4.89 (d, J = 4.3 Hz, 1H), 1.11 (s, 9H) ppm.

^{13}C NMR (DMSO-d_6, 125 MHz, 293 K): δ = 145.35, 142.45, 140.58, 134.52, 127.31, 126.97, 126.83, 126.08, 123.28, 116.46, 115.81, 115.41, 113.95, 113.27, 105.11, 103.85, 73.64, 61.07, 37.95, 26.05 ppm.

Elemental analysis calculated: C 80.21, H 7.04, N 12.76

Elemental analysis found: C 80.09, H 6.79, N 12.57

Synthesis of **B3a**

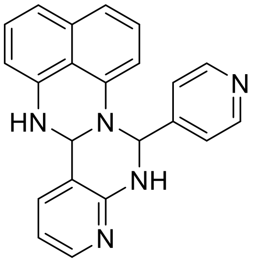

Chemical Formula: $C_{22}H_{17}N_5$

In a glovebox, 1,8-diaminonaphthalene (2 mmol, 316 mg), 2-amino-3-pyridinecarboxaldehyde (2 mmol, 225 mg), KO^{t}Bu (0.6 mmol, 67 mg, 30 mol%), Mn-**I** (0.02 mmol, 12 mg, 1 mol%) and 3 mL 2-MeTHF are added to a Schlenk tube. The reaction mixture is heated at 100 °C using an open system consisting of a reflux condenser and a bubble counter. After 2 hours reaction time, 4-pyridinecarboxaldehyde (2 mmol, 189 µL) is diluted in 0.5 mL 2-MeTHF and added with a syringe to the reaction mixture via a septum. After stirring for 15 h, the mixture is cooled down to room temperature. The product precipitates during reaction. For purification, it was filtrated, washed with H_2O and cold diethyl ether and dried at 70 °C in vacuo. The product is obtained as a light pink solid (632 mg, 1.80 mmol, 90 %).

^{1}H NMR (DMSO-d$_6$, 500 MHz, 293 K): δ = 8.65 (d, J = 5.1 Hz, 2H), 7.77 (dd, J = 12.8, 4.4 Hz, 2H), 7.52 (d, J = 5.2 Hz, 2H), 7.40 (d, J = 3.9 Hz, 1H), 7.34 (d, J = 7.3 Hz, 1H), 7.28 (t, J = 7.9 Hz, 1H), 7.19 (dd, J = 16.8, 8.3 Hz, 2H), 7.11 (d, J = 7.7 Hz, 1H), 7.02 (d, J = 8.1 Hz, 1H), 6.69 (d, J = 4.3 Hz, 1H), 6.64 (d, J = 7.4 Hz, 1H), 6.43 (dd, J = 7.2, 5.0 Hz, 1H), 5.02 (d, J = 3.8 Hz, 1H) ppm.

^{13}C NMR (DMSO-d$_6$, 125 MHz, 293 K): δ = 154.05, 150.73, 150.22, 147.33, 140.05, 139.16, 134.23, 133.03, 127.05, 126.59, 122.06, 118.76, 116.82, 115.89, 113.57, 112.57, 106.02, 105.93, 64.39, 60.02 ppm.

Elemental analysis calculated: C 75.19, H 4.88, N 19.93

Elemental analysis found: C 74.66 H 4.85 N 19.27

Synthesis of **B3b**

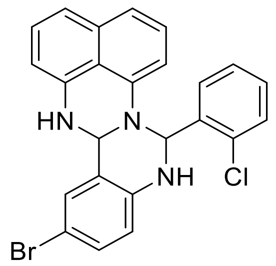

Chemical Formula: $C_{24}H_{17}BrClN_3$

In a glovebox, 1,8-diaminonaphthalene (2 mmol, 316 mg), 2-amino-5-bromobenzyl alcohol (2 mmol, 404 mg), KO^{t}Bu (0.6 mmol, 67 mg, 30 mol%), Mn-**I** (0.02 mmol, 12 mg, 1 mol%) and 3 mL 2-MeTHF are added to a Schlenk tube. The reaction mixture is heated at 100 °C using an open system consisting of a reflux condenser and a bubble counter. After 2 hours reaction time, 2-chlorobenzaldehyde (2 mmol, 225 µL) is diluted in 0.5 mL 2-MeTHF and added with a syringe to the reaction mixture via a septum. After stirring for 15 h, the mixture is cooled down to room temperature and 2 mL H_2O is added. The aqueous phase is extracted with dichloromethane (3 x 10 mL), the organic layers were dried with Na_2SO_4 and the solvent was removed in vacuo. The crude product was purified via column chromatography over Alox N (pentane/ethyl acetate: 5:2) and obtained as an orange solid (721 mg, 1.56 mmol, 78 %).

^{1}H NMR (DMSO-d$_6$, 500 MHz, 293 K): δ = 7.56 – 7.52 (m, 1H), 7.50 – 7.46 (m, 1H), 7.44 – 7.41 (m, 2H), 7.38 (d, J = 3.8 Hz, 1H), 7.29 (t, J = 7.9 Hz, 1H), 7.24 – 7.18 (m, 2H), 7.15 (d, J = 8.2 Hz, 1H), 7.10 (d, J = 7.7 Hz, 1H), 7.05 (d, J = 5.5 Hz, 2H), 7.01 (d, J = 8.1 Hz, 1H), 6.77 (d, J = 4.3 Hz, 1H), 6.61 (t, J = 7.5 Hz, 2H), 5.11 (d, J = 3.7 Hz, 1H) ppm.

^{13}C NMR (DMSO-d$_6$, 125 MHz, 293 K): δ = 142.67, 139.92, 139.19, 138.27, 134.34, 131.95, 130.80, 130.22, 129.97, 129.69, 127.75, 127.07, 126.77, 123.64, 118.24, 115.80, 115.18, 113.57, 106.54, 105.73, 105.42, 63.88, 59.26 ppm.

Elemental analysis calculated: C 62.29, H 3.70, N 9.08

Elemental analysis found: C 62.30, H 3.67, N 8.94

Synthesis of **B3c**

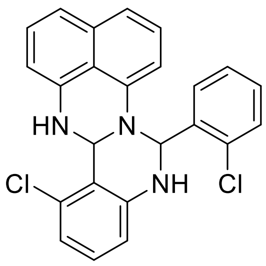

Chemical Formula: $C_{24}H_{17}Cl_2N_3$

In a glovebox, 1,8-diaminonaphthalene (2 mmol, 316 mg), 2-amino-6-chlorobenzyl alcohol (2 mmol, 315 mg), KO^{t}Bu (0.6 mmol, 67 mg, 30 mol%), Mn-**I** (0.02 mmol, 12 mg, 1 mol%) and 3 mL 2-MeTHF are added to a Schlenk tube. The reaction mixture is heated at 100 °C using an open system consisting of a reflux condenser and a bubble counter. After 2 hours reaction time, 2-chlorobenzaldehyde (2 mmol, 225 µL) is diluted in 0.5 mL 2-MeTHF and added with a syringe to the reaction mixture via a septum. After stirring for 15 h, the mixture is cooled down to room temperature and 2 mL H_2O is added. The aqueous phase is extracted with dichloromethane (3 x 10 mL), the organic layers were dried with Na_2SO_4 and the solvent was removed in vacuo. The crude product was purified via column chromatography over Alox N (pentane/ethyl acetate: 5:2) and obtained as a yellow solid (594 mg, 1.42 mmol, 71 %).

^{1}H NMR (DMSO-d$_6$, 500 MHz, 293 K): δ = 7.85 (d, J = 7.5 Hz, 1H), 7.47 (t, J = 7.5 Hz, 1H), 7.40 – 7.34 (m, 2H), 7.23 (dd, J = 15.5, 7.8 Hz, 2H), 7.13 (dd, J = 13.9, 5.5 Hz, 3H), 6.92 (t, J = 7.8 Hz, 1H), 6.81 (s, 1H), 6.77 – 6.69 (m, 3H), 6.11 (s, 1H), 5.93 (d, J = 7.1 Hz, 1H), 5.45 (s, 1H) ppm.

^{13}C NMR (DMSO-d$_6$, 125 MHz, 293 K): δ = 145.25, 141.84, 140.24, 137.25, 134.23, 132.15, 131.46, 130.29, 129.97, 128.98, 127.22, 126.69, 125.12, 122.93, 116.42, 116.03, 115.54, 112.72, 105.71, 65.08, 64.38 ppm.

Elemental analysis calculated: C 68.91, H 4.10, N 10.05

Elemental analysis found: C 68.91, H 4.11, N 10.04

Synthesis of **B3d**

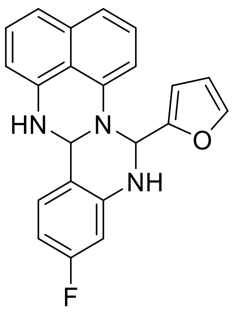

Chemical Formula: $C_{22}H_{16}FN_3O$

In a glovebox, 1,8-diaminonaphthalene (2 mmol, 316 mg), 2-amino-4-fluorobenzyl alcohol (2 mmol, 228 mg), KO^{t}Bu (0.6 mmol, 67 mg, 30 mol%), Mn-**I** (0.02 mmol, 12 mg, 1 mol%) and 3 mL 2-MeTHF are added to a Schlenk tube. The reaction mixture is heated at 100 °C using an open system consisting of a reflux condenser and a bubble counter. After 2 hours reaction time, furfural (2 mmol, 166 µL) is diluted in 0.5 mL 2-MeTHF and added with a syringe to the reaction mixture via a septum. After stirring for 15 h, the mixture is cooled down to room temperature and 2 mL H_2O is added. The aqueous phase is extracted with dichloromethane (3 x 10 mL), the organic layers were dried with Na_2SO_4 and the solvent was removed in vacuo. The crude product was purified via column chromatography over Alox N (pentane/ethyl acetate: 5:0.5) and obtained as an orange solid (607 mg, 1.80 mmol, 85 %).

^{1}H NMR (DMSO-d$_6$, 500 MHz, 293 K): δ = 7.71 (s, 1H), 7.32 (d, J = 3.4 Hz, 1H), 7.24 (t, J = 7.9 Hz, 1H), 7.17 (tt, J = 15.2, 7.5 Hz, 4H), 6.99 (dd, J = 12.0, 8.0 Hz, 2H), 6.63 (d, J = 7.4 Hz, 1H), 6.60 (d, J = 3.8 Hz, 1H), 6.47 (s, 1H), 6.42 (d, J = 3.0 Hz, 1H), 6.37 (d, J = 11.2 Hz, 1H), 6.24 (t, J = 8.7 Hz, 1H), 5.25 (d, J = 3.0 Hz, 1H) ppm.

^{13}C NMR (DMSO-d$_6$, 125 MHz, 293 K): δ = 163.25, 161.34, 153.54, 144.56, 144.47, 143.29, 140.08, 140.02, 134.30, 127.25, 127.17, 126.95, 126.52, 118.31, 117.23, 115.71, 113.90, 110.55, 109.45, 105.77, 102.37, 102.20, 99.70, 99.50, 61.29, 60.90 ppm.

^{19}F NMR (DMSO-d$_6$, 376 MHz, 293 K): δ = -114.72 (m) ppm.

Elemental analysis calculated: C 73.94, H 4.51, N 11.76

Elemental analysis found: C 73.49 H 4.43 N 11.70

Synthesis of **B3e**

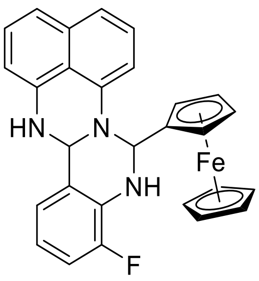

Chemical Formula: $C_{28}H_{22}FFeN_3$

In a glovebox, 1,8-diaminonaphthalene (2 mmol, 316 mg), 2-amino-3-fluorobenzyl alcohol (2 mmol, 228 mg), KO^{t}Bu (0.6 mmol, 67 mg, 30 mol%), Mn-**I** (0.01 mmol, 12 mg, 1 mol%) and 3 mL 2-MeTHF are added to a Schlenk tube. The reaction mixture is heated at 100 °C using an open system consisting of a reflux condenser and a bubble counter. After 2 hours reaction time, ferrocenecarboxaldehyde (2 mmol, 428 mg) is diluted in 0.5 mL 2-MeTHF and added with a syringe to the reaction mixture via a septum. After stirring for 15 h, the mixture is cooled down to room temperature and 2 mL H_2O is added. The aqueous phase is extracted with dichloromethane (3 x 10 mL), the organic layers were dried with Na_2SO_4 and the solvent was removed in vacuo. The crude product was purified via column chromatography over Alox N (pentane/ethyl acetate: 5:1→5:3) and obtained as an orange solid (713 mg, 1.50 mmol, 75 %).

^{1}H NMR (DMSO-d$_6$, 500 MHz, 293 K): δ = 7.39 (d, J = 4.0 Hz, 1H), 7.26 (t, J = 7.9 Hz, 1H), 7.18 – 7.08 (m, 1H), 7.07 (d, J = 24.0 Hz, 1H), 6.99 – 6.91 (m, 1H), 6.88 – 6.80 (m, 1H), 6.78 (d, J = 5.2 Hz, 1H), 6.55 (d, J = 7.4 Hz, 1H), 6.50 (d, J = 5.1 Hz, 1H), 6.36 (dd, J = 12.8, 7.8 Hz, 1H), 5.36 (d, J = 3.8 Hz, 1H), 4.38 (s, 1H), 4.35 (s, 2H), 4.23 (s, 1H), 4.14 (d, J = 11.9 Hz, 1H) ppm.

^{13}C NMR (DMSO-d$_6$, 125 MHz, 293 K): δ = 150.45, 148.55, 140.09, 139.61, 134.24, 131.50, 131.40, 126.92, 126.54, 124.31, 124.28, 121.10, 118.02, 115.40, 114.31, 114.25, 113.63, 113.50, 105.64, 105.39, 89.45, 69.03, 68.81, 68.02, 66.97, 66.28, 63.13, 59.33, 39.52 ppm.

^{19}F NMR (DMSO-d$_6$, 376 MHz, 293 K): δ = -136.35 (dd, J_1 = 11.2 Hz, J_2 = 4.6 Hz) ppm.

Elemental analysis calculated: C 70.75, H 4.67, N 8.84

Elemental analysis found: C 70.55, H 4.66, N 8.84

Synthesis of **B4a**

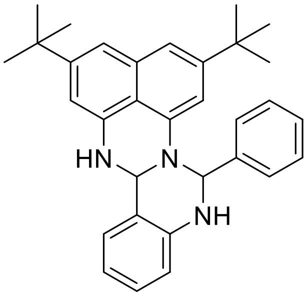

Chemical Formula: $C_{32}H_{35}N_3$

In a glovebox, 1,8-diamino-4,6-di-*tert*-butyl-naphthalene (1 mmol, 270.5 mg), 2-aminobenzyl alcohol (1 mmol, 123 mg), KO^{t}Bu (0.3 mmol, 33 mg, 30 mol%), Mn-**I** (0.01 mmol, 6 mg, 1 mol%) and 3 mL 2-MeTHF are added to a Schlenk tube. The reaction mixture is heated at 100 °C using an open system consisting of a reflux condenser and a bubble counter. After 6 hours reaction time, benzaldehyde (1 mmol, 108 µL) is diluted in 0.5 mL 2-MeTHF and added with a syringe to the reaction mixture via a septum. After stirring for 15 h, the mixture is cooled down to room temperature and 2 mL H_2O is added. The aqueous phase is extracted with dichloromethane (3x10 mL), the organic layers were dried with Na_2SO_4 and the solvent was removed in vacuo. The crude product was purified via column chromatography over Alox N (pentane/ethyl acetate: 5:1) and obtained as an orange solid (360 mg, 0.78 mmol, 78 %).

^{1}H NMR (DMSO-d$_6$, 500 MHz, 293 K): δ = 7.48 (d, J = 7.5 Hz, 1H), 7.40 (t, J = 7.6 Hz, 1H), 7.32 (t, J = 7.3 Hz, 1H), 7.08 (d, J = 7.5 Hz, 1H), 7.04 (dd, J = 4.5, 2.3 Hz, 1H), 6.95 – 6.86 (m, 1H), 6.63 (d, J = 10.4 Hz, 1H), 6.49 (d, J = 3.9 Hz, 1H), 6.42 (t, J = 7.4 Hz, 1H), 5.11 (d, J = 3.2 Hz, 1H), 1.30 (s, 1H), 1.28 (s, 1H) ppm.

^{13}C NMR (DMSO-d$_6$, 125 MHz, 293 K): δ = 149.10, 148.69, 143.36, 142.45, 140.37, 139.66, 133.97, 128.48, 128.01, 127.75, 127.27, 125.59, 121.48, 115.44, 114.01, 113.36, 111.51, 111.34, 105.40, 103.16, 99.54, 65.70, 61.19, 34.74, 34.39, 31.23, 31.13 ppm.

Elemental analysis calculated: C 83.26, H 7.64, N 9.10

Elemental analysis found: C 83.61, H 7.87, N 9.19

Synthesis of **B4b**

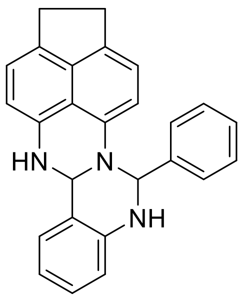

Chemical Formula: $C_{26}H_{21}N_3$

In a glovebox, 5,6-acenaphthenediamine (1 mmol, 184.3 mg), 2-aminobenzyl alcohol (1 mmol, 123 mg), KO^{t}Bu (0.3 mmol, 33 mg, 30 mol%), Mn-**I** (0.01 mmol, 6 mg, 1 mol%) and 3 mL 2-MeTHF are added to a Schlenk tube. The reaction mixture is heated at 100 °C using an open system consisting of a reflux condenser and a bubble counter. After 6 hours reaction time, benzaldehyde (1 mmol, 108 µL) is diluted in 0.5 mL 2-MeTHF and added with a syringe to the reaction mixture via a septum. After stirring for 15 h, the mixture is cooled down to room temperature and 2 mL H_2O is added. The aqueous phase is extracted with dichloromethane (3 x 10 mL), the organic layers were dried with Na_2SO_4 and the solvent was removed in vacuo. The crude product was isolated by precipitation in pentane and subsequent washing with water and drying in vacuo. A brown solid was obtained (210 mg, 0.56 mmol, 56 %).

^{1}H NMR (DMSO-d$_6$, 500 MHz, 293 K): δ = 7.50 (d, J = 7.5 Hz, 1H), 7.42 (t, J = 7.6 Hz, 1H), 7.34 (t, J = 7.3 Hz, 1H), 7.11 – 7.00 (m, 1H), 6.98 – 6.88 (m, 1H), 6.84 (t, J = 7.2 Hz, 1H), 6.58 (d, J = 7.6 Hz, 1H), 6.49 (t, J = 6.4 Hz, 1H), 6.35 (t, J = 7.1 Hz, 1H), 5.06 (d, J = 3.8 Hz, 1H), 3.15 (dd, J = 35.6, 13.0 Hz, 1H) ppm.

^{13}C NMR (DMSO-d$_6$, 125 MHz, 293 K): δ = 143.40, 142.55, 139.55, 138.08, 136.72, 134.58, 132.17, 128.53, 127.79, 127.68, 127.01, 125.64, 121.64, 119.88, 119.47, 115.28, 113.17, 112.55, 106.57, 106.17, 65.72, 60.96, 29.80, 29.73 ppm.

Elemental analysis calculated: C 83.17, H 5.64, N 11.19

Elemental analysis found: C83.15, H 5.81, N 11.25

Synthesis of **B4c**

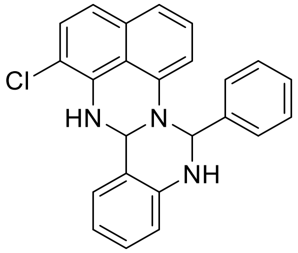

Chemical Formula: $C_{24}H_{18}ClN_3$

In a glovebox, 2-choro-1,8-diamino-naphthalene (1 mmol, 192.6 mg), 2-aminobenzyl alcohol (1 mmol, 123 mg), KO^{t}Bu (0.3 mmol, 33 mg, 30 mol%), Mn-**I** (0.01 mmol, 6 mg, 1 mol%) and 3 mL 2-MeTHF are added to a Schlenk tube. The reaction mixture is heated at 100 °C using an open system consisting of a reflux condenser and a bubble counter. After 6 hours reaction time, benzaldehyde (1 mmol, 108 µL) is diluted in 0.5 mL 2-MeTHF and added with a syringe to the reaction mixture via a septum. After stirring for 15 h, the mixture is cooled down to room temperature and 2 mL H_2O is added. The aqueous phase is extracted with dichloromethane (3 x 10 mL), the organic layers were dried with Na_2SO_4 and the solvent was removed in vacuo. The crude product was purified via column chromatography over Alox N (pentane/ethyl acetate: 5:2) and obtained as a yellow solid (272 mg, 0.71 mmol, 71 %).

^{1}H NMR (DMSO-d$_6$, 500 MHz, 293 K): δ = 7.53 (d, J = 7.3 Hz, 2H), 7.45 (t, J = 7.6 Hz, 2H), 7.40 – 7.23 (m, 4H), 7.18 (dd, J = 18.9, 7.9 Hz, 2H), 7.05 – 6.96 (m, 2H), 6.91 (d, J = 7.6 Hz, 1H), 6.87 (dd, J = 11.8, 4.5 Hz, 1H), 6.65 (d, J = 4.4 Hz, 1H), 6.61 (d, J = 7.5 Hz, 1H), 6.37 (dd, J = 10.8, 4.0 Hz, 1H), 5.23 (d, J = 4.5 Hz, 1H) ppm.

^{13}C NMR (DMSO-d$_6$, 125 MHz, 293 K): δ = 143.11, 142.25, 140.56, 135.34, 132.80, 128.67, 128.13, 127.81, 127.14, 126.98, 126.92, 125.15, 120.96, 118.12, 116.34, 115.51, 114.23, 113.34, 108.67, 106.83, 65.42, 59.89 ppm.

Elemental analysis calculated: C 75.09, H 4.73, N 10.95

Elemental analysis found: C 75.19, H 4.58, N 11.15

Synthesis of **B5a**

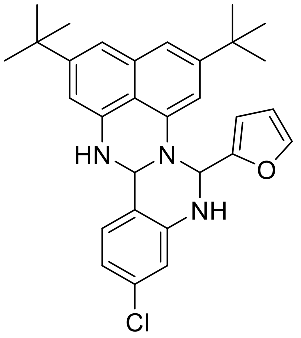

Chemical Formula: $C_{30}H_{32}ClN_3O$

In a glovebox, 1,8-diamino-4,6-di-*tert*-butyl-naphthalene (1 mmol, 270.5 mg), 2-amino-4-chloro-benzyl alcohol (1 mmol, 157 mg), KO^{t}Bu (0.3 mmol, 33 mg, 30 mol%), Mn-**I** (0.01 mmol, 6 mg, 1 mol%) and 3 mL 2-MeTHF are added to a Schlenk tube. The reaction mixture is heated at 100 °C using an open system consisting of a reflux condenser and a bubble counter. After 6 hours reaction time, furfural (1 mmol, 83 µL) is diluted in 0.5 mL 2-MeTHF and added with a syringe to the reaction mixture via a septum. After stirring for 15 h, the mixture is cooled down to room temperature and 2 mL H_2O is added. The aqueous phase is extracted with ethyl acetate (3 x 10 mL), the organic layers were dried with Na_2SO_4 and the solvent was removed in vacuo. The crude product was purified via column chromatography over Alox N (pentane/ethyl acetate: 5:2) and obtained as an orange solid (354 mg, 0.73 mmol, 73 %).

^{1}H NMR (DMSO-d_6, 500 MHz, 293 K): δ = 7.68 (d, J = 0.7 Hz, 1H), 7.15 (dd, J = 9.9, 6.0 Hz, 2H), 7.09 (d, J = 0.8 Hz, 1H), 7.03 (d, J = 3.0 Hz, 1H), 6.97 (d, J = 1.5 Hz, 1H), 6.89 (s, 1H), 6.67 (dd, J = 10.2, 1.8 Hz, 2H), 6.56 (d, J = 3.6 Hz, 1H), 6.50 (dd, J = 8.2, 2.0 Hz, 1H), 6.45 (dd, J = 3.1, 1.8 Hz, 1H), 6.40 (d, J = 3.2 Hz, 1H), 5.24 (d, J = 2.9 Hz, 1H), 1.30 (s, 20H) ppm.

^{13}C NMR (DMSO-d_6, 125 MHz, 293 K): δ = 153.56, 149.17, 148.71, 144.38, 143.14, 139.67, 139.39, 133.99, 132.44, 127.47, 119.69, 115.40, 114.36, 112.60, 111.99, 111.42, 110.51, 109.41, 105.37, 103.78, 61.93, 61.12, 34.80, 34.42, 31.21, 31.11 ppm.

Elemental analysis calculated: C 74.13, H 6.64, N 8.65

Elemental analysis found: C 74.33, H 6.63, N 8.42

Synthesis of **B5b**

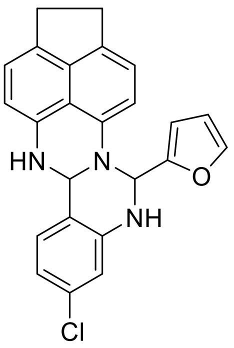

Chemical Formula: $C_{24}H_{18}ClN_3O$

In a glovebox, 5,6-acenaphthenediamine (1 mmol, 184.3 mg), 2-amino-4-chloro-benzyl alcohol (1 mmol, 157 mg), KO^{t}Bu (0.3 mmol, 33 mg, 30 mol%), Mn-**I** (0.01 mmol, 6 mg, 1 mol%) and 3 mL 2-MeTHF are added to a Schlenk tube. The reaction mixture is heated at 100 °C using an open system consisting of a reflux condenser and a bubble counter. After 6 hours reaction time, furfural (1 mmol, 83 µL) is diluted in 0.5 mL 2-MeTHF and added with a syringe to the reaction mixture via a septum. After stirring for 15 h, the mixture is cooled down to room temperature and 2 mL H_2O is added. The crude product was purified by precipitation in pentane and subsequent washing with water and drying in vacuo. An orange solid was obtained (272 mg, 0.68 mmol, 68 %).

^{1}H NMR (DMSO-d_6, 500 MHz, 293 K): δ = 7.71 (s, 1H), 7.14 (d, J = 4.1 Hz, 1H), 7.11 – 7.06 (m, 1H), 7.02 (d, J = 7.4 Hz, 1H), 6.98 (d, J = 7.3 Hz, 1H), 6.88 (d, J = 7.6 Hz, 1H), 6.57 (d, J = 2.0 Hz, 1H), 6.54 (t, J = 5.7 Hz, 1H), 6.47 (dd, J = 3.1, 1.8 Hz, 1H), 6.42 – 6.35 (m, 1H), 5.21 (d, J = 3.7 Hz, 1H), 3.23 – 3.04 (m, 1H) ppm.

^{13}C NMR (DMSO-d_6, 125 MHz, 293 K): δ = 153.73, 144.45, 143.28, 139.55, 136.83, 136.46, 135.15, 132.71, 132.22, 127.42, 119.99, 119.42, 115.13, 112.35, 110.53, 109.43, 106.82, 106.77, 67.03, 61.71, 61.55, 29.79, 29.72, 25.14 ppm.

Elemental analysis calculated: C 72.09, H 4.54, N 10.51

Elemental analysis found: C 72.26, H 4.52, N 10.73

Synthesis of **B5c**

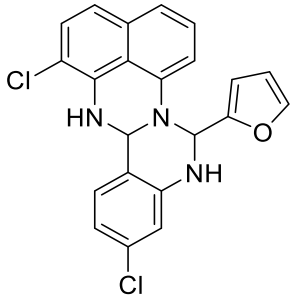

Chemical Formula: $C_{22}H_{15}Cl_2N_3O$

In a glovebox, 2-choro-1,8-diamino-naphthalene (1 mmol, 192.6 mg), 2-amino-4-chloro-benzyl alcohol (1 mmol, 157 mg), KO^{t}Bu (0.3 mmol, 33 mg, 30 mol%), Mn-**I** (0.01 mmol, 6 mg, 1 mol%) and 3 mL 2-MeTHF are added to a Schlenk tube. The reaction mixture is heated at 100 °C using an open system consisting of a reflux condenser and a bubble counter. After 6 hours reaction time, furfural (1 mmol, 83 µL) is diluted in 0.5 mL 2-MeTHF and added with a syringe to the reaction mixture via a septum. After stirring for 15 h, the mixture is cooled down to room temperature and 2 mL H_2O is added. The aqueous phase is extracted with DCM (3x10 mL), the organic layers were dried with Na_2SO_4 and the solvent was removed in vacuo. The crude product was purified via column chromatography over Alox N (pentane/ethyl acetate: 5:3) and obtained as a dark-orange solid (298 mg, 0.73 mmol, 73 %).

^{1}H NMR (DMSO-d$_6$, 500 MHz, 293 K): δ = 7.76 (s, 1H), 7.35 (d, J = 4.6 Hz, 1H), 7.30 (dd, J = 16.4, 8.4 Hz, 2H), 7.24 (d, J = 4.3 Hz, 1H), 7.19 (d, J = 8.1 Hz, 1H), 7.15 (d, J = 7.8 Hz, 1H), 7.05 (d, J = 8.8 Hz, 1H), 6.91 (d, J = 8.2 Hz, 1H), 6.73 (d, J = 4.2 Hz, 1H), 6.60 (d, J = 2.0 Hz, 1H), 6.51 (dd, J = 3.2, 1.8 Hz, 1H), 6.48 (d, J = 3.2 Hz, 1H), 6.43 (dd, J = 8.2, 2.0 Hz, 1H), 5.36 (d, J = 4.6 Hz, 1H) ppm.

^{13}C NMR (DMSO-d$_6$, 125 MHz, 293 K): δ = 153.39, 144.21, 143.61, 139.24, 134.98, 132.80, 132.52, 127.09, 127.07, 126.83, 119.47, 118.63, 116.76, 115.35, 114.22, 112.51, 110.64, 109.79, 109.17, 107.02, 61.52, 60.39 ppm.

Elemental analysis calculated (product + 1 ethyl acetate): C 62.91, H 4.67, N 8.47

Elemental analysis found: C 62.81, H 4.54, N, 8.67

Synthesis of **B6a**

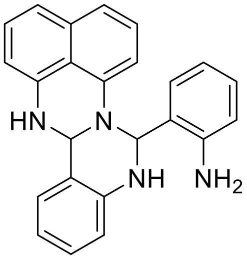

Chemical Formula: $C_{24}H_{20}N_4$

In a glovebox, Mn-**I** (0.02 mmol, 12.3 mg, dissolved in 0.5 mL 2-MeTHF), KO*ᵗ*Bu (0.6 mmol, 67 mg, dissolved in 0.5 mL 2-MeTHF), 1,8-diaminonaphthalene (2.0 mmol, 316 mg) and 2-aminobenzyl alcohol (2.0 mmol, 247 mg) are added to a Schlenk tube and dissolved in 3 mL 2-MeTHF. The reaction mixture is heated at 100 °C under light argon counter flow using an open system consisting of a reflux condenser and a bubble counter. After 2 hours reaction time, 2-aminobenzyl alcohol (2.0 mmol, 247 mg) is dissolved in 1.0 mL 2-MeTHF and added with a syringe to the reaction mixture via a septum. After 15 hours, the reaction is stopped by cooling to room temperature and 4 mL water are added. The reaction mixture is diluted with 15 mL pentane, the precipitate is filtrated and washed with water and pentane. The dried solid is mortared, slurred with ethanol and stirred for 10 min at 100 °C. After filtration, the product is dried in vacuo and obtained as light green solid (424 mg, 1.16 mmol, 58 %).

^{1}H NMR (DMSO-d_6, 500 MHz, 293 K): δ = 7.43 (d, J = 3.7 Hz, 1H), 7.28 (d, J = 7.8 Hz, 1H), 7.19 – 7.11 (m, 4H), 7.10 – 7.04 (m, 2H), 6.97 (d, J = 8.1 Hz, 1H), 6.89 – 6.82 (m, 2H), 6.78 (d, J = 7.8 Hz, 1H), 6.63 (t, J = 7.4 Hz, 1H), 6.58 (d, J = 7.0 Hz, 2H), 6.40 – 6.35 (m, 2H), 5.20 (d, J = 3.7 Hz, 1H), 4.97 (s, 2H) ppm.

^{13}C NMR (DMSO-d_6, 125 MHz, 293 K): δ = 145.53, 143.35, 140.26, 139.82, 134.35, 128.59, 128.20, 128.10, 126.99, 126.63, 125.35, 124.40, 120.96, 118.33, 116.41, 115.94, 115.50, 115.39, 114.13, 113.05, 106.14, 105.49, 63.73, 60.44 ppm.

LC-HRMS (ESI+) *m/z* calculated for $[C_{24}H_{21}N_4]^+$: 365.17607, found: 365.17610.

Synthesis of **B6b**

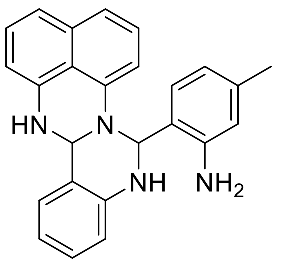

Chemical Formula: $C_{25}H_{22}N_4$

In a glovebox, Mn-**I** (0.02 mmol, 12.3 mg, dissolved in 0.5 mL 2-MeTHF), KO^{t}Bu (0.6 mmol, 67 mg, dissolved in 0.5 mL 2-MeTHF), 1,8-diaminonaphthalene (2.0 mmol, 316 mg) and 2-aminobenzyl alcohol (2.0 mmol, 247 mg) are added to a Schlenk tube and dissolved in 3 mL 2-MeTHF. The reaction mixture is heated at 100 °C under light argon counter flow using an open system consisting of a reflux condenser and a bubble counter. After 2 hours reaction time, 2-amino-4-methylbenzyl alcohol (2.2 mmol, 302 mg) is added to the reaction mixture via a funnel under argon counter flow and diluted with 1.0 mL 2-MeTHF. After 15 hours, the reaction is stopped by cooling to room temperature and 4 mL water are added. The reaction mixture is diluted with 15 mL pentane, the precipitate is filtrated and washed with water and pentane. The dried solid is mortared, slurred with ethanol and stirred for 10 min at 100 °C. After filtration, the product is dried in vacuo and obtained as dark green solid (287 mg, 0.76 mmol, 38 %).

^{1}H NMR (DMSO-d$_6$, 500 MHz, 293 K): δ = 7.42 (d, J = 3.7 Hz, 1H), 7.29 – 7.26 (m, 1H), 7.18 – 7.10 (m, 3H), 7.04 (d, J = 7.6 Hz, 2H), 6.96 (d, J = 8.1 Hz, 1H), 6.85 (t, J = 7.6 Hz, 1H), 6.81 (d, J = 4.0, 1H), 6.61 – 6.55 (m, 3H), 6.44 (d, J = 7.5 Hz, 1H), 6.38 – 6.33 (m, 2H), 5.19 (d, J = 3.4 Hz, 1H), 4.88 (s, 2H), 2.19 (s, 3H) ppm.

^{13}C NMR (DMSO-d$_6$, 125 MHz, 293 K): δ = 145.33, 143.37, 140.33, 139.86, 137.66, 134.34, 128.22, 128.05, 126.96, 126.63, 125.33, 121.79, 120.98, 118.25, 117.28, 116.51, 115.42, 115.36, 114.13, 113.03, 106.10, 105.44, 63.59, 60.35, 20.93 ppm.

LC-HRMS (ESI+) *m/z* calculated for $[C_{25}H_{23}N_4]^+$: 379.19172, found: 379.19267.

Synthesis of **B6c**

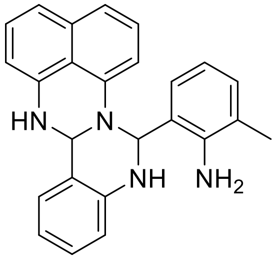

Chemical Formula: $C_{25}H_{22}N_4$

In a glovebox, Mn-**I** (0.02 mmol, 12.3 mg, dissolved in 0.5 mL 2-MeTHF), KO^{t}Bu (0.6 mmol, 67 mg, dissolved in 0.5 mL 2-MeTHF), 1,8-diaminonaphthalene (2.0 mmol, 316 mg) and 2-aminobenzyl alcohol (2.0 mmol, 247 mg) are added to a Schlenk tube and dissolved in 3 mL 2-MeTHF. The reaction mixture is heated at 100 °C under light argon counter flow using an open system consisting of a reflux condenser and a bubble counter. After 2 hours reaction time, 2-amino-3-methylbenzyl alcohol (2.2 mmol, 302 mg) is dissolved in 1.0 mL 2-MeTHF and added with a syringe to the reaction mixture via a septum. After 15 hours, the reaction is stopped by cooling to room temperature and 4 mL water are added. The reaction mixture is diluted with 15 mL pentane, the precipitate is filtrated and washed with water and pentane. The dried solid is mortared, slurred with ethanol and stirred for 10 min at 100 °C. After filtration, the product is dried in vacuo and obtained as greyish green solid (602 mg, 1.59 mmol, 79 %).

^{1}H NMR (DMSO-d_6, 500 MHz, 293 K): δ = 7.43 (s, 1H), 7.30 (t, J = 7.5 Hz, 1H), 7.20 – 7.13 (m, 3H), 7.07 (dd, J_1 = 11.7 Hz, J_2 = 8.0 Hz, 2H), 7.02 (d, J = 6.9 Hz, 1H), 6.98 (d, J = 8.1 Hz, 1H), 6.90 – 6.83 (m, 2H), 6.59 (d, J = 7.0 Hz, 3H), 6.43 – 6.35 (m, 2H), 5.21 (s, 1H), 4.75 (s, 2H), 2.15 (s, 3H) ppm.

^{13}C NMR (DMSO-d_6, 125 MHz, 293 K): δ = 143.35, 143.19, 140.21, 139.78, 134.35, 129.85, 128.11, 127.02, 126.62, 126.16, 125.33, 123.89, 122.36, 120.89, 118.42, 116.10, 115.46, 115.39, 114.12, 113.00, 106.09, 105.49, 63.91, 60.47, 17.53 ppm.

LC-HRMS (ESI+) *m/z* calculated for $[C_{25}H_{23}N_4]^+$: 379.19172, found: 379.19234/ 379.19238.

Synthesis of **C1**

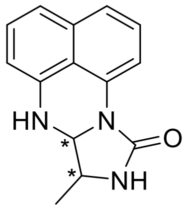

Chemical Formula: $C_{14}H_{13}N_3O$

In a glovebox, **A25** (2.0 mmol, 426 mg), KO^{t}Bu (0.6 mmol, 67 mg, dissolved in 1.5 mL 1,4-dioxane) and carbonyldiimidazol (2.3 mmol, 373 mg) are added to a pressure tube and dissolved in 8.5 mL 1,4-dioxane. The sealed pressure tube is heated at 130 °C for 2h in an oil bath. After cooling down to room temperature 30 mL water were added and the product is extracted with diethyl ether (4 x 50 mL). The combined organic phases are dried with Na_2SO_4 and the solvent was removed in vacuo. The crude product was purified via gradient column chromatography over Alox N (pentane/ethyl acetate 1:1 → pure ethyl acetate) and obtained as light brown solid (406 mg, 1.7 mmol, 85 %, contains ~ 2 % 1,4-dioxane). Diastereomeric ratio: 71:29.

Main isomer: **^{1}H NMR** (DMSO-d_6, 500 MHz, 293 K): δ = 7.62 (dd, J_1 = 7.3 Hz, J_2 = 1.2 Hz, 1H), 7.44 – 7.36 (m, 3H), 7.29 – 7.25 (m, 1H), 7.19 (dd, J_1 = 8.2 Hz, J_2 = 0.7 Hz, 1H), 6.98 (s, 1H), 6.64 (dd, J_1 = 7.3 Hz, J_2 = 0.9 Hz, 1H), 4.68 (d. J = 4.1 Hz, 1H), 3.63 – 3.57 (m, 1H), 1.31 (d, J = 6.3 Hz, 3H) ppm.

^{13}C NMR (DMSO-d_6, 125 MHz, 293 K): δ = 156.53, 141.37, 134.06, 132.68, 126.14, 121.25, 116.96, 114.72, 111.70, 106.87, 70.83, 51.41, 19.91 ppm.

Minor isomer: **^{1}H NMR** (DMSO-d_6, 500 MHz, 293 K): δ = 7.71 (dd, J_1 = 7.3 Hz, J_2 = 1.2 Hz, 1H), 7.44 – 7.36 (m, 1H), 7.36 – 7.34 (m, 1H), 7.33 (s, 1H), 7.29 – 7.25 (m, 1H), 7.18 (dd, J_1 = 8.2 Hz, J_2 = 0.7 Hz, 1H), 6.73 (dd, J_1 = 7.5 Hz, J_2 = 0.9 Hz, 1H), 6.63 (s, 1H), 5.07 (d. J = 7.2 Hz, 1H), 3.95 (quin. J = 6.6 Hz, 1H), 1.25 (d, J = 6.6 Hz, 3H) ppm.

^{13}C NMR (DMSO-d_6, 125 MHz, 293 K): δ = 156.63, 141.79, 133.96, 133.16, 126.92, 120.97, 116.80, 114.03, 110.92, 107.26, 66.20, 47.92, 15.52 ppm.

LC-HRMS (ESI+) *m/z* calculated for $[C_{14}H_{14}N_3O]^+$: 240.11314, found: 240.11347.

Synthesis of **C2**

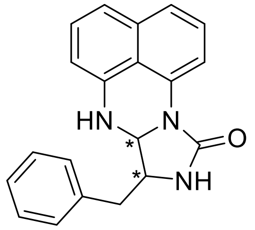

Chemical Formula: $C_{20}H_{17}N_3O$

In a glovebox, **A26** (2.0 mmol, 578 mg), KO^{t}Bu (0.6 mmol, 67 mg, dissolved in 1.5 mL 1,4-dioxane) and carbonyldiimidazol (2.3 mmol, 373 mg) are added to a pressure tube and dissolved in 8.5 mL 1,4-dioxane. The sealed pressure tube is heated at 130 °C for 2h in an oil bath. After cooling down to room temperature 30 mL water were added and the product is extracted with diethyl ether (4 x 50 mL). The combined organic phases are dried with Na_2SO_4 and the solvent was removed in vacuo. The crude product was purified via gradient column chromatography over Alox N (pentane/ethyl acetate 1:1 → pure ethyl acetate) and obtained as reddish brown solid (573 mg, 1.82 mmol, 91 %, contains ~ 5% ethyl acetate). Diastereomeric ratio: 88:12.

Main isomer: **^{1}H NMR** (DMSO-d$_6$, 500 MHz, 293 K): δ = 7.49 (s, 1H), 7.46 – 7.42 (m, 2H), 7.36 (t, J = 7.8 Hz, 1H), 7.34 – 7.30 (m, 4H), 7.27 – 7.23 (m, 2H), 7.20 – 7.18 (m, 1H), 6.83 (s, 1H), 6.62 (d, J = 7.3 Hz, 1H), 4.78 (d, J = 3.1 Hz, 1H), 3.84 – 3.78 (m, 1H), 3.03 – 2.88 (m, 2H) ppm.

^{13}C NMR (DMSO-d$_6$, 125 MHz, 293 K): δ = 156.84, 141.29, 136.92, 134.01, 132.40, 129.62, 128.42, 126.88, 126.53, 126.05, 121.66, 116.98, 115.07, 113.01, 106.95, 68.20, 56.30, 39.64 ppm.

LC-HRMS (ESI+) *m/z* calculated for $[C_{20}H_{18}N_3O]^+$: 316.14444, found: 316.14440.

Minor isomer: **^{1}H NMR** (DMSO-d$_6$, 500 MHz, 293 K): δ = 7.76 (dd, J_1 = 7.4 Hz, J_2 = 1.1 Hz, 1H), 7.42 (dd, J_1 = 8.2 Hz, J_2 = 1.1 Hz, 1H), 7.39 – 7.35 (m, 1H), 7.34 – 7.31 (m, 4H), 7.29 (d, J = 7.5 Hz, 1H), 7.26 – 7.23 (m, 2H), 7.21 (d, J = 7.6 Hz, 1H), 6.77 (dd, J_1 = 7.4 Hz, J_2 = 0.8 Hz, 1H), 6.76 (s, 1H), 5.22 (d, J = 7.2 Hz, 1H), 4.15 – 4.09 (m, 1H), 3.23 (dd, J_1 = 14.0 Hz, J_2 = 3.4 Hz, 1H), 2.76 (dd, J_1 = 14.0 Hz, J_2 = 10.0 Hz, 1H) ppm.

^{13}C NMR (DMSO-d$_6$, 125 MHz, 293 K): δ = 156.56, 141.58, 138.14, 133.97, 133.19, 129.36, 128.45, 126.94, 126.29, 126.20, 120.98, 116.97, 114.03, 110.74, 107.41, 66.50, 53.46, 35.81 ppm.

LC-HRMS (ESI+) *m/z* calculated for $[C_{20}H_{18}N_3O]^+$: 316.14444, found: 316.14431.

Synthesis of **C3**

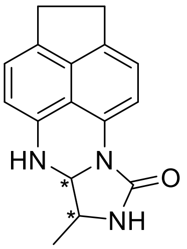

Chemical Formula: $C_{16}H_{15}N_3O$

In a glovebox, **A27** (2.0 mmol, 478 mg), KO^tBu (0.6 mmol, 67 mg, dissolved in 1.5 mL 1,4-dioxane) and carbonyldiimidazol (2.3 mmol, 373 mg) are added to a pressure tube and dissolved in 8.5 mL 1,4-dioxane. The sealed pressure tube is heated at 130 °C for 2h in an oil bath. After cooling down to room temperature 30 mL water were added and the product is extracted with diethylether (4 x 50 mL). The combined organic phases are dried with Na_2SO_4 and the solvent was removed in vacuo. The crude product was purified via gradient column chromatography over Alox N (pentane/ethyl acetate 1:1 → pure ethyl acetate) and obtained as light brown solid (403 mg, 1.52 mmol, 76 %, contains ~ 5% ethyl acetate). Diastereomeric ratio: 81:19.

Main isomer: **^{1}H NMR** (DMSO-d_6, 500 MHz, 293 K): δ = 7.50 (d, J = 7.5 Hz, 1H), 7.28 (s, 1H), 7.15 (d, J = 7.5 Hz, 1H), 7.07 (d, J = 7.2 Hz, 1H), 6.76 (s, 1H), 6.55 (d, J = 7.3 Hz, 1H), 4.64 (dd, J_1 = 5.0 Hz, J_2 = 0.9 Hz, 1H), 3.63 – 3.57 (m, 1H), 3.29 – 3.21 (m, 4H), 1.32 (d, J = 6.4 Hz, 3H) ppm.

^{13}C NMR (DMSO-d_6, 125 MHz, 293 K): δ = 156.48, 139.34, 138.04, 137.95, 133.88, 129.49, 120.05, 119.27, 113.18, 111.39, 107.68, 72.21, 51.72, 30.13, 29.68, 19.76 ppm.

LC-HRMS (ESI+) *m/z* calculated for $[C_{16}H_{16}N_3O]^+$: 266.12879, found: 266.12865.

Minor isomer: **^{1}H NMR** (DMSO-d_6, 500 MHz, 293 K): δ = 7.56 (d, J = 7.5 Hz, 1H), 7.23 (s, 1H), 7.14 (d, J = 7.3 Hz, 1H), 7.07 (d, J = 7.3 Hz, 1H), 6.61 (d, J = 7.3 Hz, 1H), 6.47 (s, 1H), 5.03 (d, J = 7.3 Hz, 1H), 3.95 (quin, J = 6.7 Hz, 1H), 3.29 – 3.20 (m, 4H), 1.23 (d, J = 6.4 Hz, 3H) ppm.

^{13}C NMR (DMSO-d_6, 125 MHz, 293 K): δ = 156.56, 139.23, 138.38, 137.76, 133.55, 129.98, 120.05, 119.22, 112.44, 110.79, 107.80, 67.45, 48.20, 30.13, 29.68, 15.69 ppm.

LC-HRMS (ESI+) *m/z* calculated for $[C_{16}H_{16}N_3O]^+$: 266.12879, found: 266.12852.

14. NMR spectra of isolated products

NMR spectra of A1

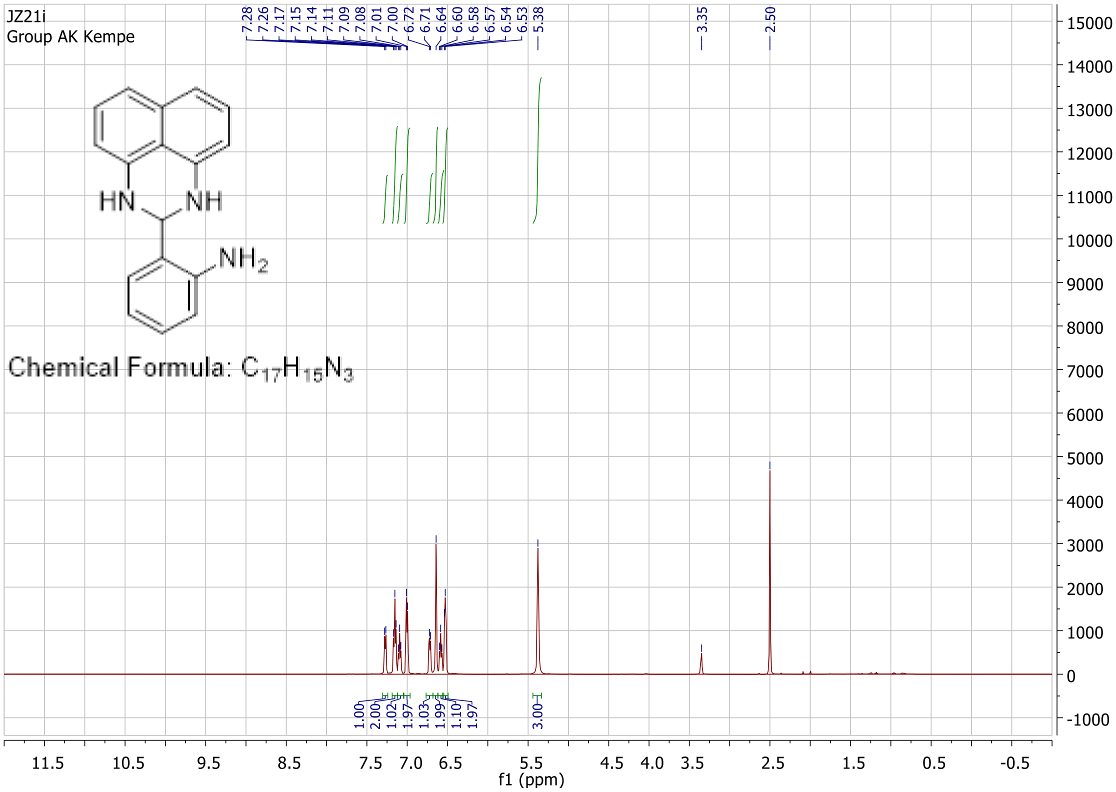

Supplementary Figure 31 ^{1}H NMR spectrum of compound **A1**. (500 MHz, 293 K, DMSO-d_6).

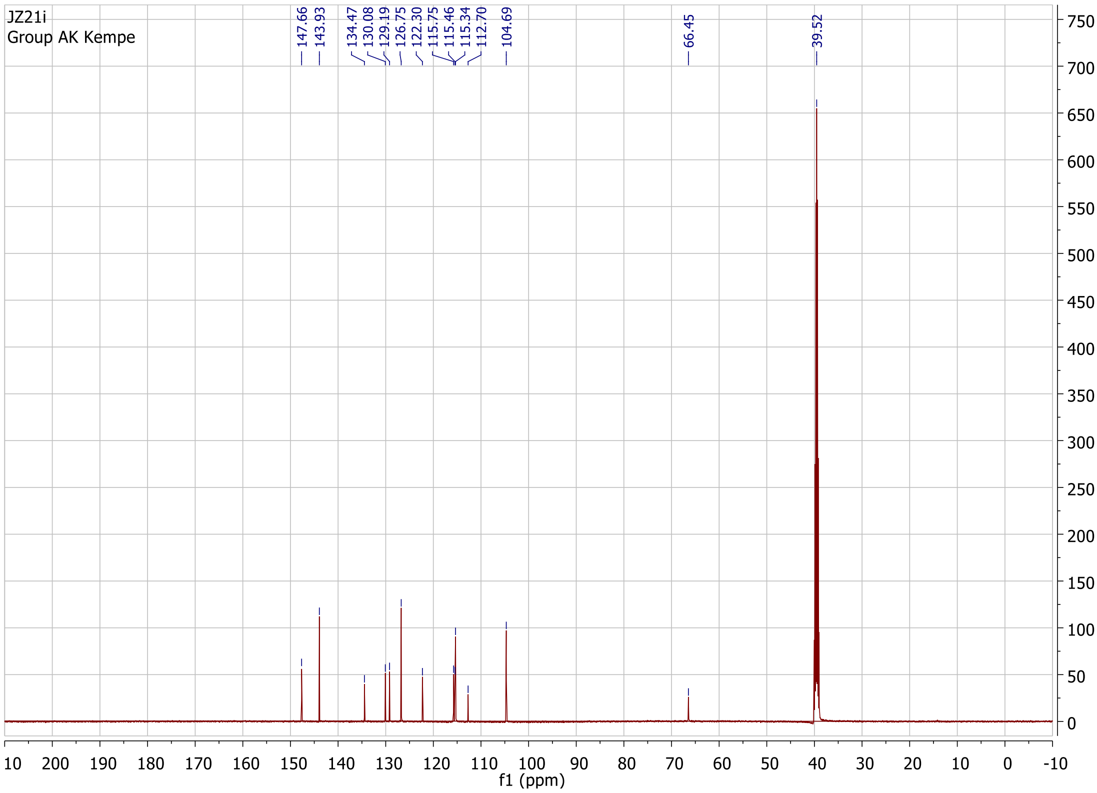

Supplementary Figure 32 ^{13}C NMR spectrum of compound **A1**. (125 MHz, 293 K, DMSO-d_6).

NMR spectra of A2

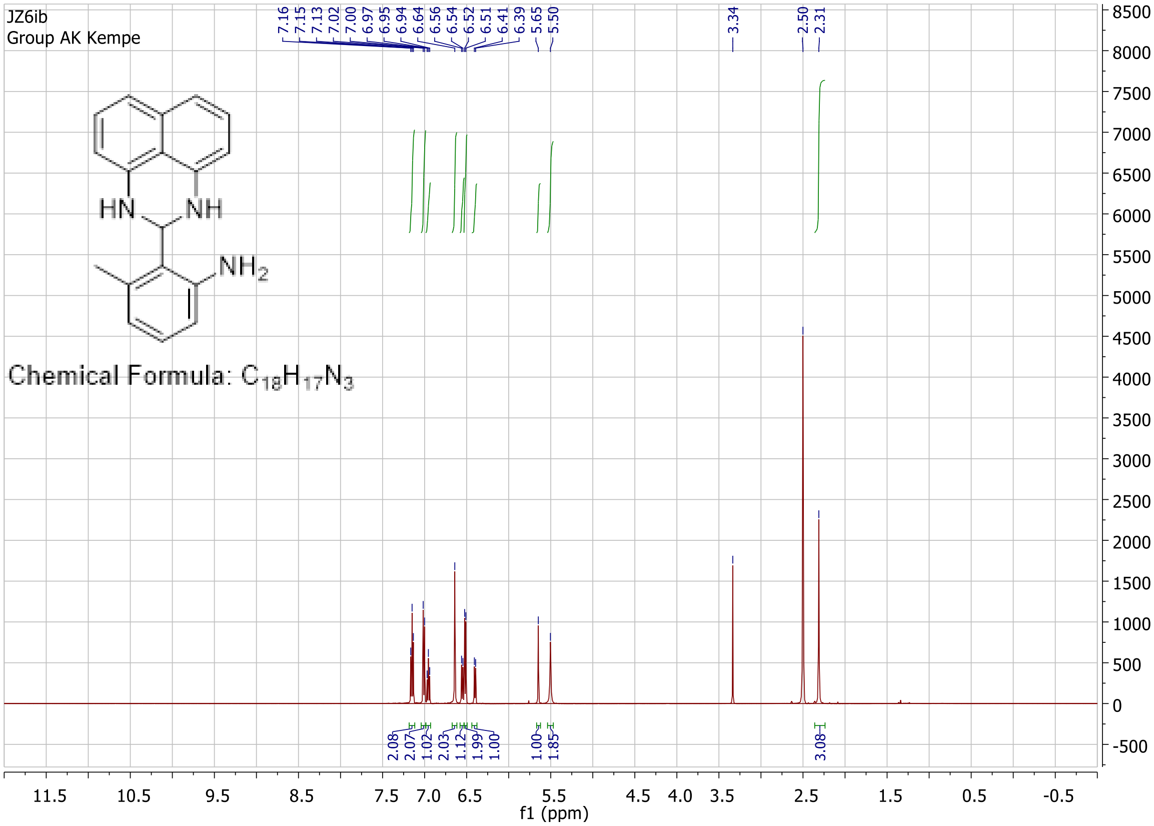

Supplementary Figure 33 ^{1}H NMR spectrum of compound **A2**. (500 MHz, 293 K, DMSO-d_6).

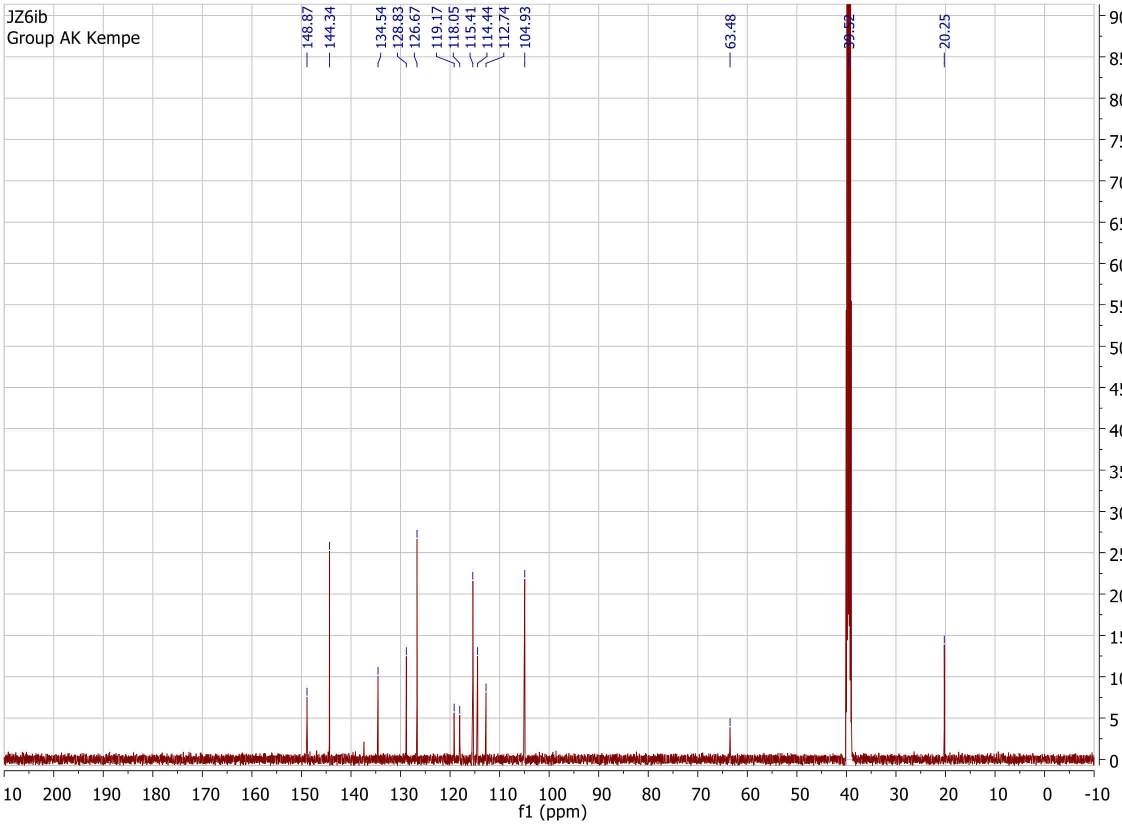

Supplementary Figure 34 ^{13}C NMR spectrum of compound **A2**. (125 MHz, 293 K, DMSO-d_6).

NMR spectra of A3

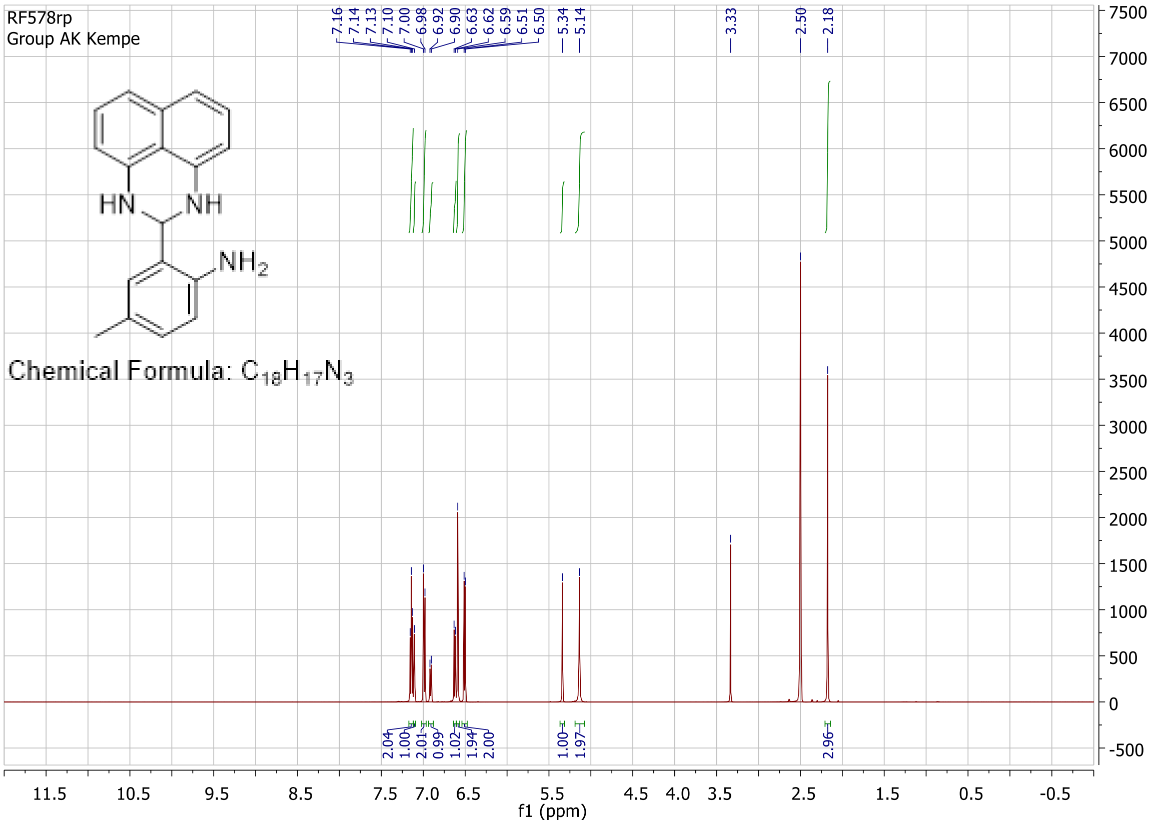

Supplementary Figure 35 ^{1}H NMR spectrum of compound **A3**. (500 MHz, 293 K, DMSO-d_6).

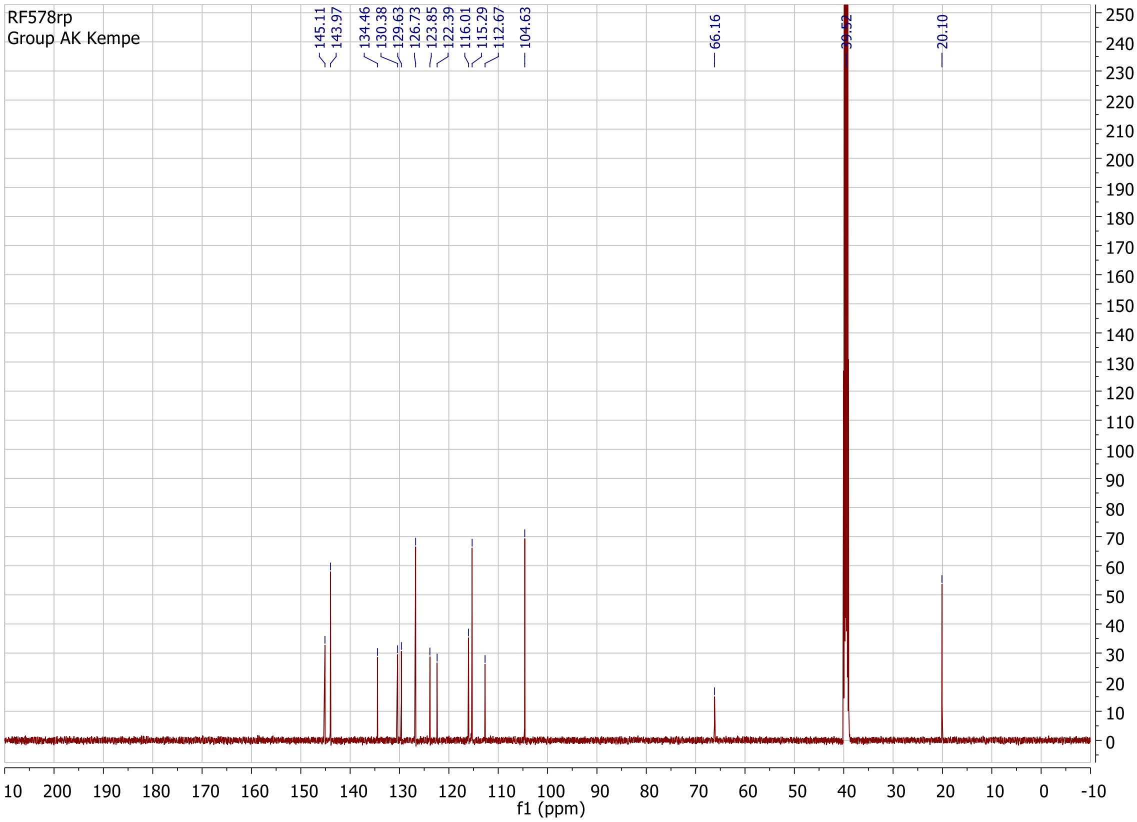

Supplementary Figure 36 ^{13}C NMR spectrum of compound **A3**. (125 MHz, 293 K, DMSO-d_6).

NMR spectra of A4

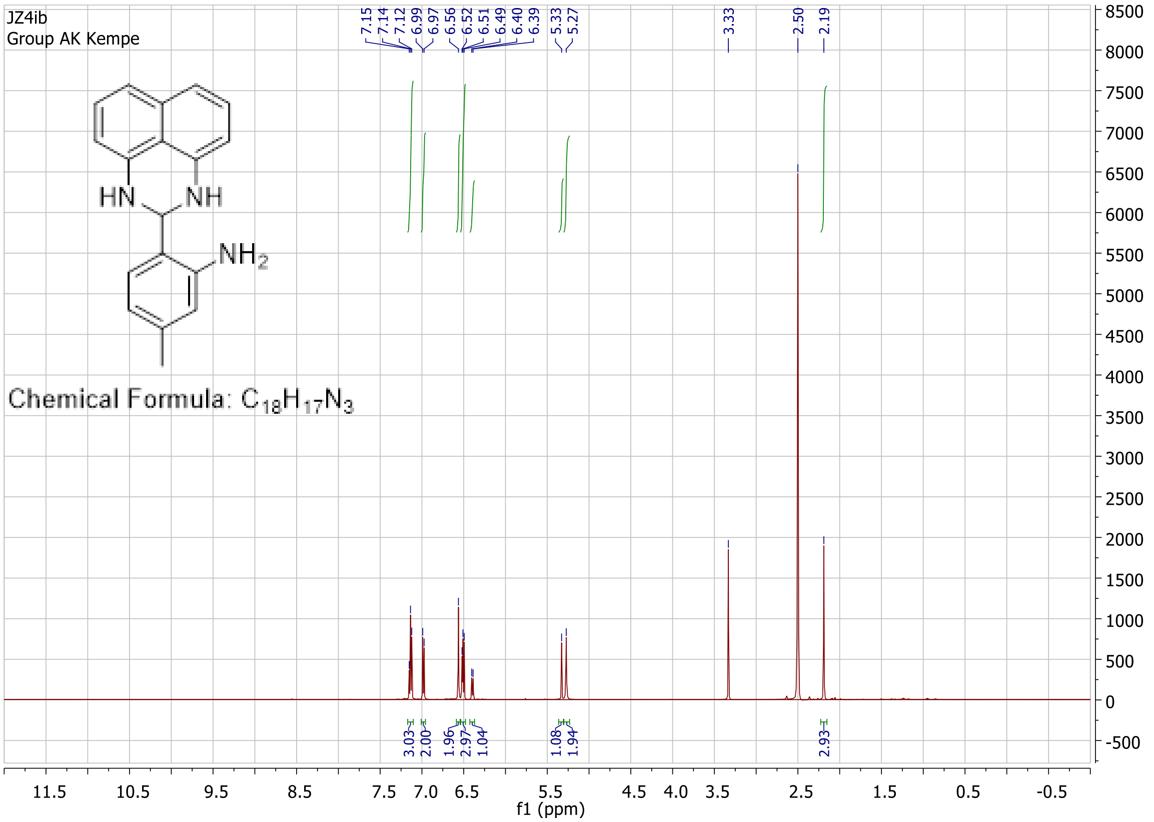

Supplementary Figure 37 ^{1}H NMR spectrum of compound **A4**. (500 MHz, 293 K, DMSO-d_6).

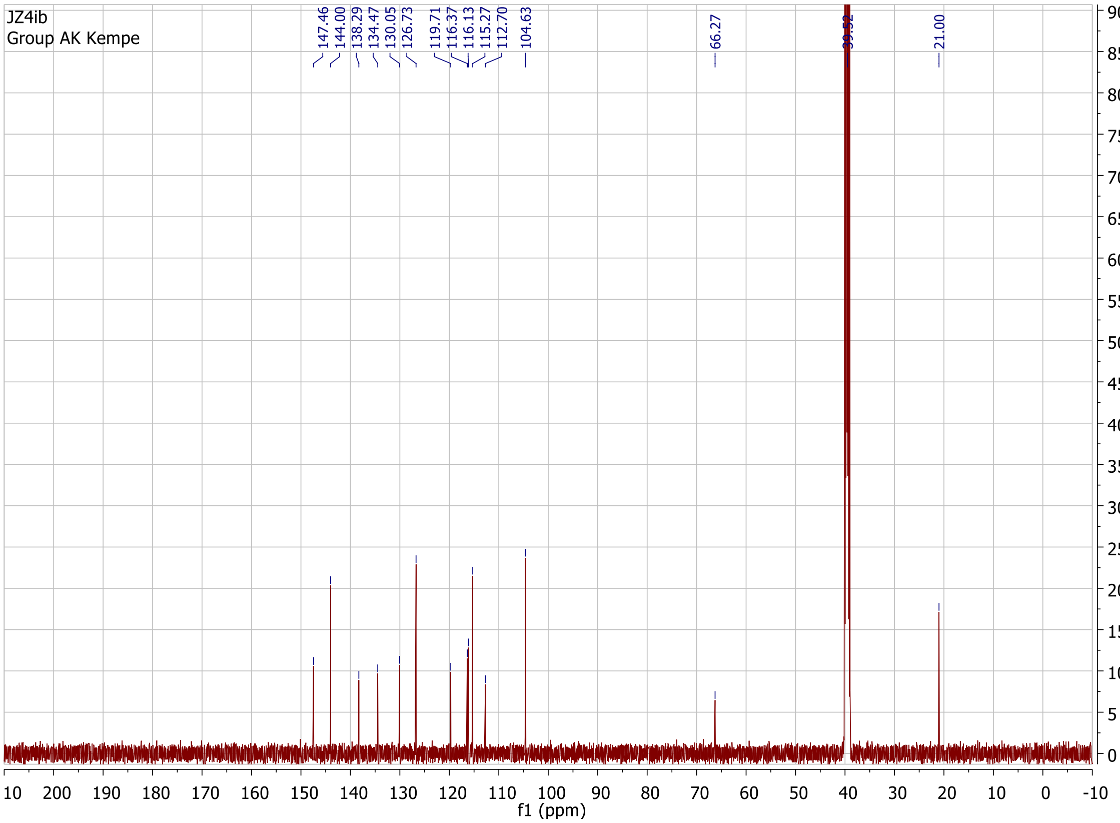

Supplementary Figure 38 ^{13}C NMR spectrum of compound **A4**. (125 MHz, 293 K, DMSO-d_6).

NMR spectra of A5

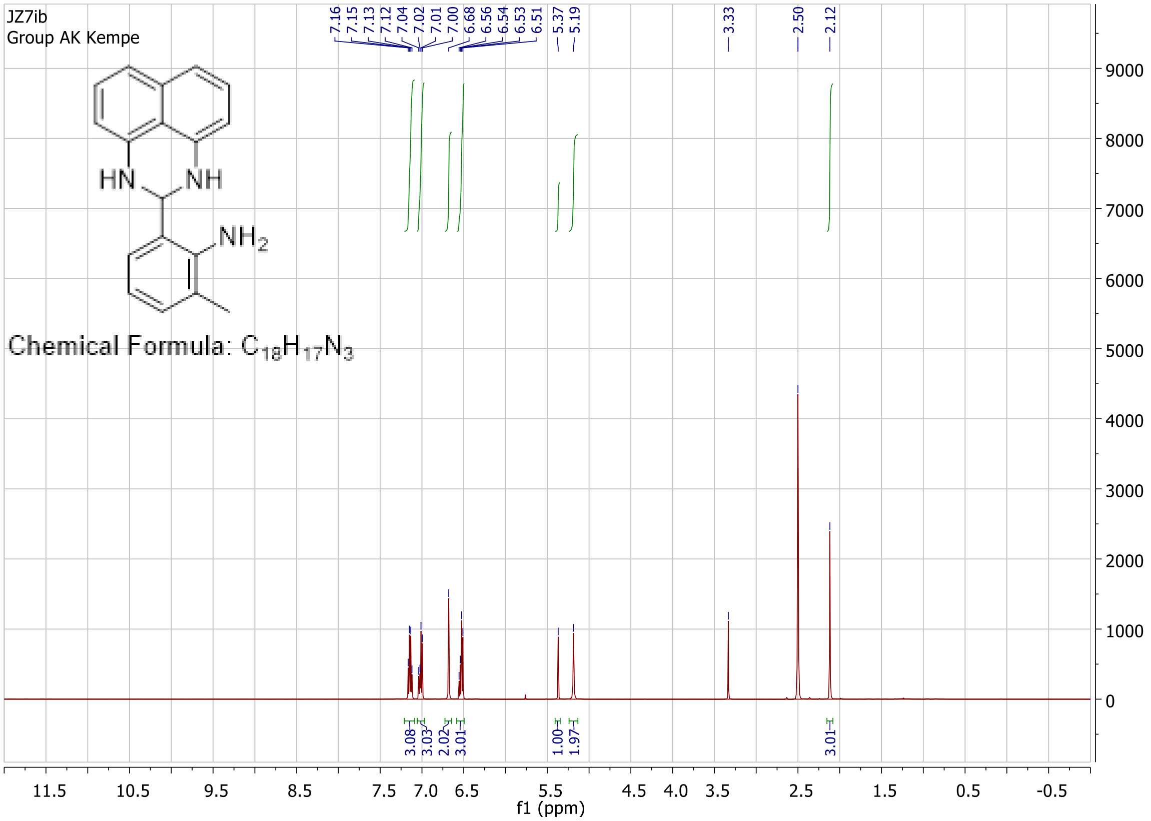

Supplementary Figure 39 ^{1}H NMR spectrum of compound **A5**. (500 MHz, 293 K, DMSO-d$_6$).

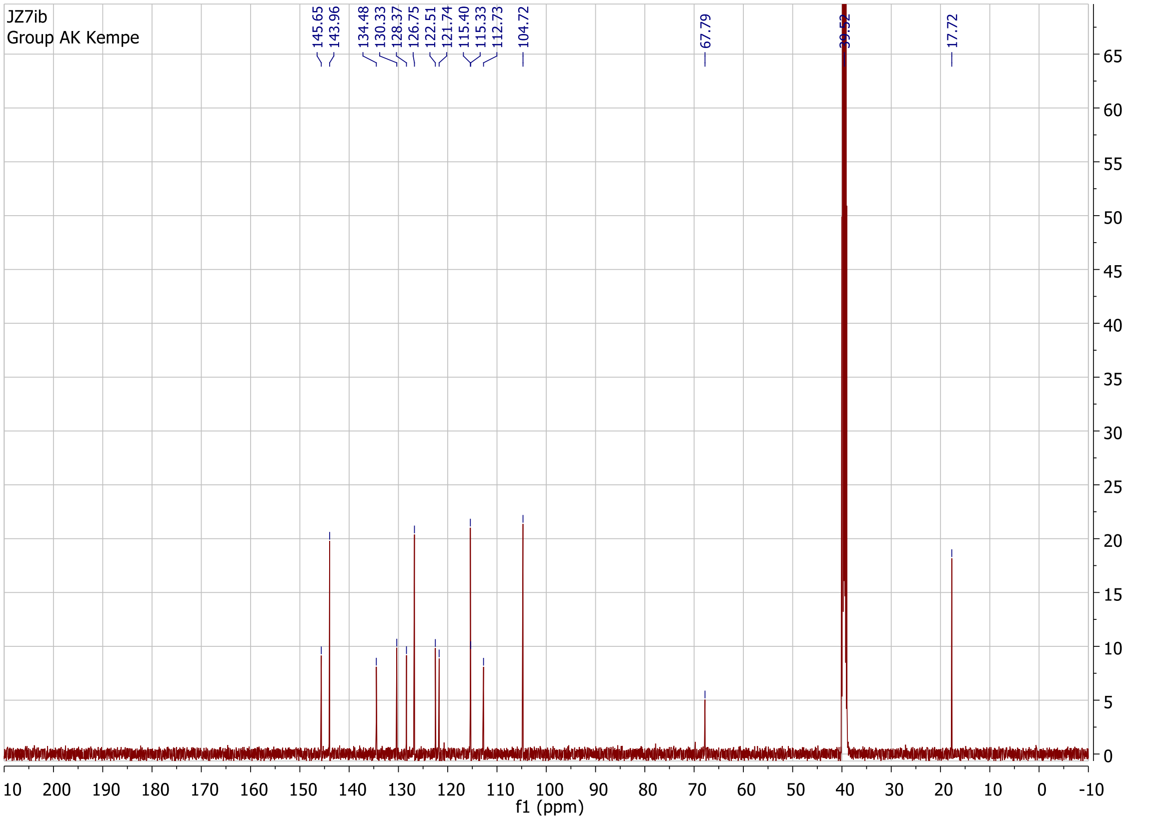

Supplementary Figure 40 ^{13}C NMR spectrum of compound **A5**. (125 MHz, 293 K, DMSO-d$_6$).

NMR spectra of A6

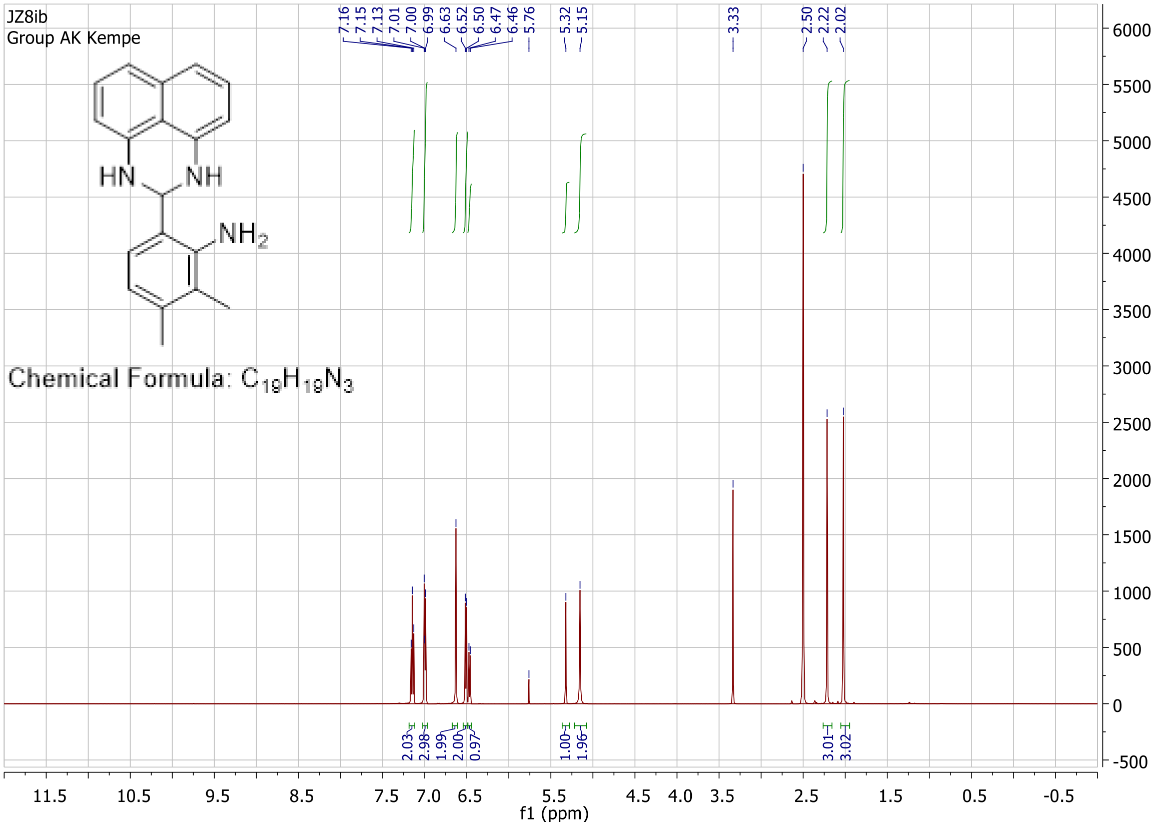

Supplementary Figure 41 ^{1}H NMR spectrum of compound **A6**. (500 MHz, 293 K, DMSO-d_6).

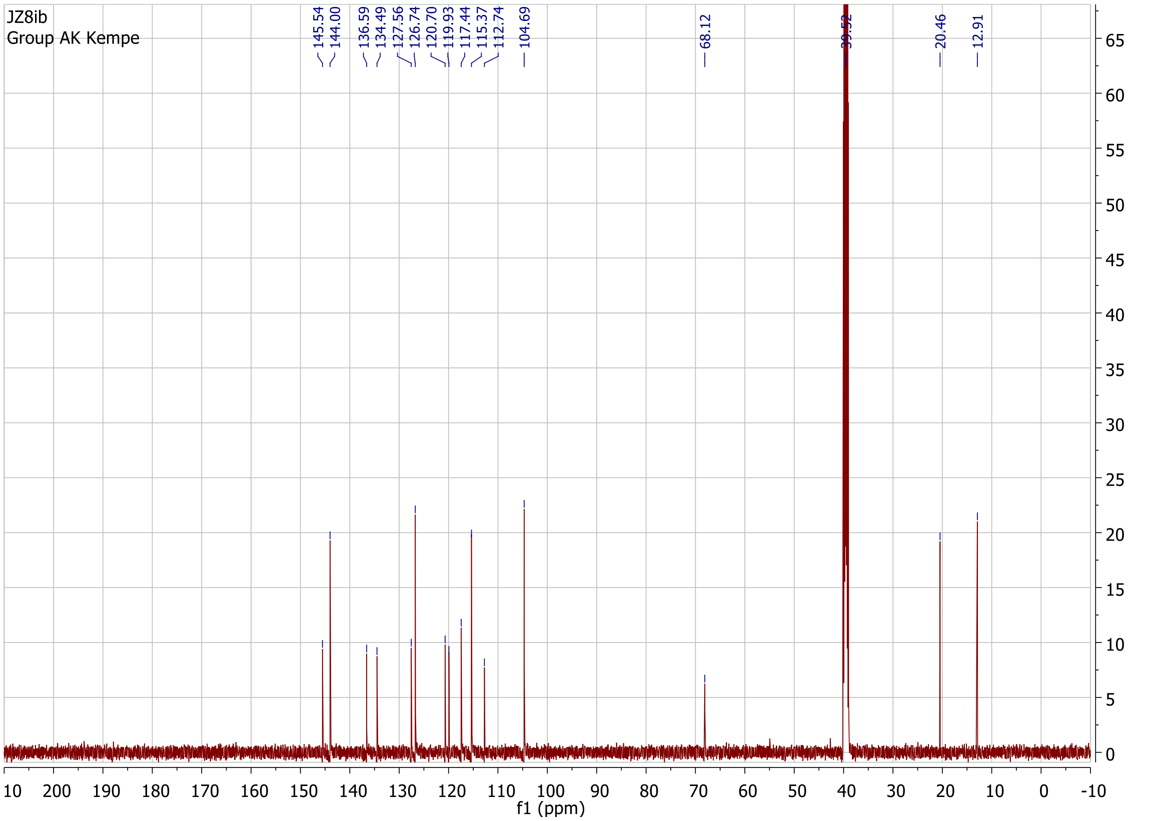

Supplementary Figure 42 ^{13}C NMR spectrum of compound **A6**. (125 MHz, 293 K, DMSO-d_6).

NMR spectra of A7

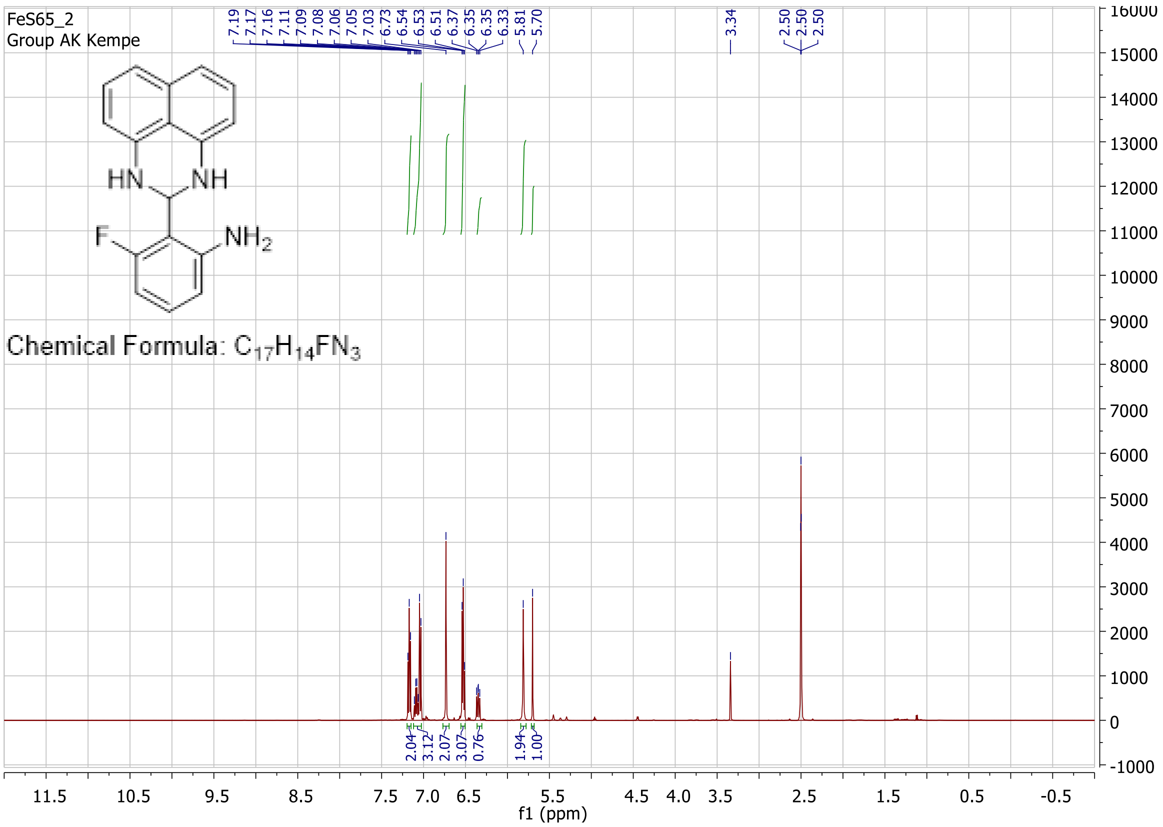

Supplementary Figure 43 ^{1}H NMR spectrum of compound **A7**. (500 MHz, 293 K, DMSO-d_6).

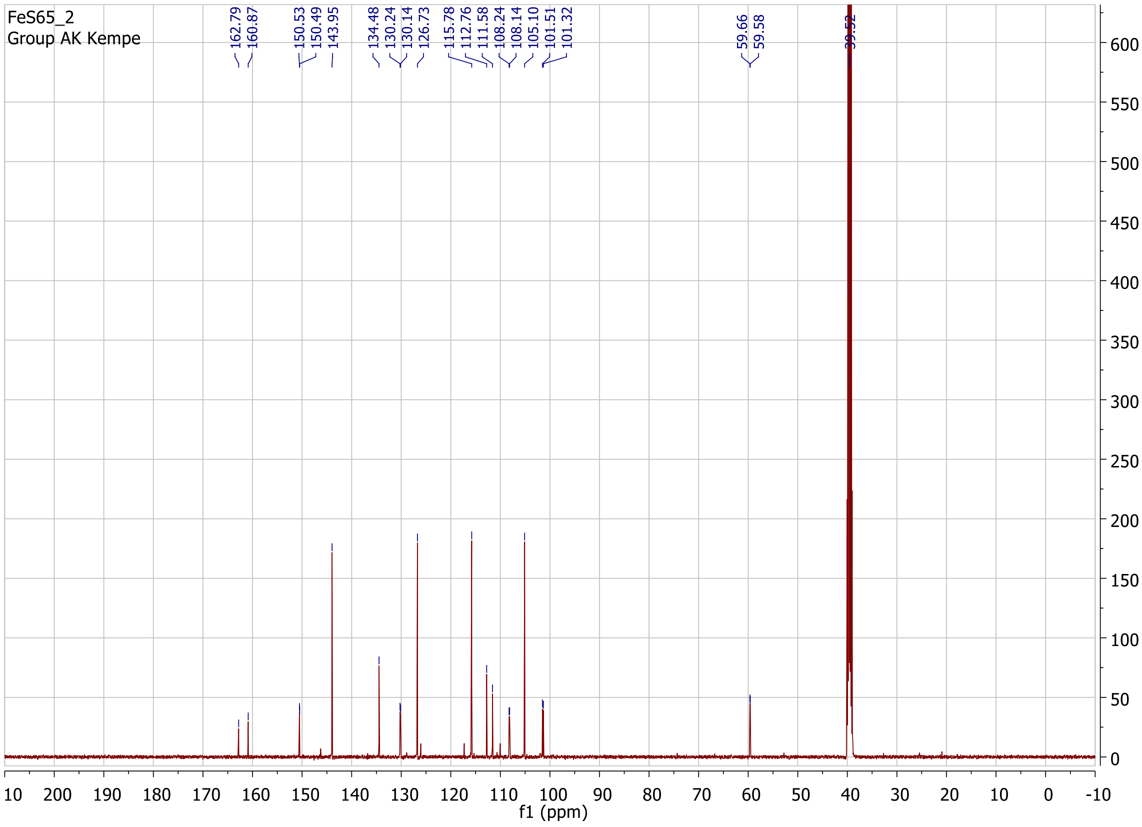

Supplementary Figure 44 ^{13}C NMR spectrum of compound **A7**. (125 MHz, 293 K, DMSO-d_6).

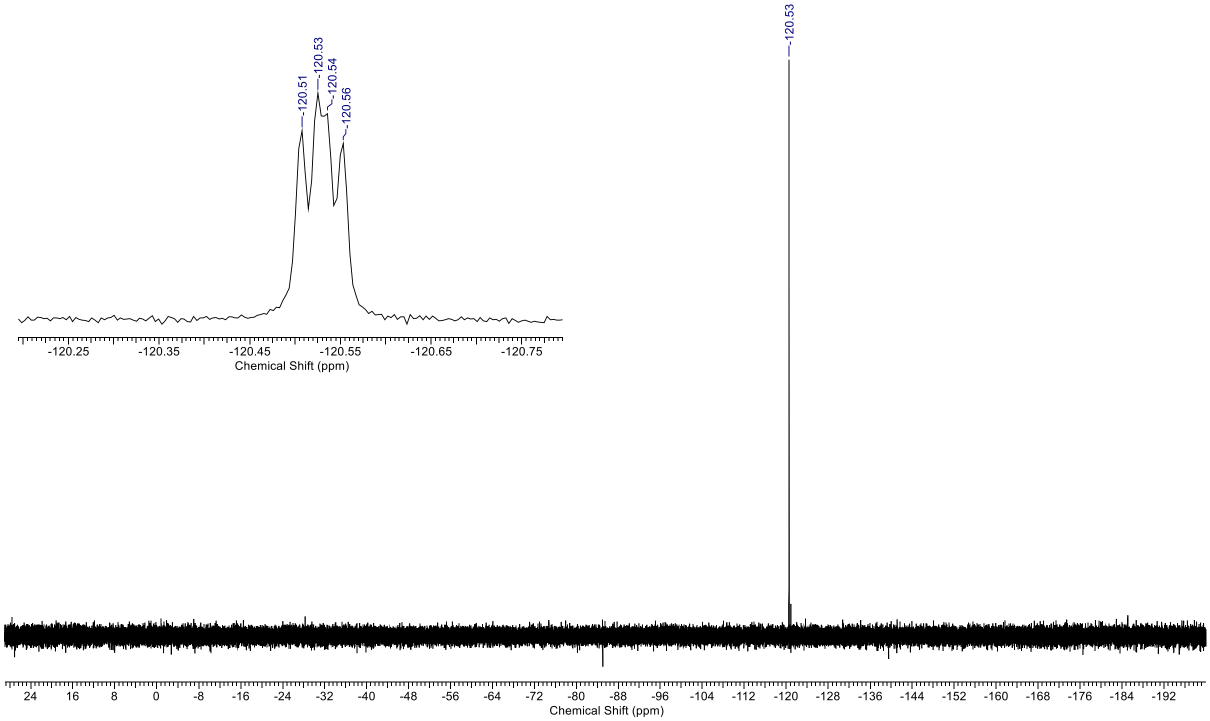

Supplementary Figure 45 ^{19}F NMR spectrum of compound **A7**. (376 MHz, 293 K, DMSO-d_6).

NMR spectra of A8

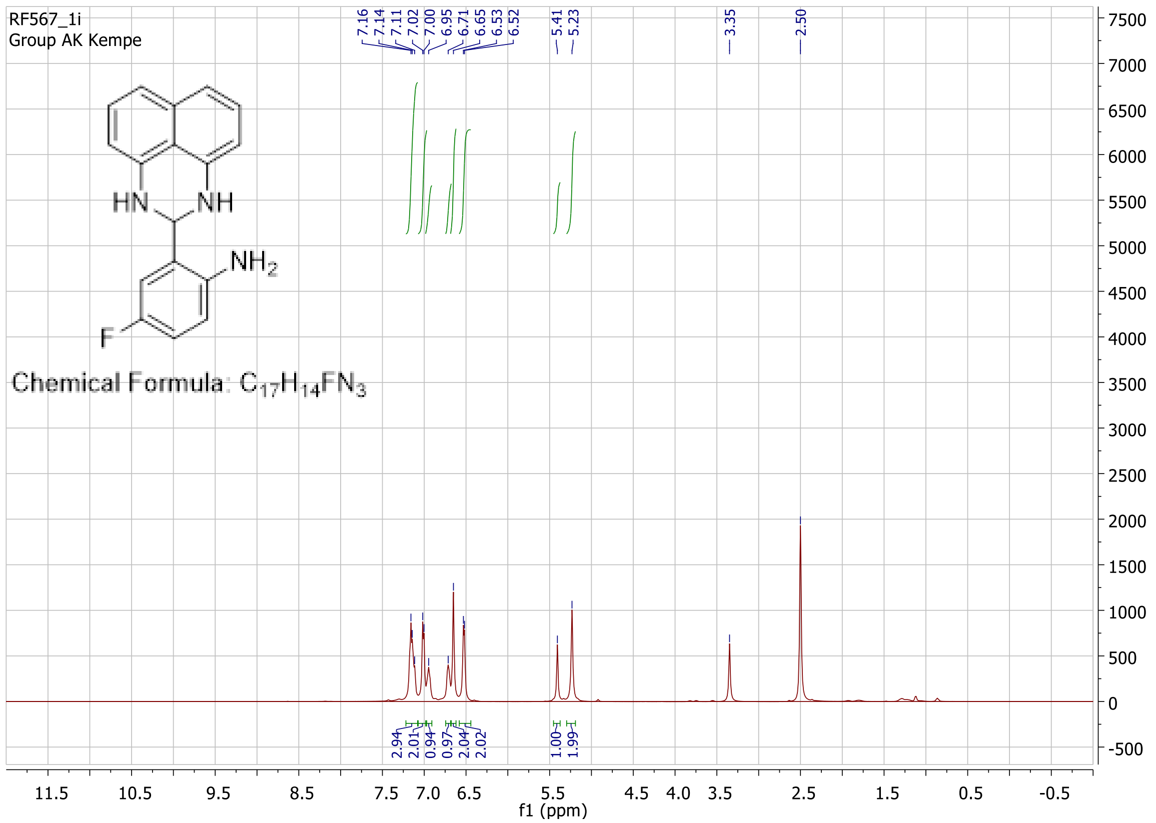

Supplementary Figure 46 ^{1}H NMR spectrum of compound **A8**. (500 MHz, 293 K, DMSO-d_6).

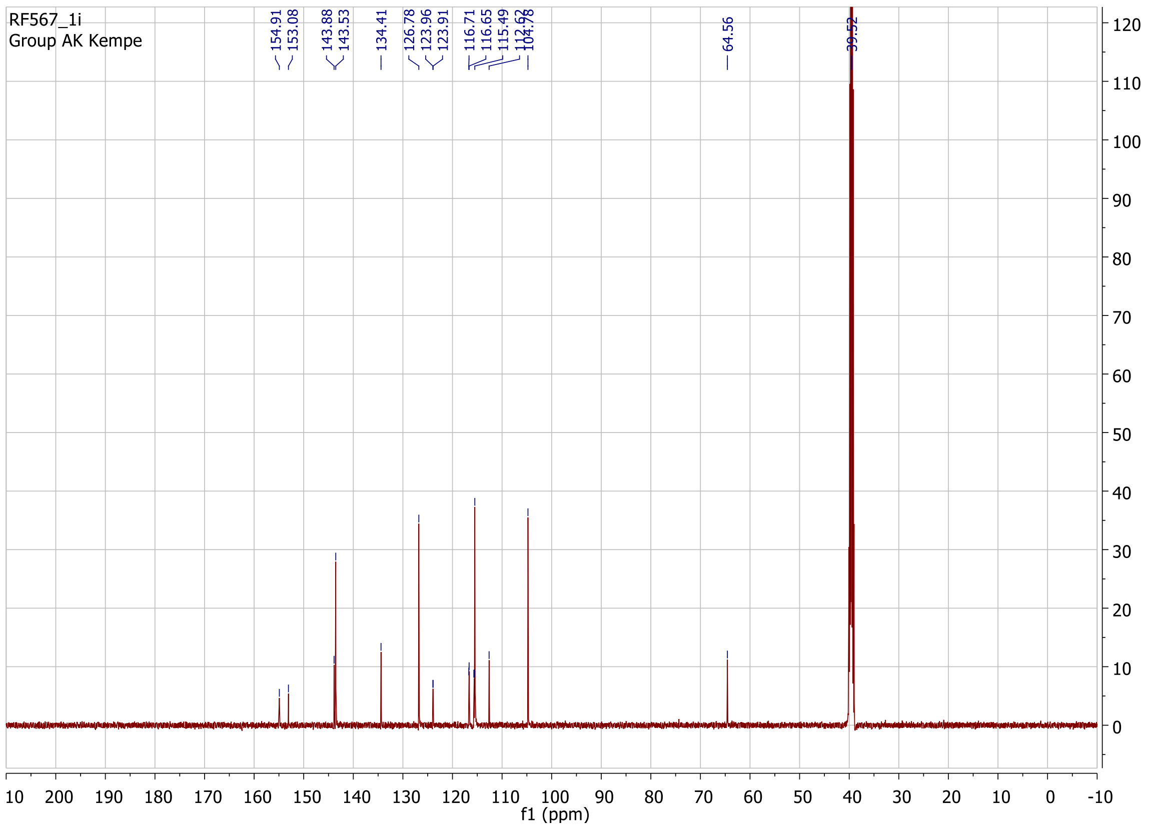

Supplementary Figure 47 ^{13}C NMR spectrum of compound **A8**. (125 MHz, 293 K, DMSO-d_6).

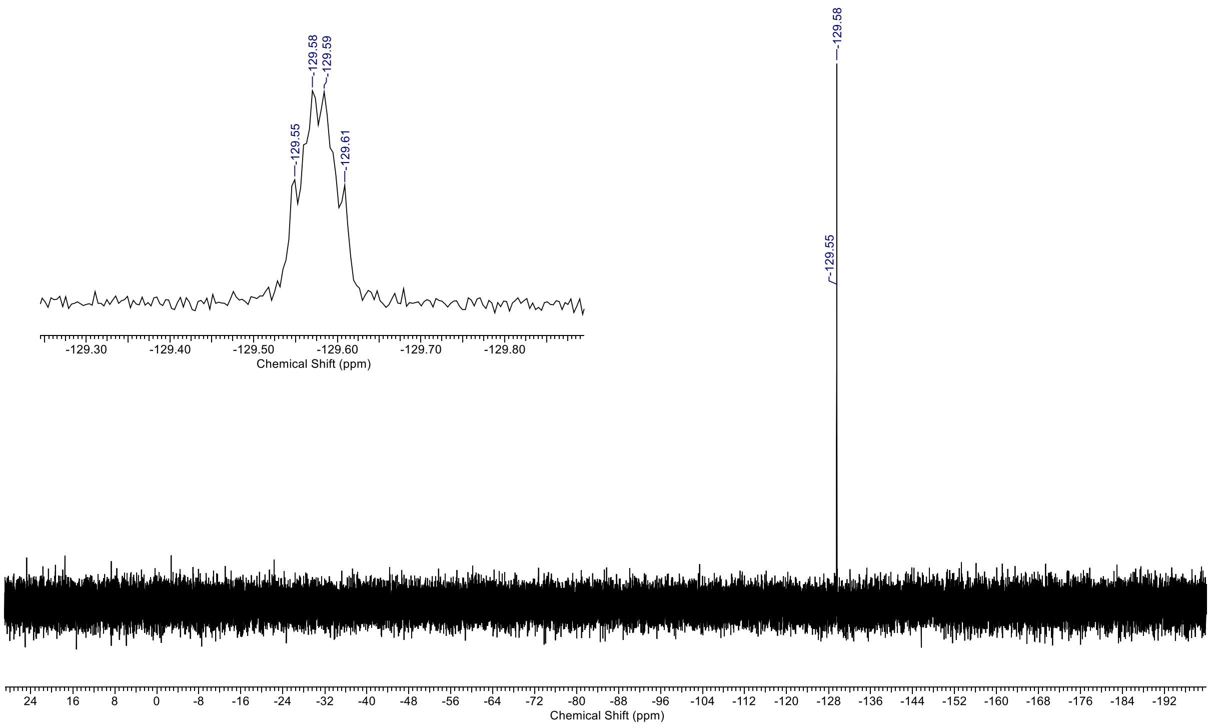

Supplementary Figure 48 ^{19}F NMR spectrum of compound **A8**. (376 MHz, 293 K, DMSO-d$_6$).

NMR spectra of A9

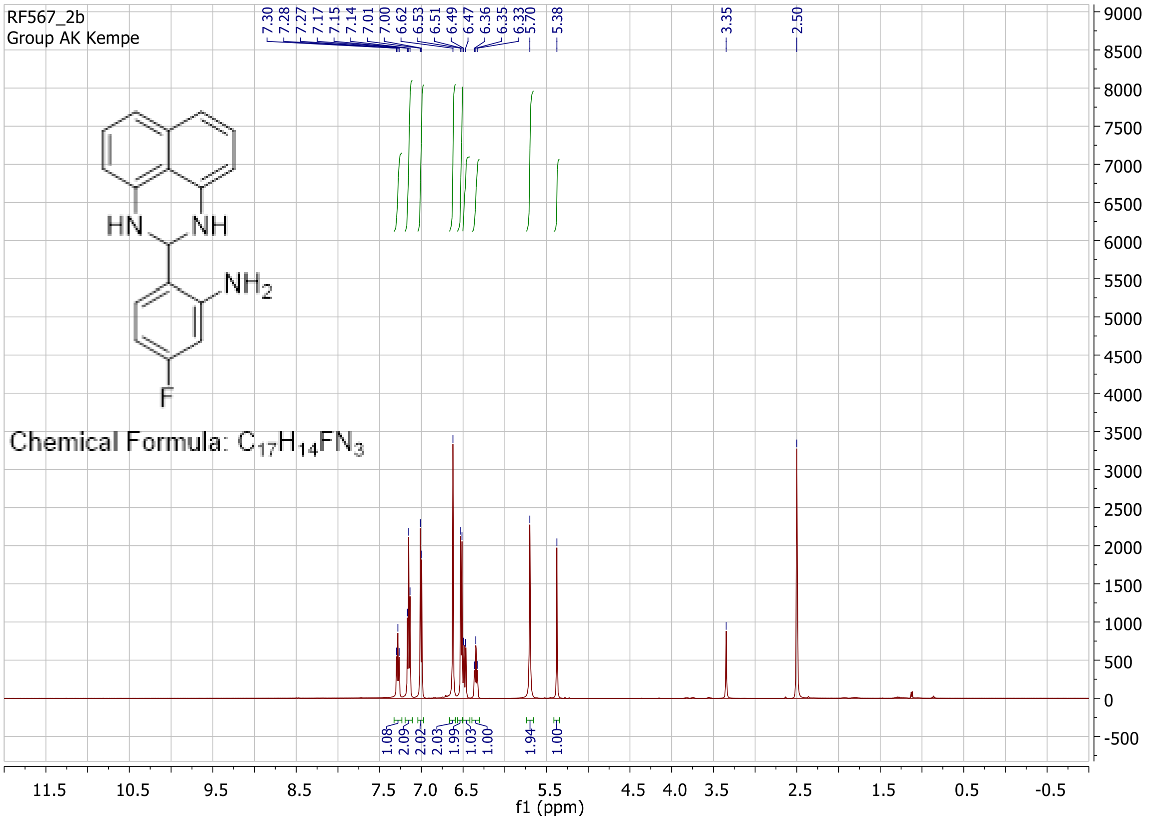

Supplementary Figure 49 ^{1}H NMR spectrum of compound **A9**. (500 MHz, 293 K, DMSO-d_6).

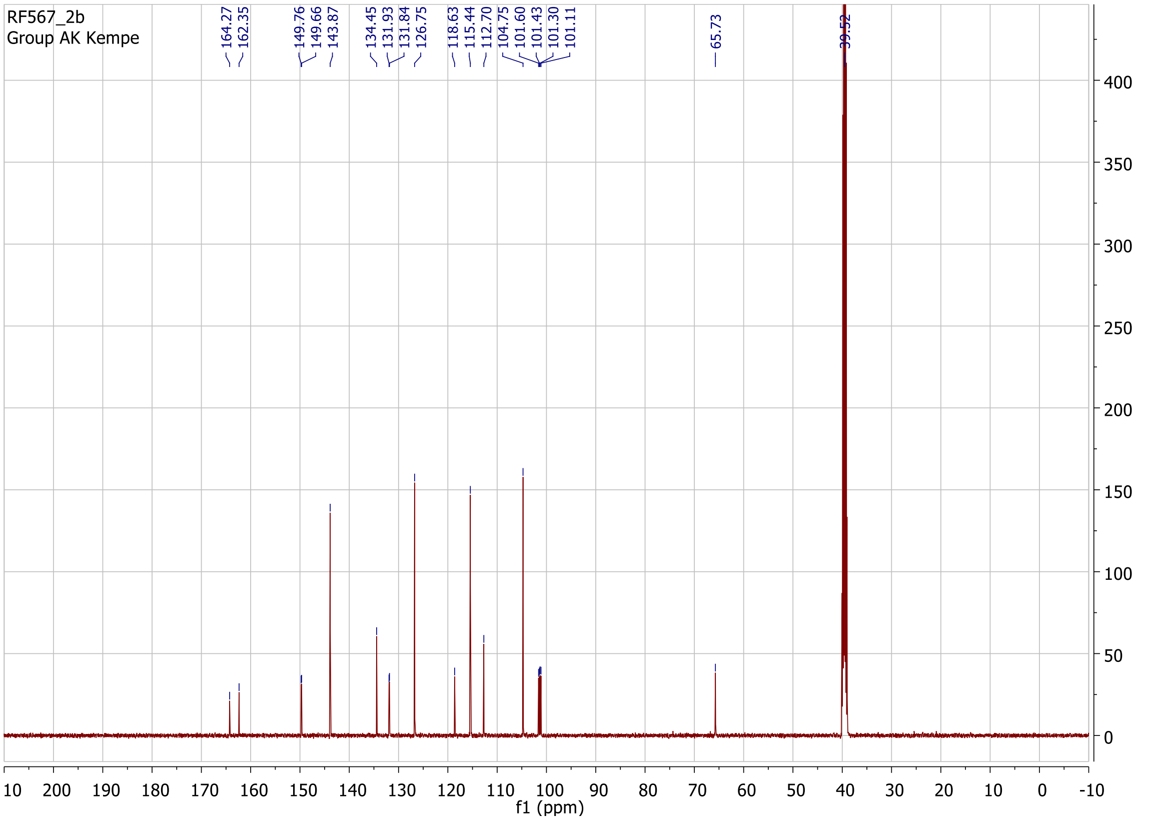

Supplementary Figure 50 ^{13}C NMR spectrum of compound **A9**. (125 MHz, 293 K, DMSO-d_6).

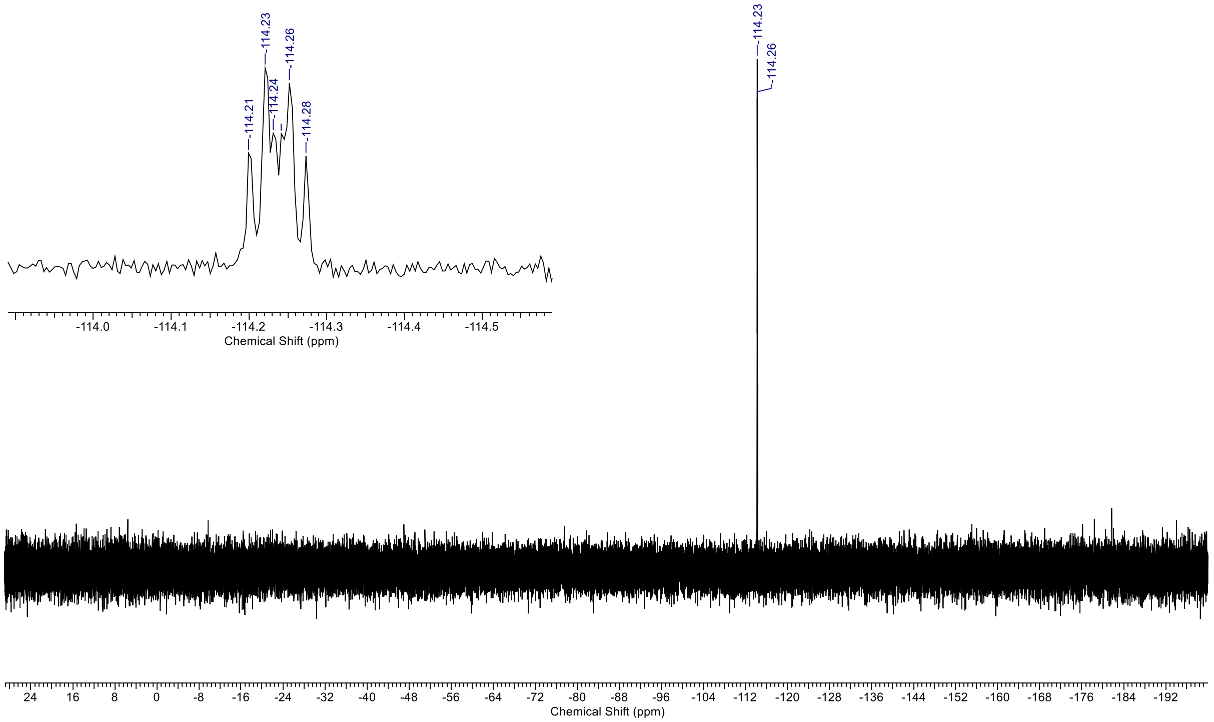

Supplementary Figure 51 ^{19}F NMR spectrum of compound **A9**. (376 MHz, 293 K, DMSO-d_6).

NMR spectra of A10

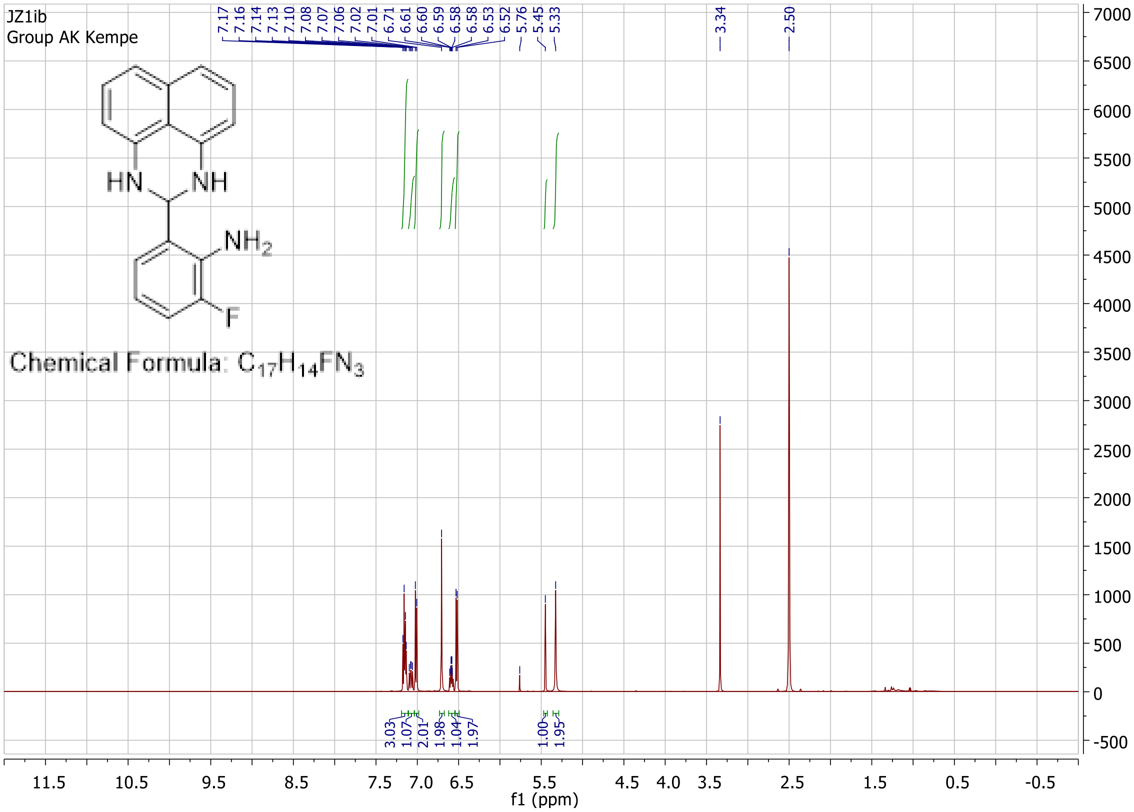

Supplementary Figure 52 ^{1}H NMR spectrum of compound **A10**. (500 MHz, 293 K, DMSO-d_6).

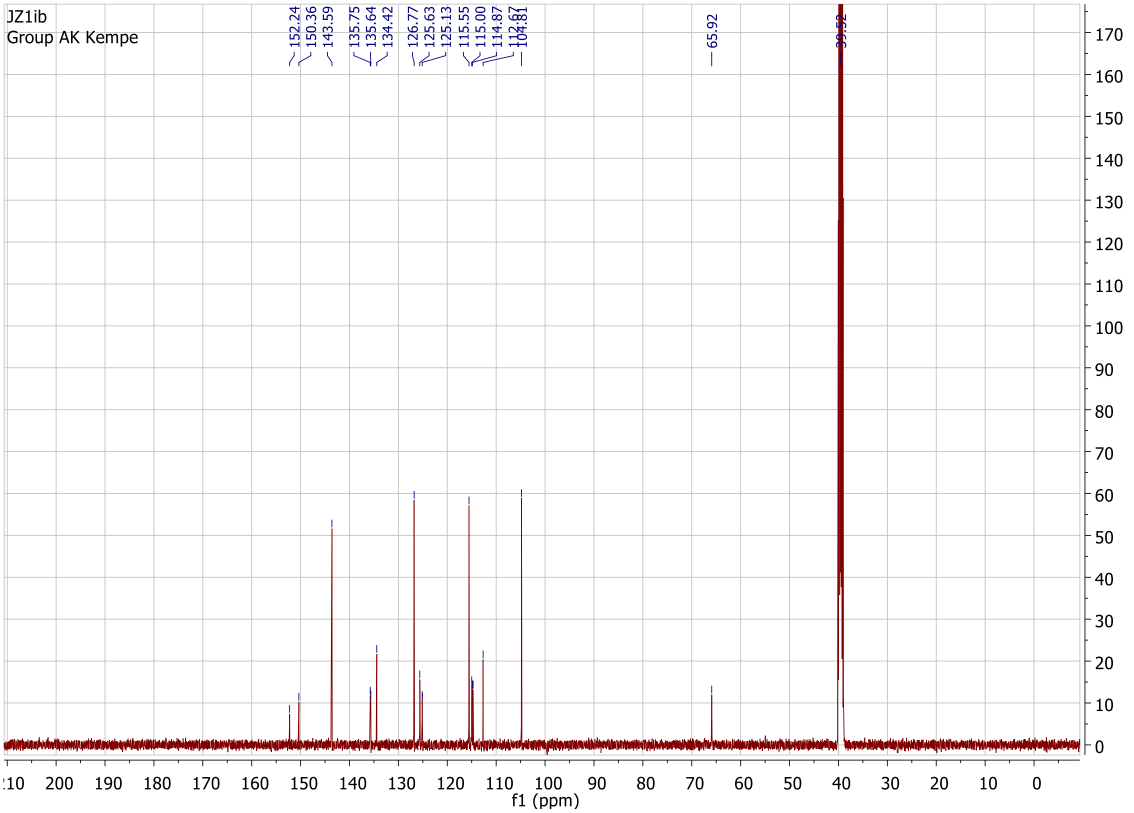

Supplementary Figure 53 ^{13}C NMR spectrum of compound **A10**. (125 MHz, 293 K, DMSO-d_6).

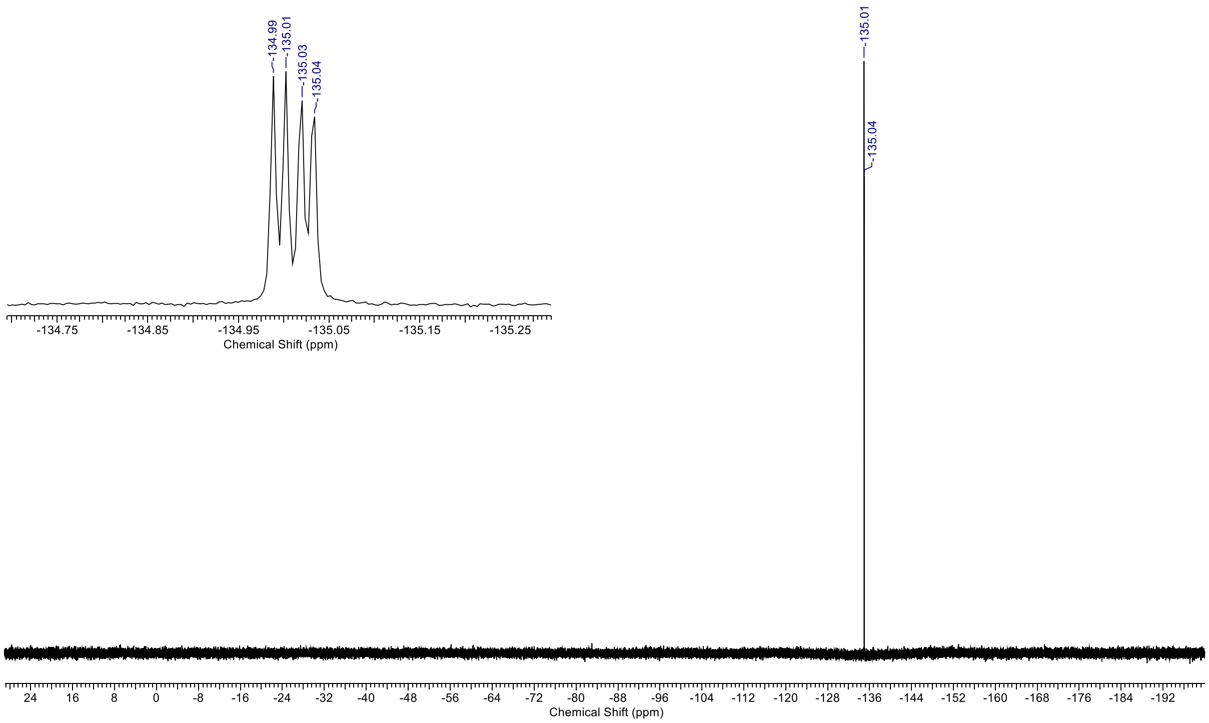

Supplementary Figure 54 ^{19}F NMR spectrum of compound **A10**. (376 MHz, 293 K, DMSO-d_6).

NMR spectra of A11

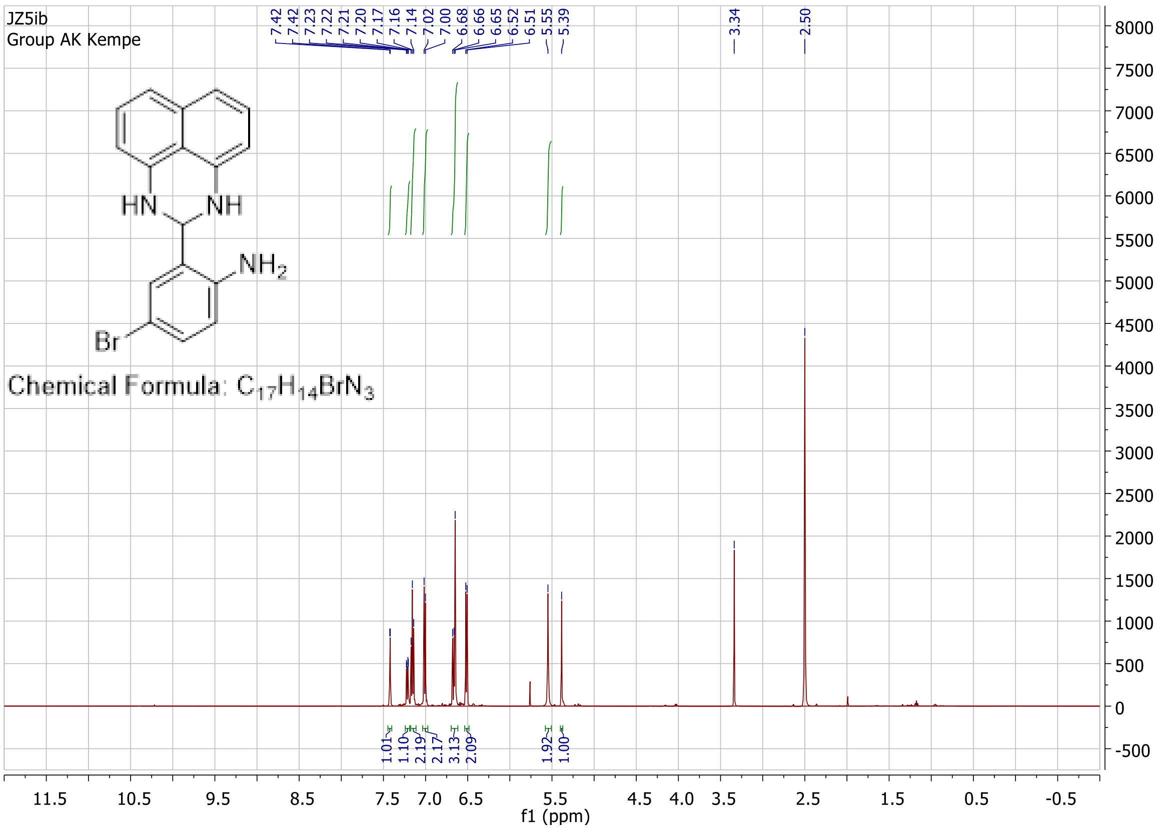

Supplementary Figure 55 ^{1}H NMR spectrum of compound **A11**. (500 MHz, 293 K, DMSO-d_6).

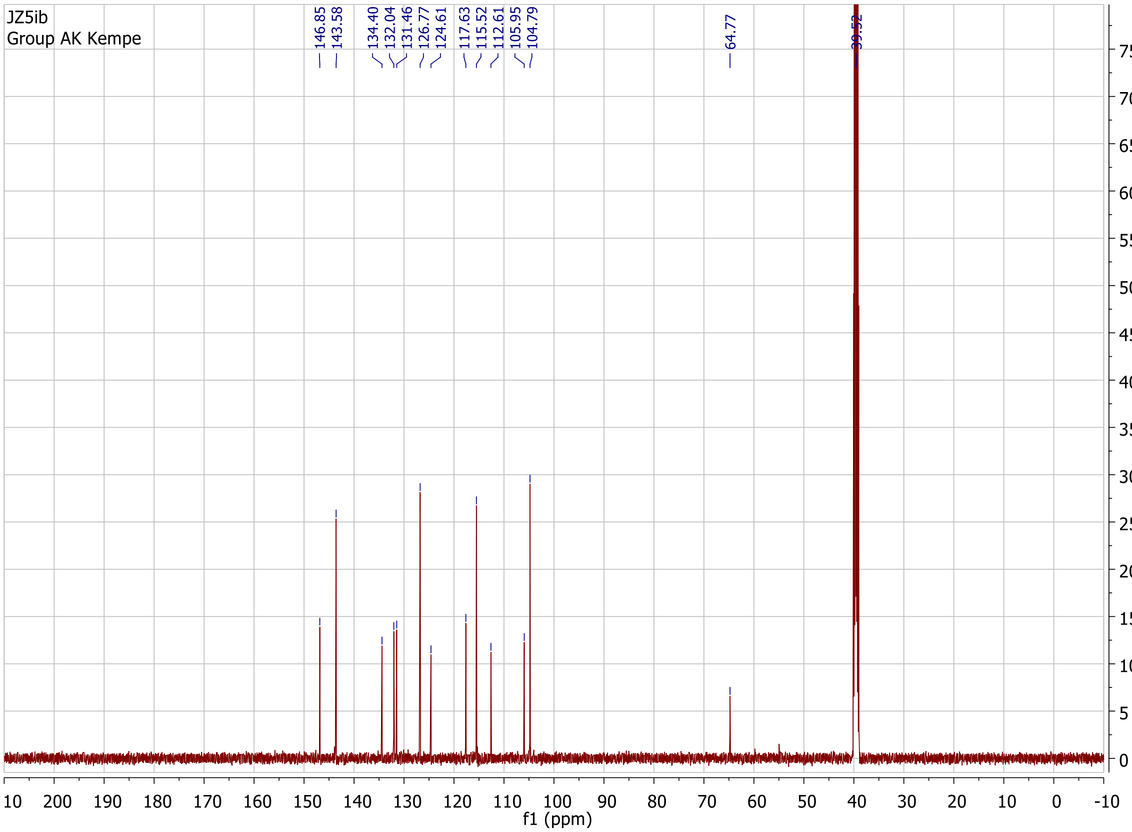

Supplementary Figure 56 ^{13}C NMR spectrum of compound **A11**. (125 MHz, 293 K, DMSO-d_6).

NMR spectra of A12

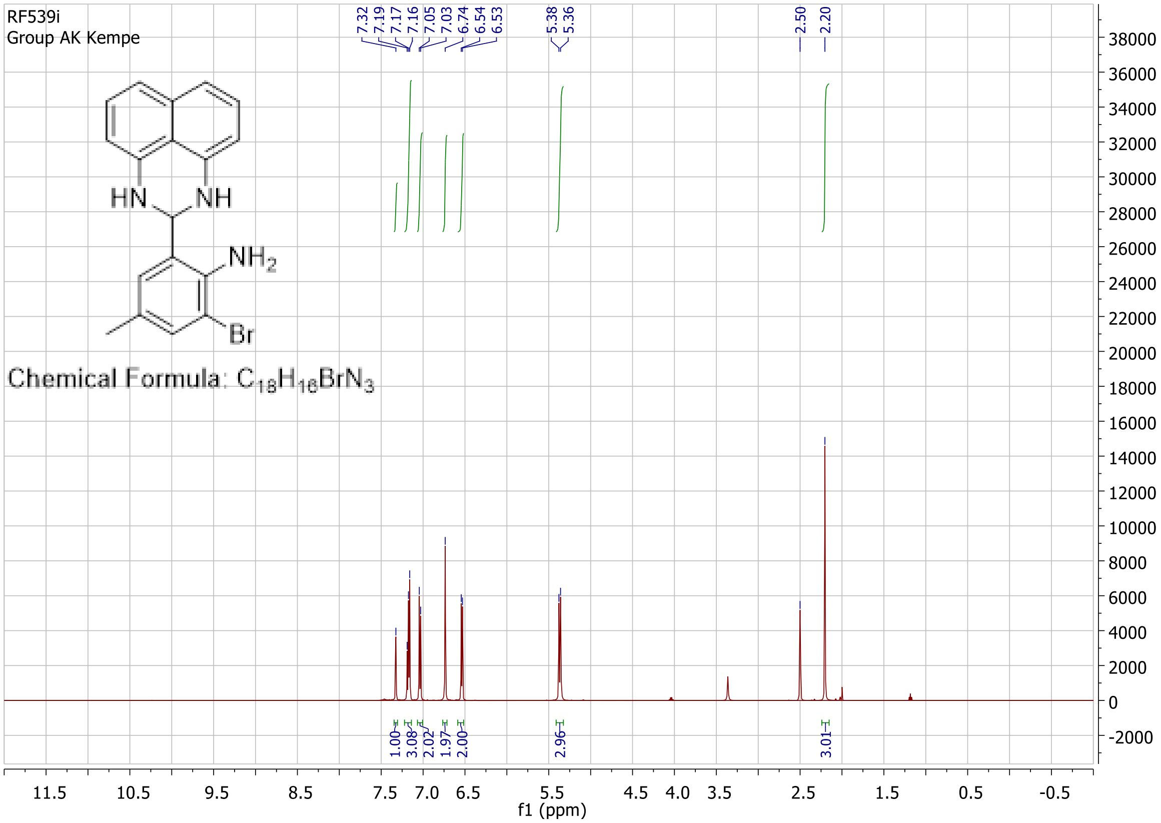

Supplementary Figure 57 ^{1}H NMR spectrum of compound **A12**. (500 MHz, 293 K, DMSO-d$_6$).

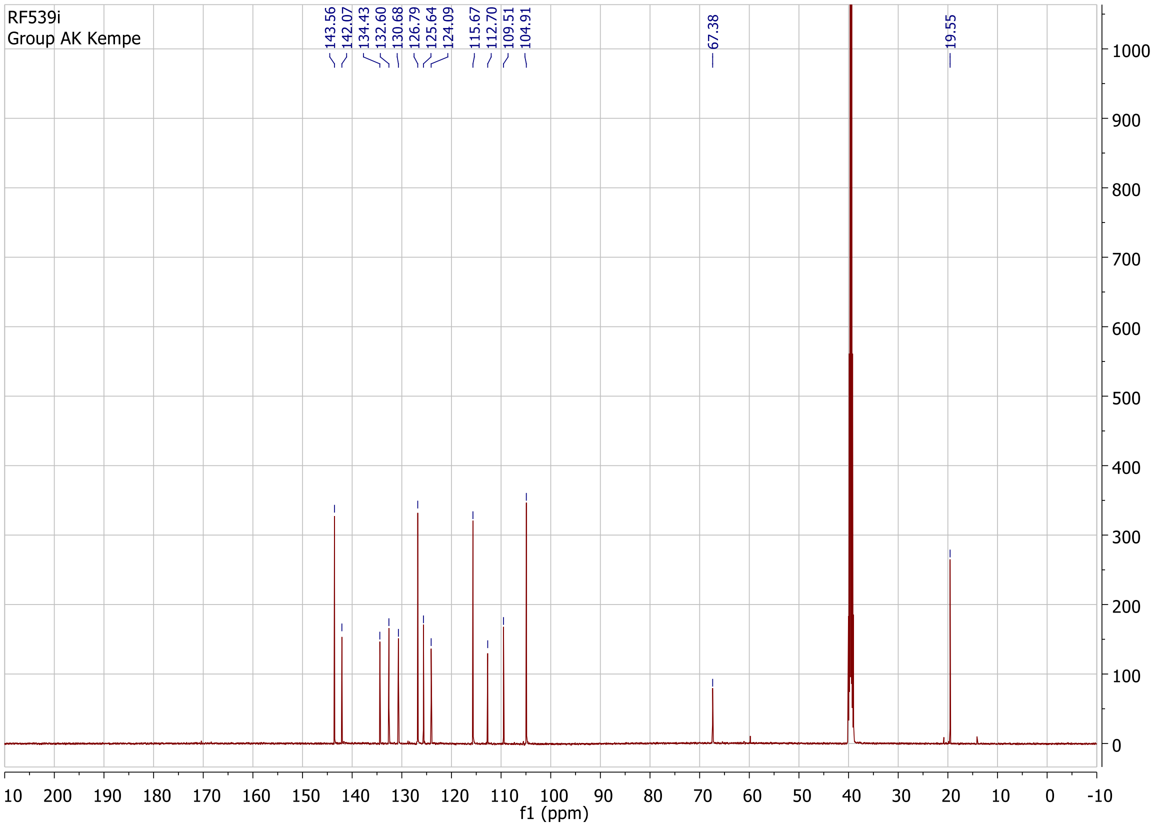

Supplementary Figure 58 ^{13}C NMR spectrum of compound **A12**. (125 MHz, 293 K, DMSO-d$_6$).

NMR spectra of A13

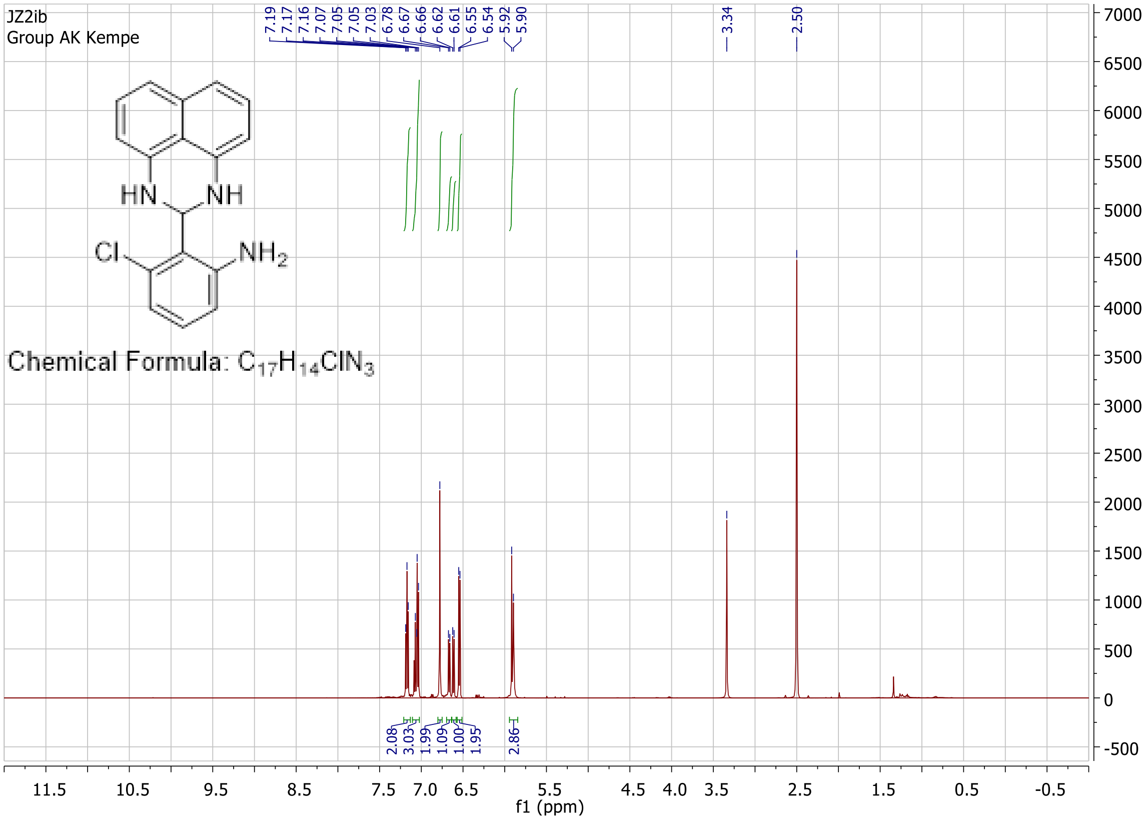

Supplementary Figure 59 ^{1}H NMR spectrum of compound **A13**. (500 MHz, 293 K, DMSO-d$_6$).

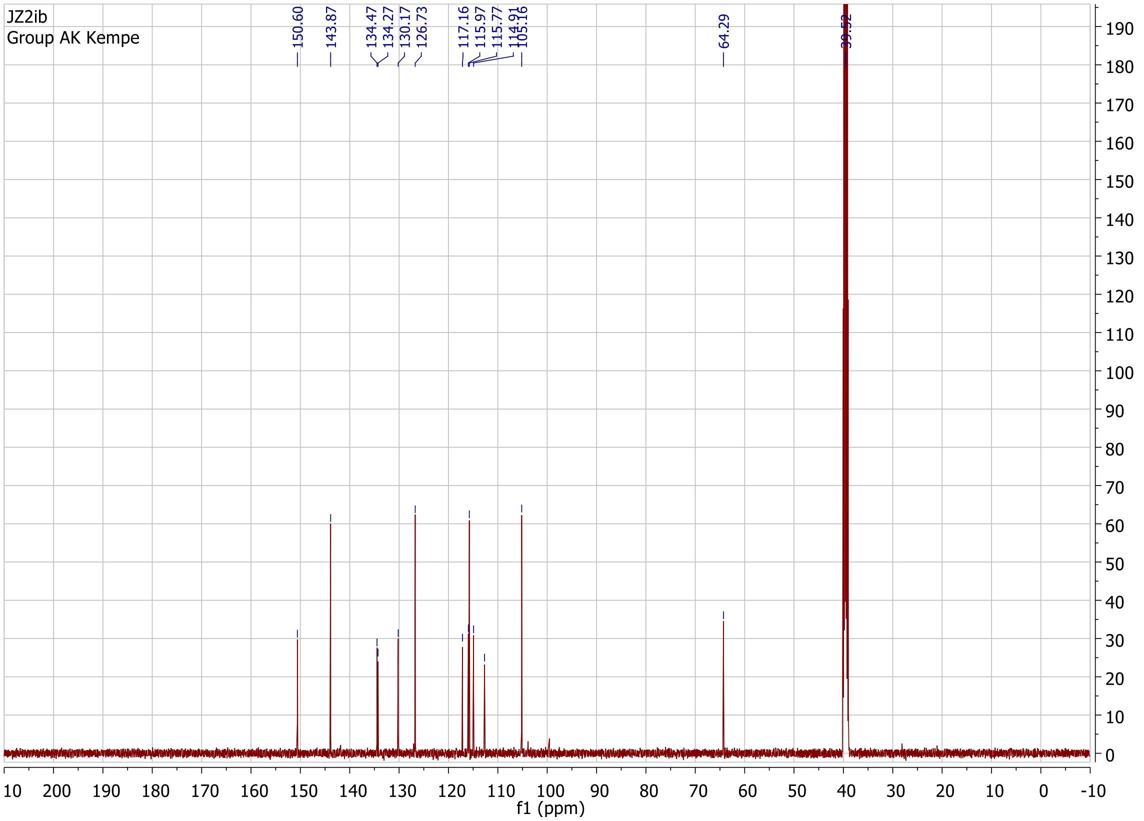

Supplementary Figure 60 ^{13}C NMR spectrum of compound **A13**. (125 MHz, 293 K, DMSO-d$_6$).

NMR spectra of A14

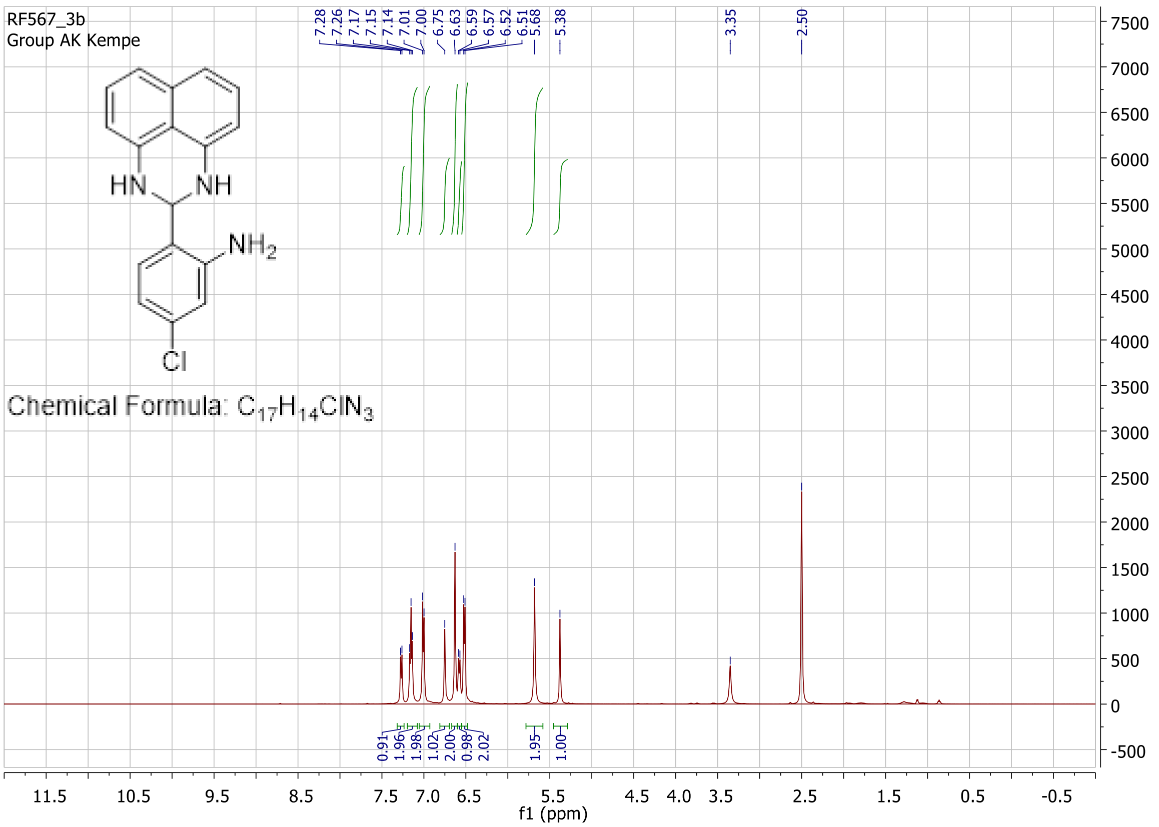

Supplementary Figure 61 ^{1}H NMR spectrum of compound **A14**. (500 MHz, 293 K, DMSO-d_6).

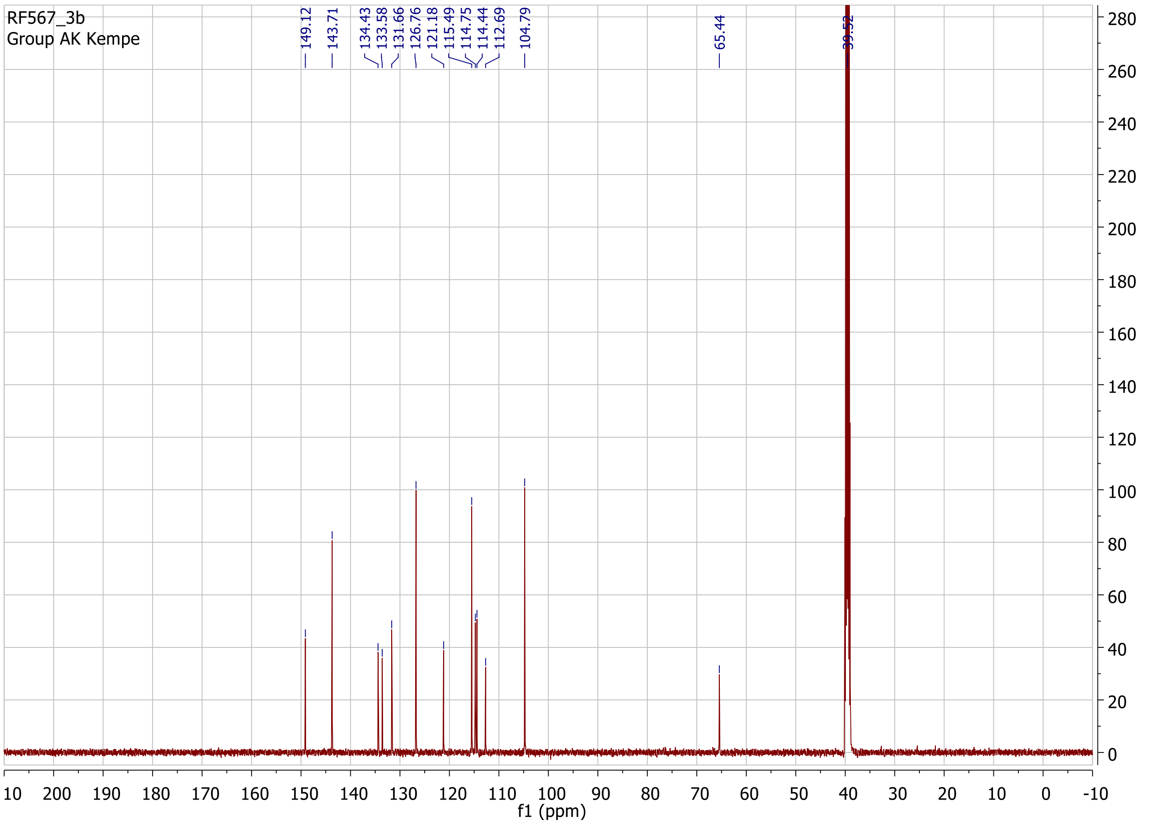

Supplementary Figure 62 ^{13}C NMR spectrum of compound **A14**. (125 MHz, 293 K, DMSO-d_6).

NMR spectra of A15

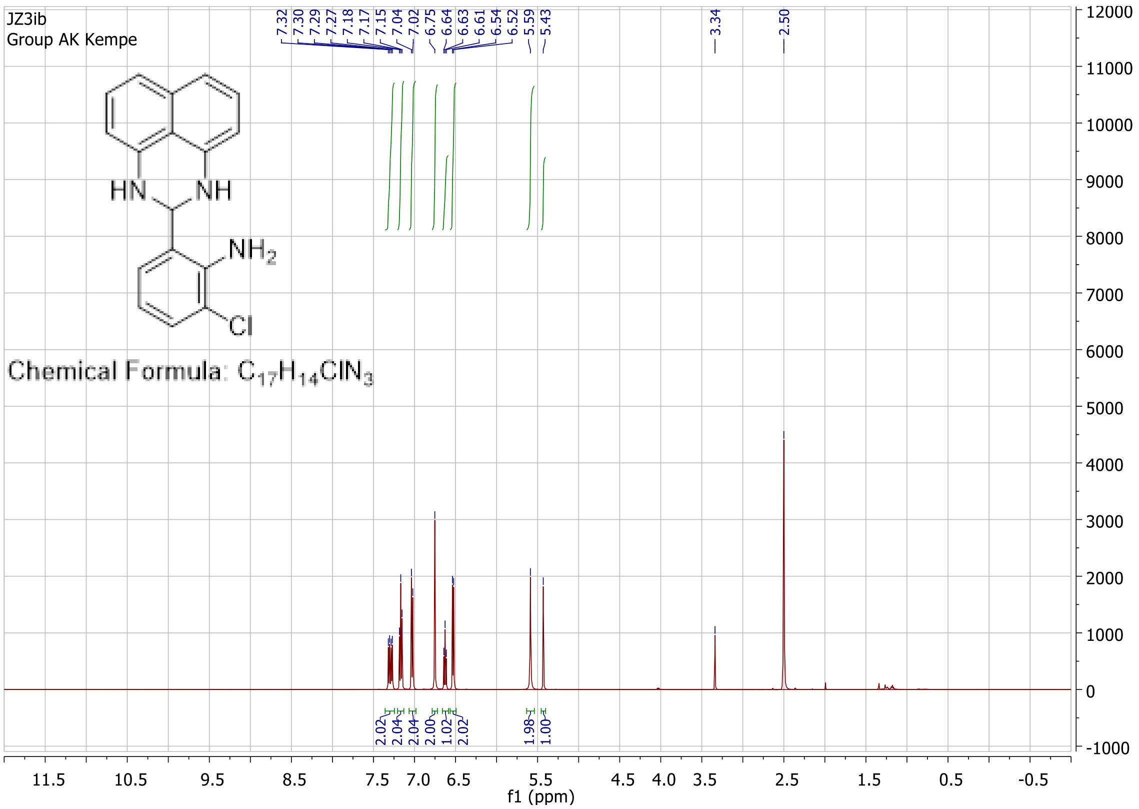

Supplementary Figure 63 ^{1}H NMR spectrum of compound **A15**. (500 MHz, 293 K, DMSO-d_6).

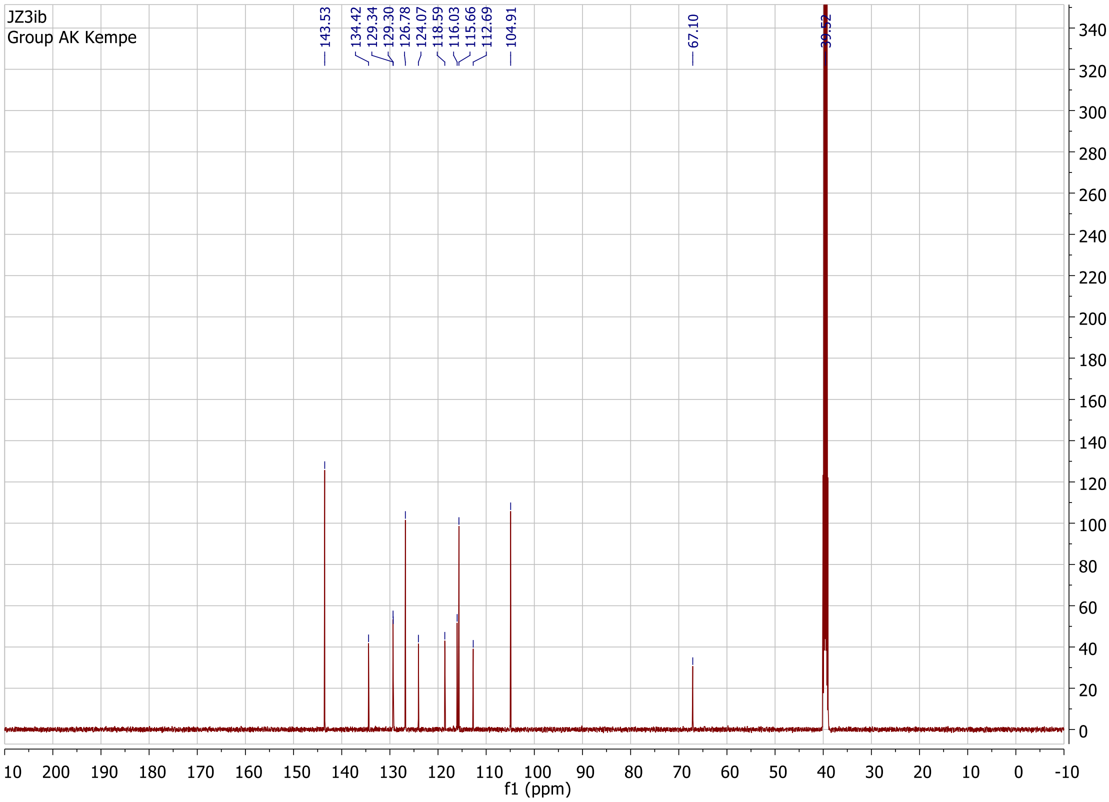

Supplementary Figure 64 ^{13}C NMR spectrum of compound **A15**. (125 MHz, 293 K, DMSO-d_6).

NMR spectra of A16

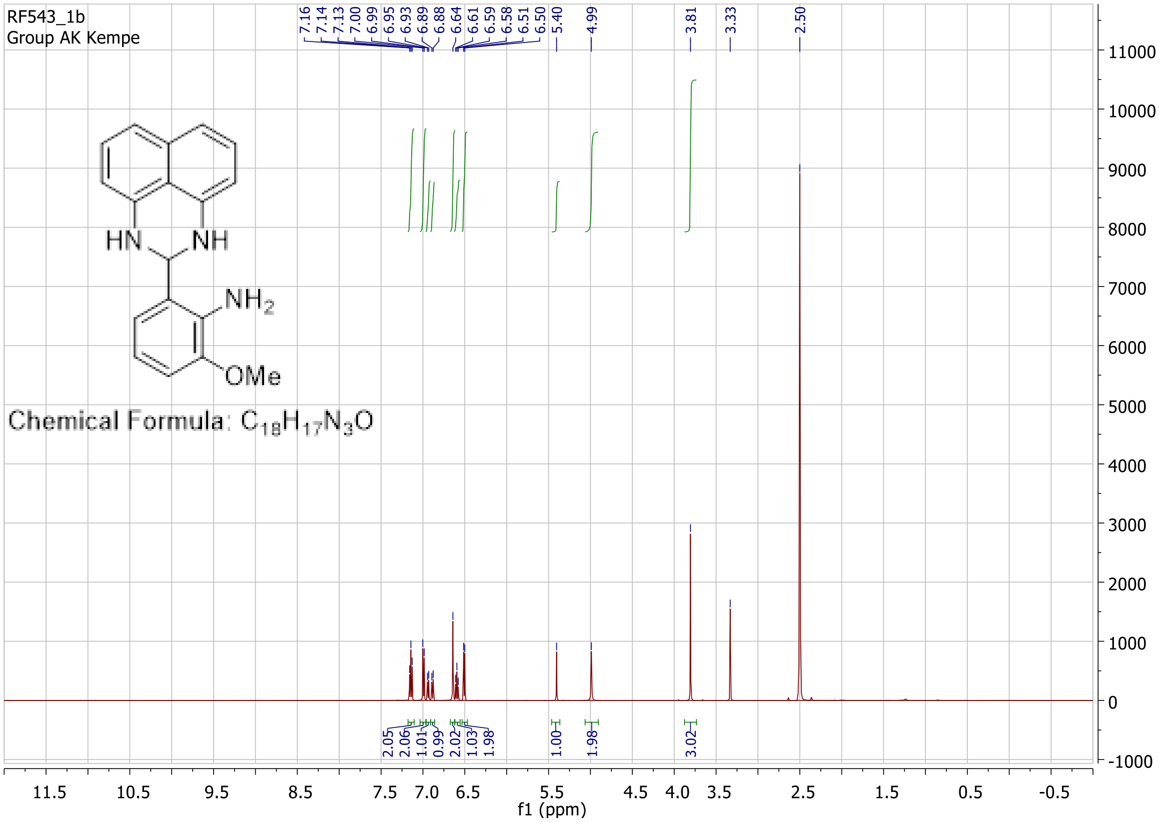

Supplementary Figure 65 ^{1}H NMR spectrum of compound **A16**. (500 MHz, 293 K, DMSO-d_6).

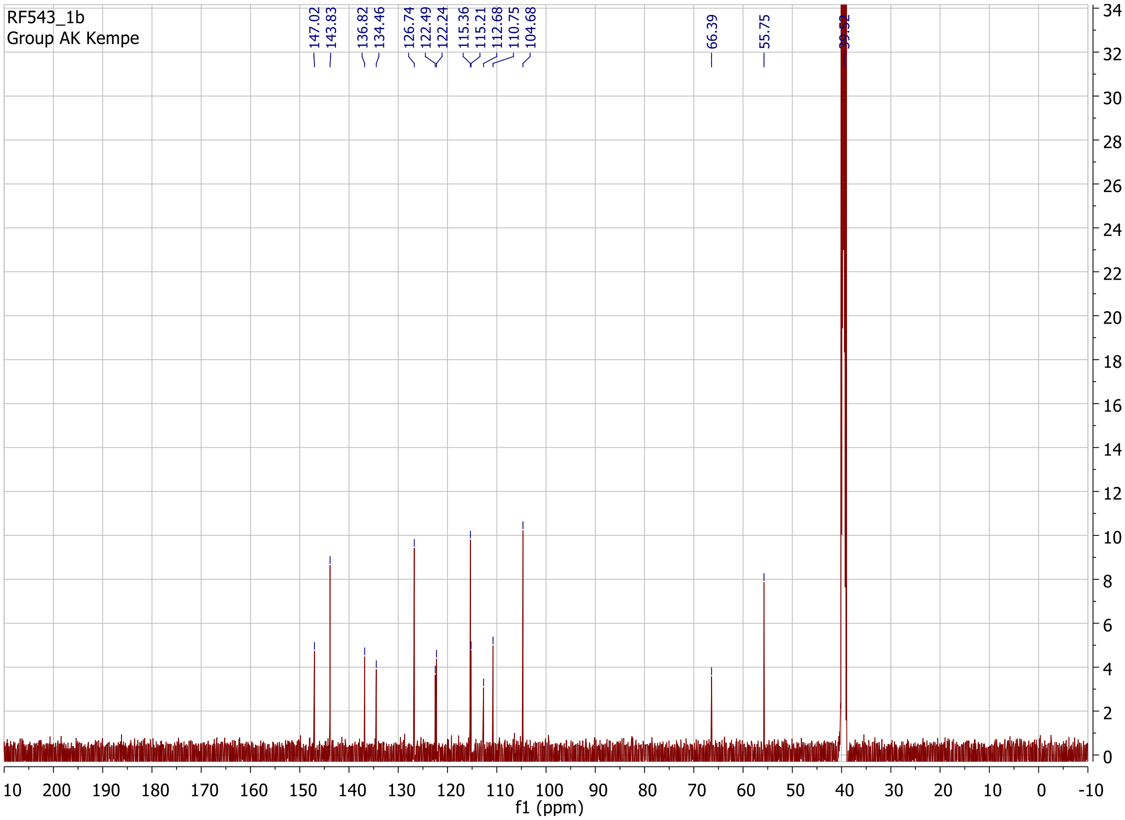

Supplementary Figure 66 ^{13}C NMR spectrum of compound **A16**. (125 MHz, 293 K, DMSO-d_6).

NMR spectra of A17

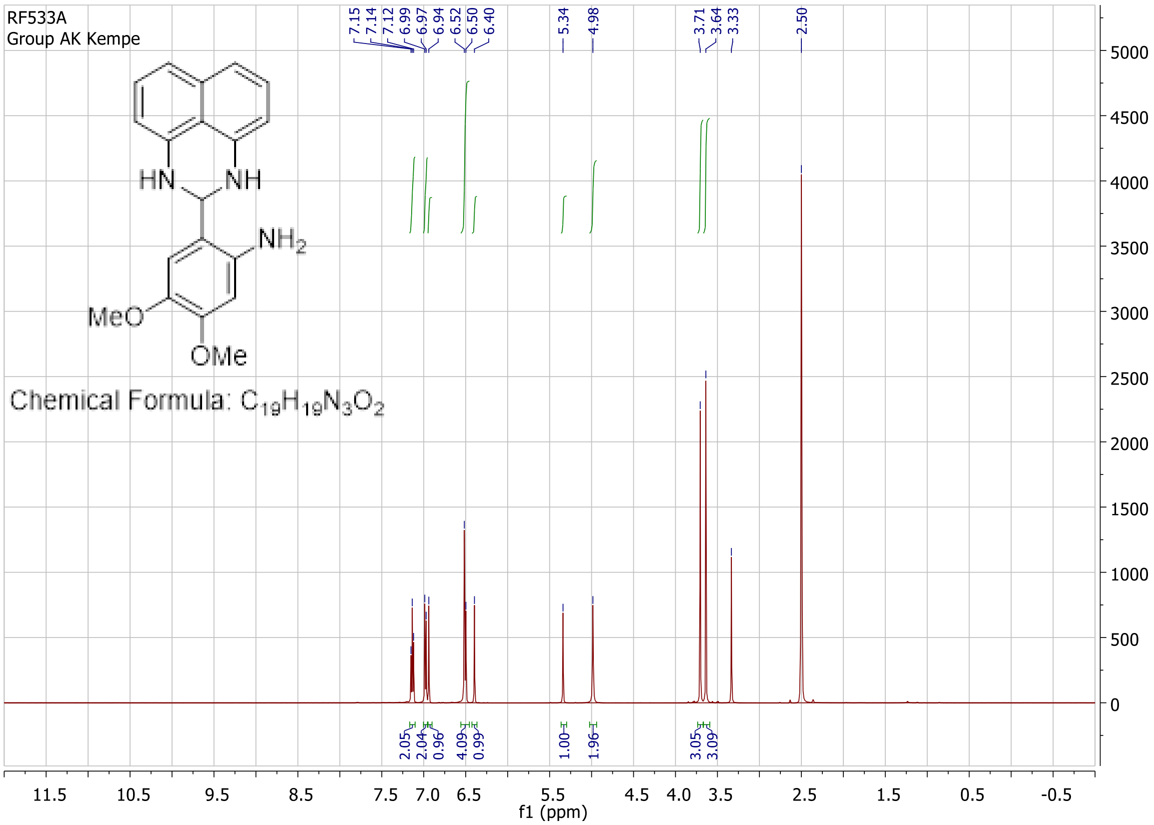

Supplementary Figure 67 ^{1}H NMR spectrum of compound **A17**. (500 MHz, 293 K, DMSO-d_6).

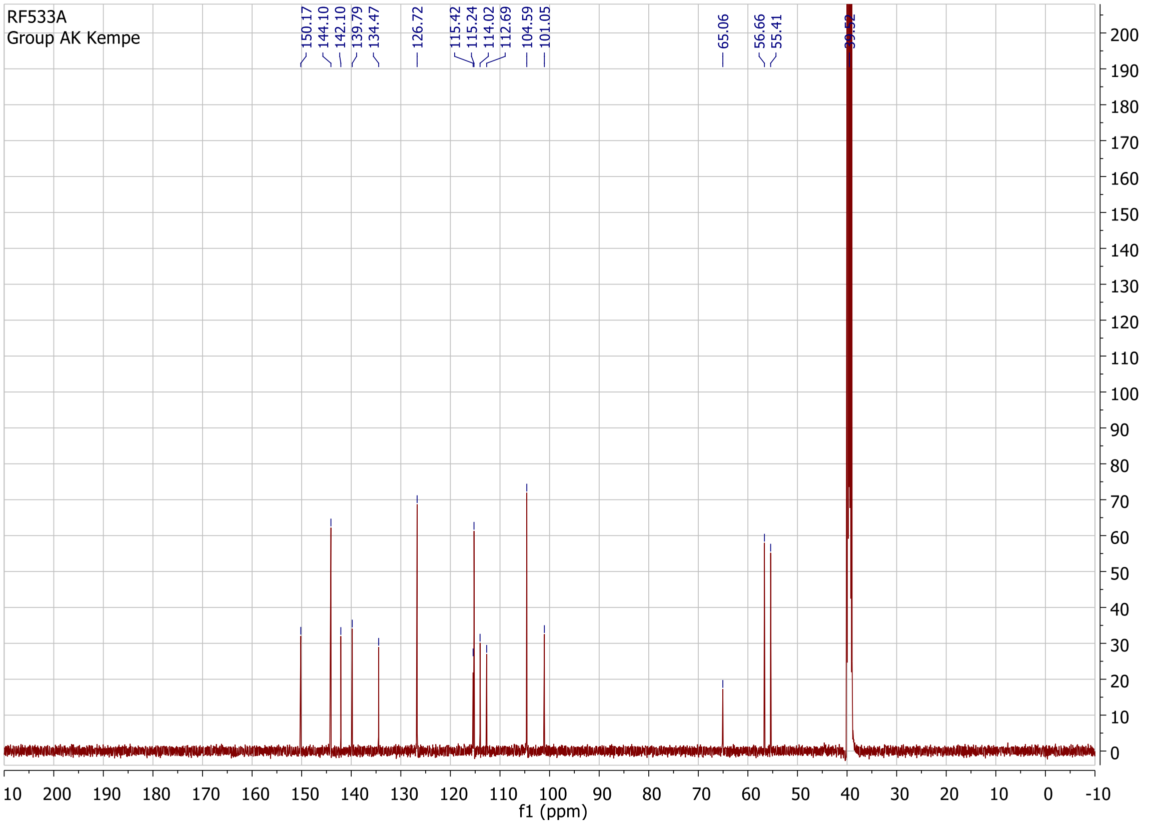

Supplementary Figure 68 ^{13}C NMR spectrum of compound **A17**. (125 MHz, 293 K, DMSO-d_6).

NMR spectra of A18

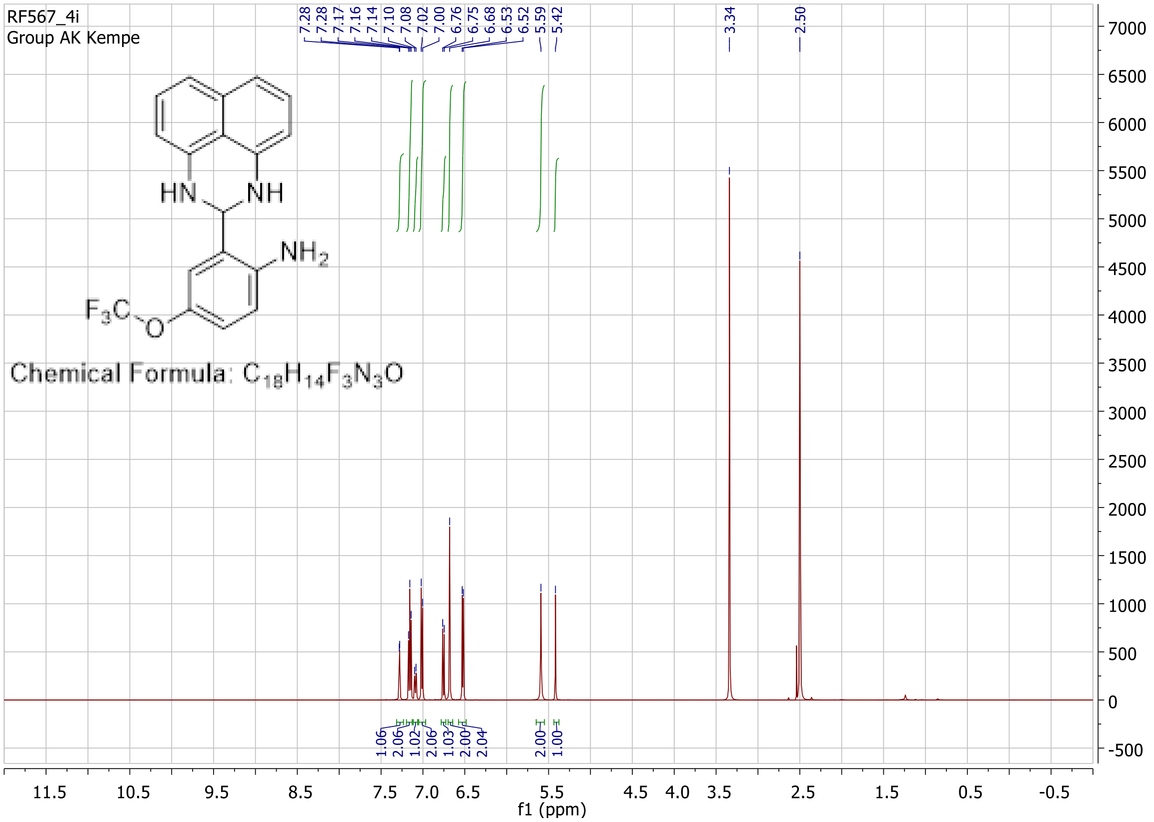

Supplementary Figure 69 ^{1}H NMR spectrum of compound **A18**. (500 MHz, 293 K, DMSO-d_6).

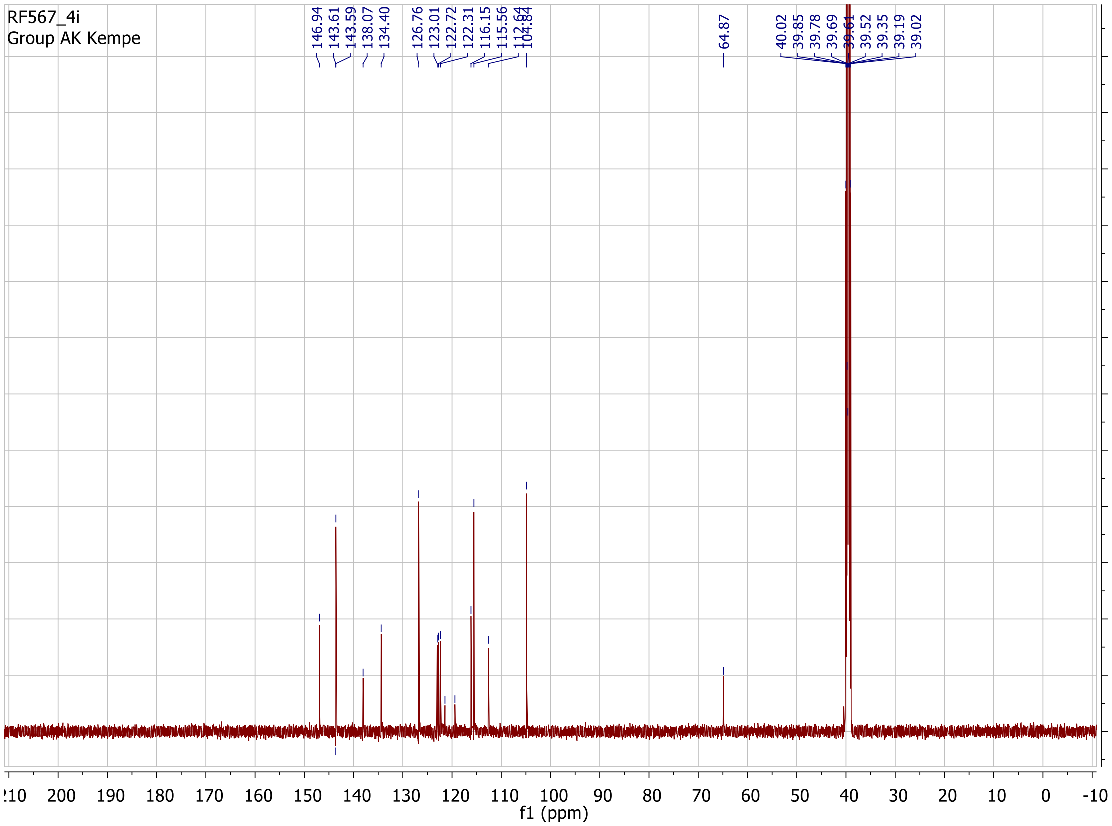

Supplementary Figure 70 ^{13}C NMR spectrum of compound **A18**. (125 MHz, 293 K, DMSO-d_6).

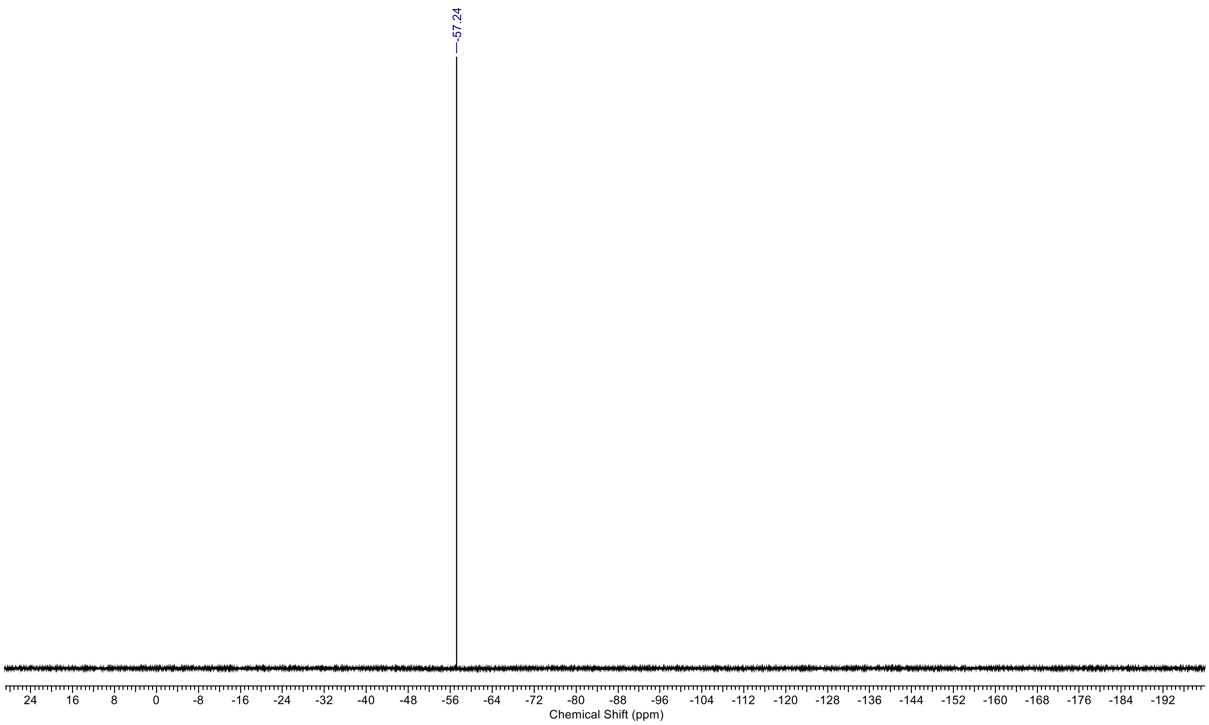

Supplementary Figure 71 ^{19}F NMR spectrum of compound **A18**. (376 MHz, 293 K, DMSO-d_6).

NMR spectra of A19

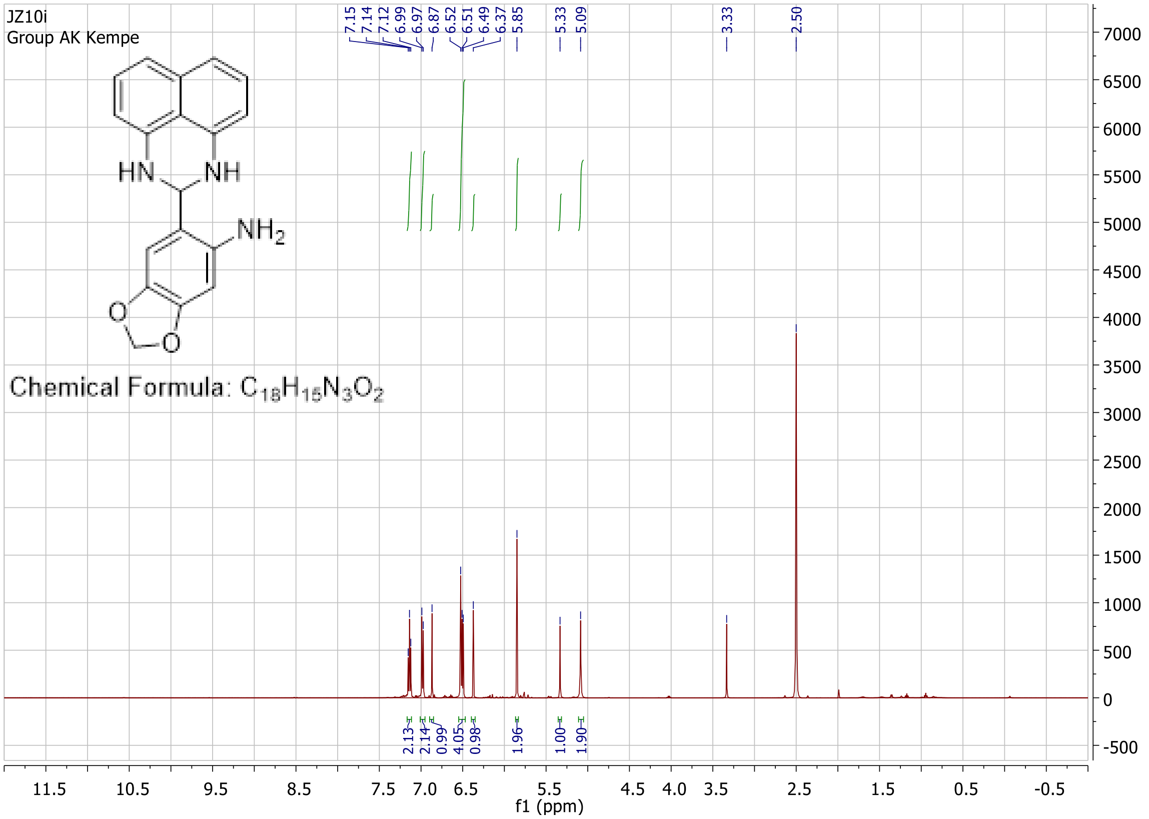

Supplementary Figure 72 ^{1}H NMR spectrum of compound **A19**. (500 MHz, 293 K, DMSO-d_6).

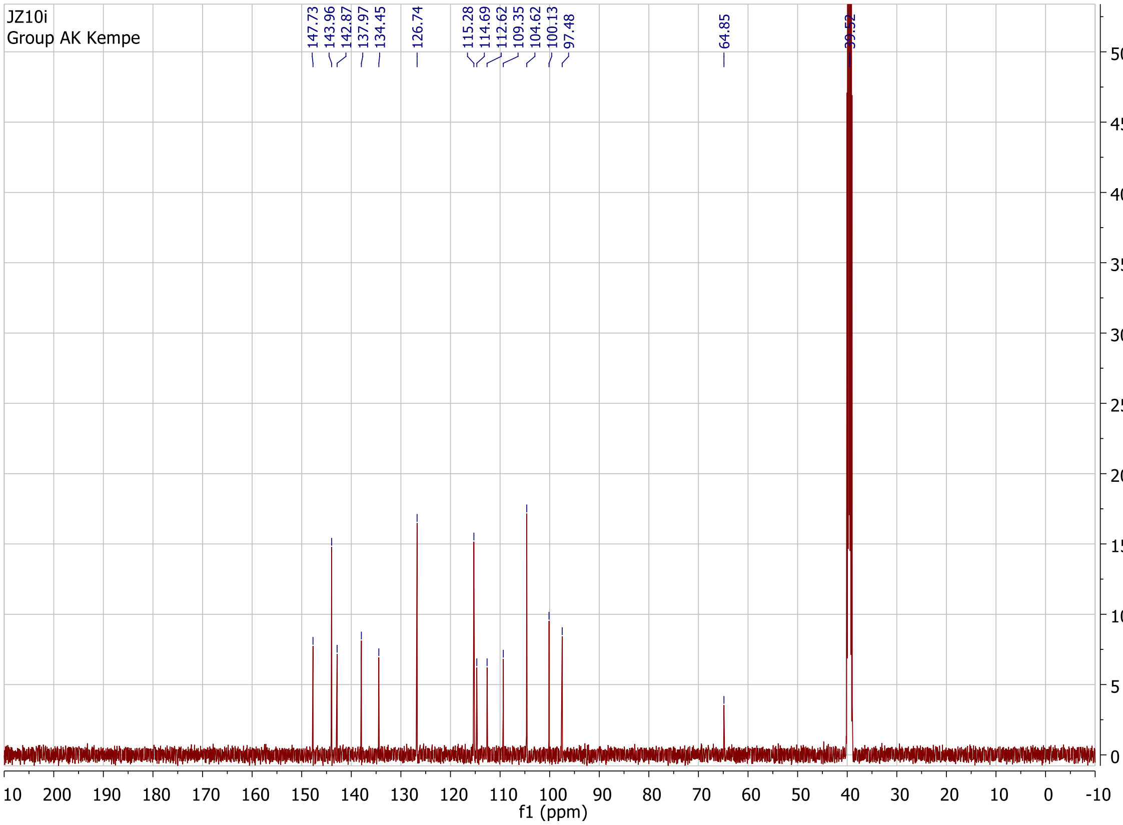

Supplementary Figure 73 ^{13}C NMR spectrum of compound **A19**. (125 MHz, 293 K, DMSO-d_6).

NMR spectra of A20

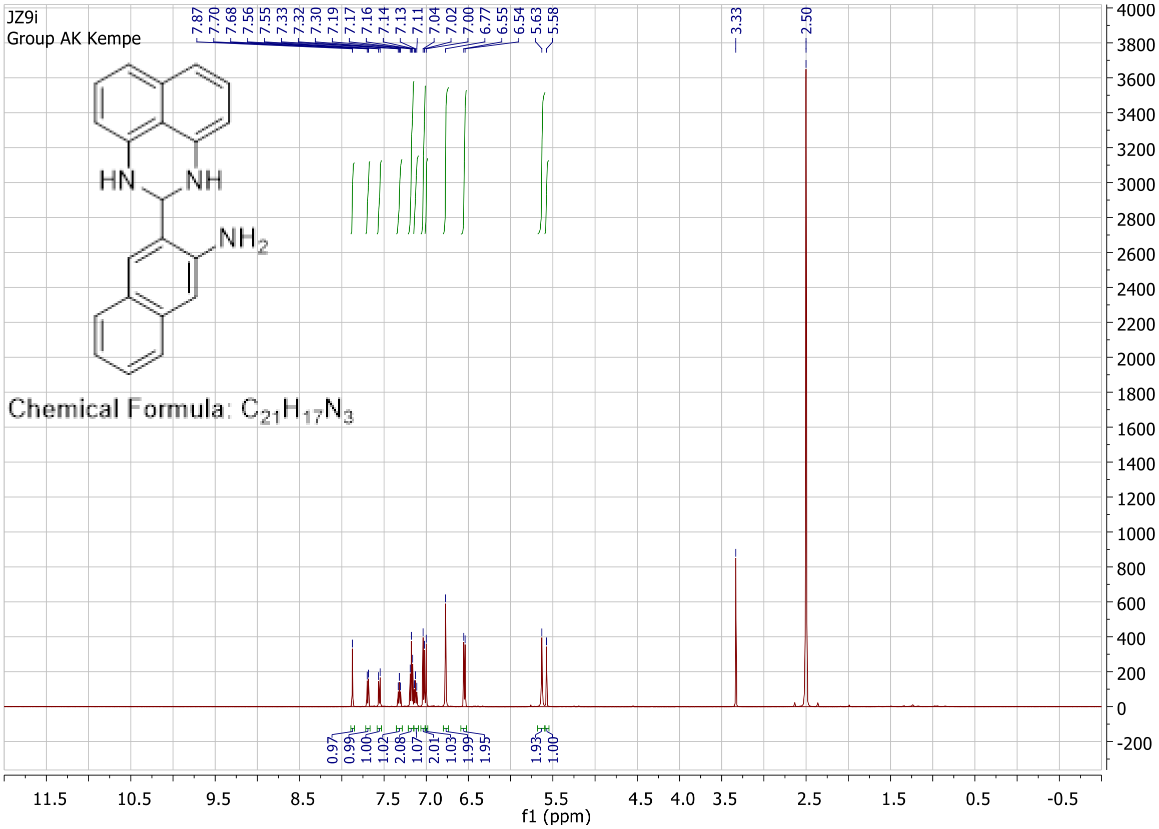

Supplementary Figure 74 ^{1}H NMR spectrum of compound **A20**. (500 MHz, 293 K, DMSO-d_6).

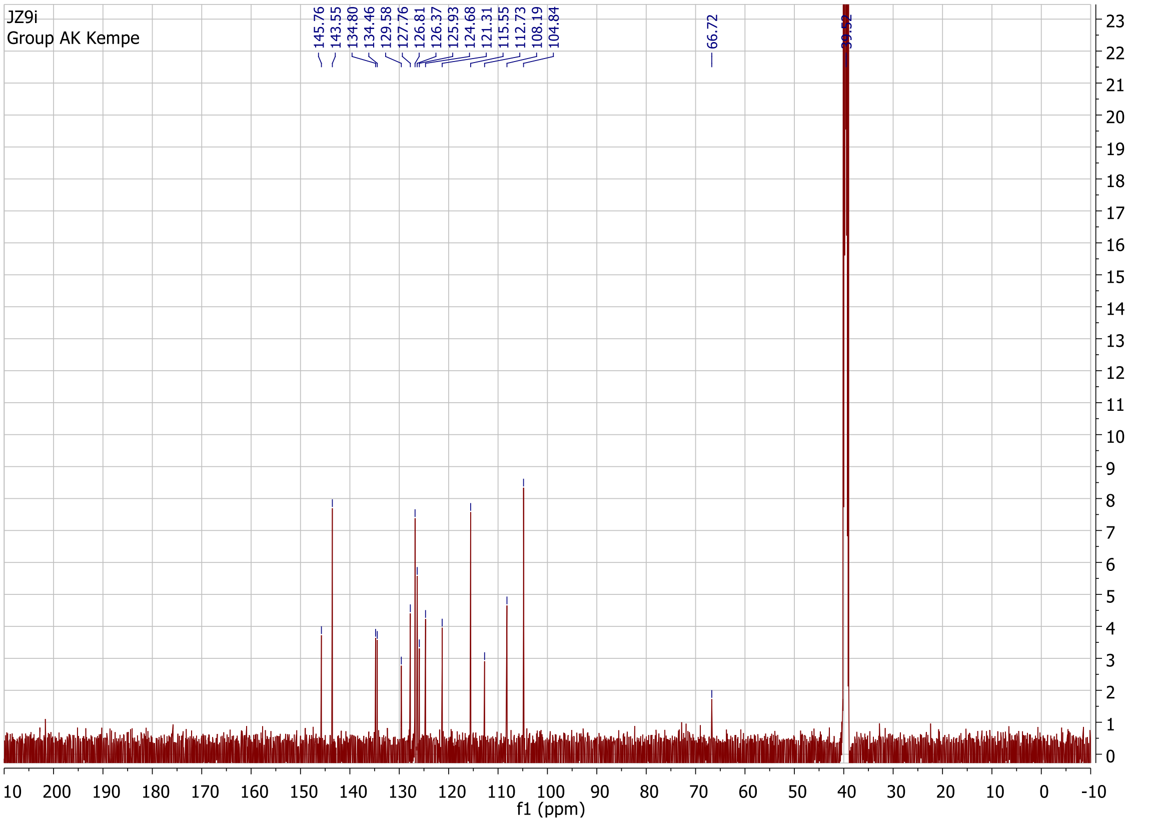

Supplementary Figure 75 ^{13}C NMR spectrum of compound **A20**. (125 MHz, 293 K, DMSO-d_6).

NMR spectra of A21

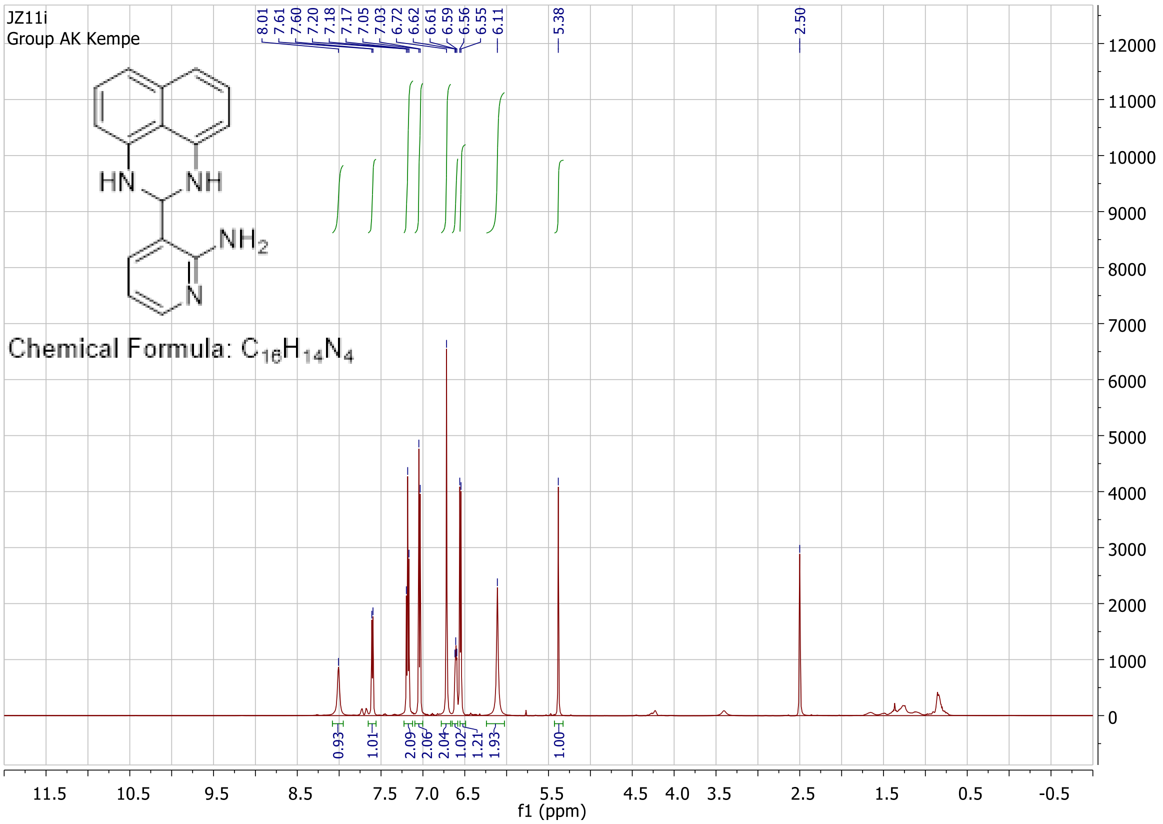

Supplementary Figure 76 ^{1}H NMR spectrum of compound **A21**. (500 MHz, 293 K, DMSO-d_6).

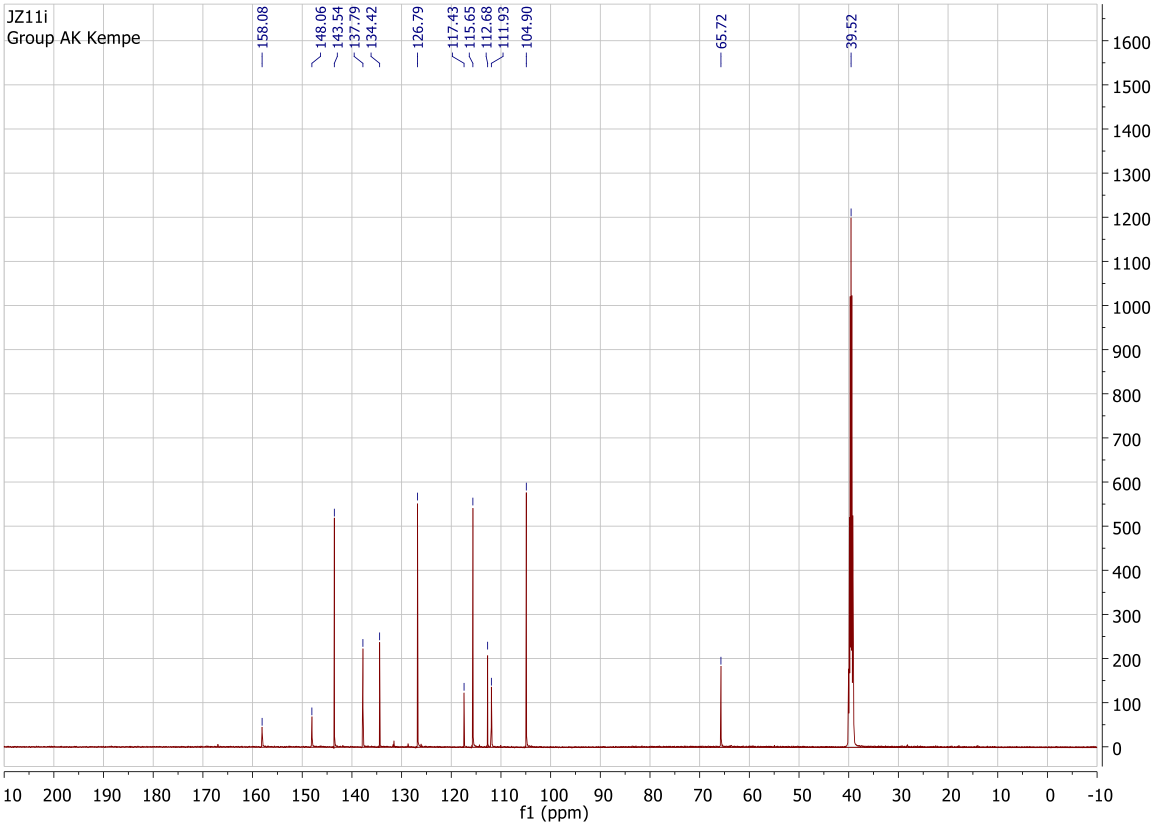

Supplementary Figure 77 ^{13}C NMR spectrum of compound **A21**. (125 MHz, 293 K, DMSO-d_6).

NMR spectra of A22

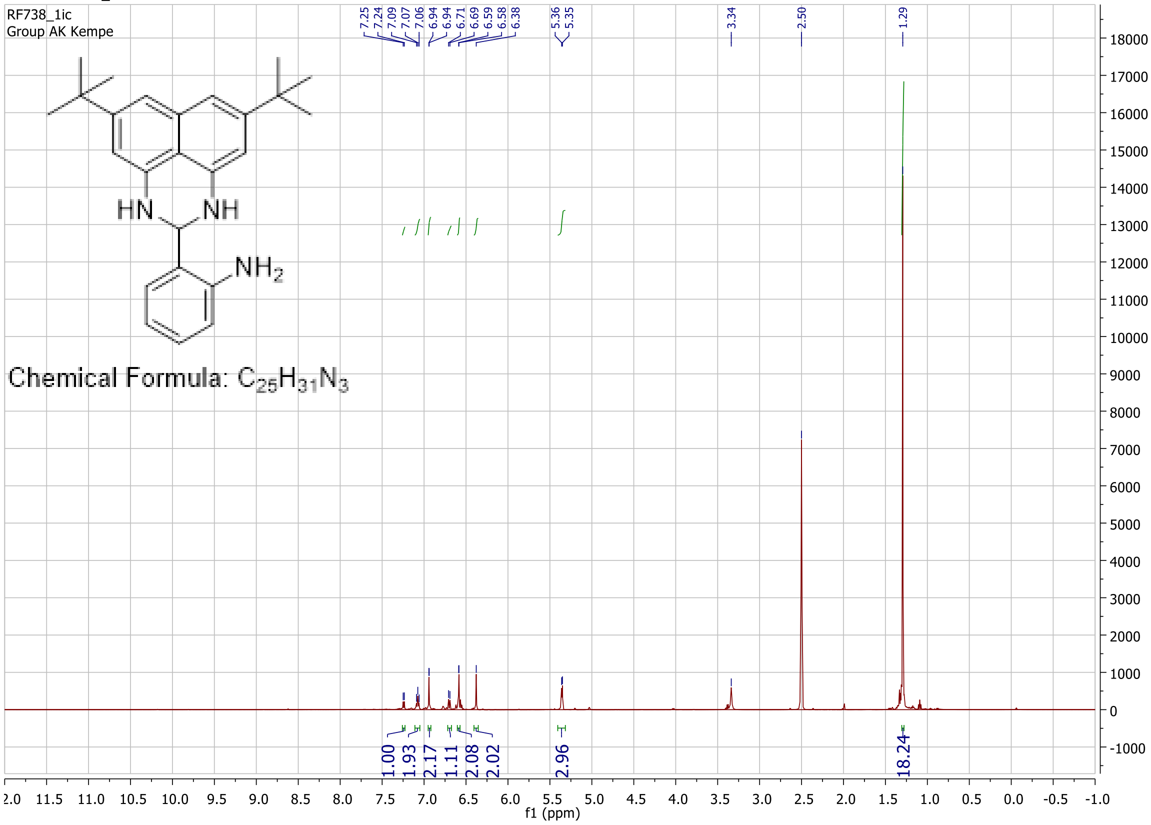

Supplementary Figure 78 ^{1}H NMR spectrum of compound **A22**. (500 MHz, 293 K, DMSO-d_6).

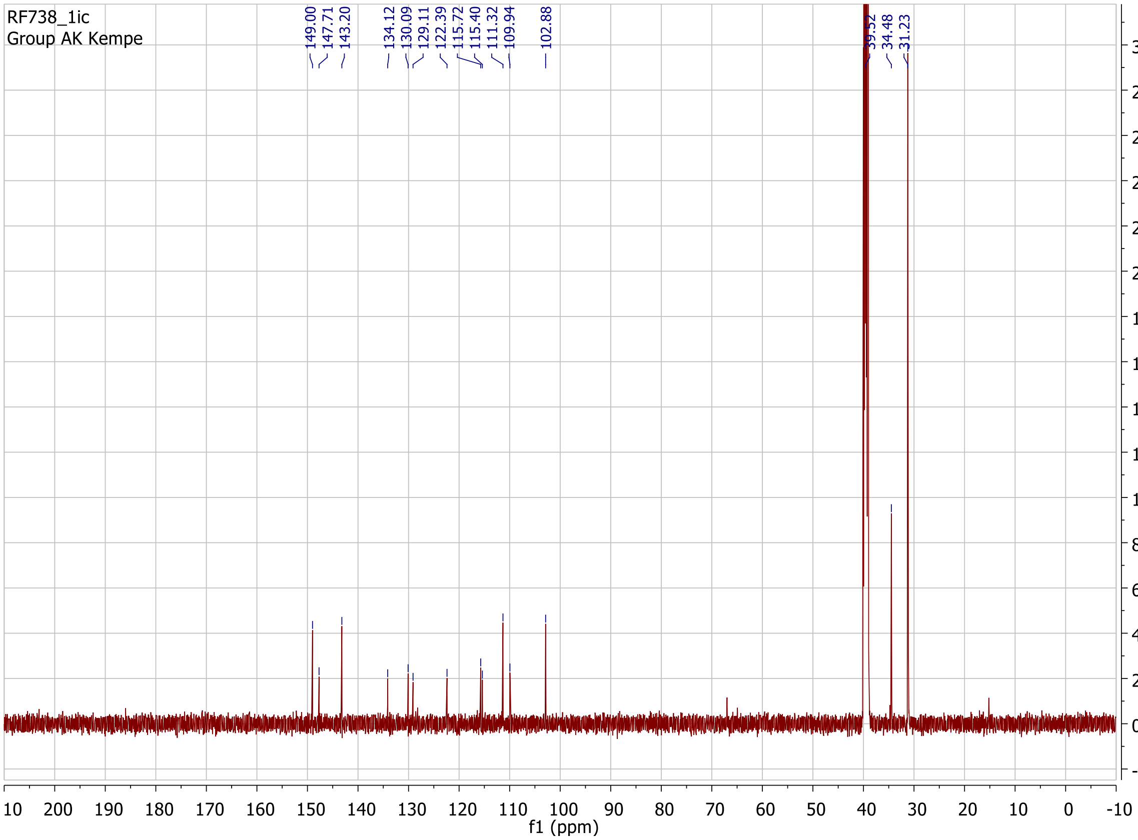

Supplementary Figure 79 ^{13}C NMR spectrum of compound **A22**. (125 MHz, 293 K, DMSO-d_6).

NMR spectra A23

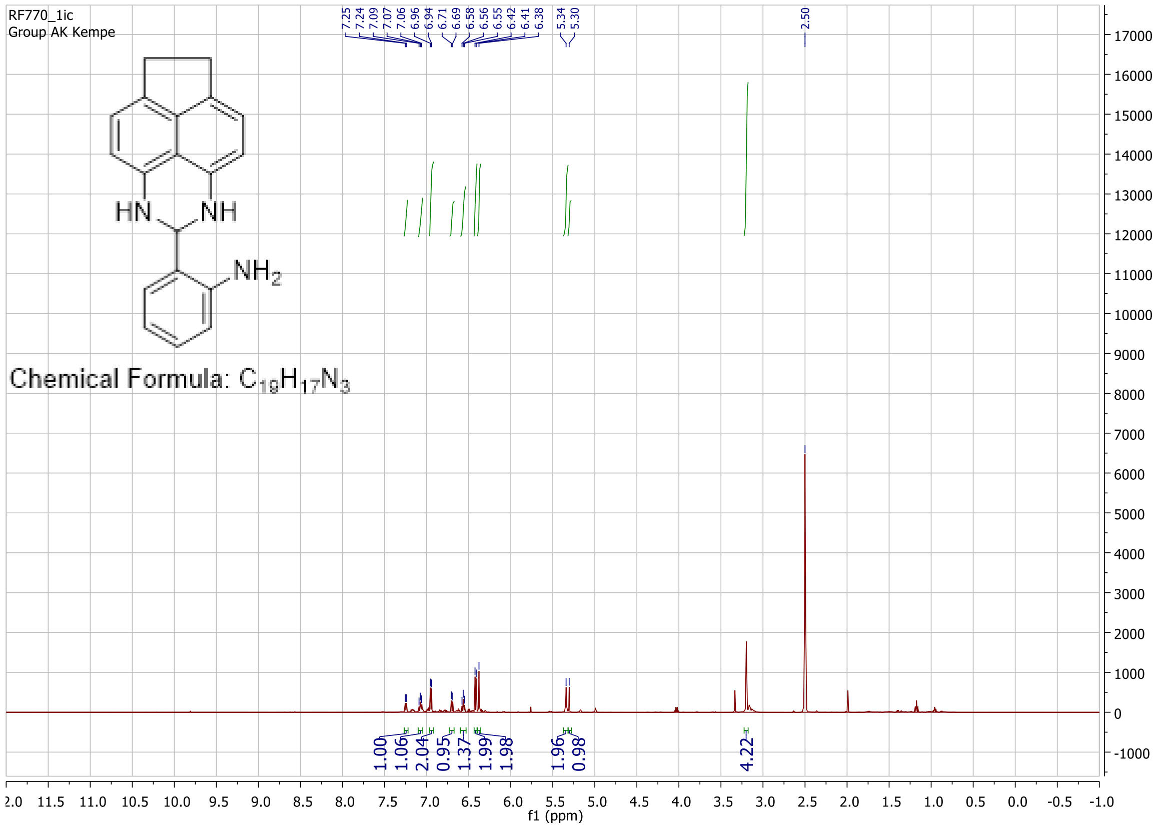

Supplementary Figure 80 ^{1}H NMR spectrum of compound **A23**. (500 MHz, 293 K, DMSO-d_6).

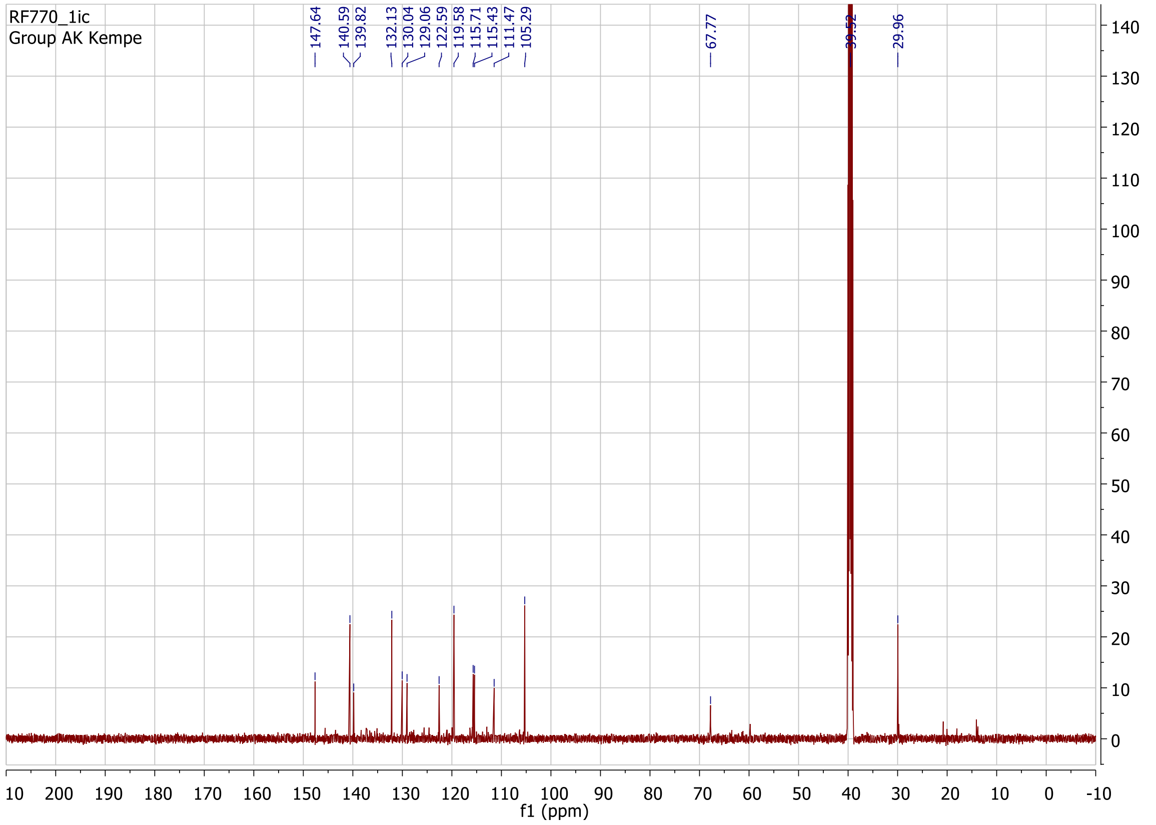

Supplementary Figure 81 ^{13}C NMR spectrum of compound **A23**. (125 MHz, 293 K, DMSO-d_6).

NMR spectra of A24

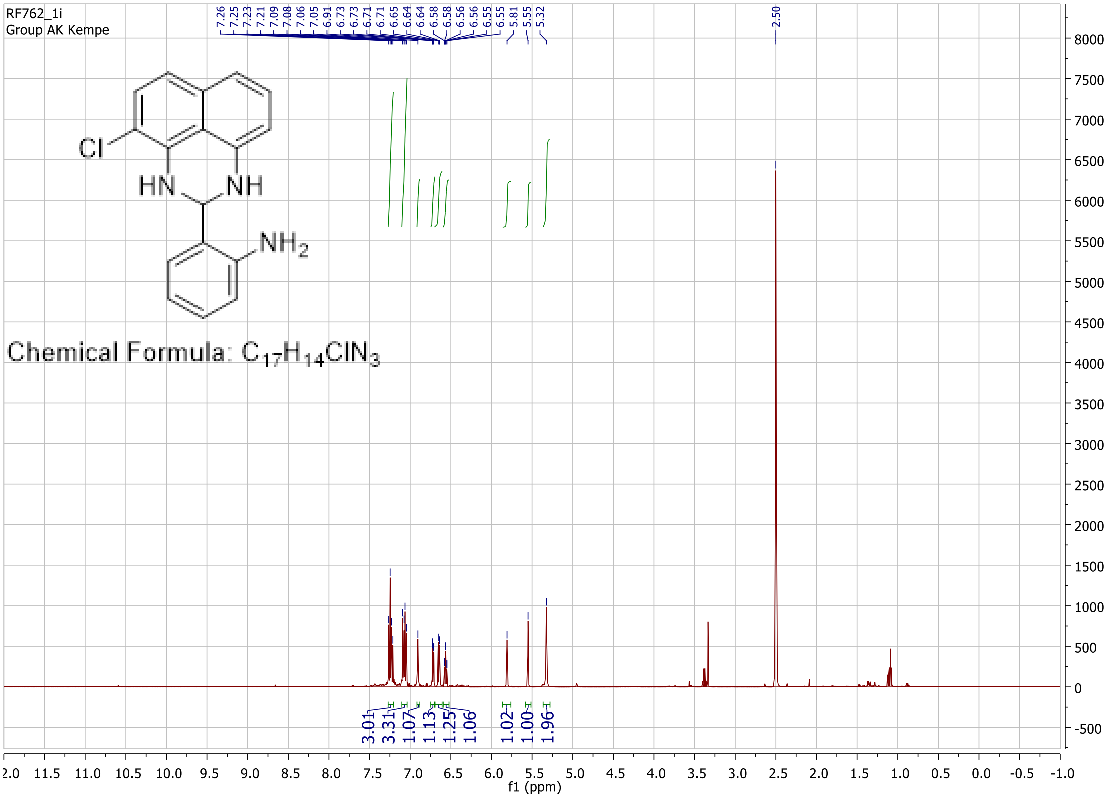

Supplementary Figure 82 ^{1}H NMR spectrum of compound **A24**. (500 MHz, 293 K, DMSO-d$_6$).

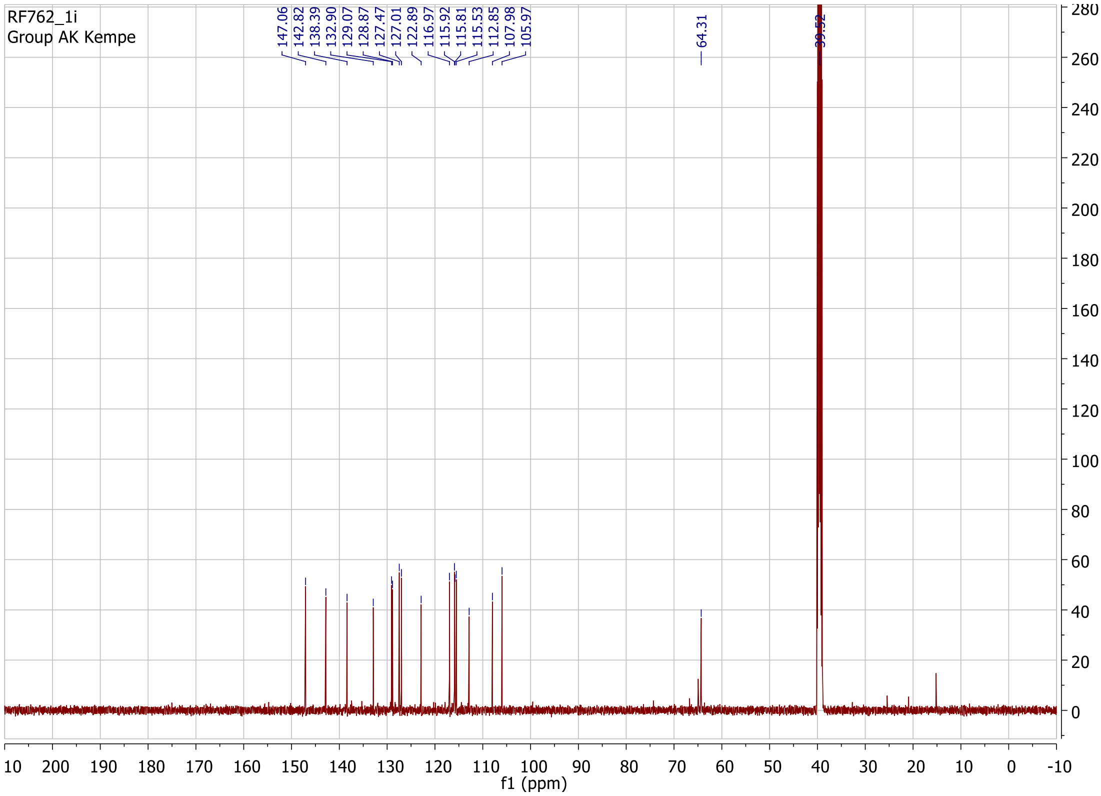

Supplementary Figure 83 ^{13}C NMR spectrum of compound **A24**. (125 MHz, 293 K, DMSO-d$_6$).

NMR spectra of A25

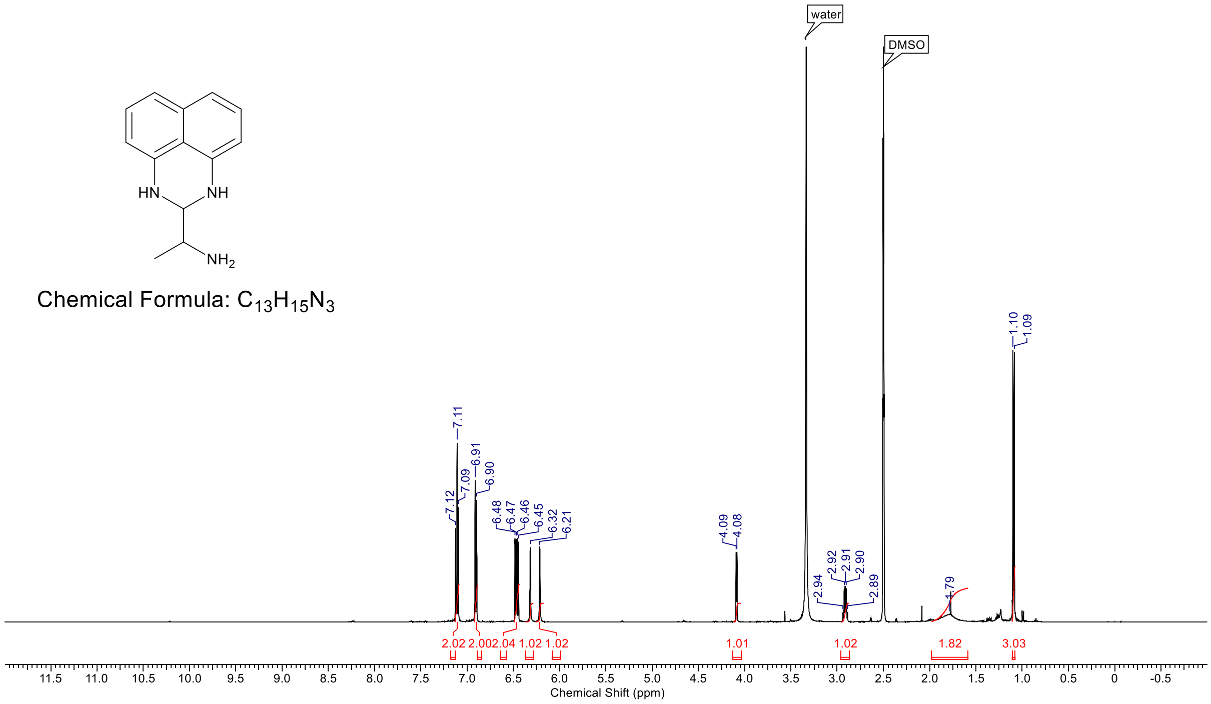

Supplementary Figure 84 ^{1}H NMR spectrum of compound **A25**. (500 MHz, 293 K, DMSO-d_6).

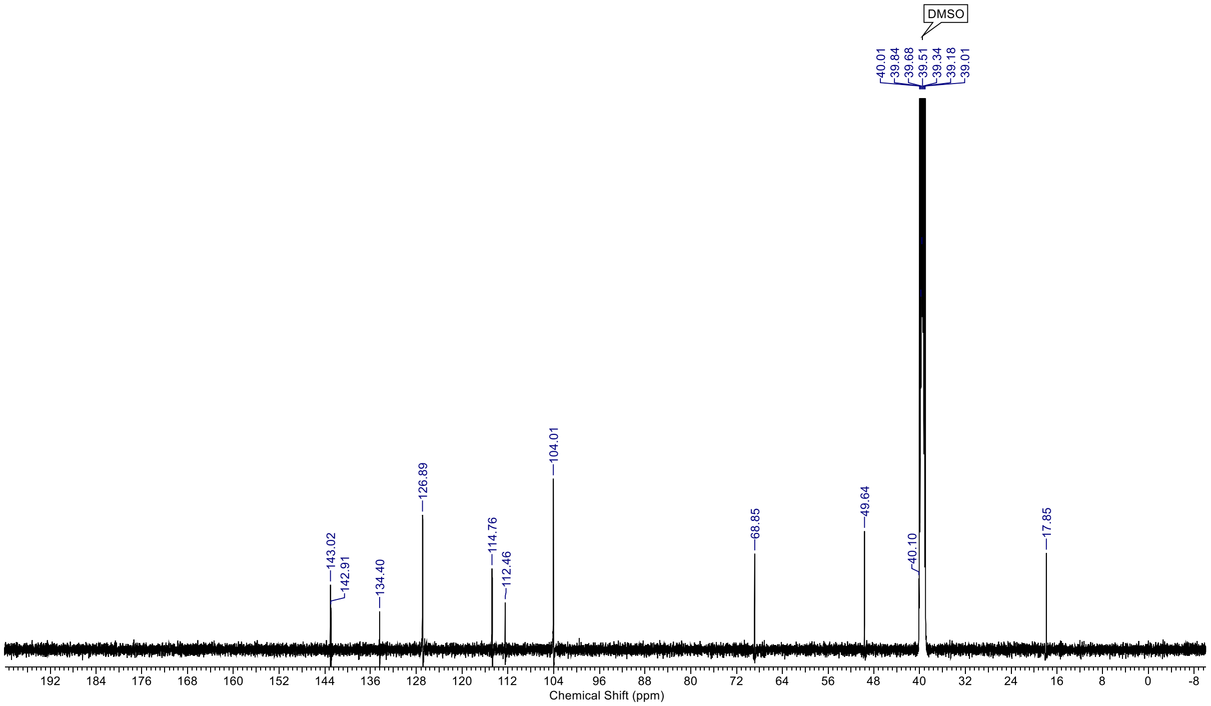

Supplementary Figure 85 ^{13}C NMR spectrum of compound **A25**. (125 MHz, 293 K, DMSO-d_6).

NMR spectra of A26

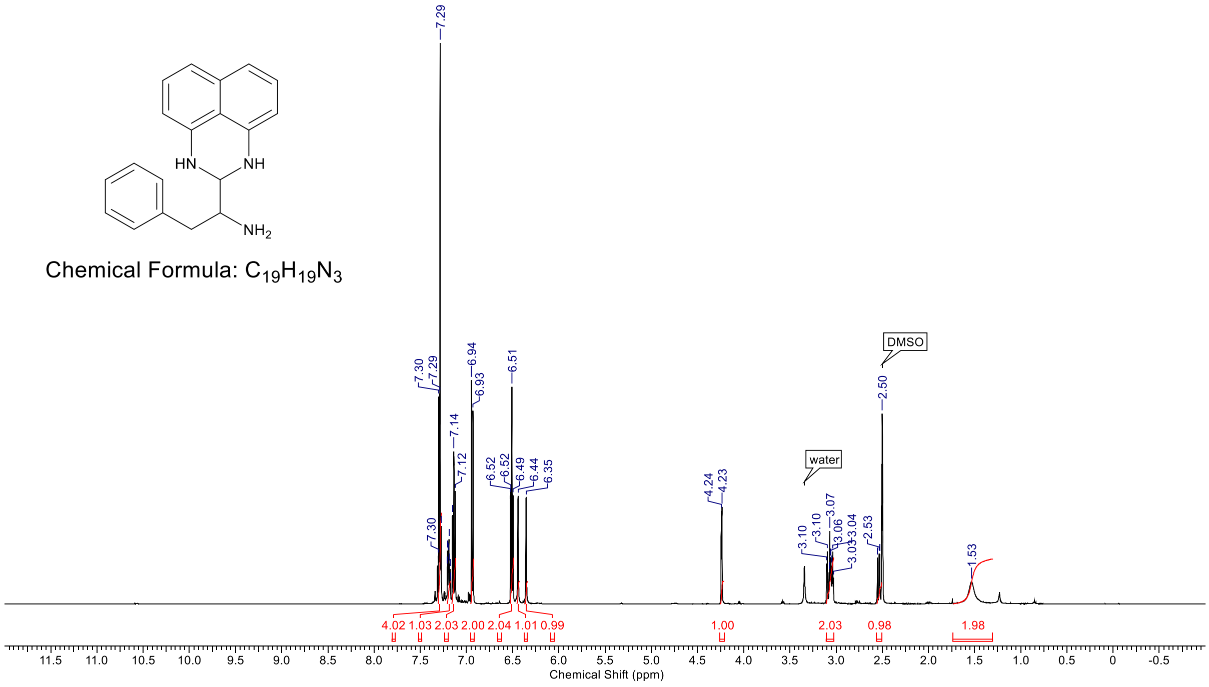

Supplementary Figure 86 ^{1}H NMR spectrum of compound **A26**. (500 MHz, 293 K, DMSO-d_6).

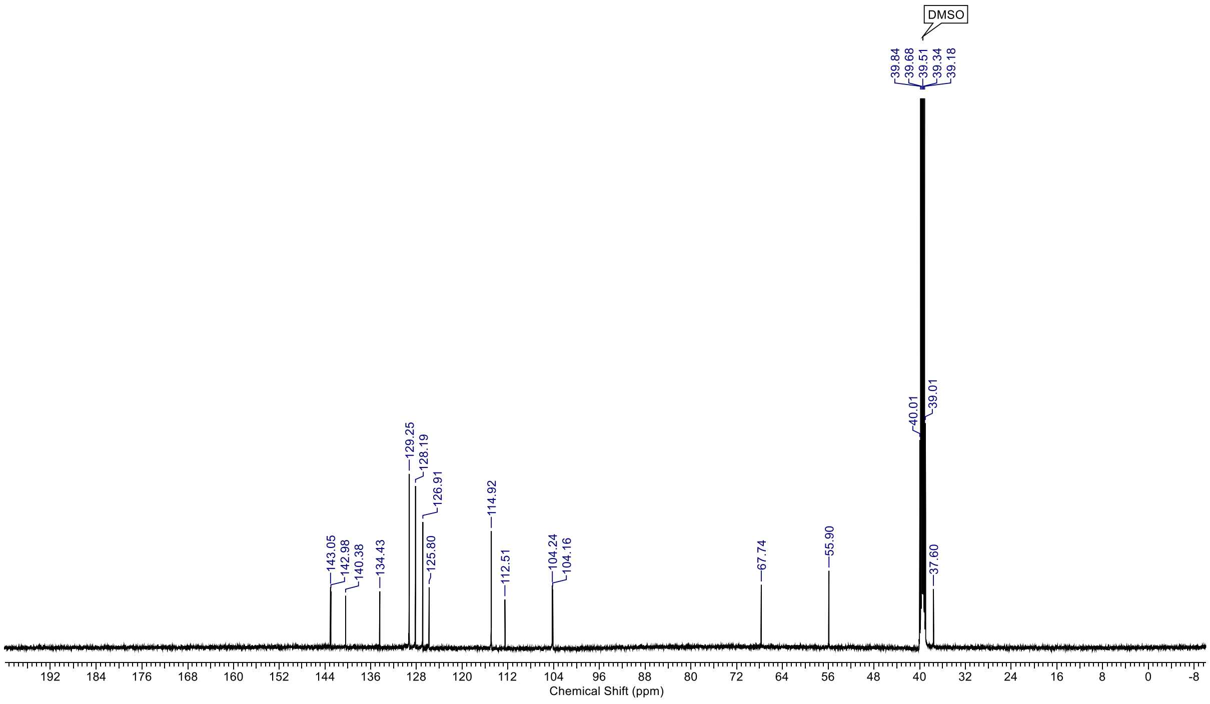

Supplementary Figure 87 ^{13}C NMR spectrum of compound **A26**. (125 MHz, 293 K, DMSO-d_6).

NMR spectra of A27

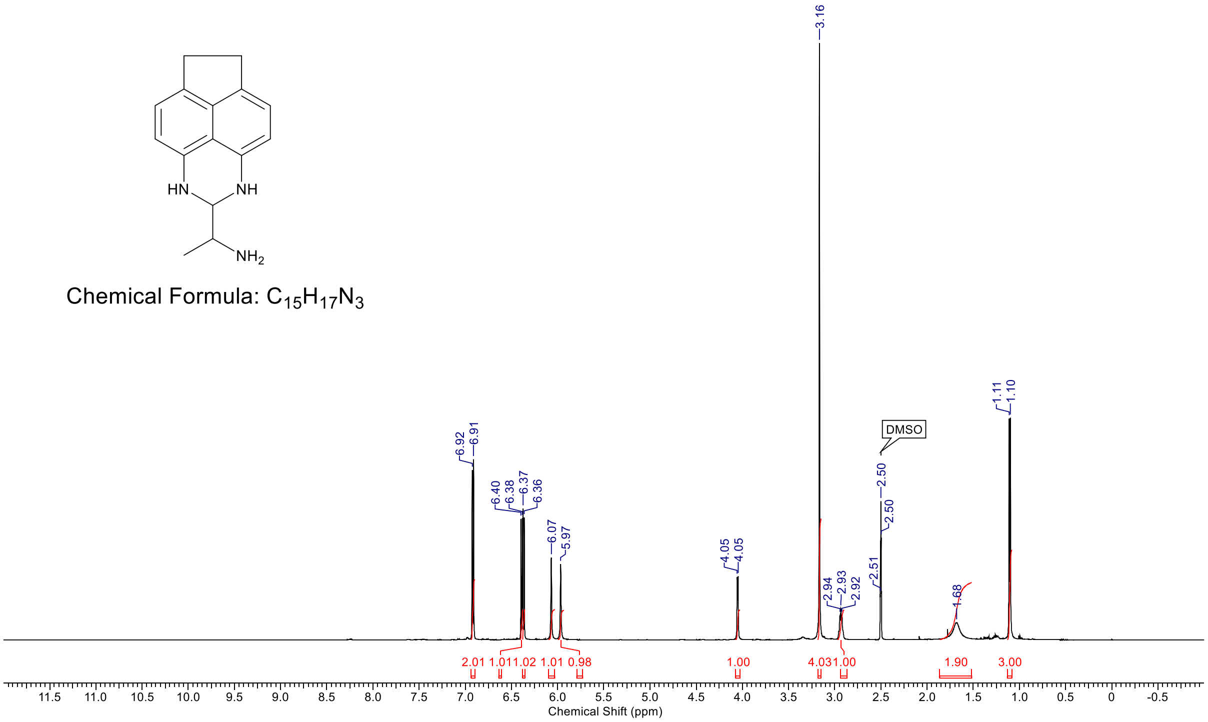

Supplementary Figure 88 ^{1}H NMR spectrum of compound **A27**. (500 MHz, 293 K, DMSO-d_6).

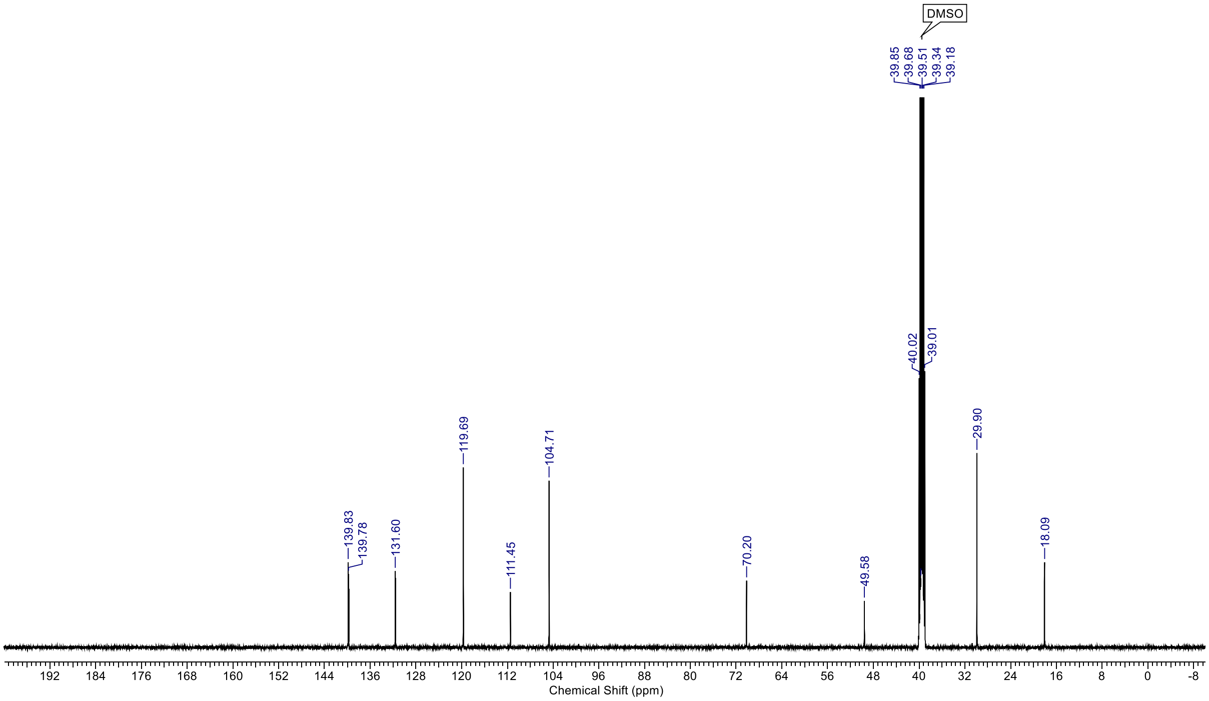

Supplementary Figure 89 ^{13}C NMR spectrum of compound **A27**. (125 MHz, 293 K, DMSO-d_6).

NMR spectra of B1a

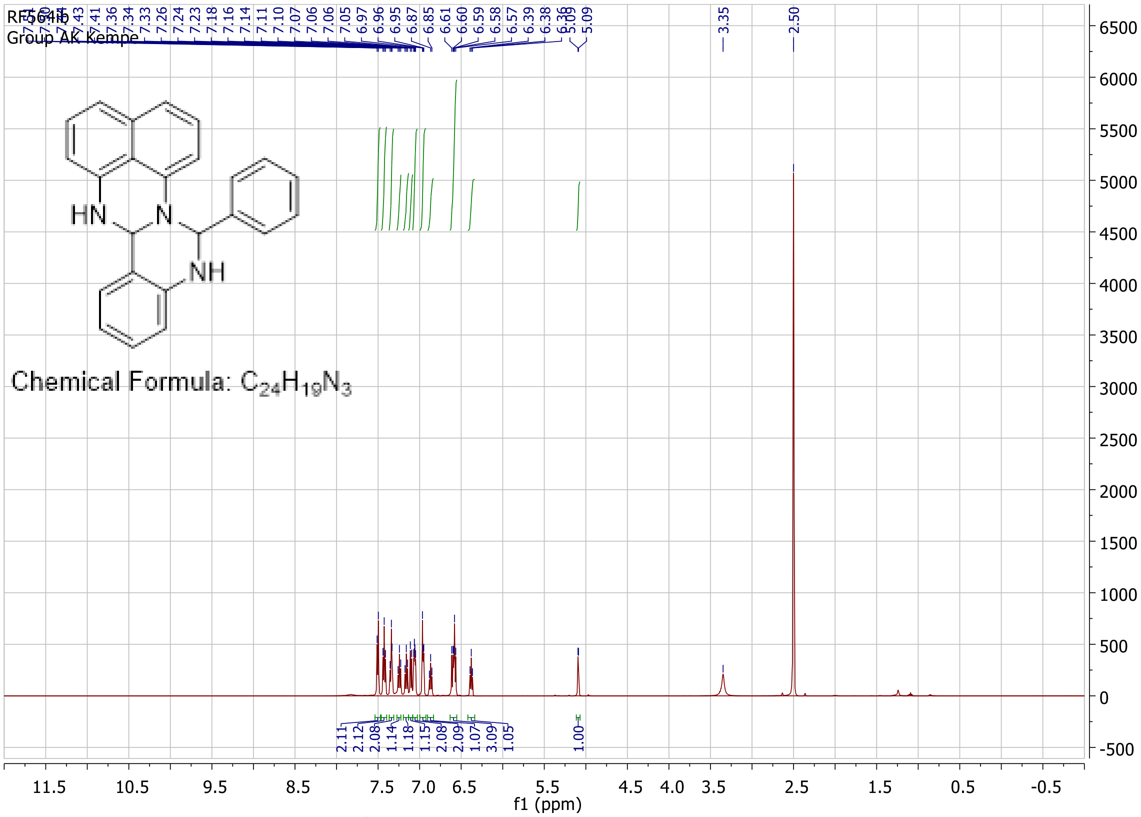

Supplementary Figure 90 ^{1}H NMR spectrum of compound **B1a**. (500 MHz, 293 K, DMSO-d_6).

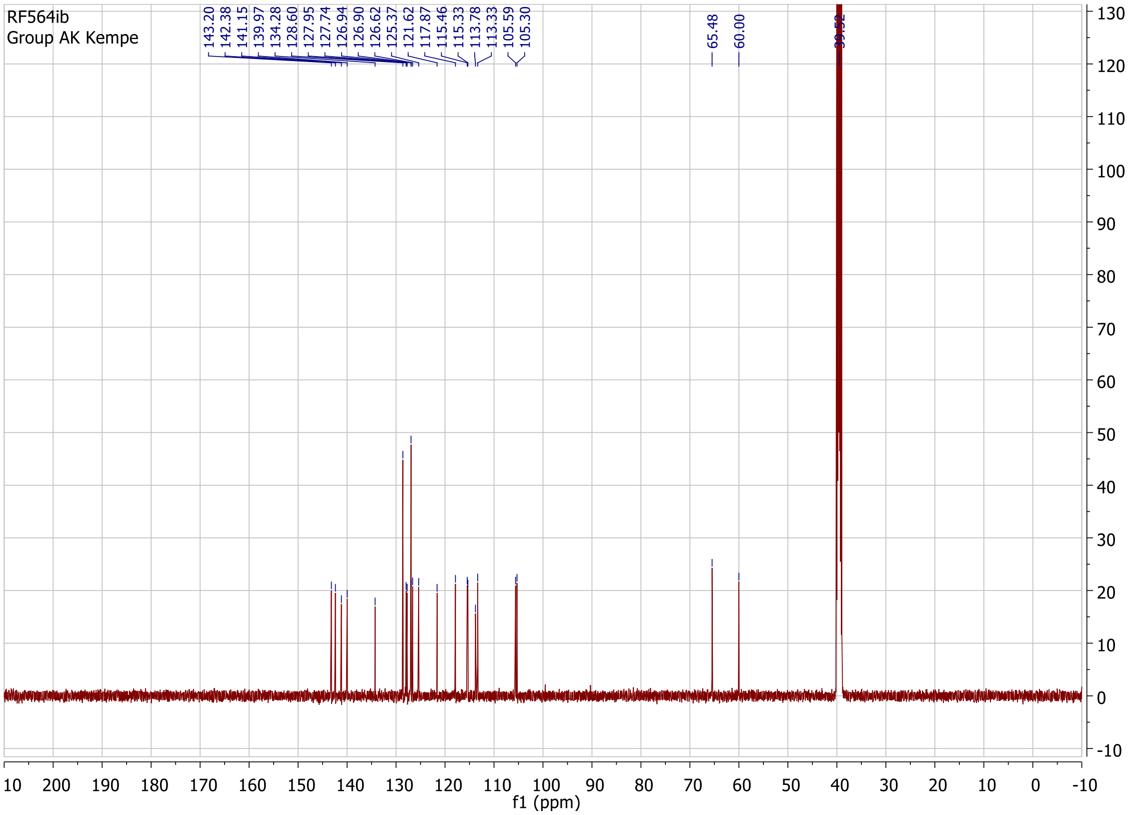

Supplementary Figure 91 ^{13}C NMR spectrum of compound **B1a**. (125 MHz, 293 K, DMSO-d_6).

NMR spectra of B1b

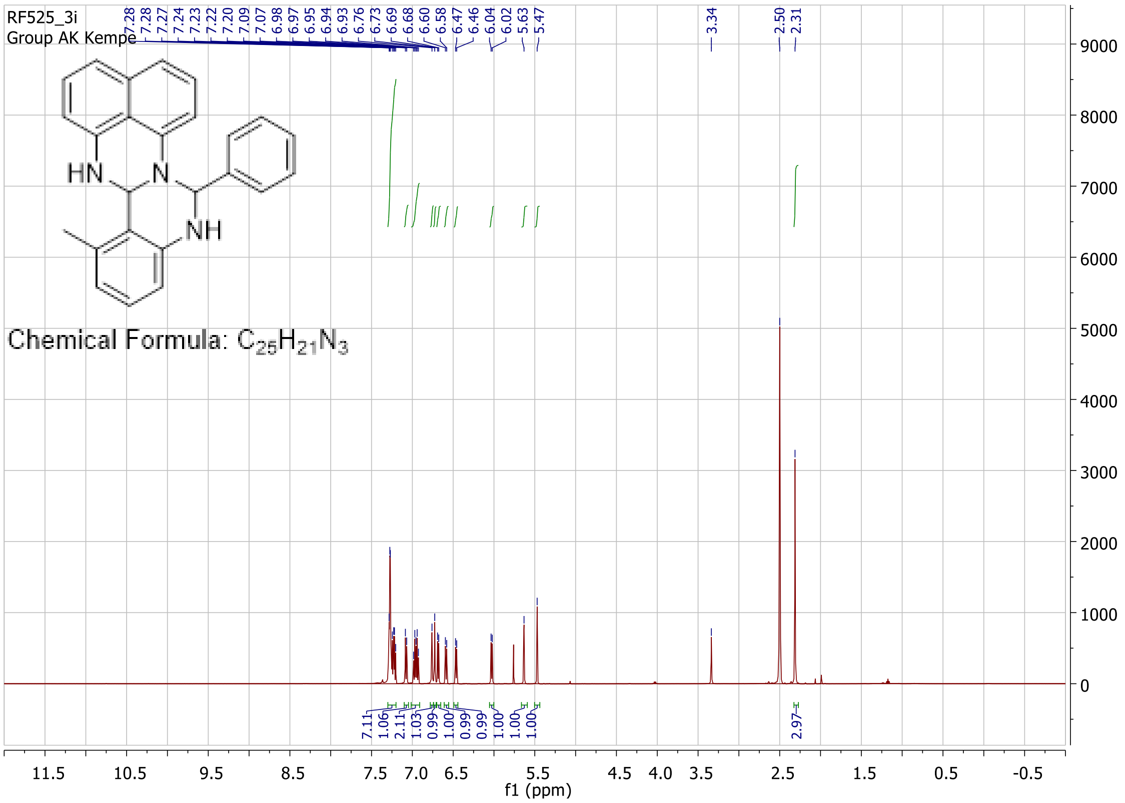

Supplementary Figure 92 ^{1}H NMR spectrum of compound **B1b**. (500 MHz, 293 K, DMSO-d$_6$).

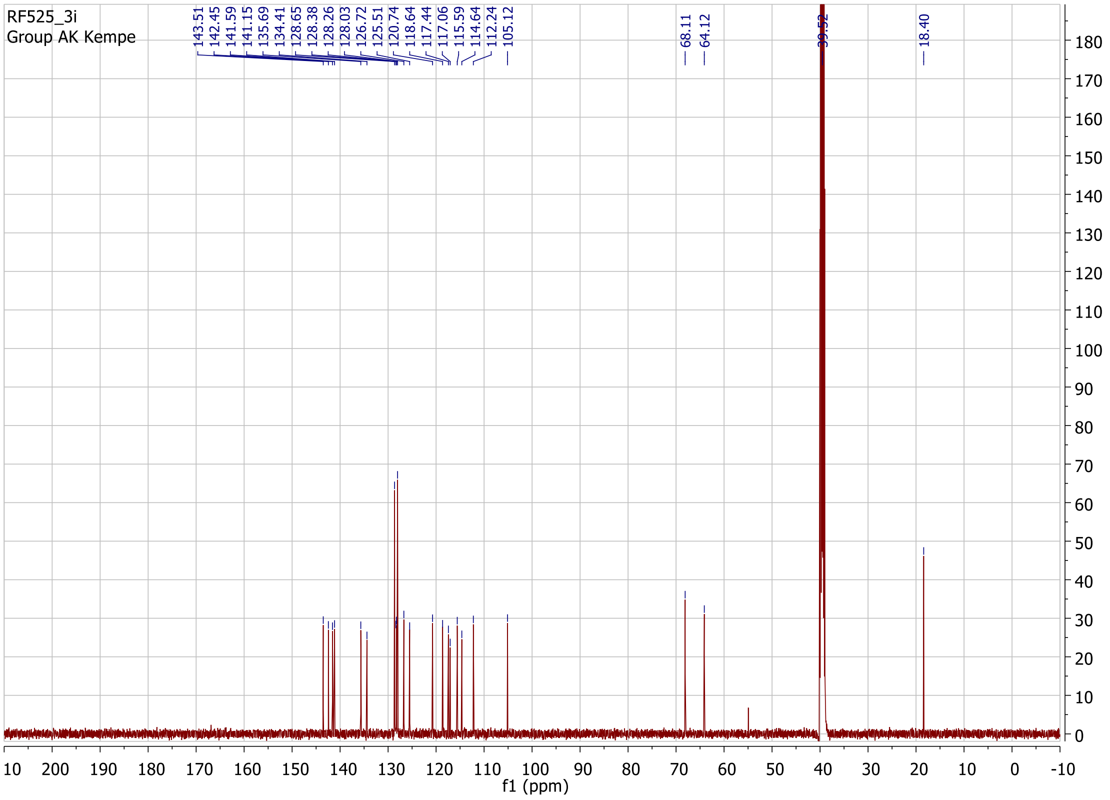

Supplementary Figure 93 ^{13}C NMR spectrum of compound **B1b**. (125 MHz, 293 K, DMSO-d$_6$).

NMR spectra of B1c

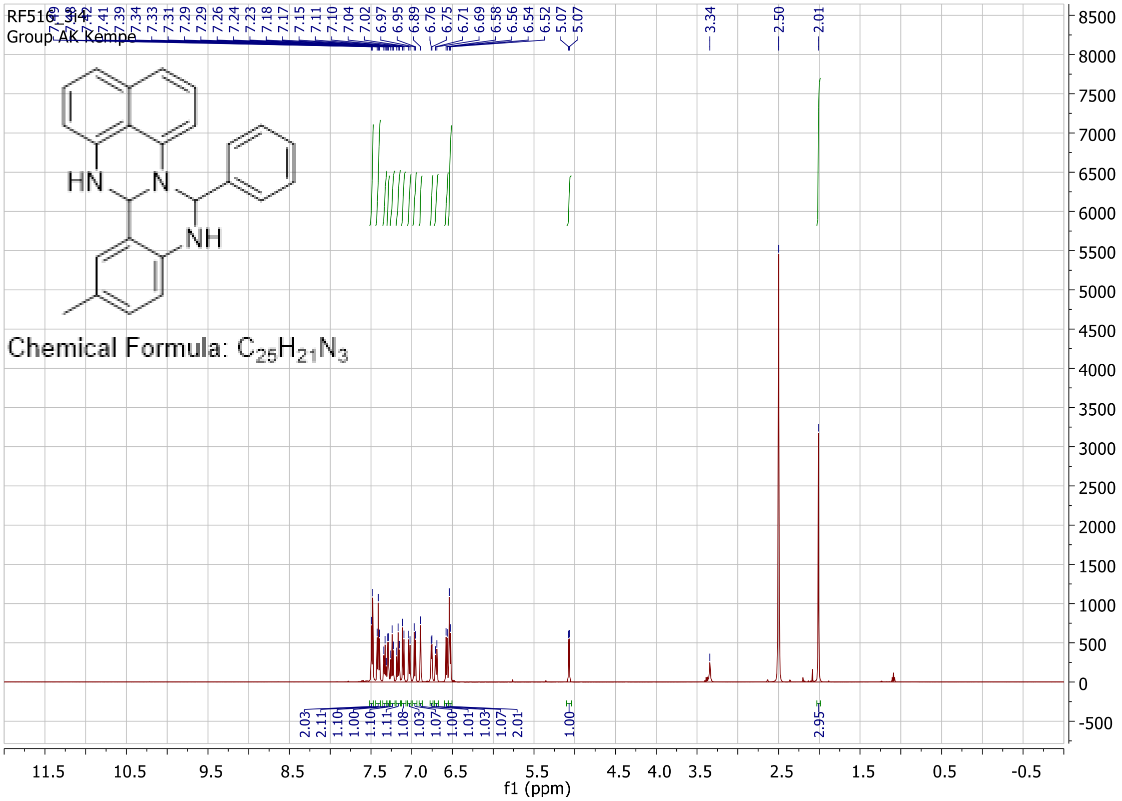

Supplementary Figure 94 ^{1}H NMR spectrum of compound **B1c**. (500 MHz, 293 K, DMSO-d_6).

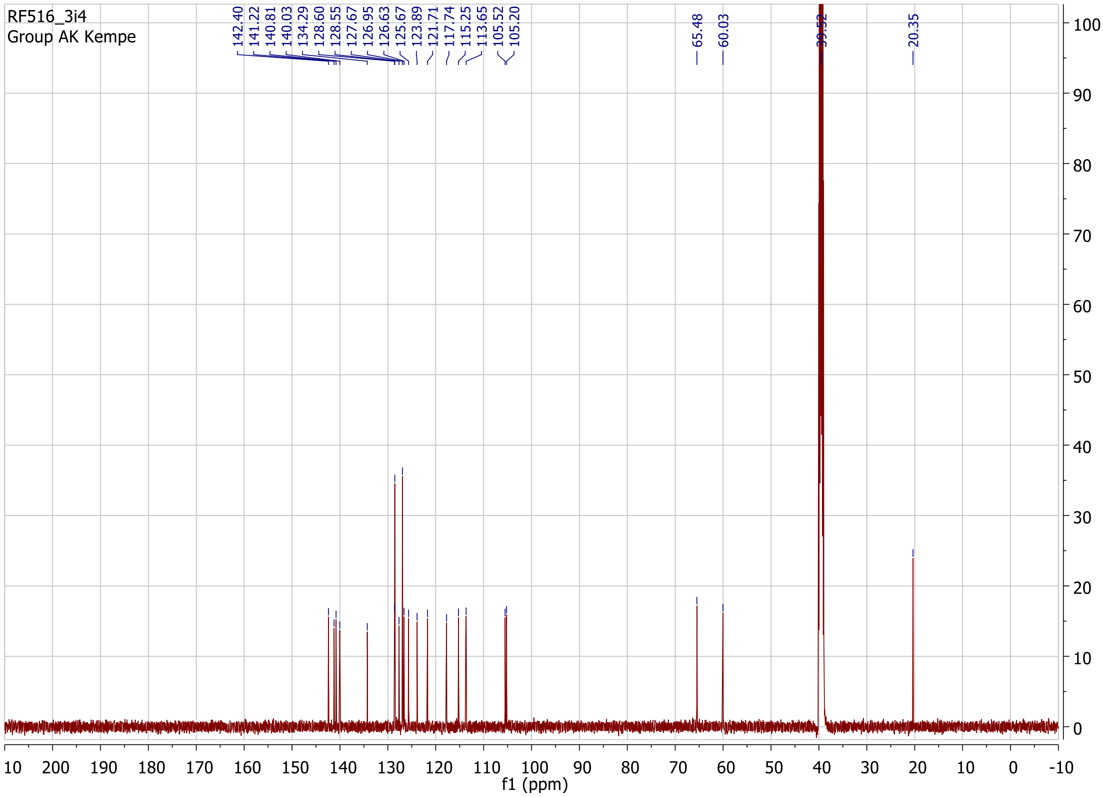

Supplementary Figure 95 ^{13}C NMR spectrum of compound **B1c**. (125 MHz, 293 K, DMSO-d_6).

NMR spectra of B1d

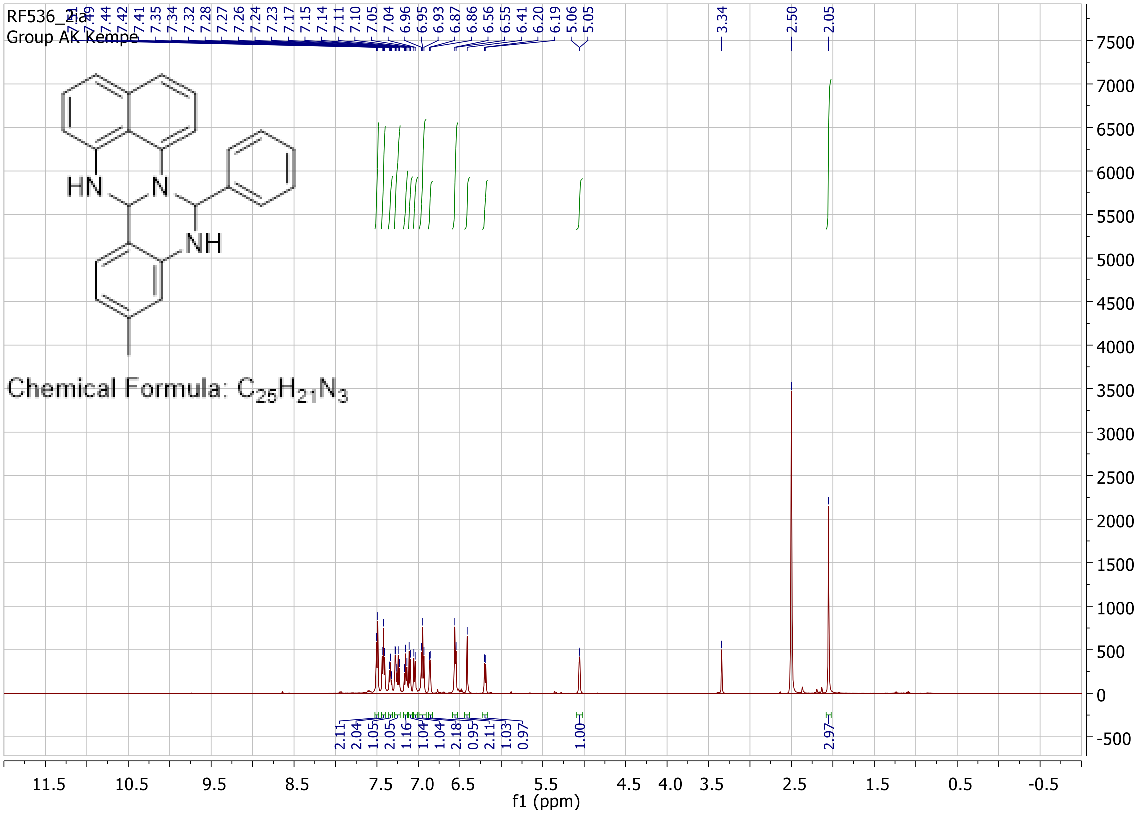

Supplementary Figure 96 ^{1}H NMR spectrum of compound **B1d**. (500 MHz, 293 K, DMSO-d_6).

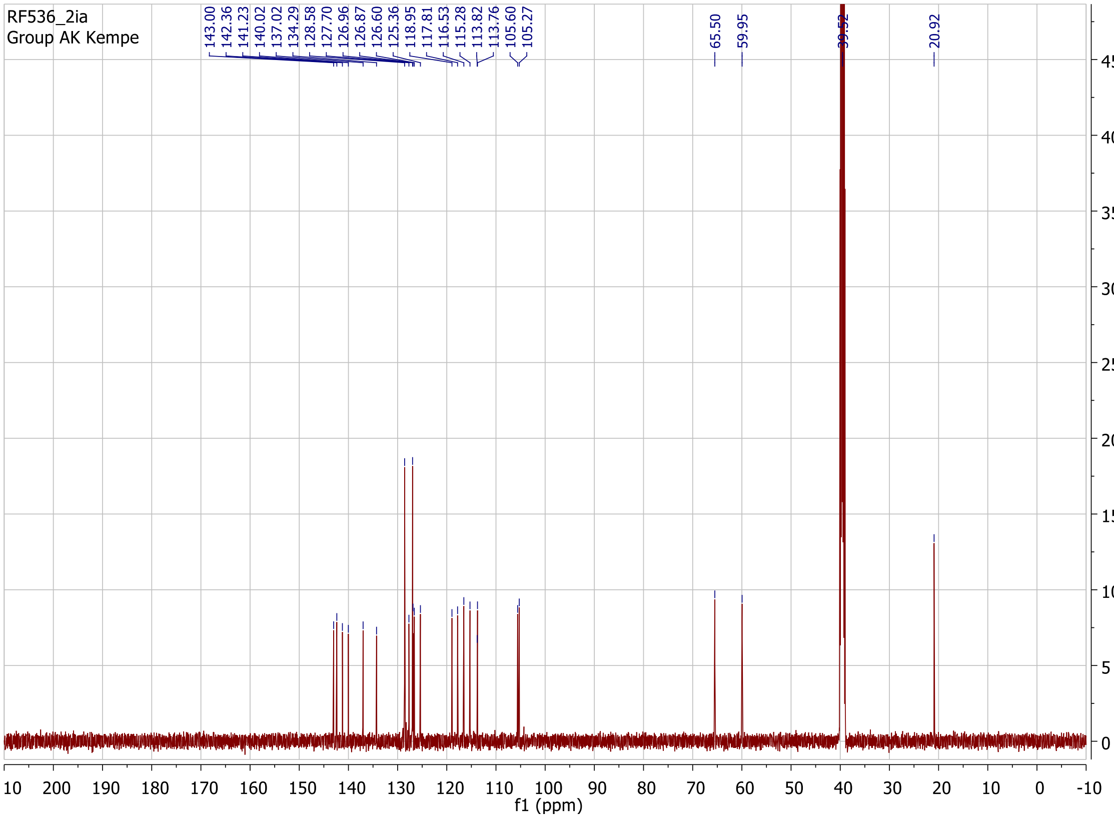

Supplementary Figure 97 ^{13}C NMR spectrum of compound **B1d**. (125 MHz, 293 K, DMSO-d_6).

NMR spectra of B1e

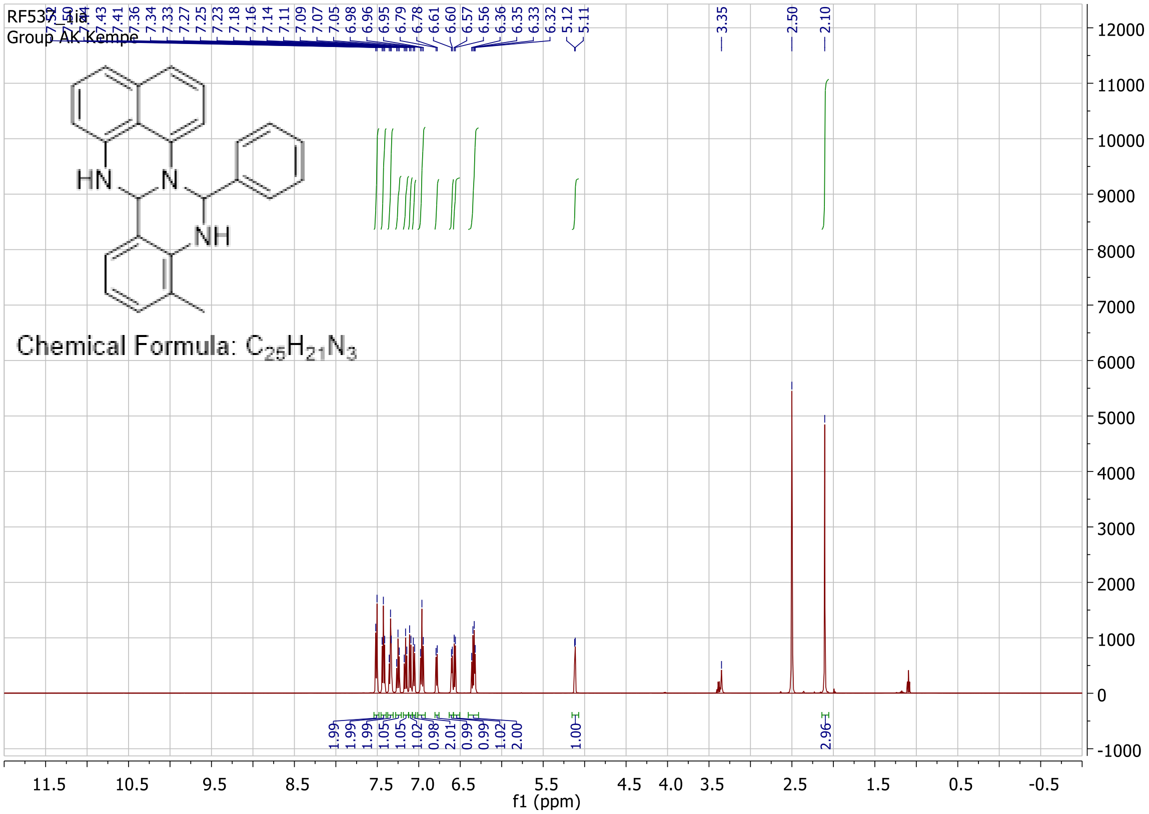

Supplementary Figure 98 ^{1}H NMR spectrum of compound **B1e**. (500 MHz, 293 K, DMSO-d$_6$).

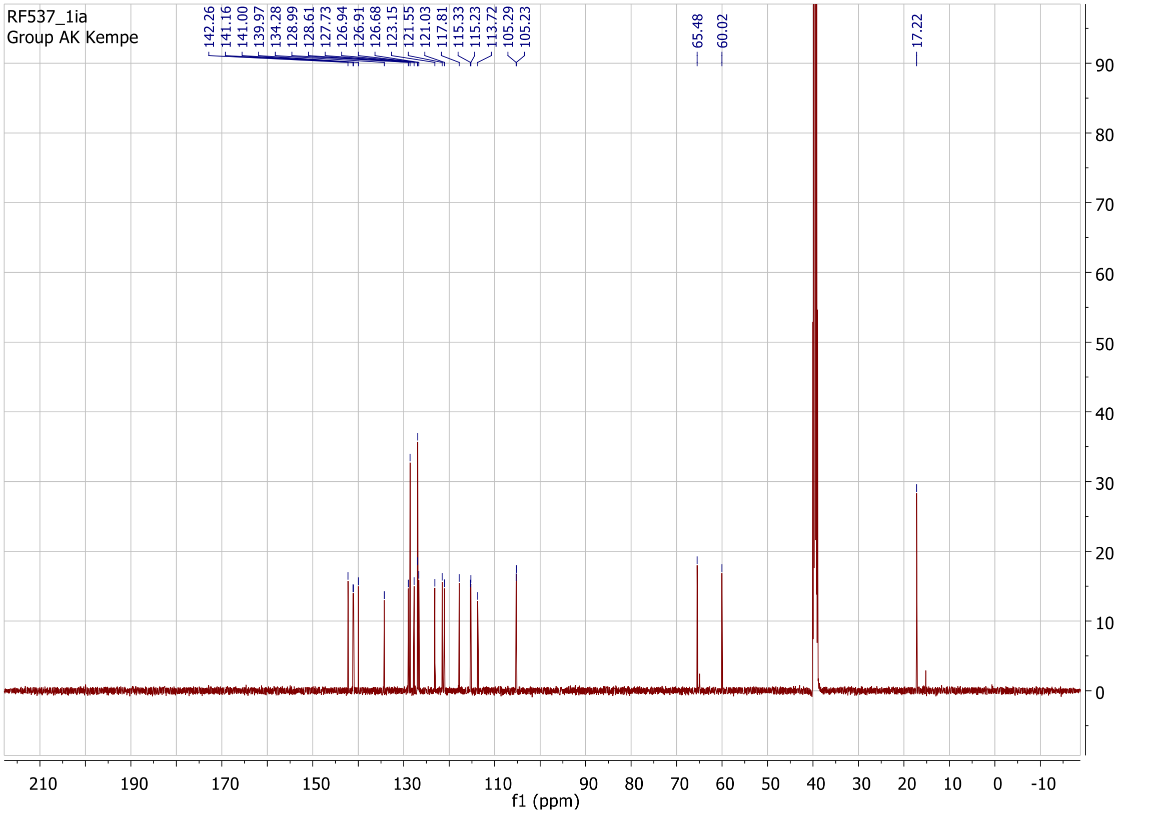

Supplementary Figure 99 ^{13}C NMR spectrum of compound **B1e**. (125 MHz, 293 K, DMSO-d$_6$).

NMR spectra of B1f

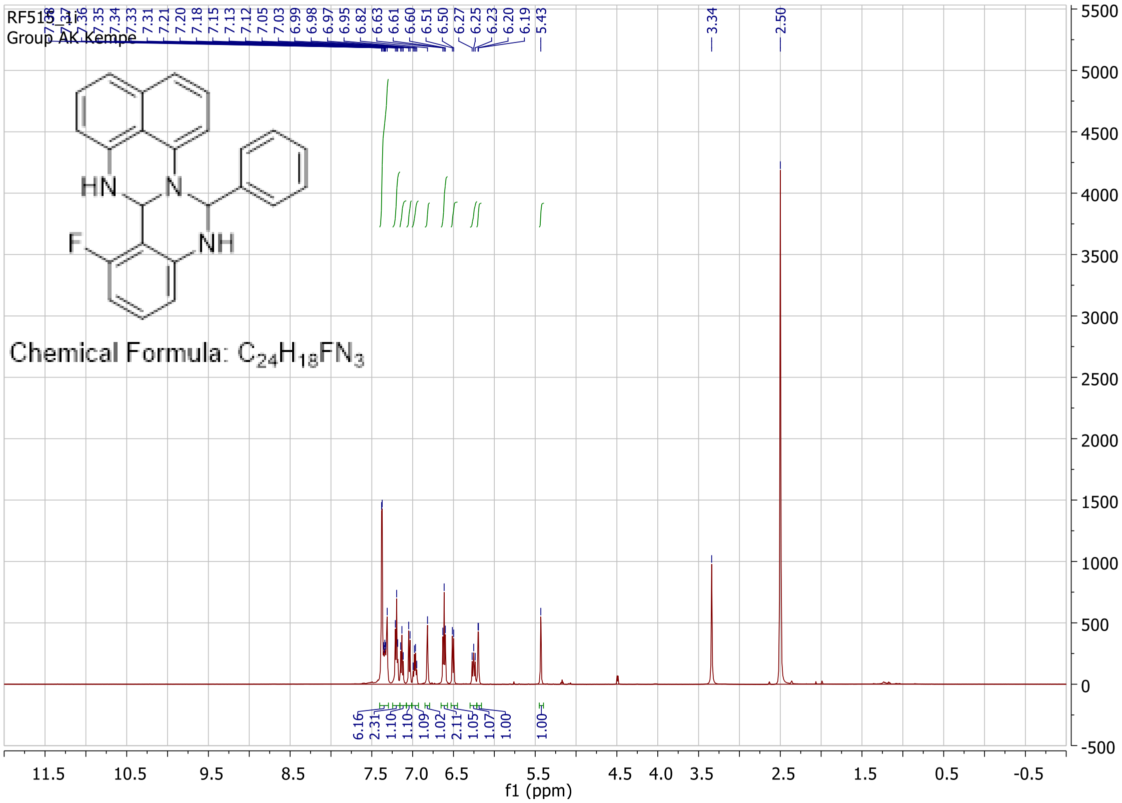

Supplementary Figure 100 ^{1}H NMR spectrum of compound **B1f**. (500 MHz, 293 K, DMSO-d_6).

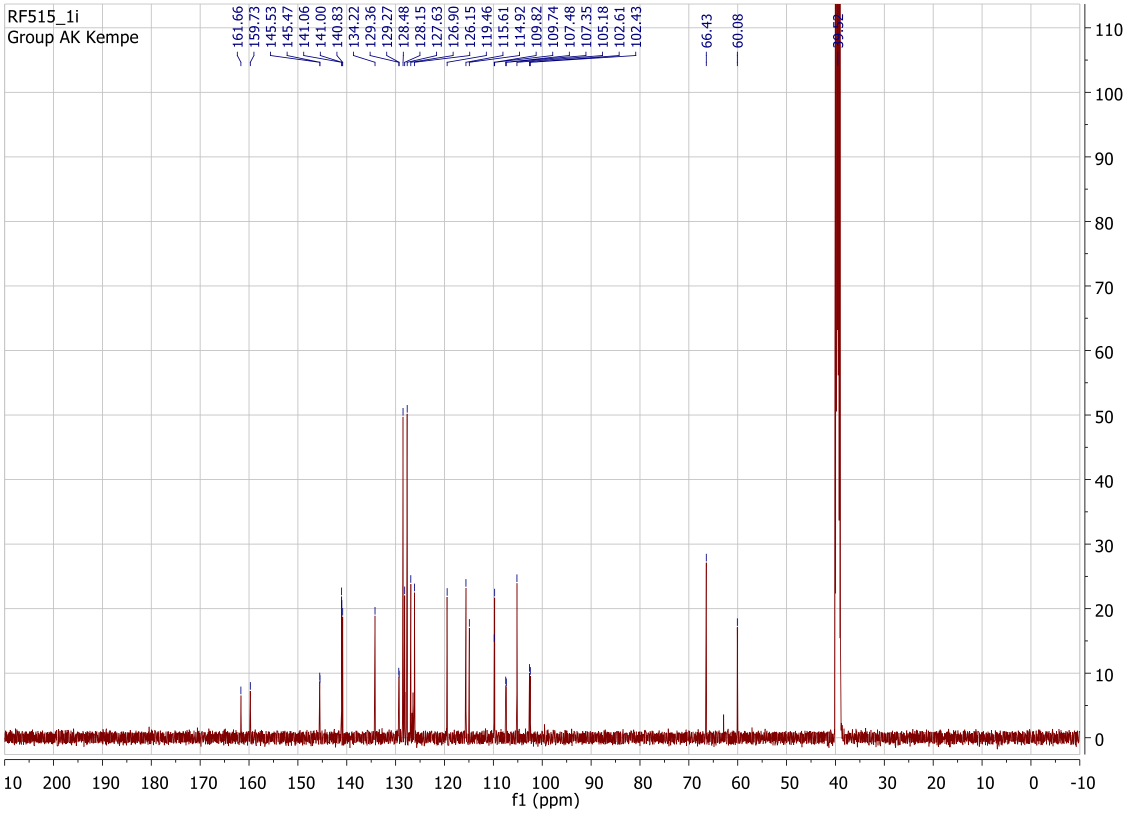

Supplementary Figure 101 ^{13}C NMR spectrum of compound **B1f**. (125 MHz, 293 K, DMSO-d_6).

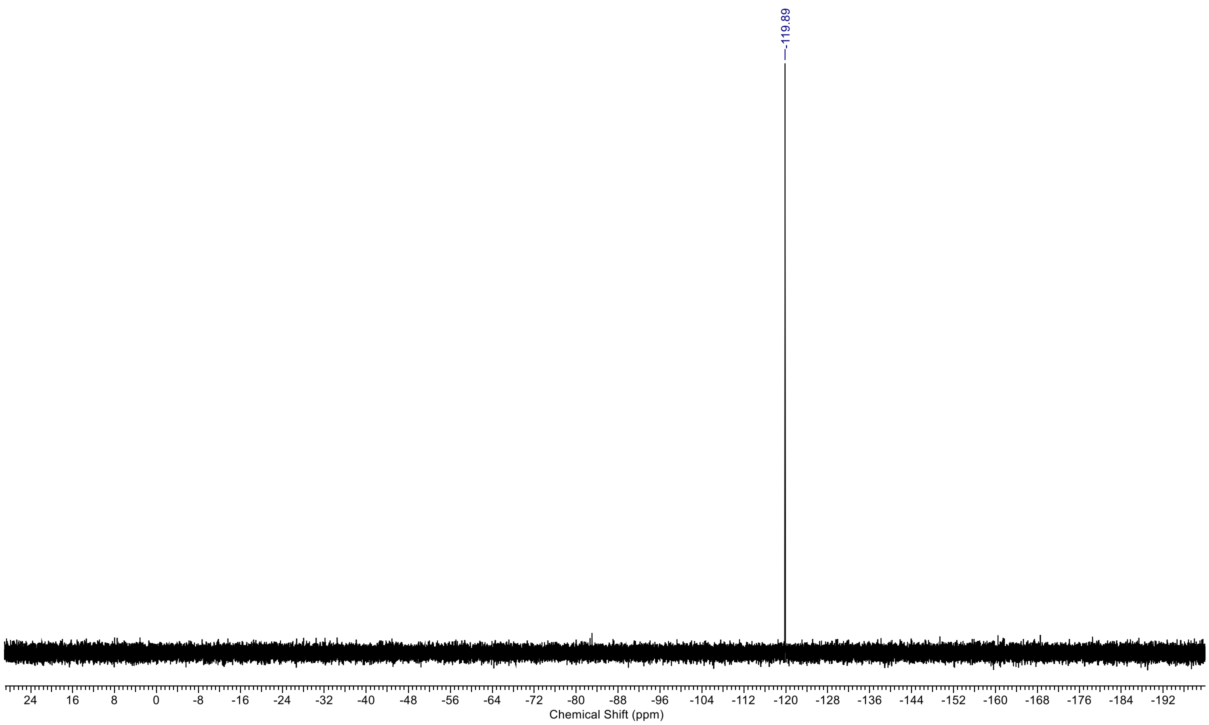

Supplementary Figure 102 ^{19}F NMR spectrum of compound **B1f**. (376 MHz, 293 K, DMSO-d_6).

NMR spectra of B1g

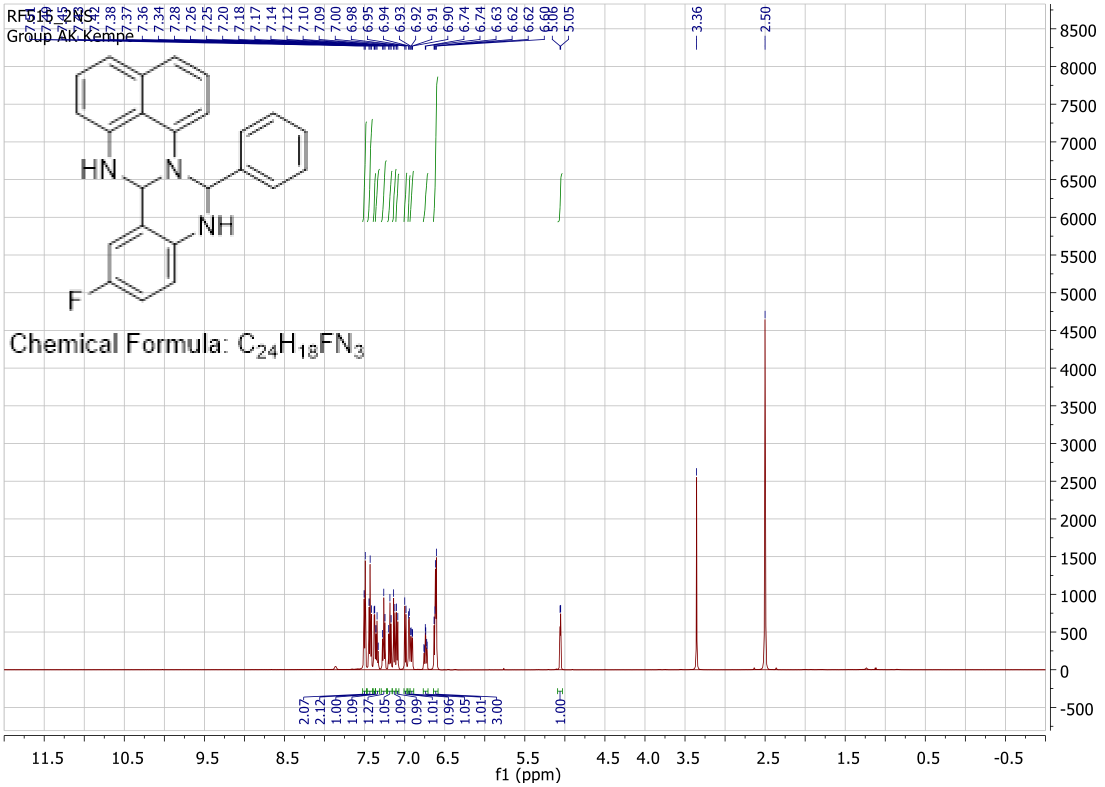

Supplementary Figure 103 ^{1}H NMR spectrum of compound **B1g**. (500 MHz, 293 K, DMSO-d_6).

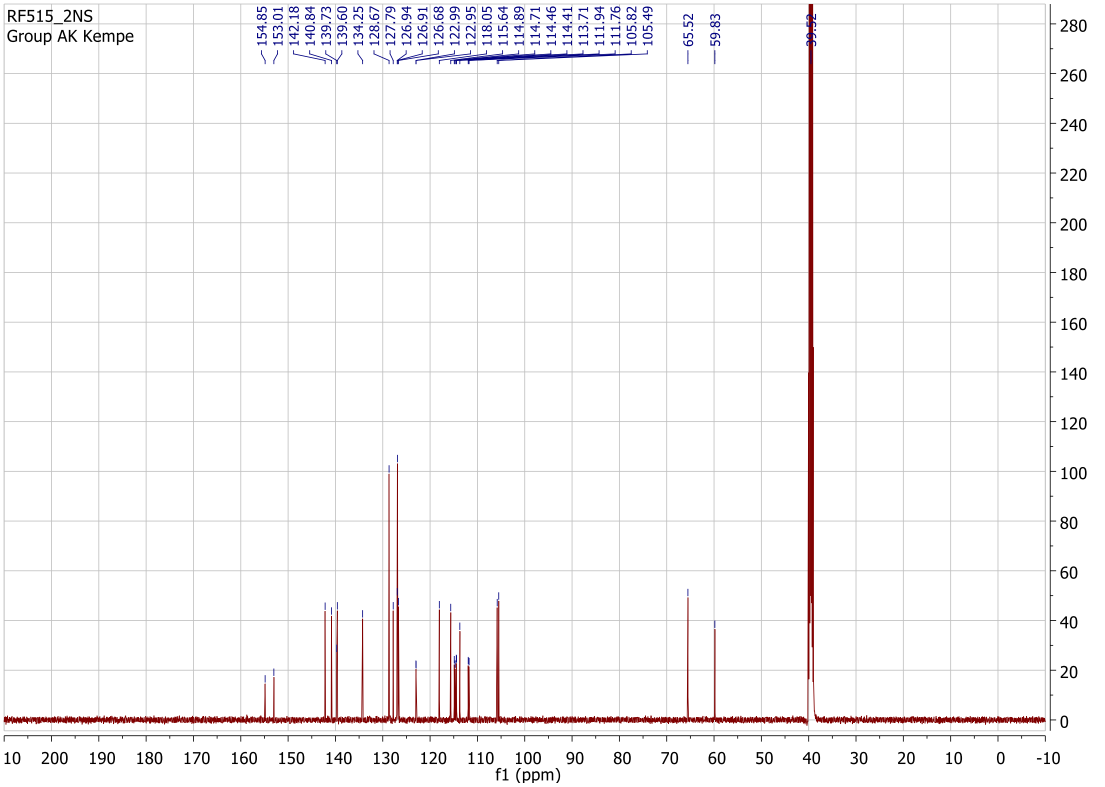

Supplementary Figure 104 ^{13}C NMR spectrum of compound **B1g**. (125 MHz, 293 K, DMSO-d_6).

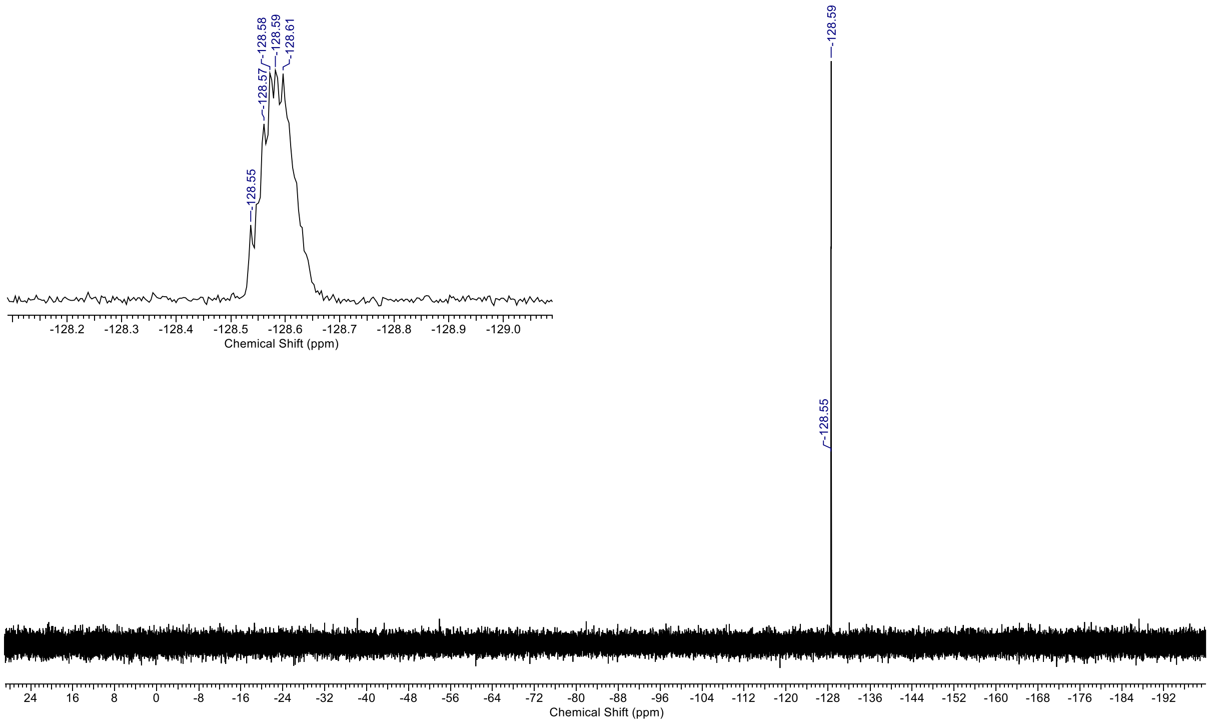

Supplementary Figure 105 ^{19}F NMR spectrum of compound **B1g**. (376 MHz, 293 K, DMSO-d_6).

NMR spectra of B1h

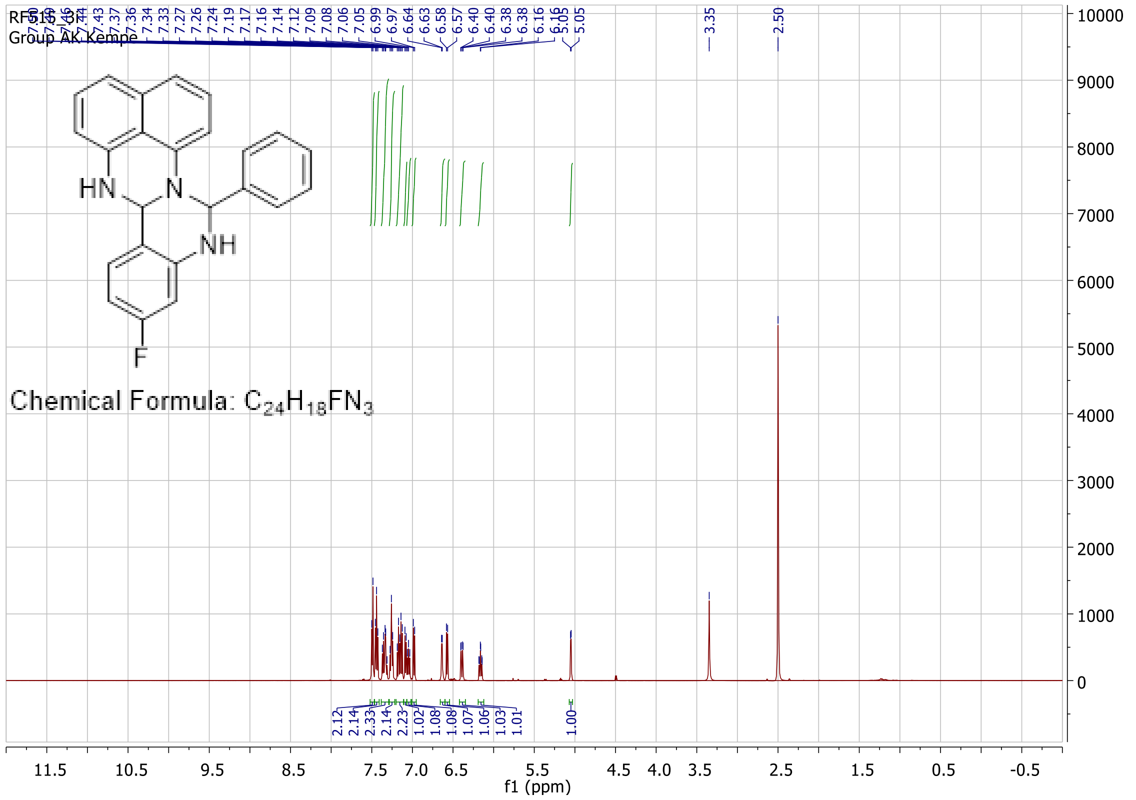

Supplementary Figure 106 ^{1}H NMR spectrum of compound **B1h**. (500 MHz, 293 K, DMSO-d$_6$).

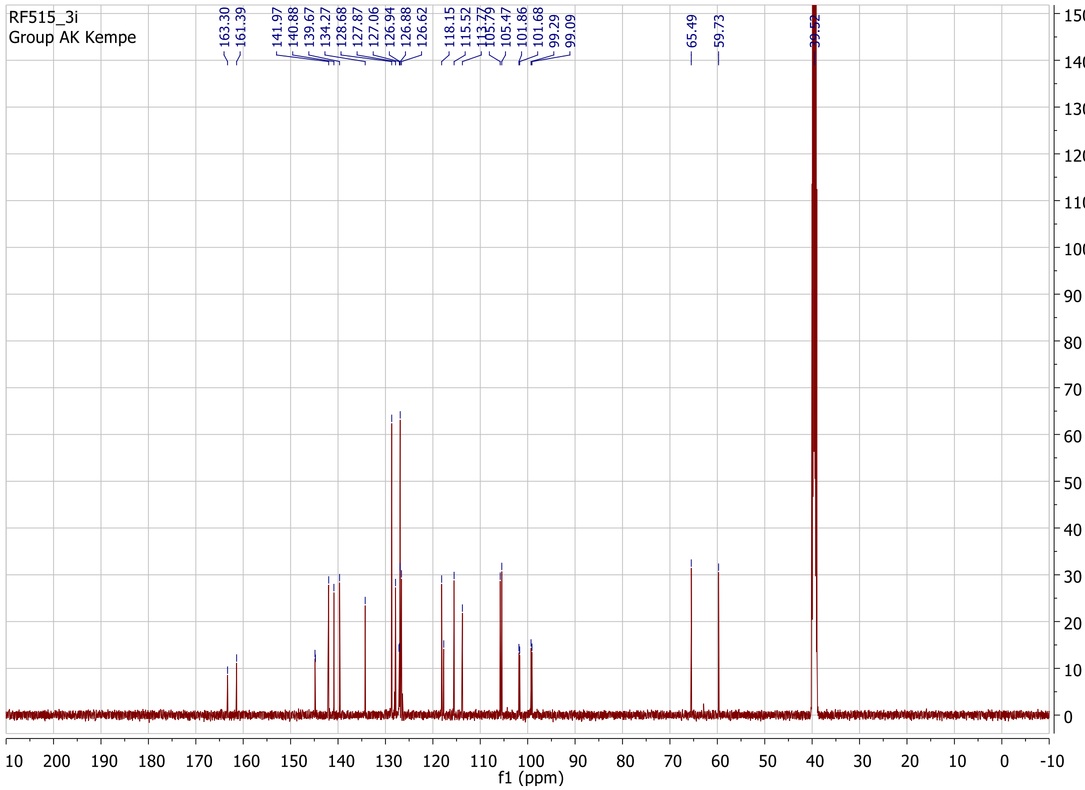

Supplementary Figure 107 ^{13}C NMR spectrum of compound **B1h**. (125 MHz, 293 K, DMSO-d$_6$).

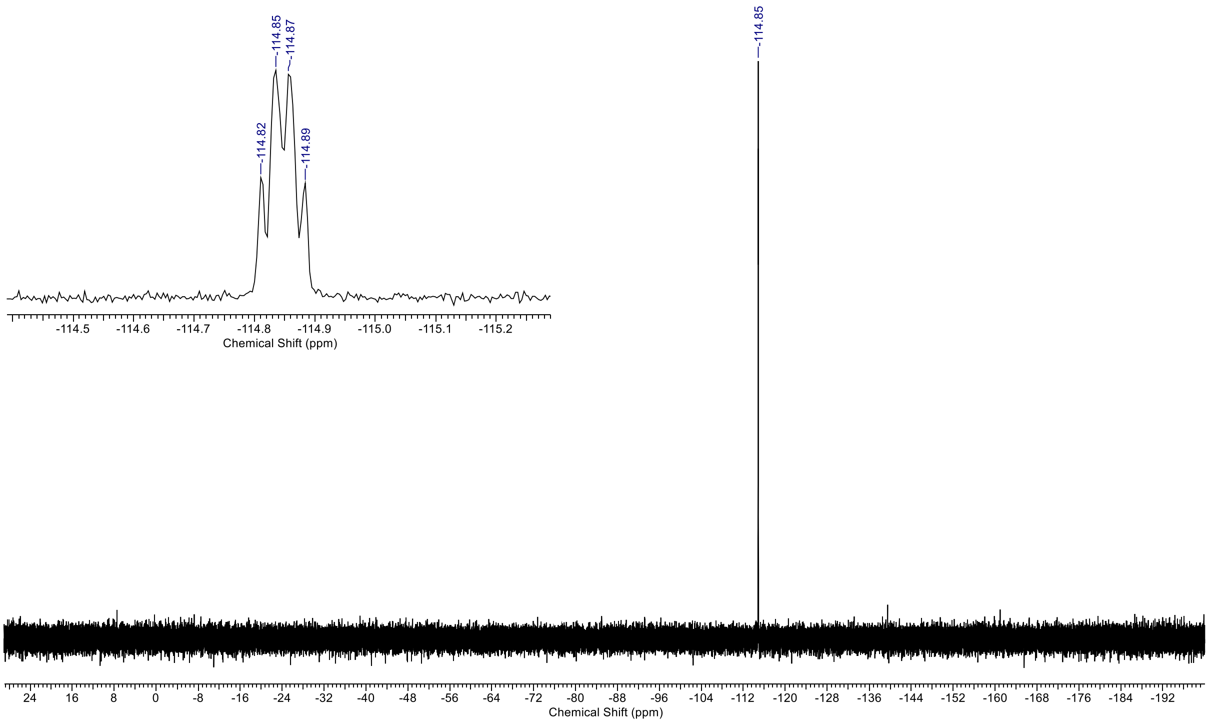

Supplementary Figure 108 ^{19}F NMR spectrum of compound **B1h**. (376 MHz, 293 K, DMSO-d_6).

NMR spectra of B1i

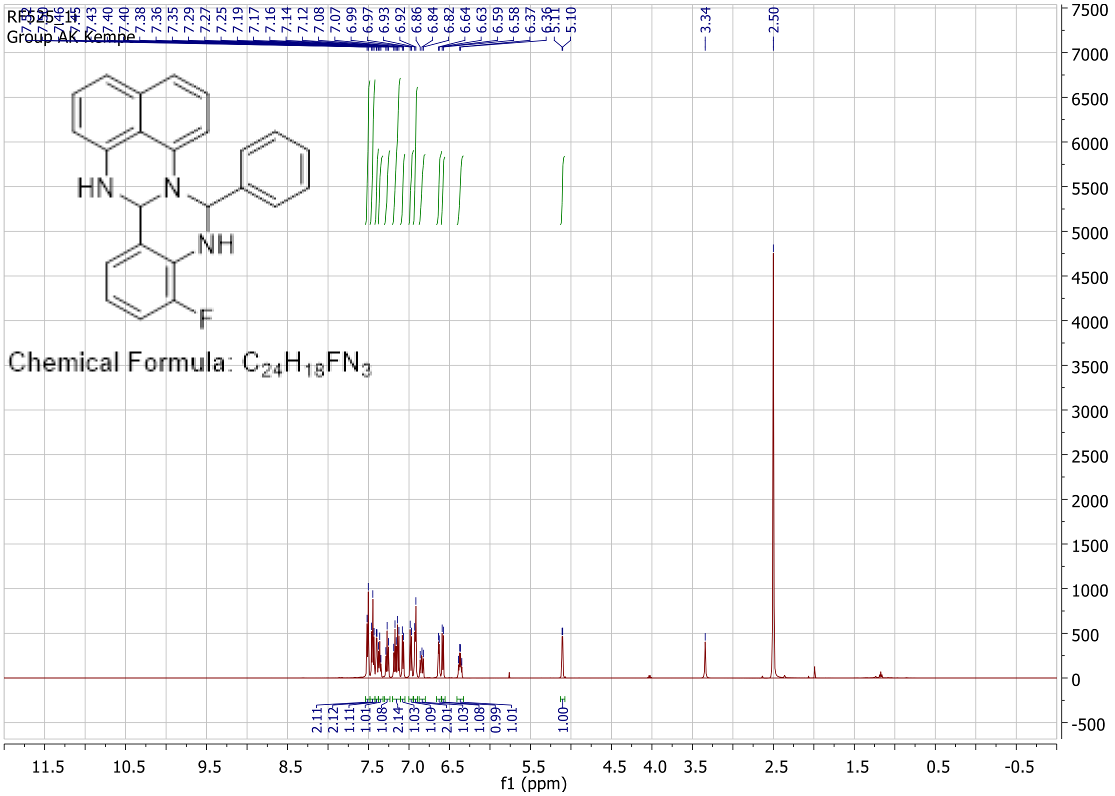

Supplementary Figure 109 ^{1}H NMR spectrum of compound **B1i**. (500 MHz, 293 K, DMSO-d_6).

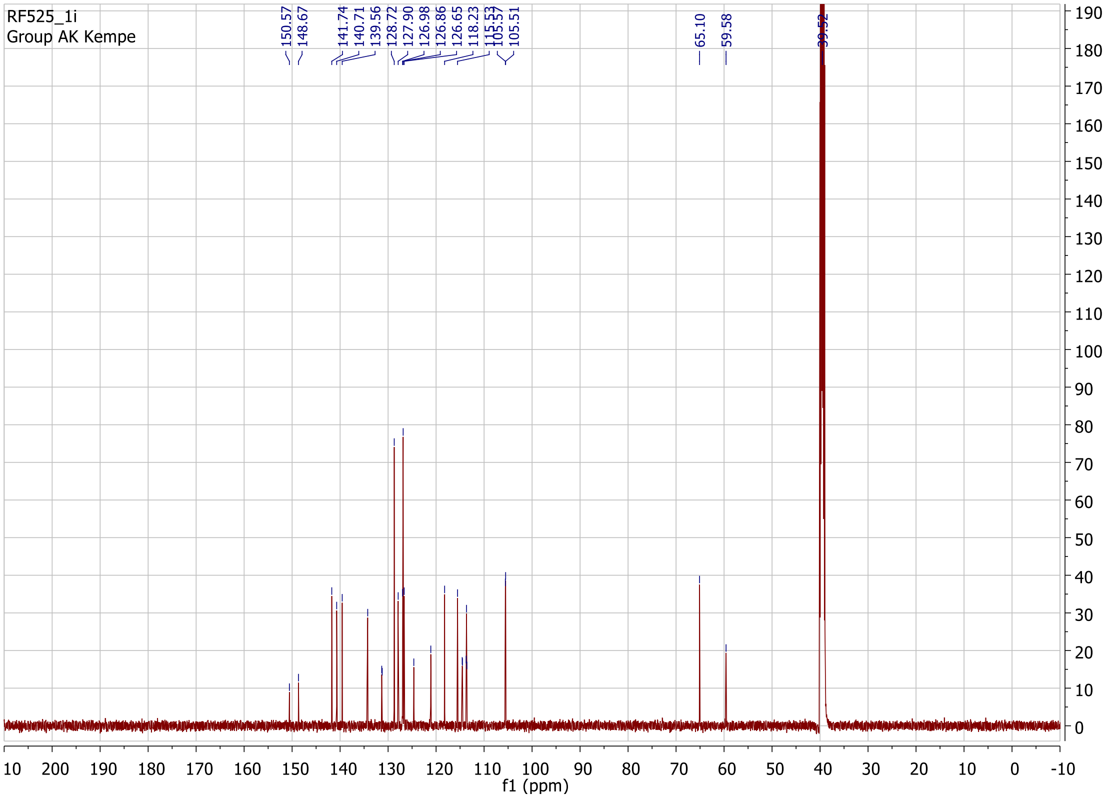

Supplementary Figure 110 ^{13}C NMR spectrum of compound **B1i**. (125 MHz, 293 K, DMSO-d_6).

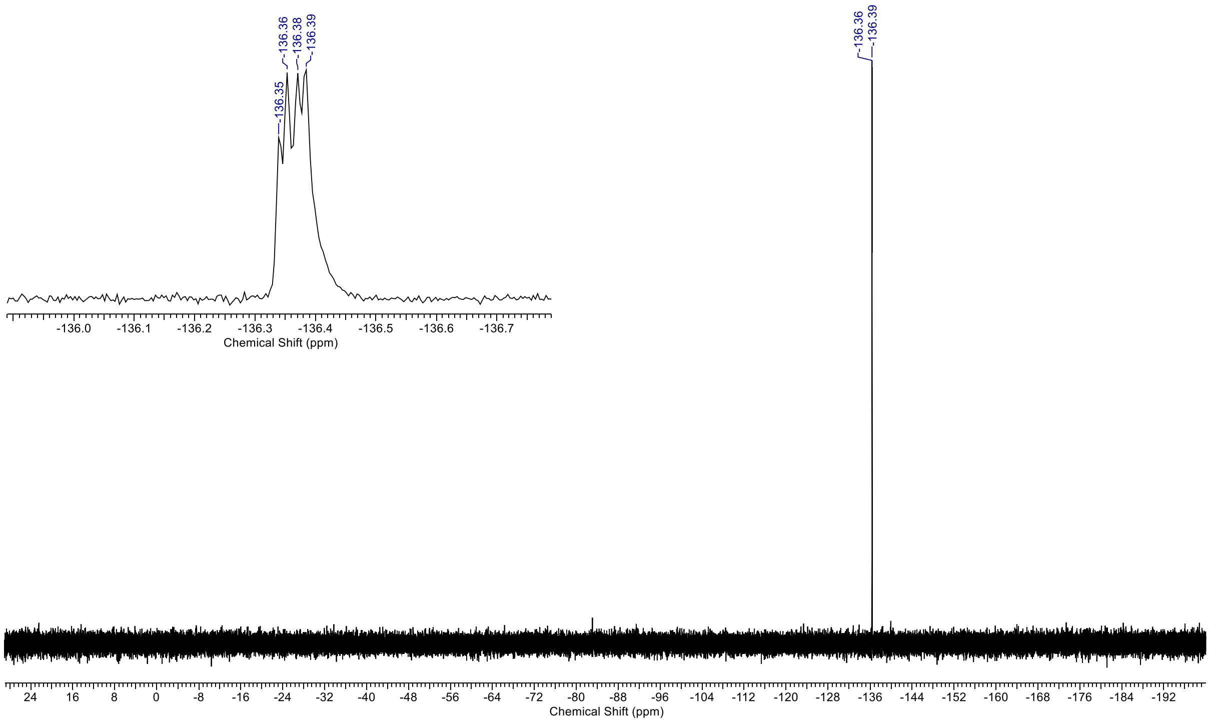

Supplementary Figure 111 ^{19}F NMR spectrum of compound **B1i**. (376 MHz, 293 K, DMSO-d_6).

NMR spectra of B1j

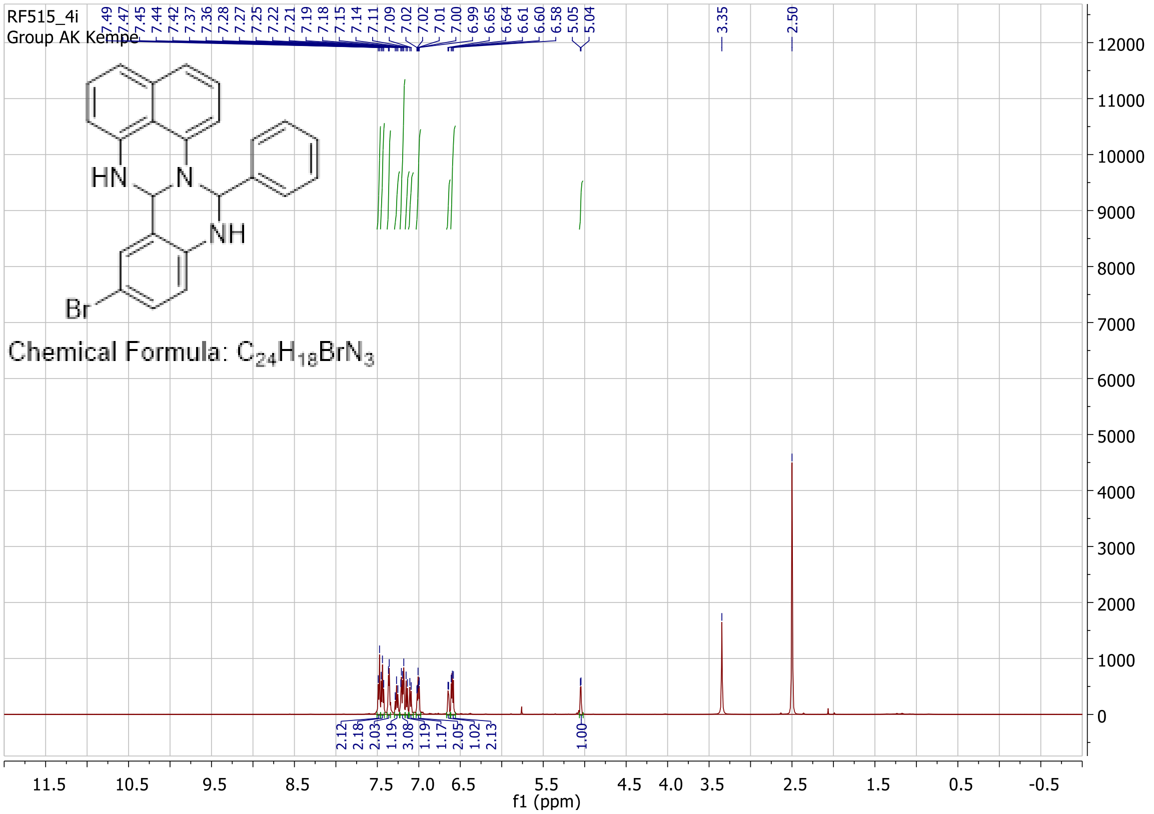

Supplementary Figure 112 ^{1}H NMR spectrum of compound **B1j**. (500 MHz, 293 K, DMSO-d_6).

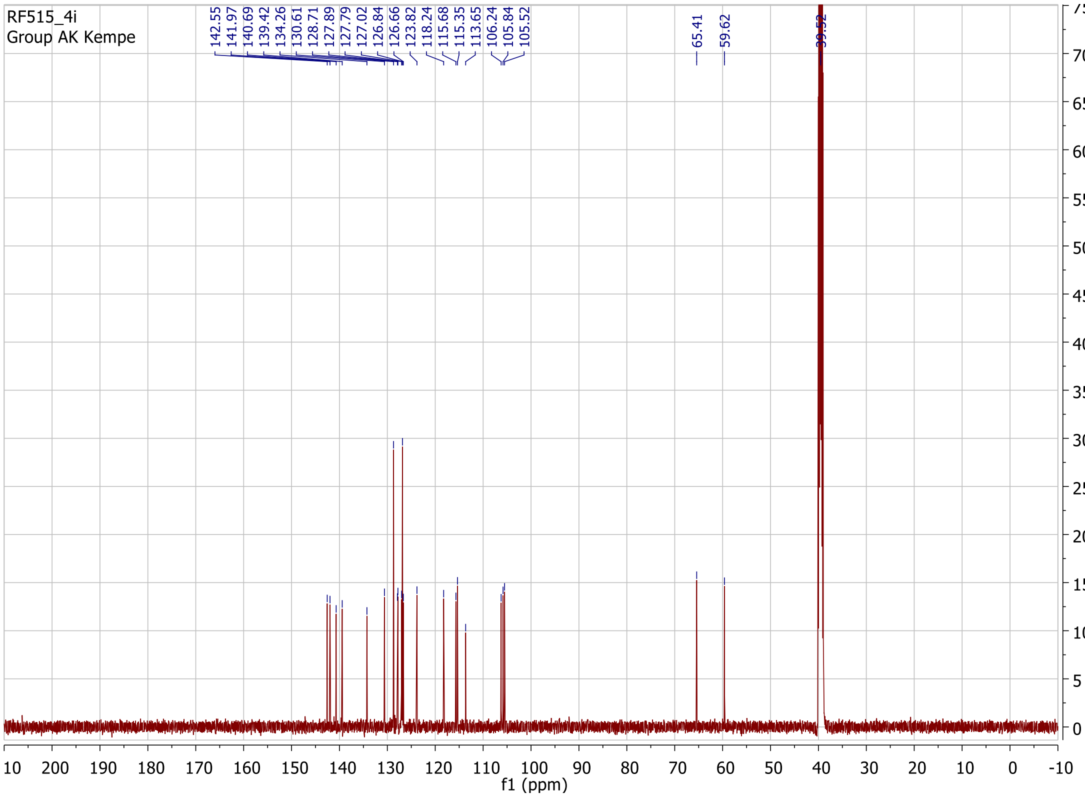

Supplementary Figure 113 ^{13}C NMR spectrum of compound **B1j**. (125 MHz, 293 K, DMSO-d_6).

NMR spectra of B1k

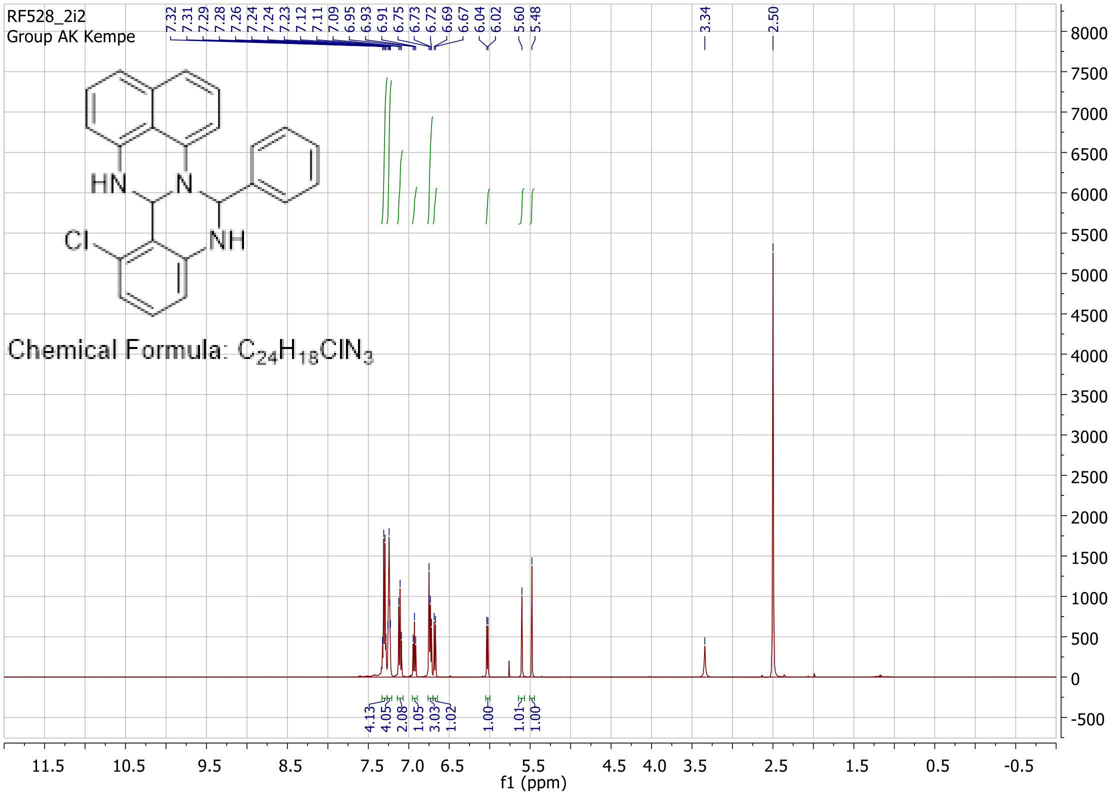

Supplementary Figure 114 ^{1}H NMR spectrum of compound **B1k**. (500 MHz, 293 K, DMSO-d_6).

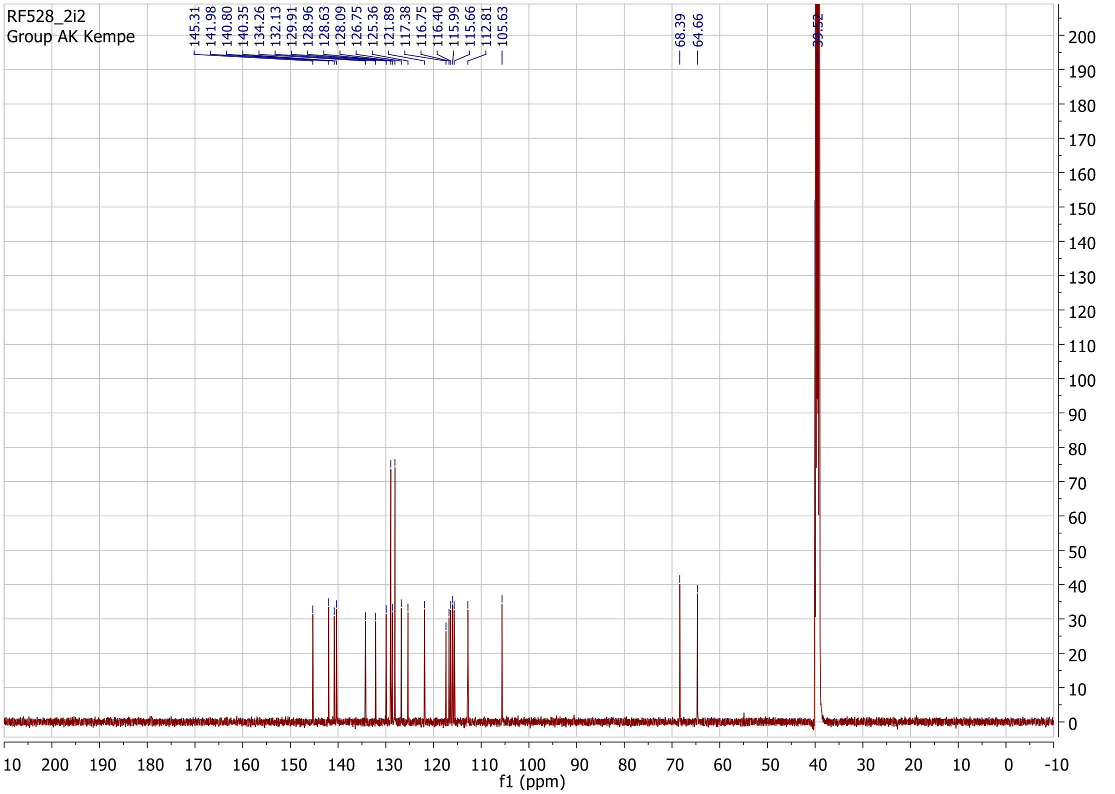

Supplementary Figure 115 ^{13}C NMR spectrum of compound **B1k**. (125 MHz, 293 K, DMSO-d_6).

NMR spectra of B1l

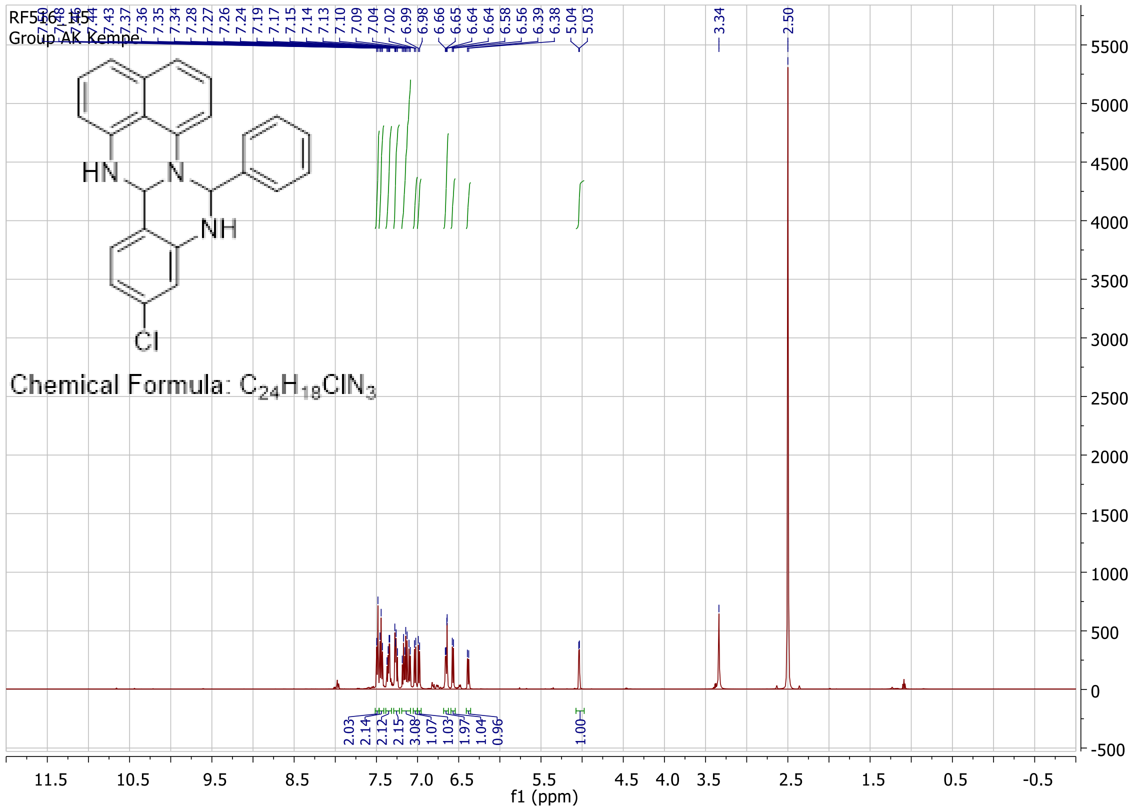

Supplementary Figure 116 ^{1}H NMR spectrum of compound **B1l**. (500 MHz, 293 K, DMSO-d_6).

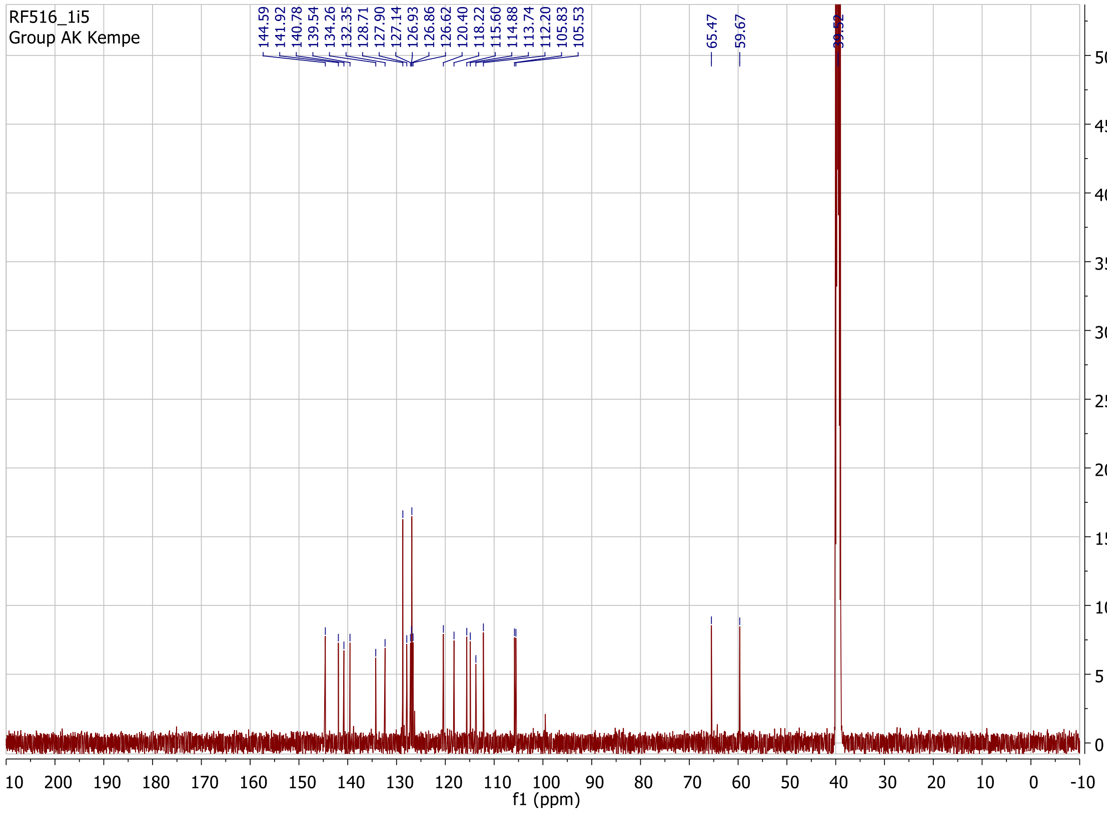

Supplementary Figure 117 ^{13}C NMR spectrum of compound **B1l**. (125 MHz, 293 K, DMSO-d_6).

NMR spectra of B1m

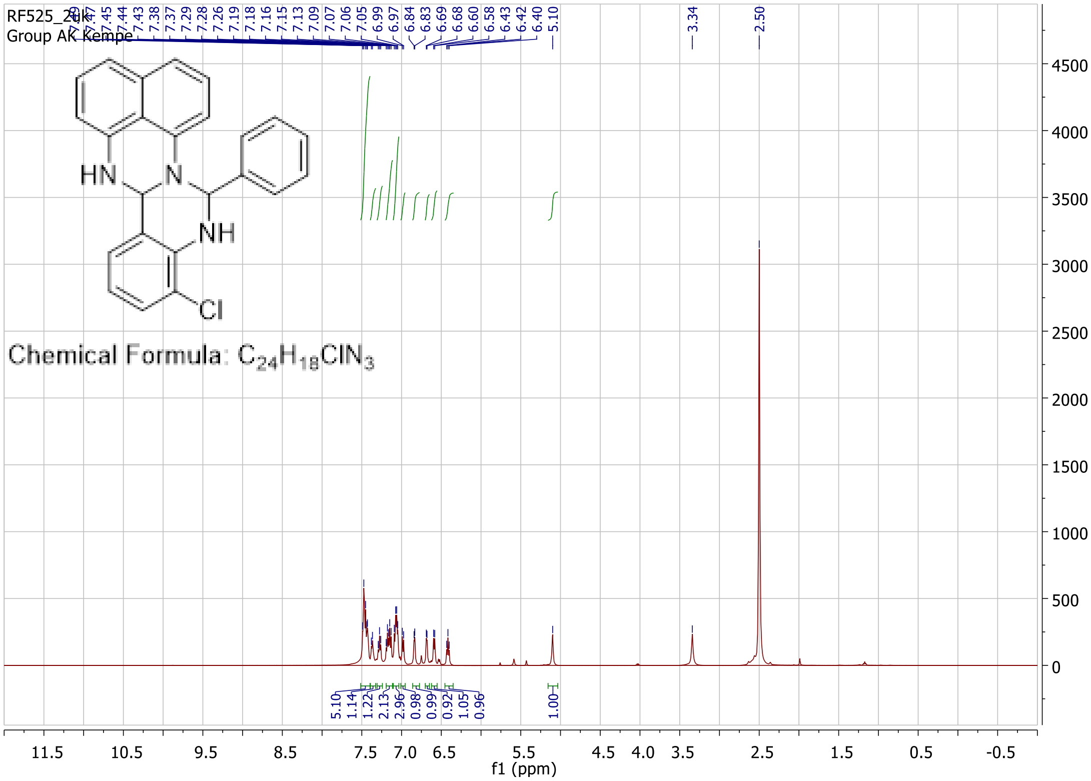

Supplementary Figure 118 ^{1}H NMR spectrum of compound **B1m**. (500 MHz, 293 K, DMSO-d_6).

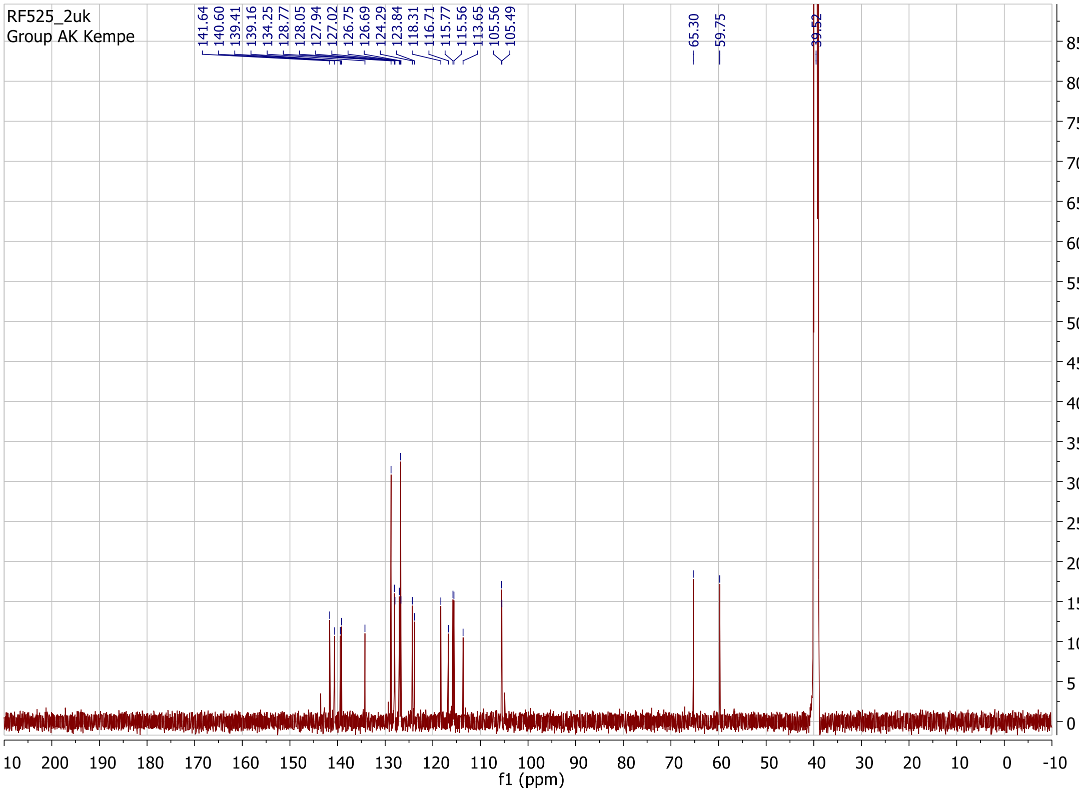

Supplementary Figure 119 ^{13}C NMR spectrum of compound **B1m**. (125 MHz, 293 K, DMSO-d_6).

NMR spectra of B1n

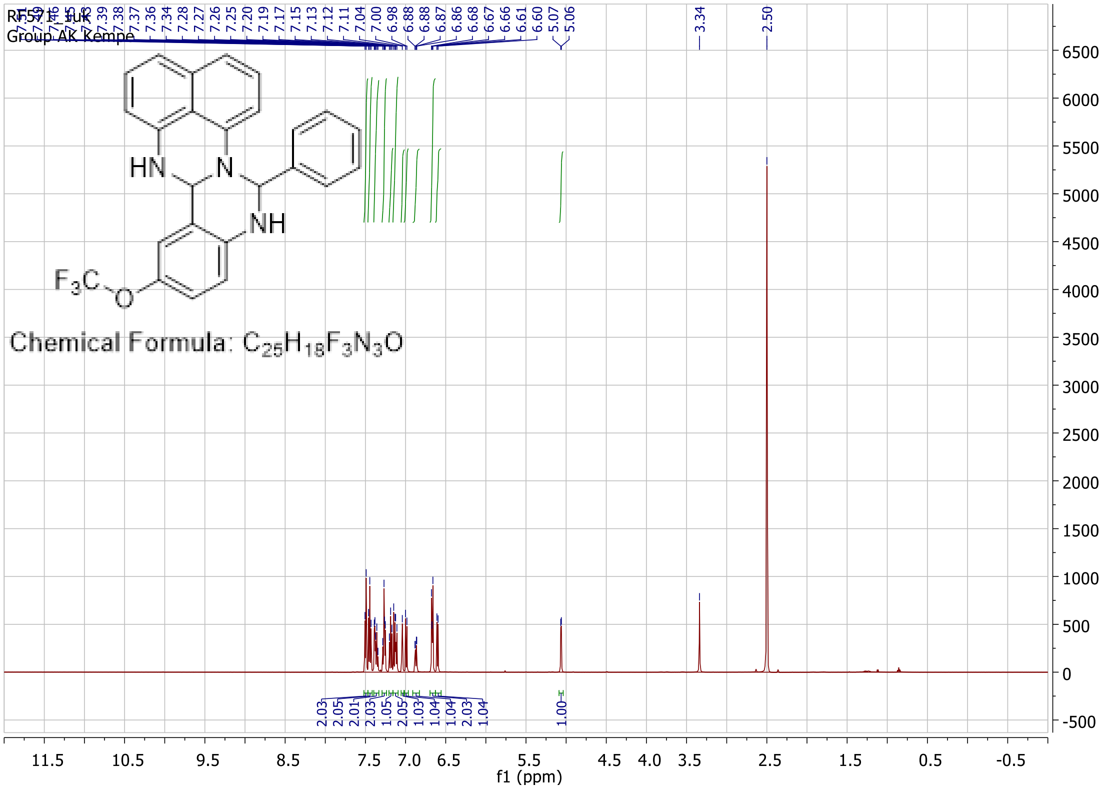

Supplementary Figure 120 ^{1}H NMR spectrum of compound **B1n**. (500 MHz, 293 K, DMSO-d_6).

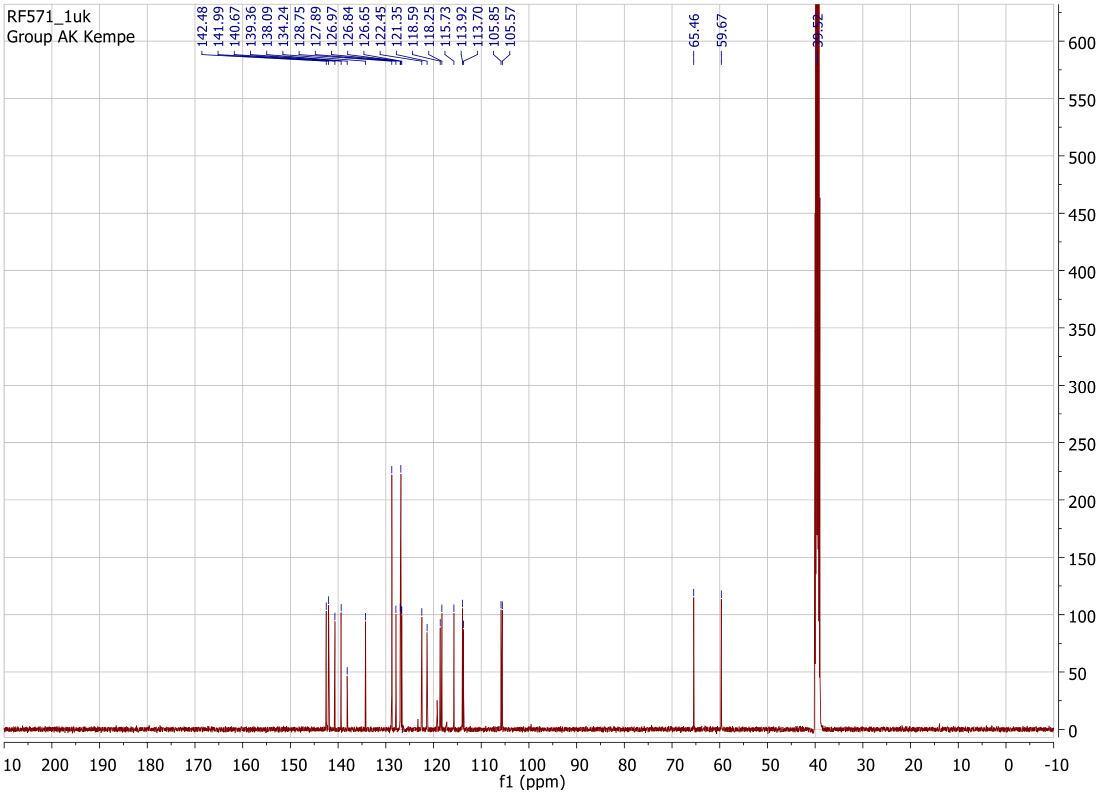

Supplementary Figure 121 ^{13}C NMR spectrum of compound **B1n**. (125 MHz, 293 K, DMSO-d_6).

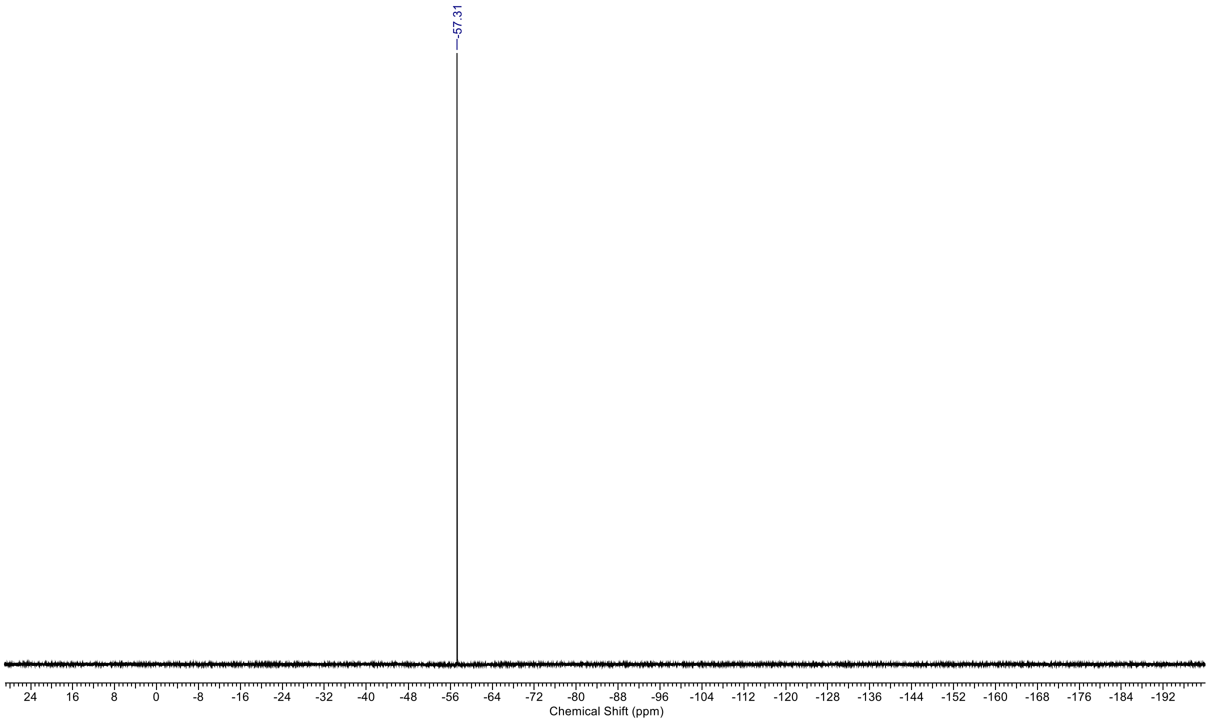

Supplementary Figure 122 ^{19}F NMR spectrum of compound **B1n**. (125 MHz, 293 K, DMSO-d_6).

NMR spectra of B1o

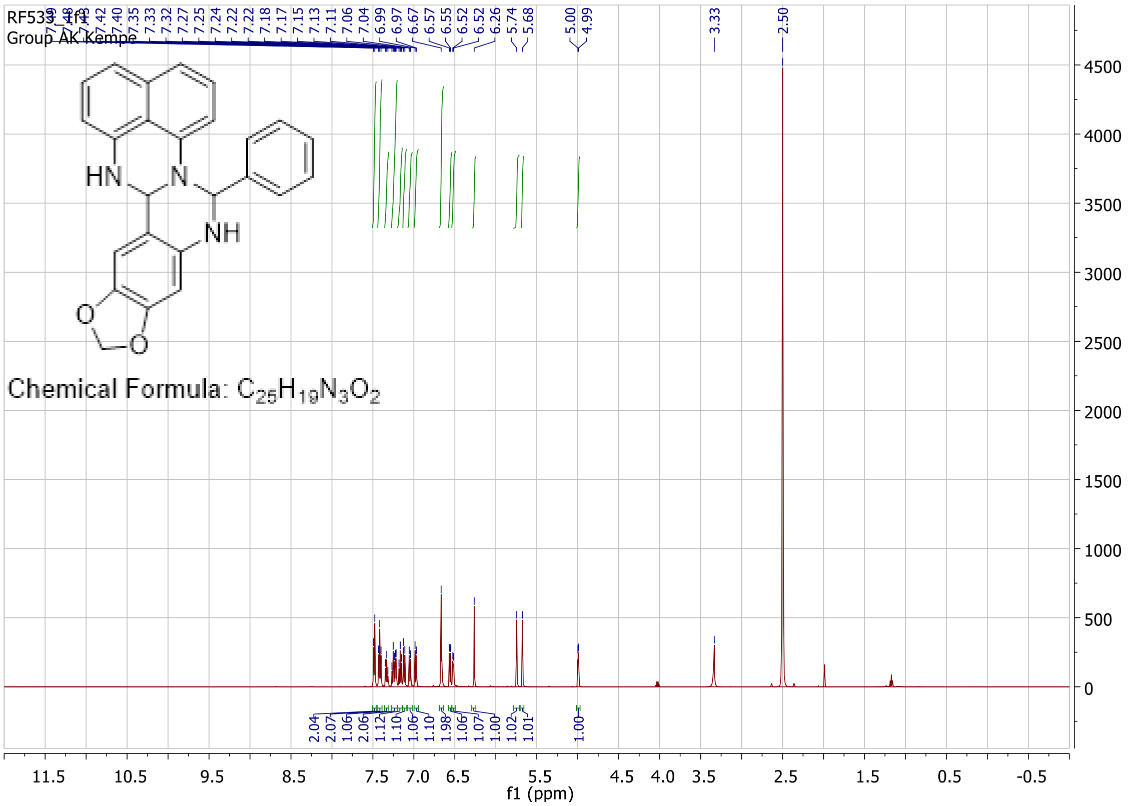

Supplementary Figure 123 ^{1}H NMR spectrum of compound **B1o**. (500 MHz, 293 K, DMSO-d$_6$).

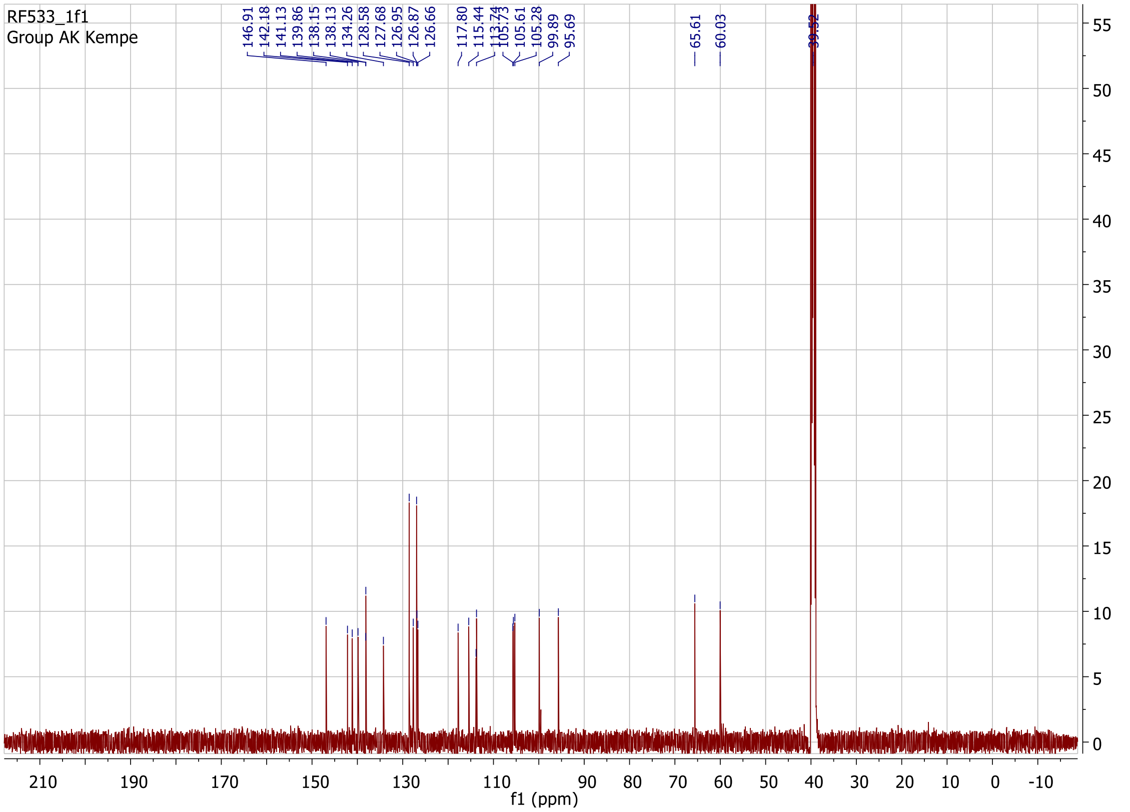

Supplementary Figure 124 ^{13}C NMR spectrum of compound **B1o**. (125 MHz, 293 K, DMSO-d$_6$).

NMR spectra of B1p

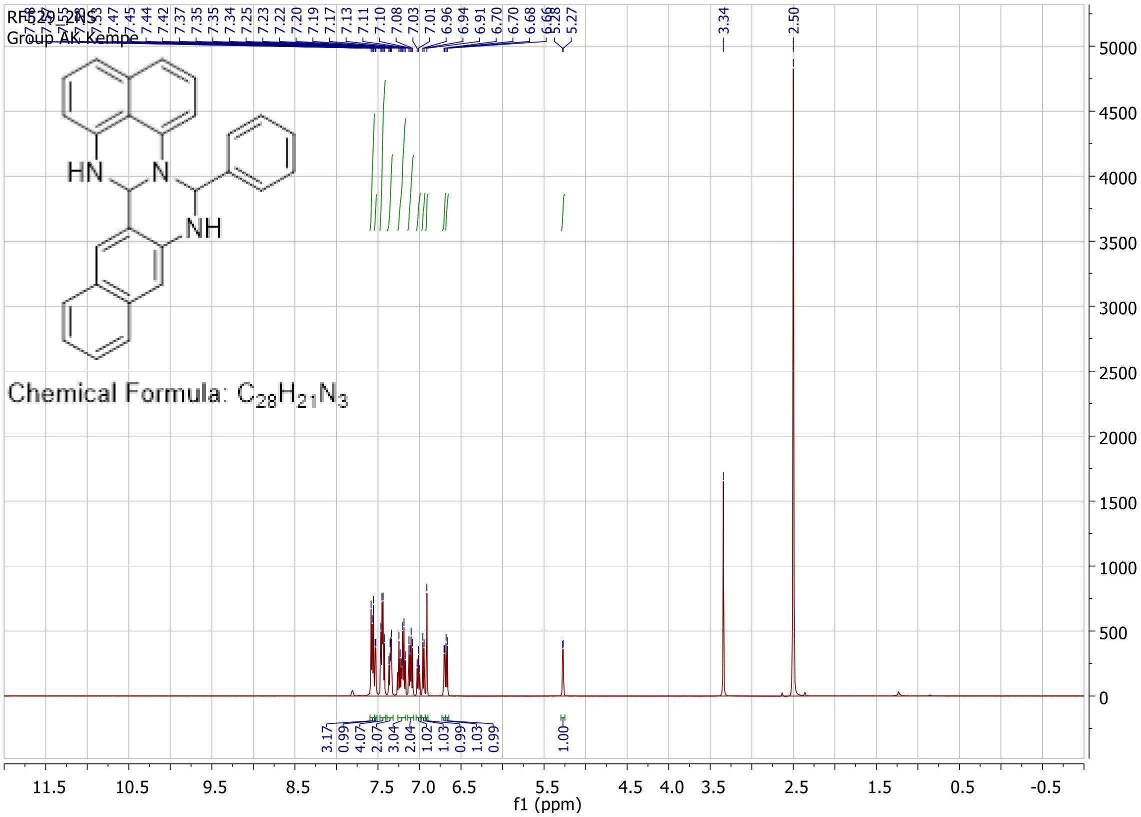

Supplementary Figure 125 ^{1}H NMR spectrum of compound **B1p**. (500 MHz, 293 K, DMSO-d_6).

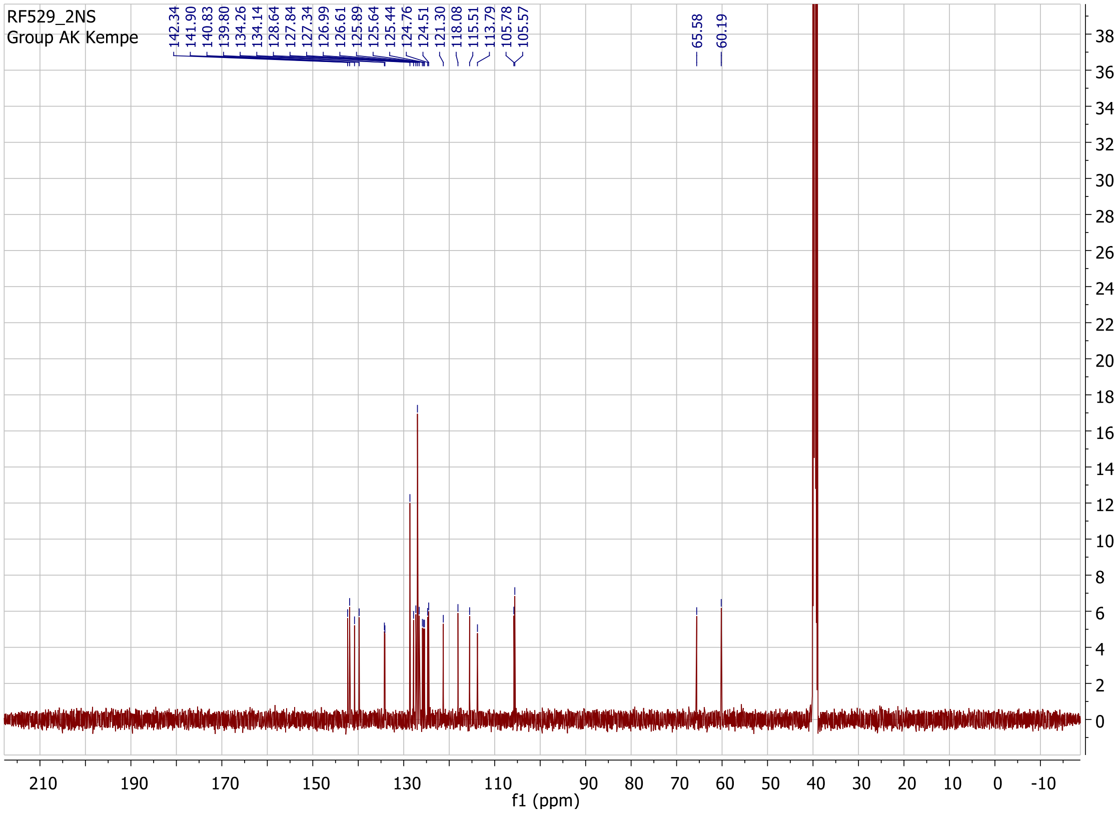

Supplementary Figure 126 ^{13}C NMR spectrum of compound **B1p**. (125 MHz, 293 K, DMSO-d_6).

NMR spectra of B1q

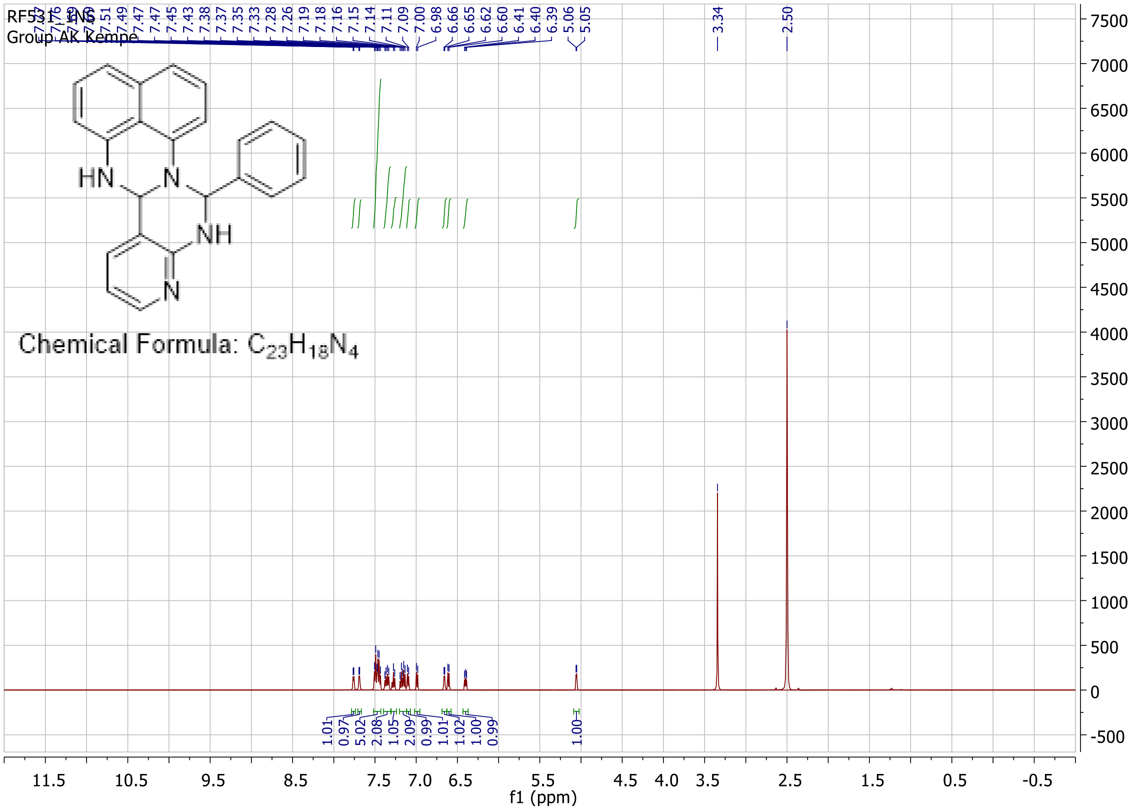

Supplementary Figure 127 ^{1}H NMR spectrum of compound **B1q**. (500 MHz, 293 K, DMSO-d$_6$).

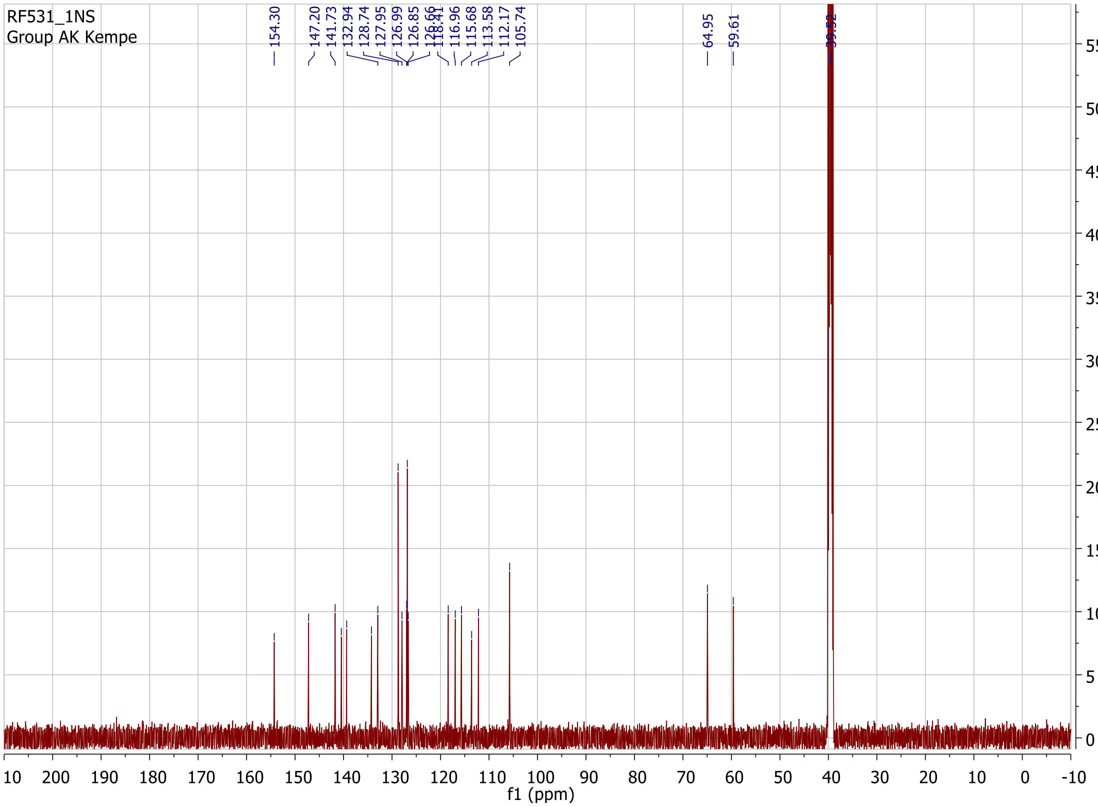

Supplementary Figure 128 ^{13}C NMR spectrum of compound **B1q**. (125 MHz, 293 K, DMSO-d$_6$).

NMR spectra of B2a

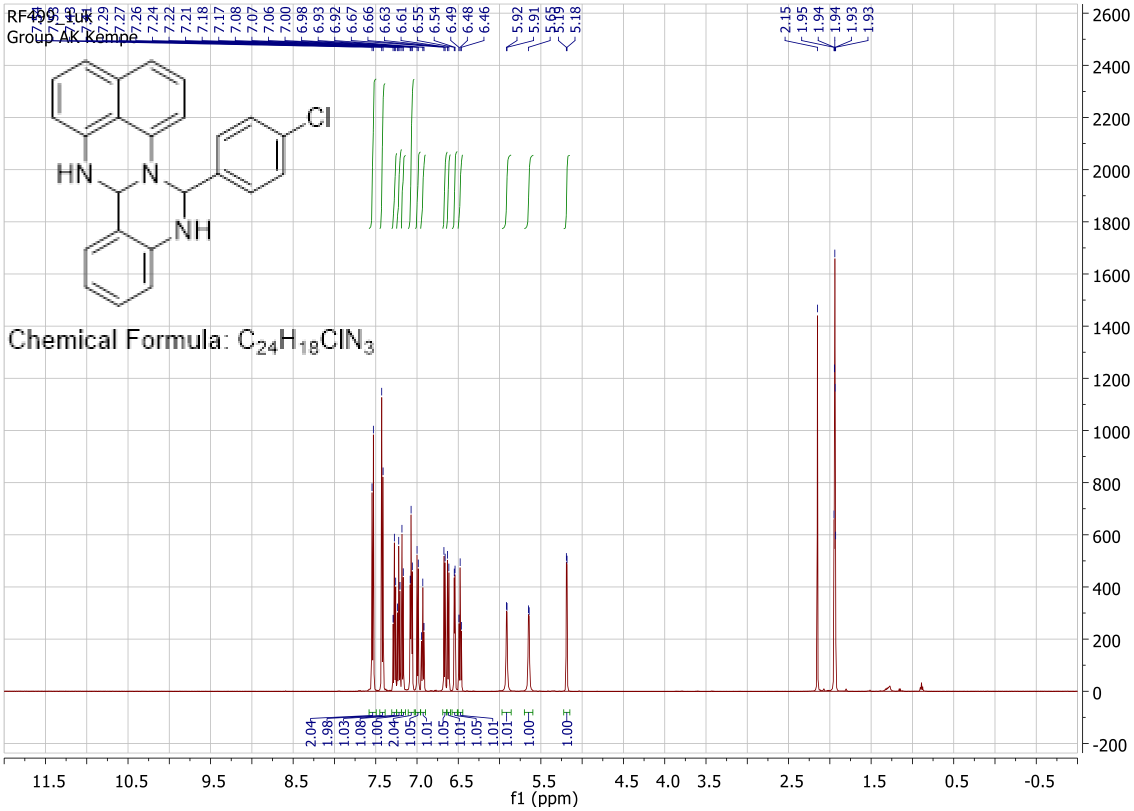

Supplementary Figure 129 ^{1}H NMR spectrum of compound **B2a**. (500 MHz, 293 K, DMSO-d_6).

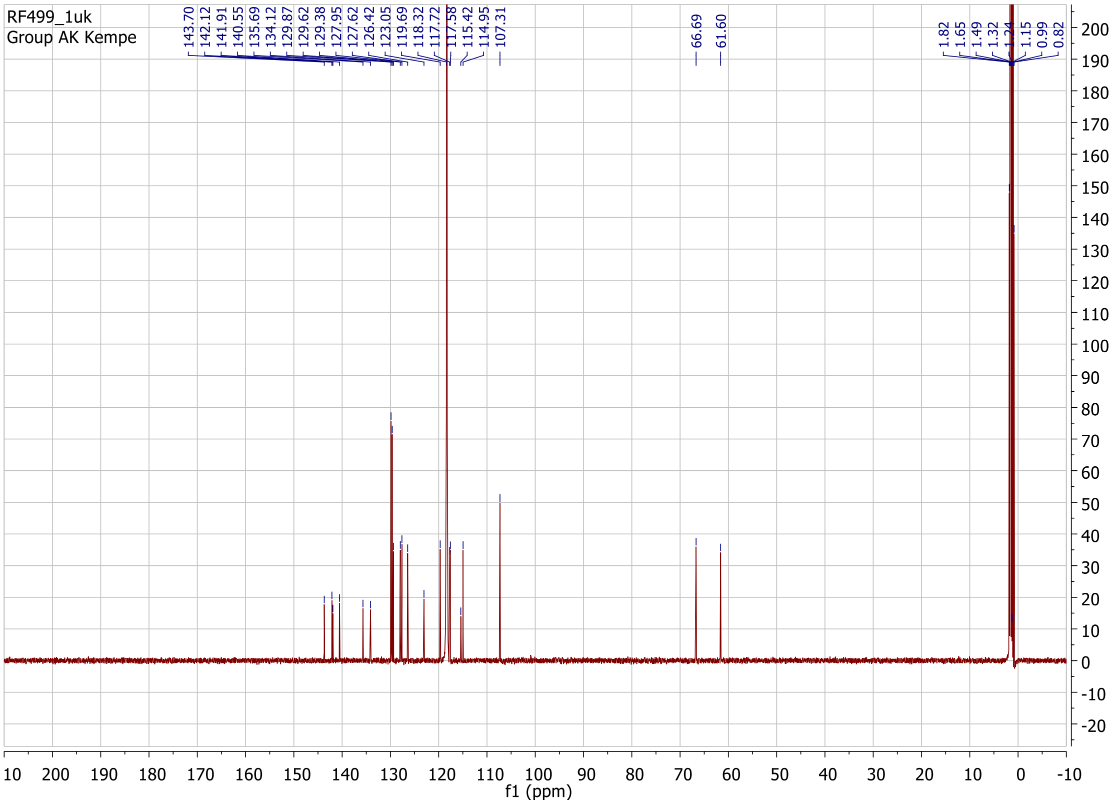

Supplementary Figure 130 ^{13}C NMR spectrum of compound **B2a**. (125 MHz, 293 K, DMSO-d_6).

NMR spectra of B2b

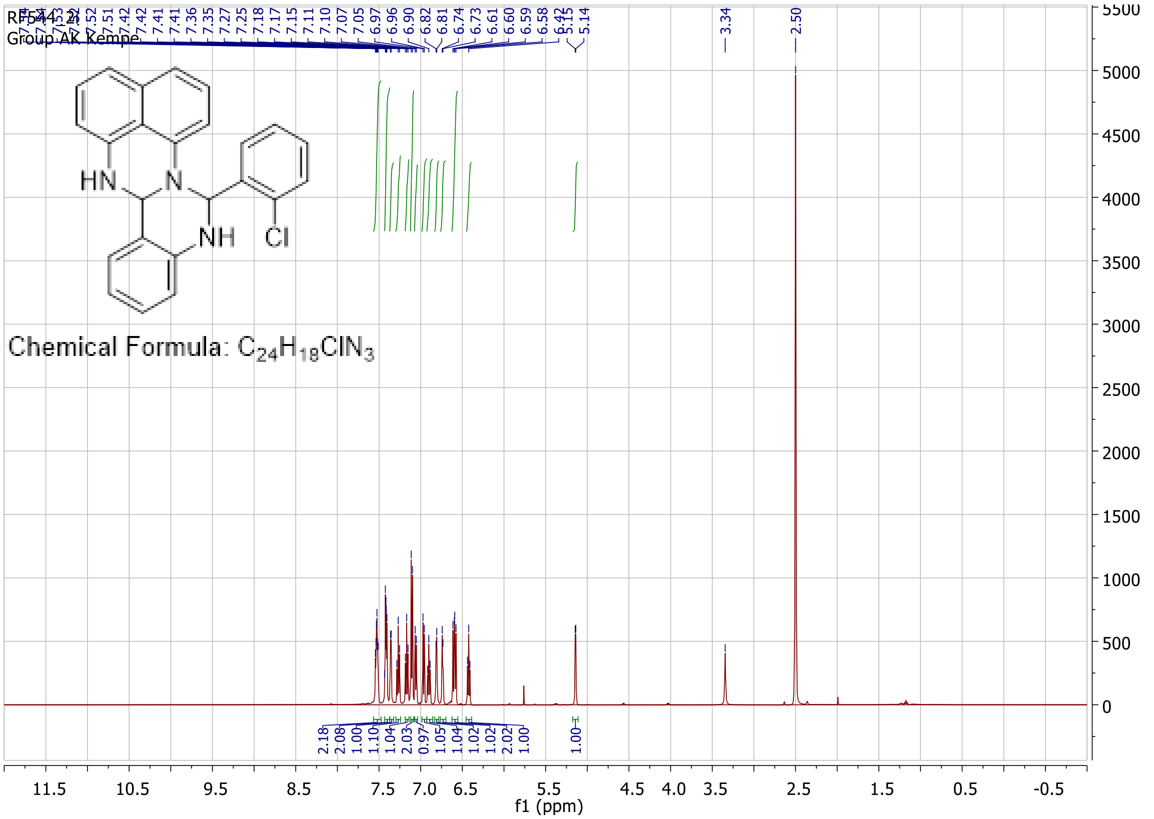

Supplementary Figure 131 ^{1}H NMR spectrum of compound **B2b**. (500 MHz, 293 K, DMSO-d_6).

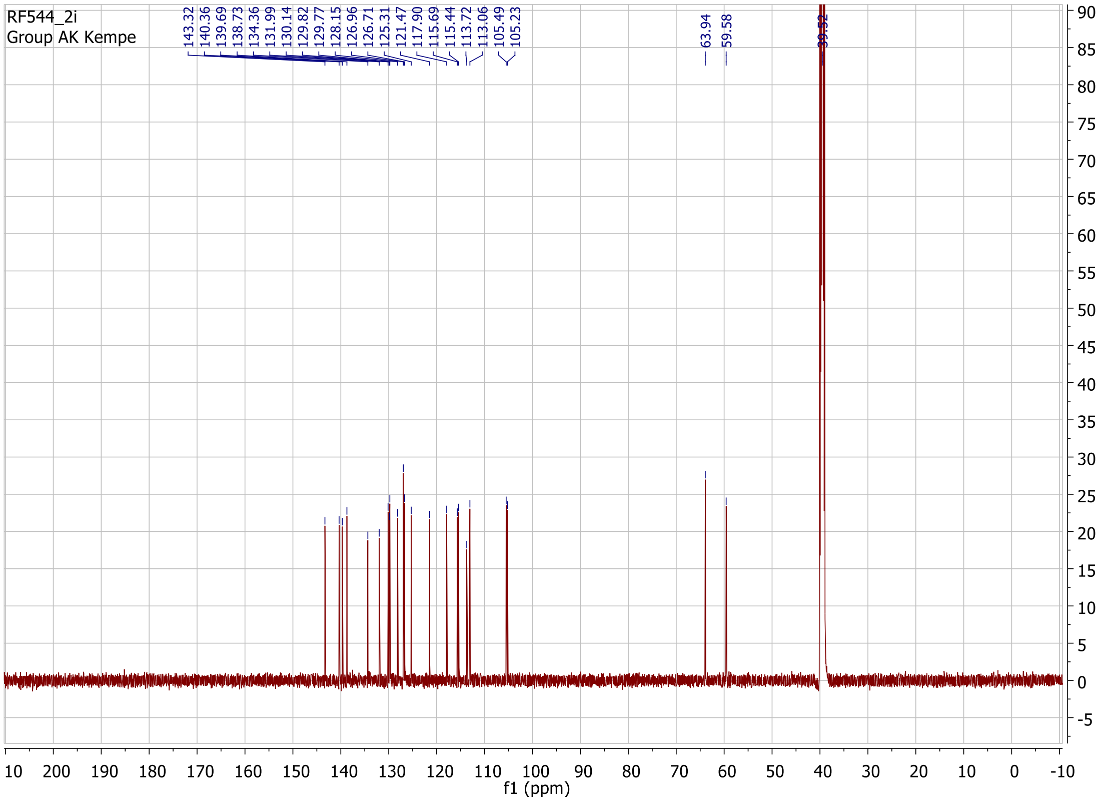

Supplementary Figure 132 ^{13}C NMR spectrum of compound **B2b**. (125 MHz, 293 K, DMSO-d_6).

NMR spectra of B2c

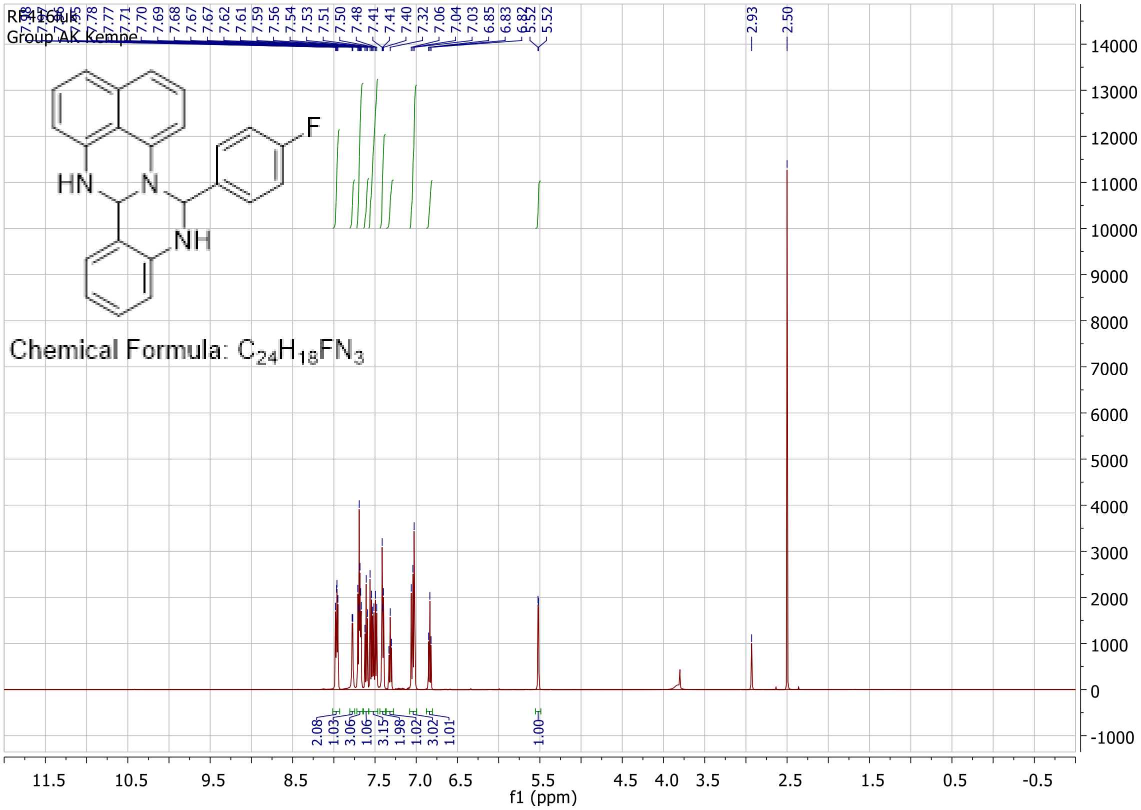

Supplementary Figure 133 ^{1}H NMR spectrum of compound **B2c**. (500 MHz, 293 K, DMSO-d$_6$).

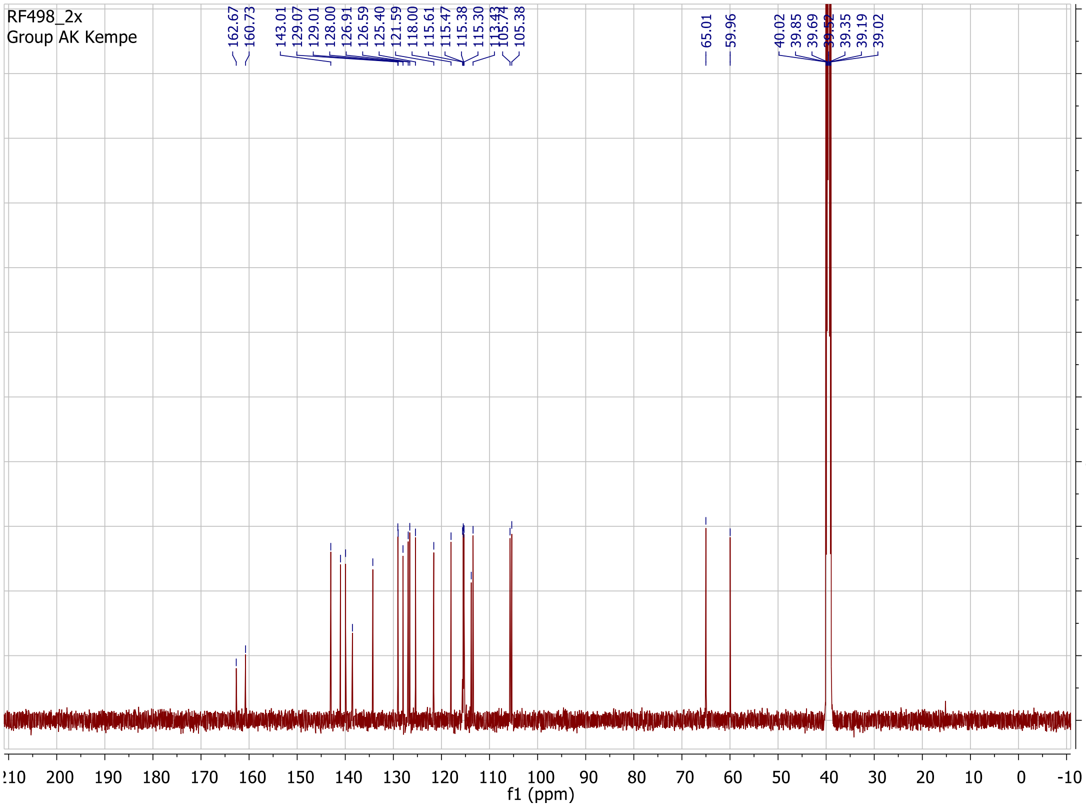

Supplementary Figure 134 ^{13}C NMR spectrum of compound **B2c**. (125 MHz, 293 K, DMSO-d$_6$).

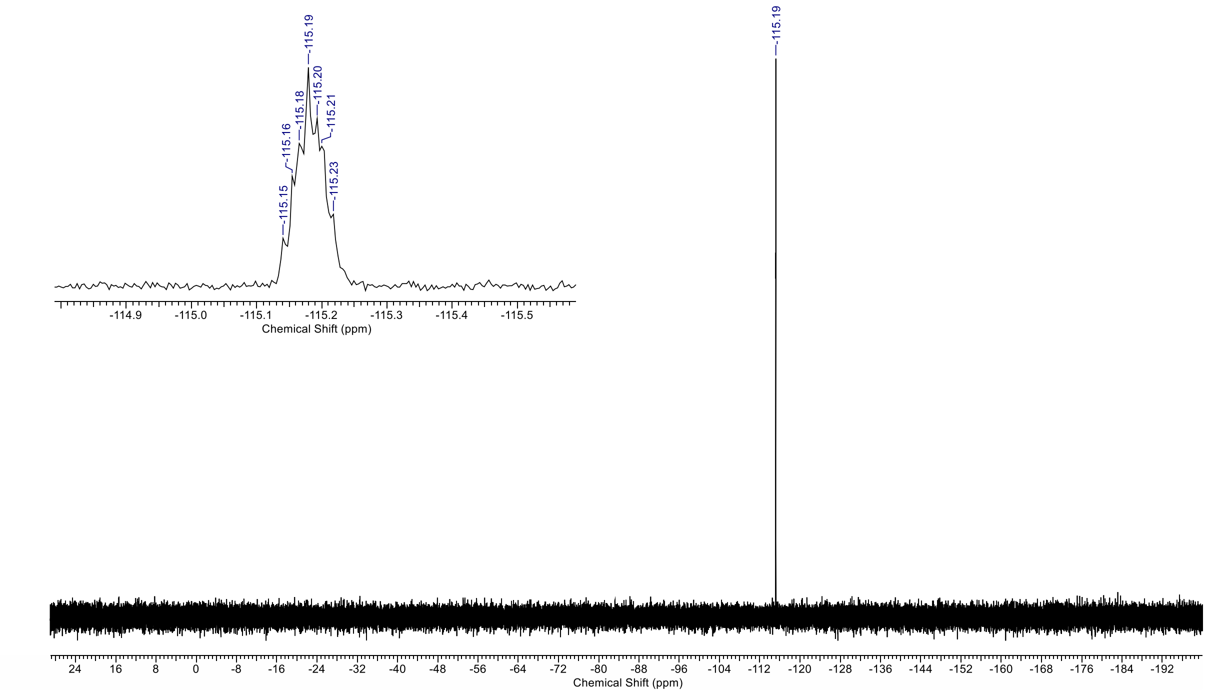

Supplementary Figure 135 ^{19}F NMR spectrum of compound **B2c**. (376 MHz, 293 K, DMSO-d_6).

NMR spectra of B2d

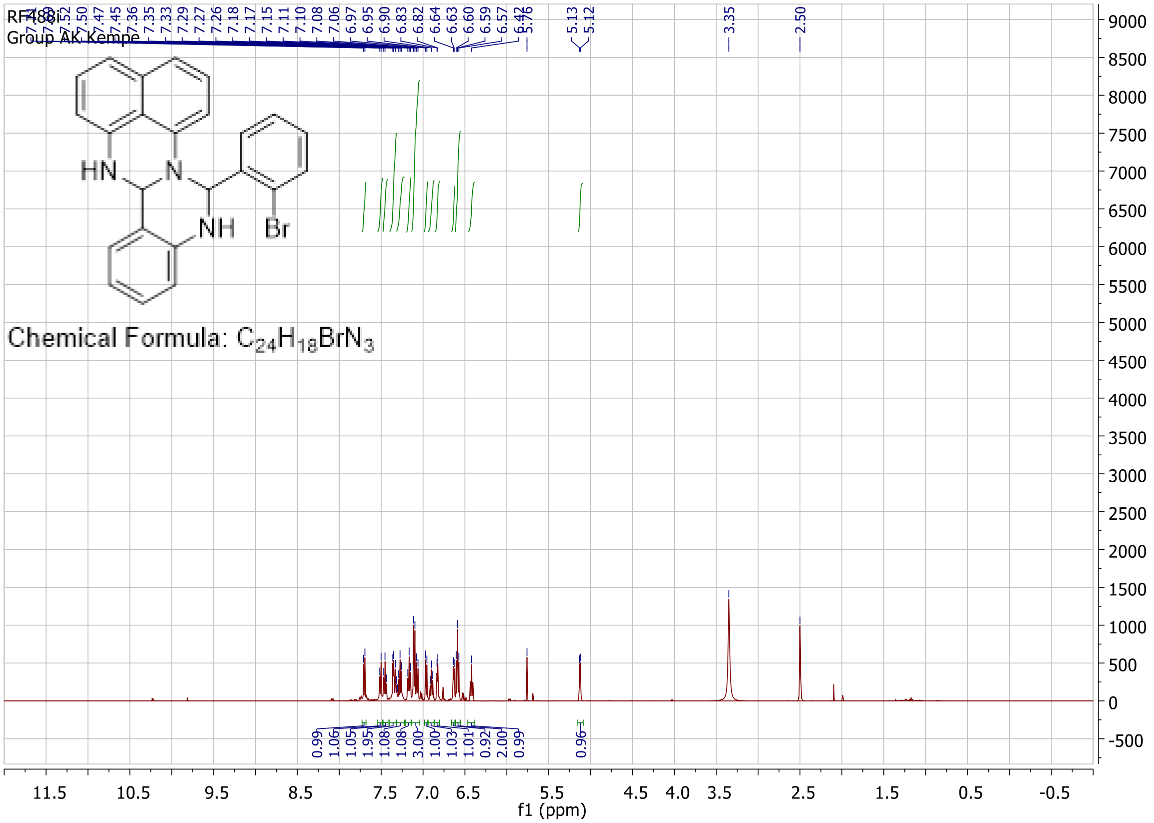

Supplementary Figure 136 ^{1}H NMR spectrum of compound **B2d**. (500 MHz, 293 K, DMSO-d$_6$).

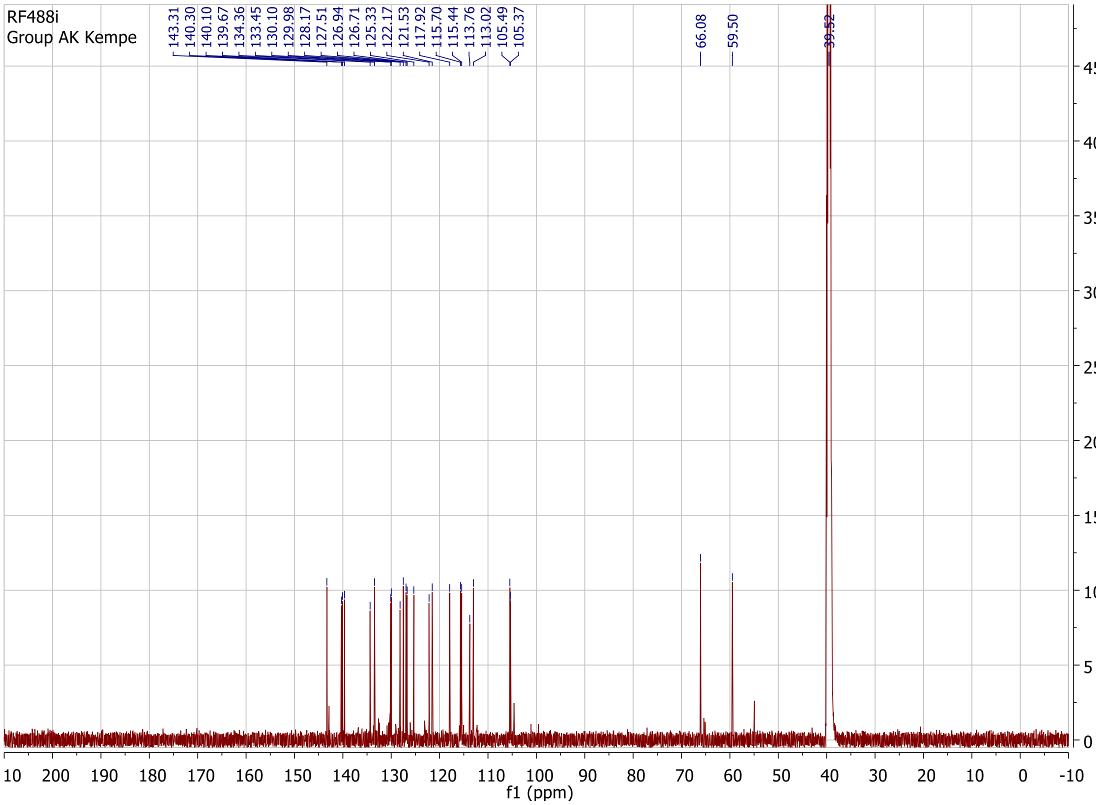

Supplementary Figure 137 ^{13}C NMR spectrum of compound **B2d**. (125 MHz, 293 K, DMSO-d$_6$).

NMR spectra of B2e

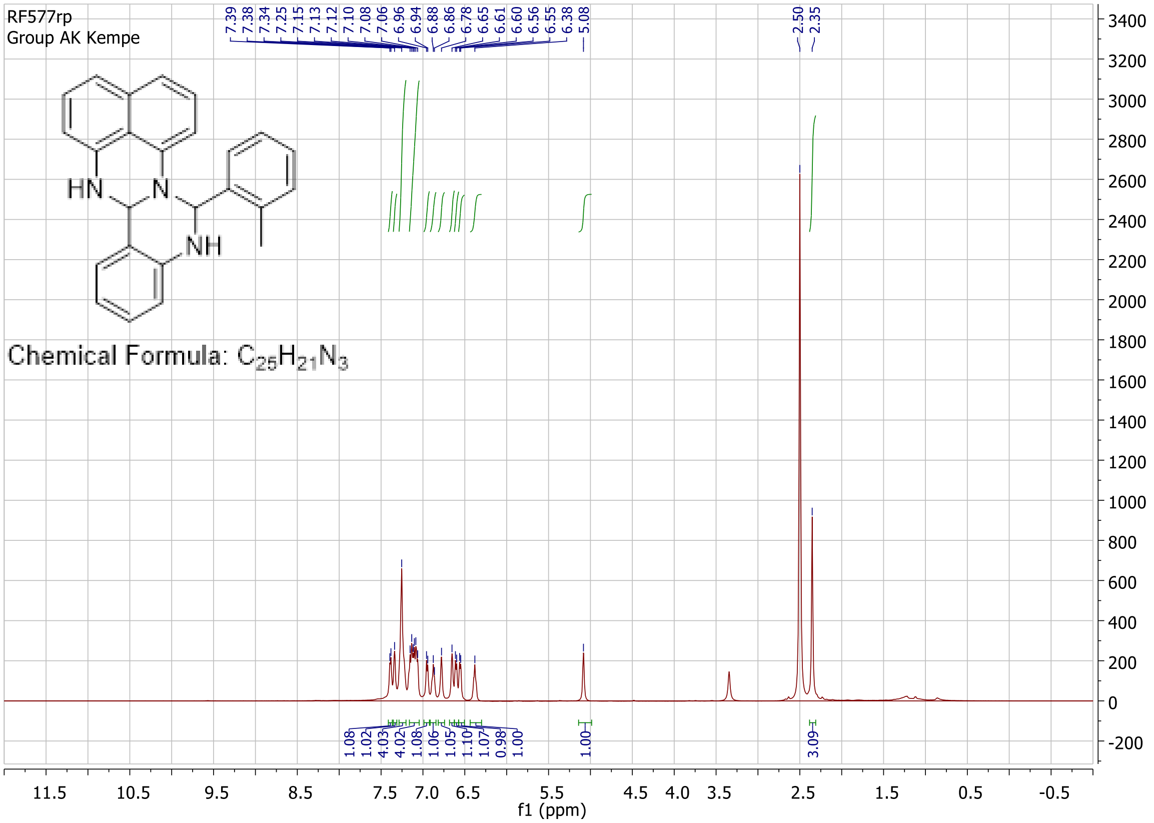

Supplementary Figure 138 ^{1}H NMR spectrum of compound **B2e**. (500 MHz, 293 K, DMSO-d_6).

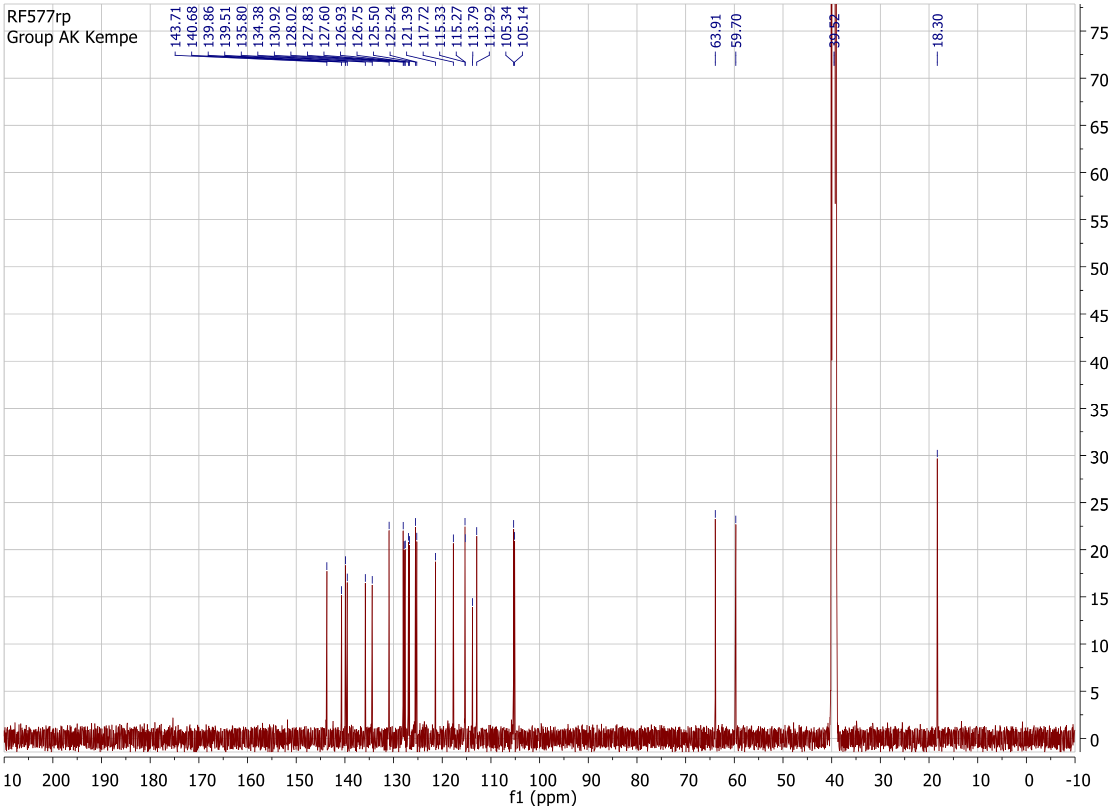

Supplementary Figure 139 ^{13}C NMR spectrum of compound **B2e**. (125 MHz, 293 K, DMSO-d_6).

NMR spectra of B2f

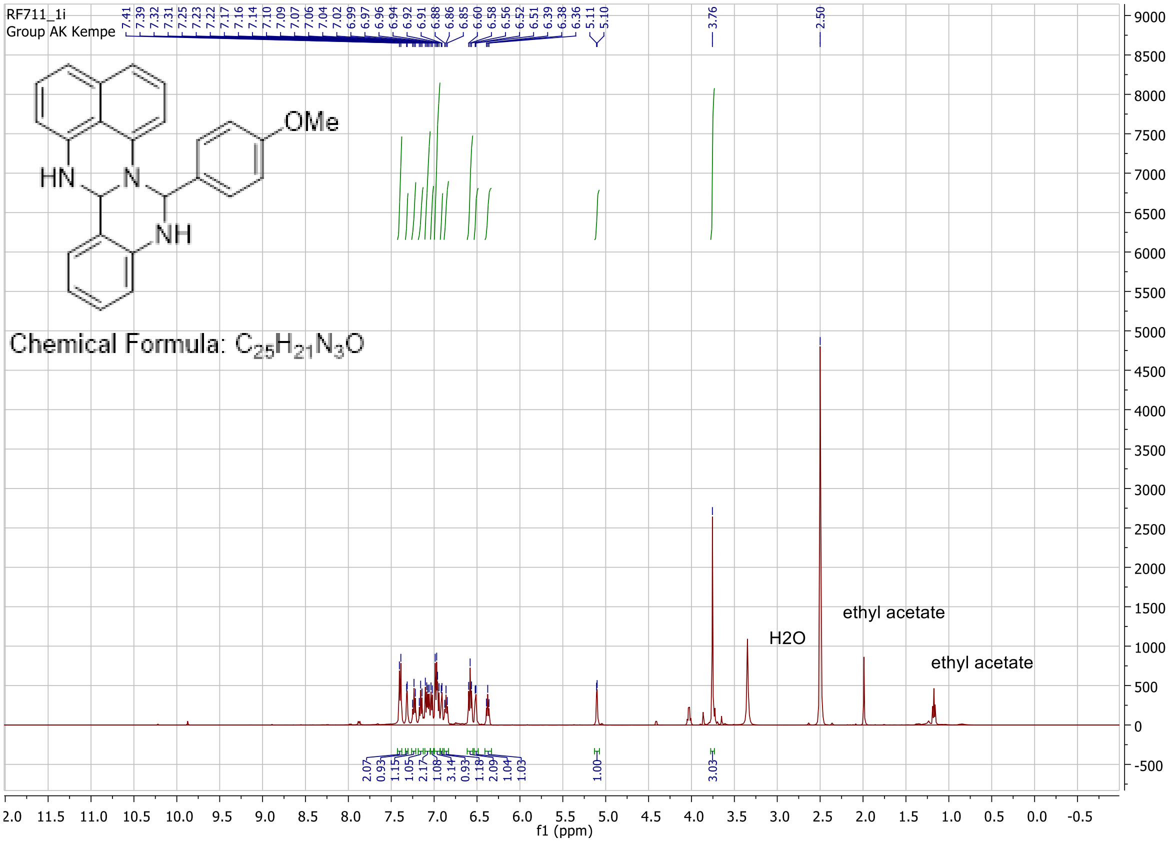

Supplementary Figure 140 ^{1}H NMR spectrum of compound **B2f**. (500 MHz, 293 K, DMSO-d_6).

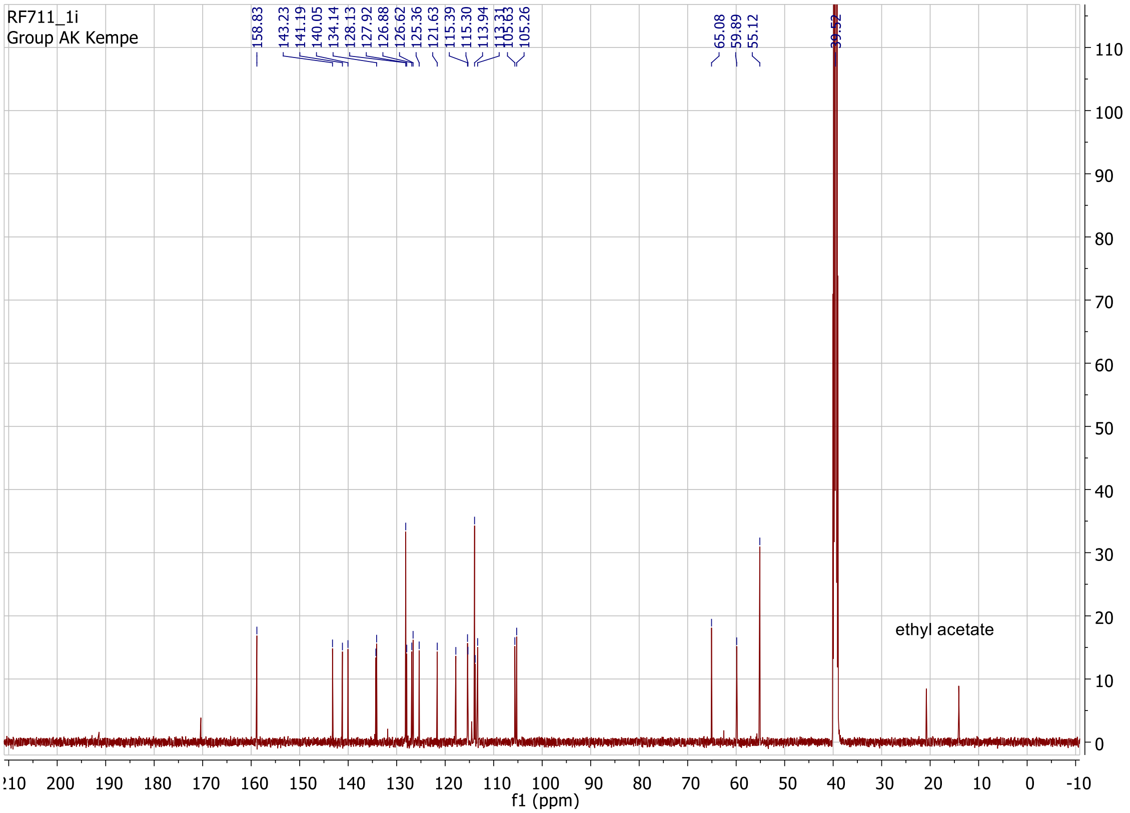

Supplementary Figure 141 ^{13}C NMR spectrum of compound **B2f**. (125 MHz, 293 K, DMSO-d_6).

NMR spectra of B2g

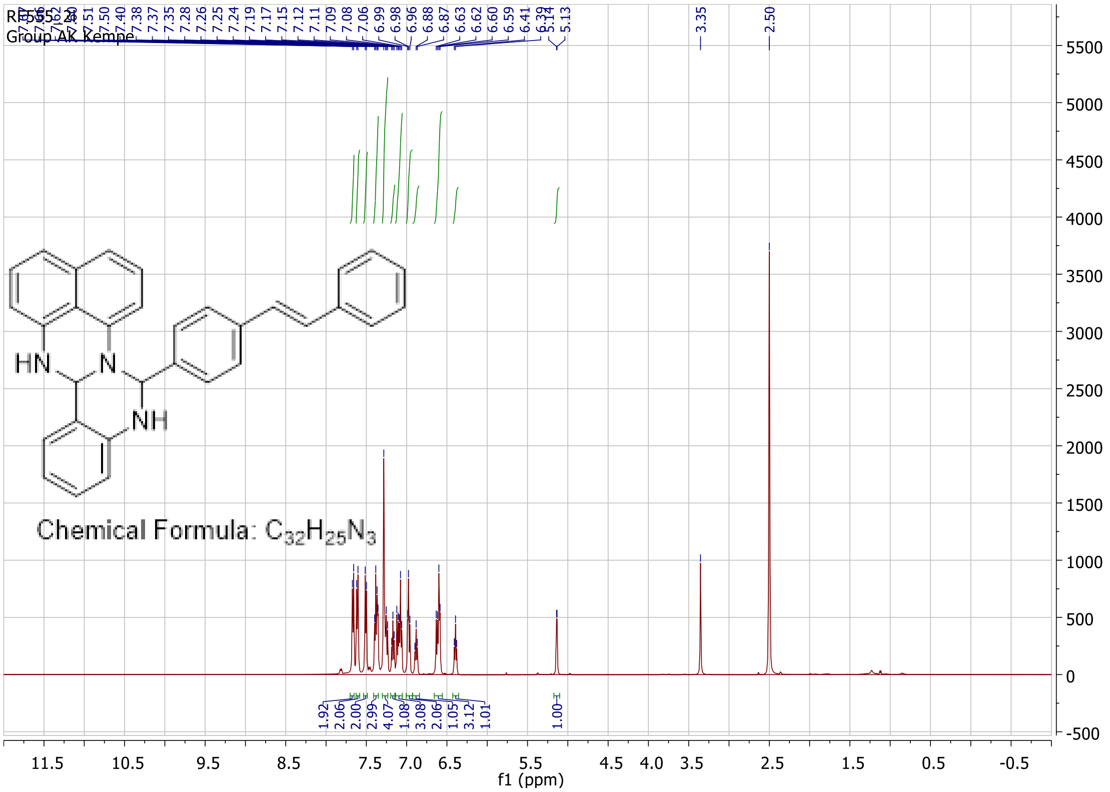

Supplementary Figure 142 ^{1}H NMR spectrum of compound **B2g**. (500 MHz, 293 K, DMSO-d_6).

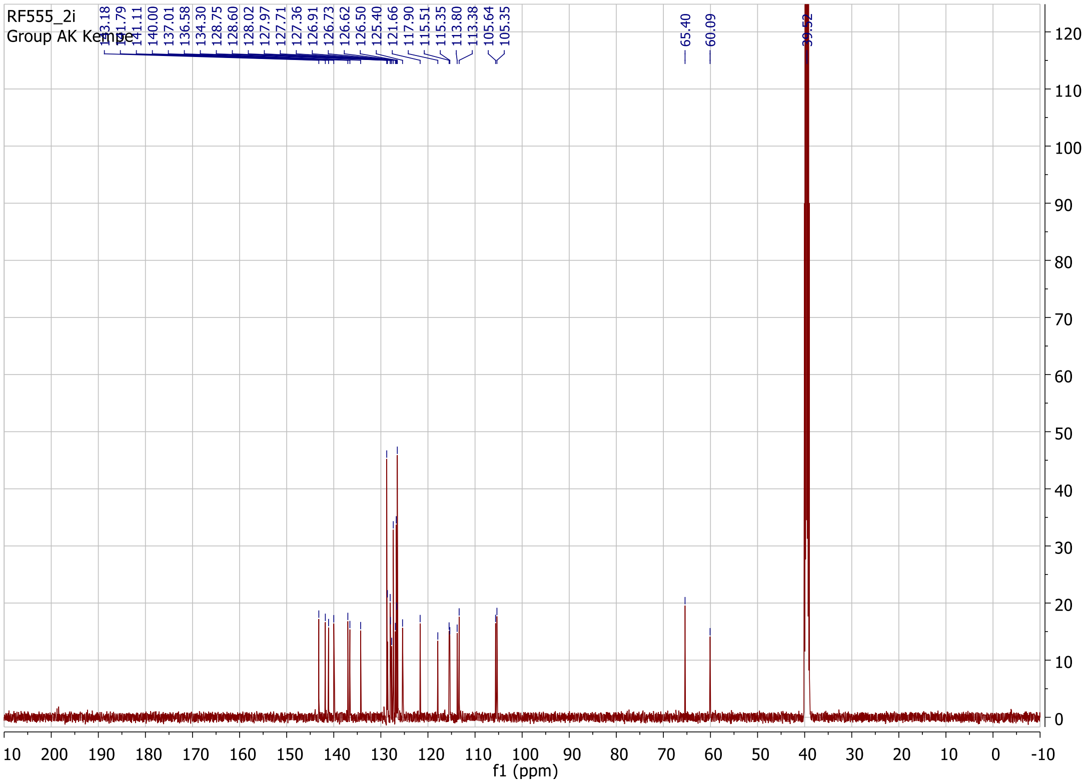

Supplementary Figure 143 ^{13}C NMR spectrum of compound **B2g**. (125 MHz, 293 K, DMSO-d_6).

NMR spectra of B2h

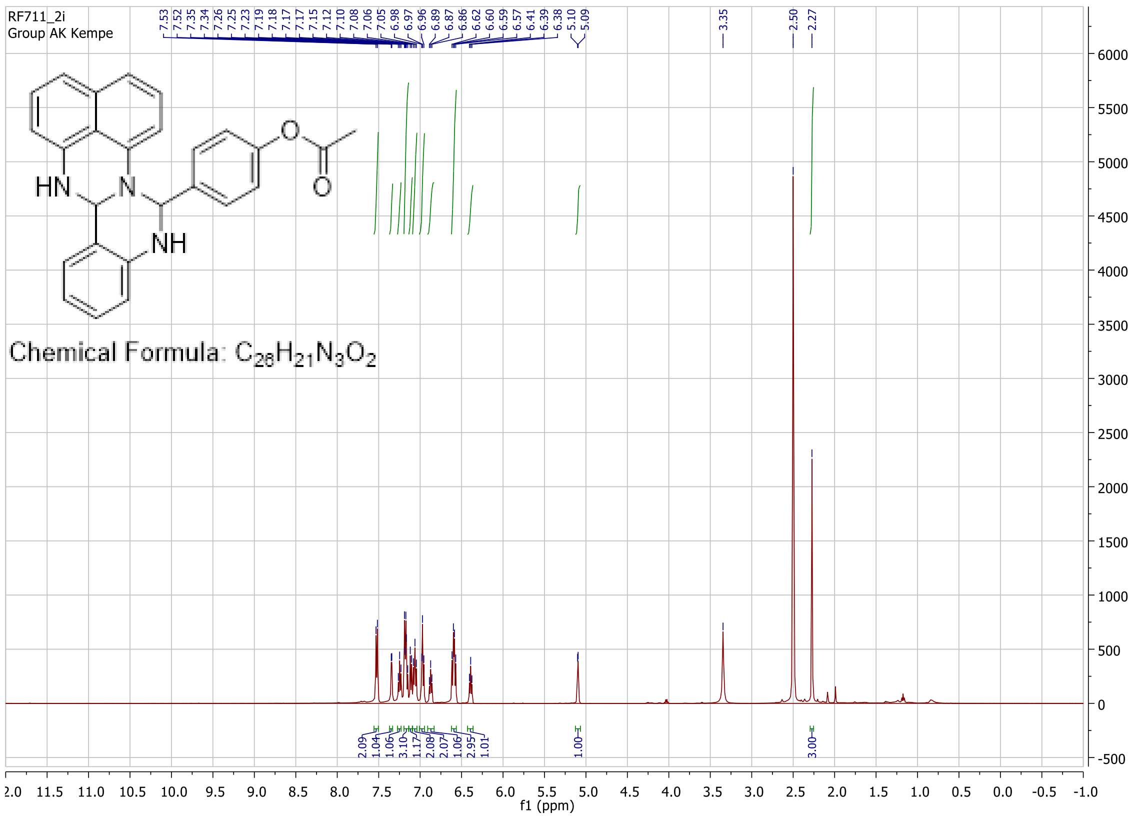

Supplementary Figure 144 ^{1}H NMR spectrum of compound **B2h**. (500 MHz, 293 K, DMSO-d_6).

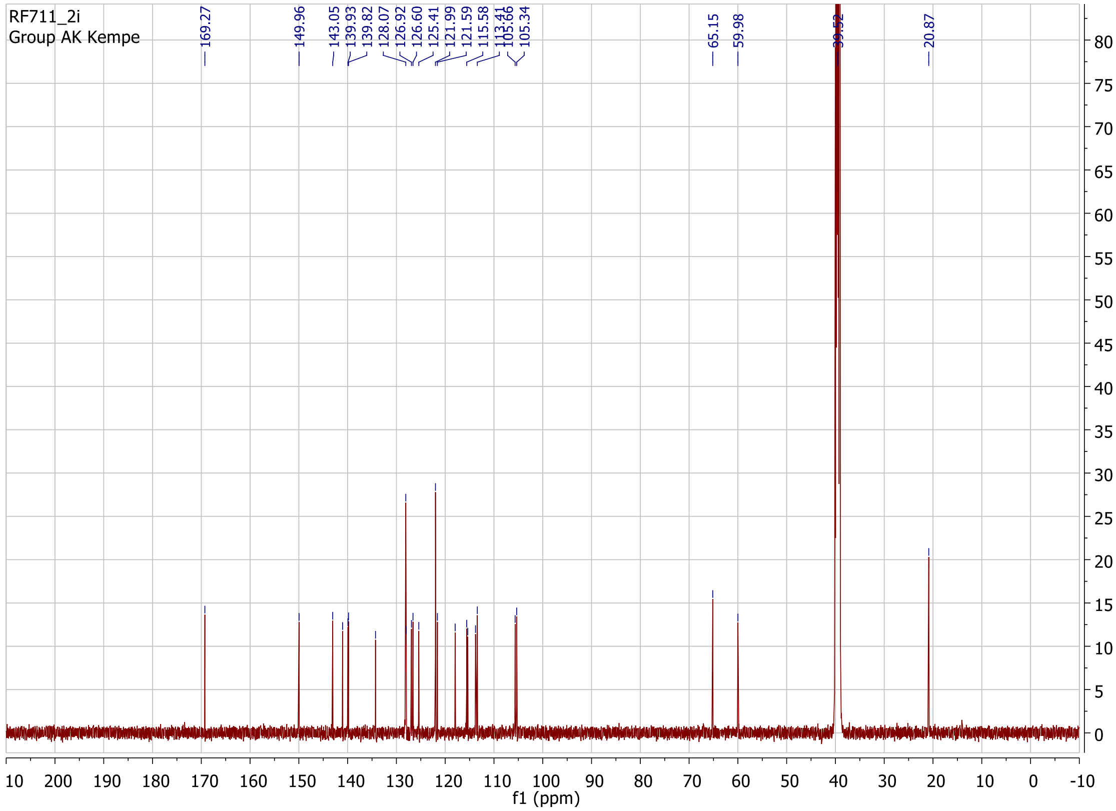

Supplementary Figure 145 ^{13}C NMR spectrum of compound **B2h**. (125 MHz, 293 K, DMSO-d_6).

NMR spectra of B2i

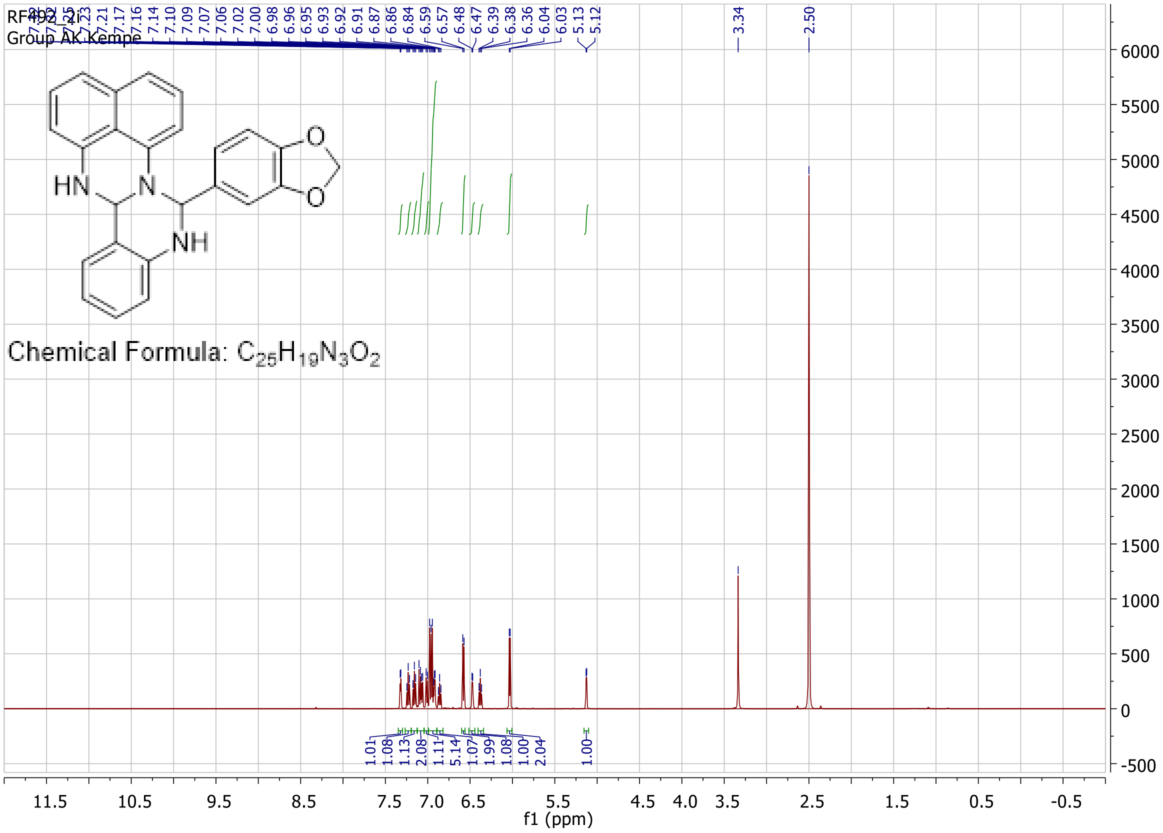

Supplementary Figure 146 ^{1}H NMR spectrum of compound **B2i**. (500 MHz, 293 K, DMSO-d$_6$).

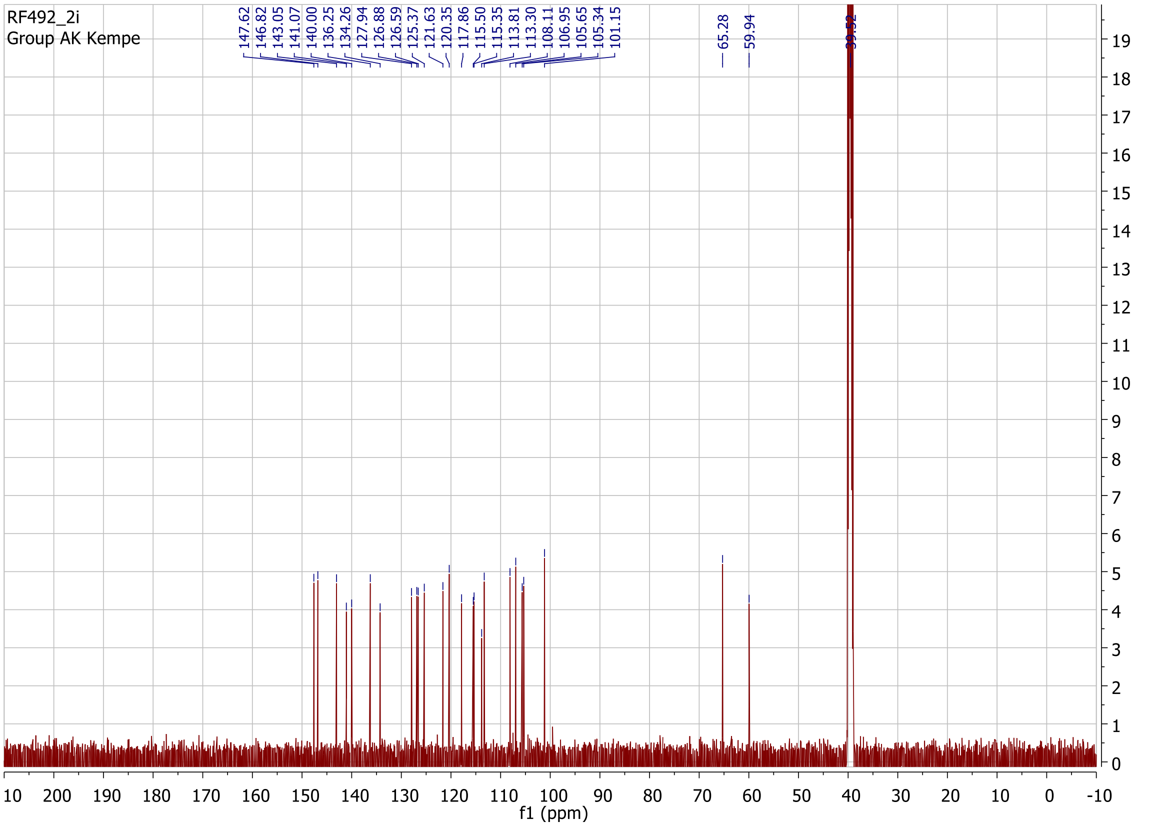

Supplementary Figure 147 ^{13}C NMR spectrum of compound **B2i**. (125 MHz, 293 K, DMSO-d$_6$).

NMR spectra of B2j

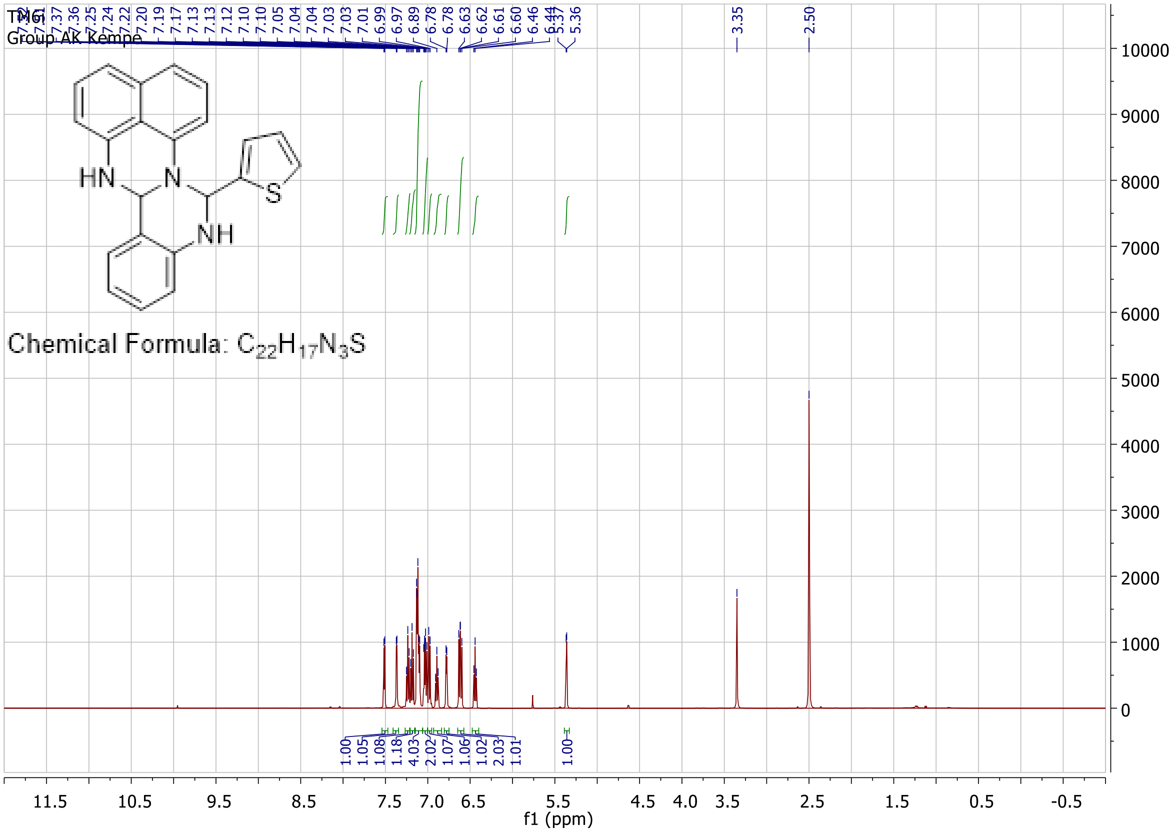

Supplementary Figure 148 ^{1}H NMR spectrum of compound **B2j**. (500 MHz, 293 K, DMSO-d_6).

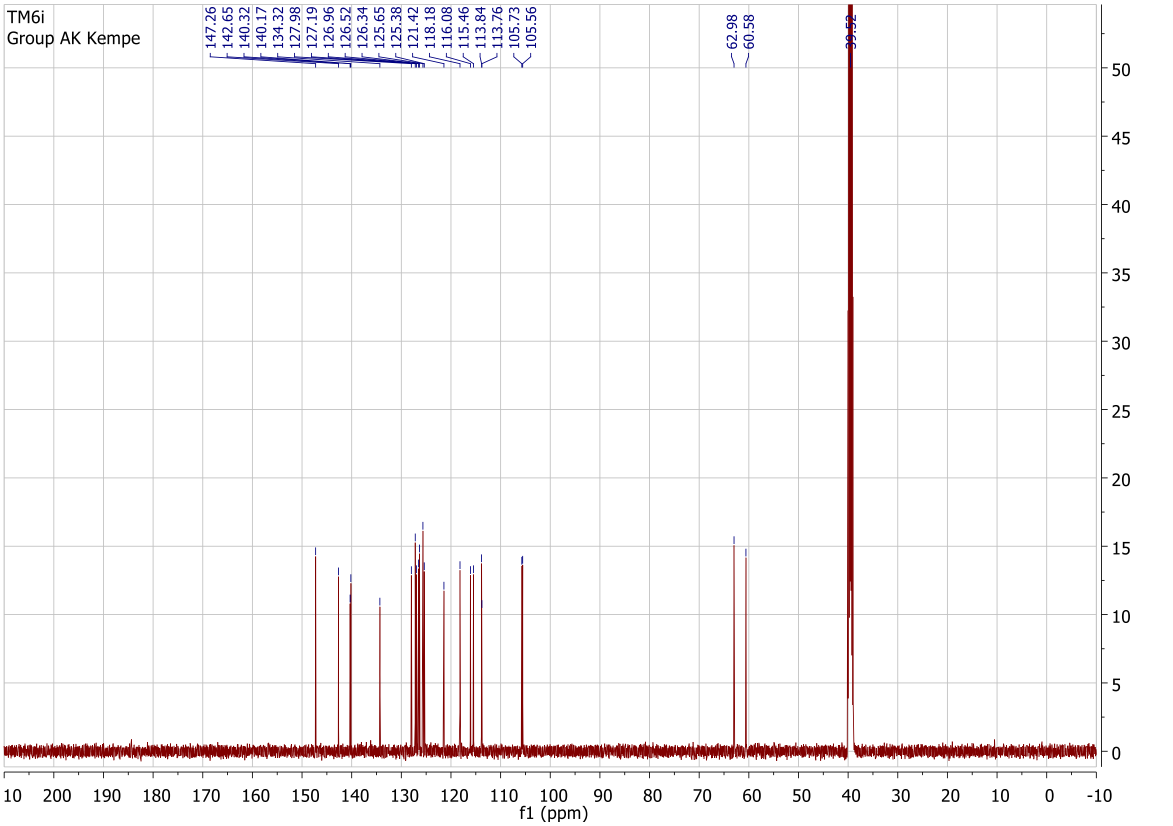

Supplementary Figure 149 ^{13}C NMR spectrum of compound **B2j**. (125 MHz, 293 K, DMSO-d_6).

NMR spectra of B2k

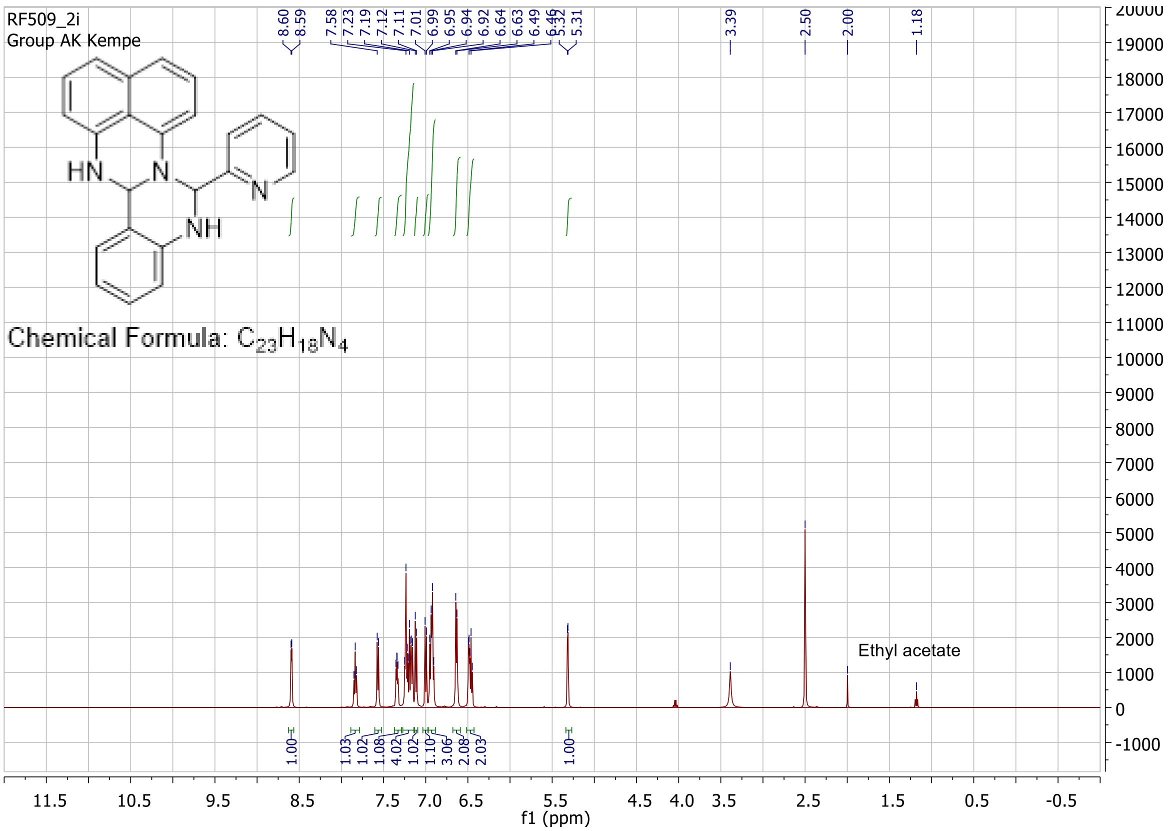

Supplementary Figure 150 ^{1}H NMR spectrum of compound **B2k**. (500 MHz, 293 K, DMSO-d_6).

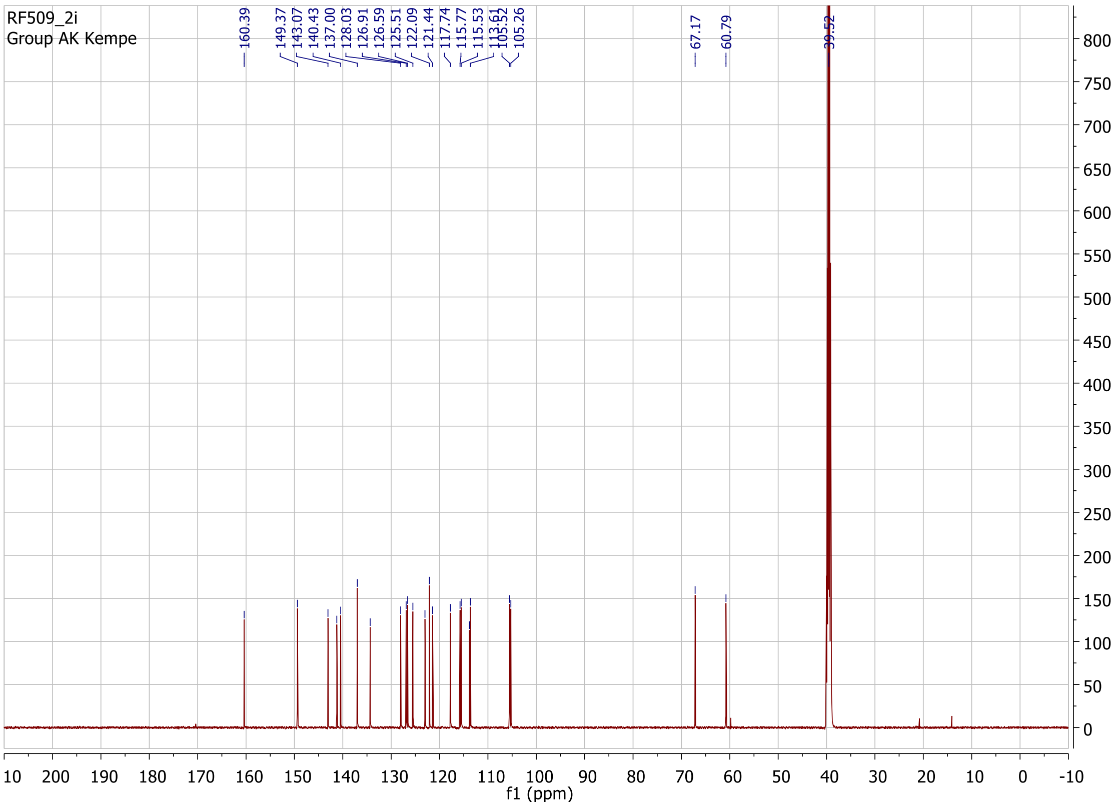

Supplementary Figure 151 ^{13}C NMR spectrum of compound **B2k**. (125 MHz, 293 K, DMSO-d_6).

NMR spectra of B2l

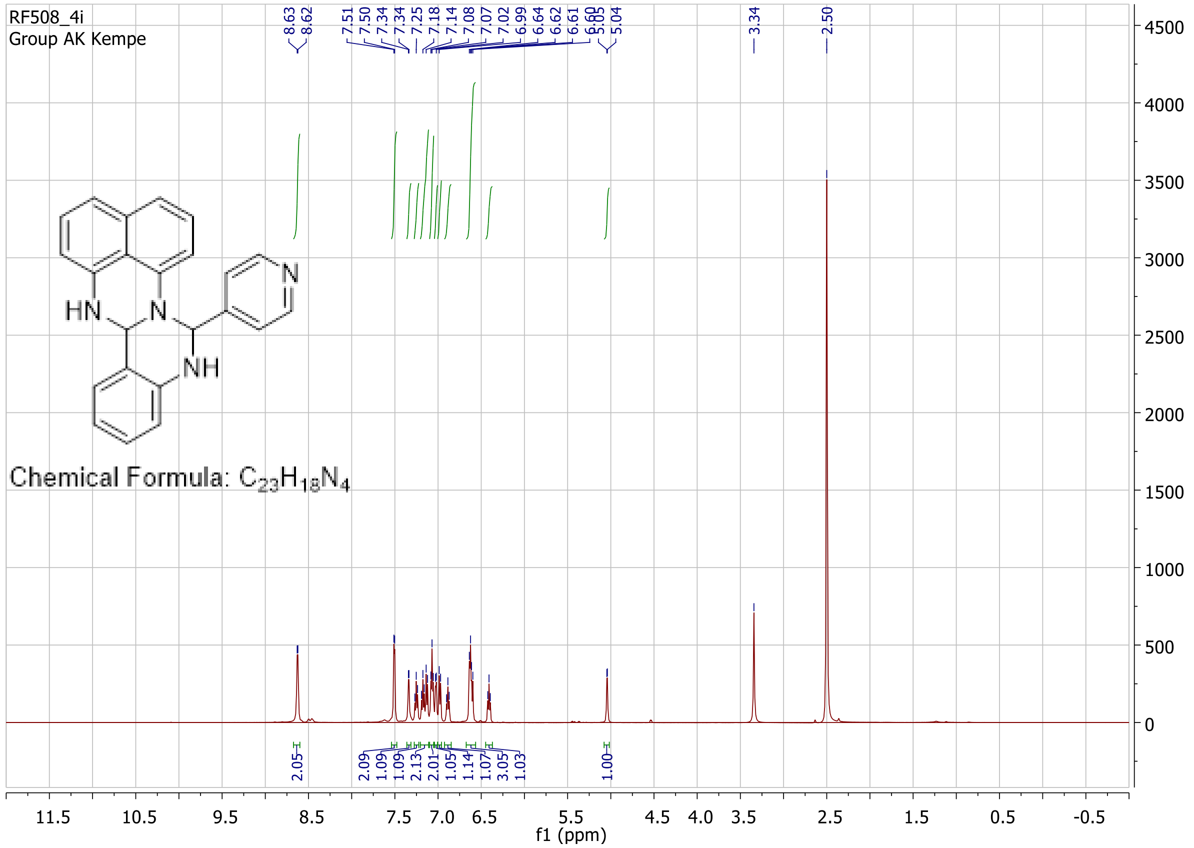

Supplementary Figure 152 ^{1}H NMR spectrum of compound **B2l**. (500 MHz, 293 K, DMSO-d$_6$).

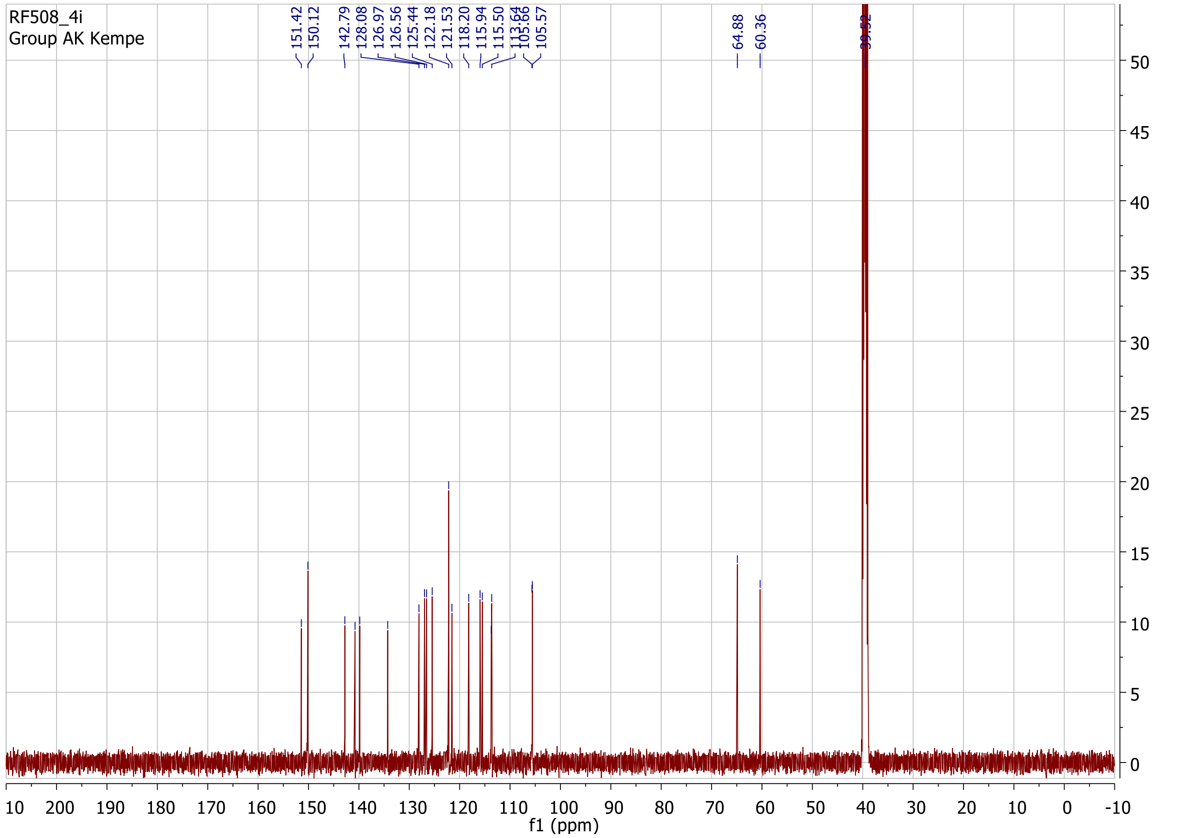

Supplementary Figure 153 ^{13}C NMR spectrum of compound **B2l**. (125 MHz, 293 K, DMSO-d$_6$).

NMR spectra of B2m

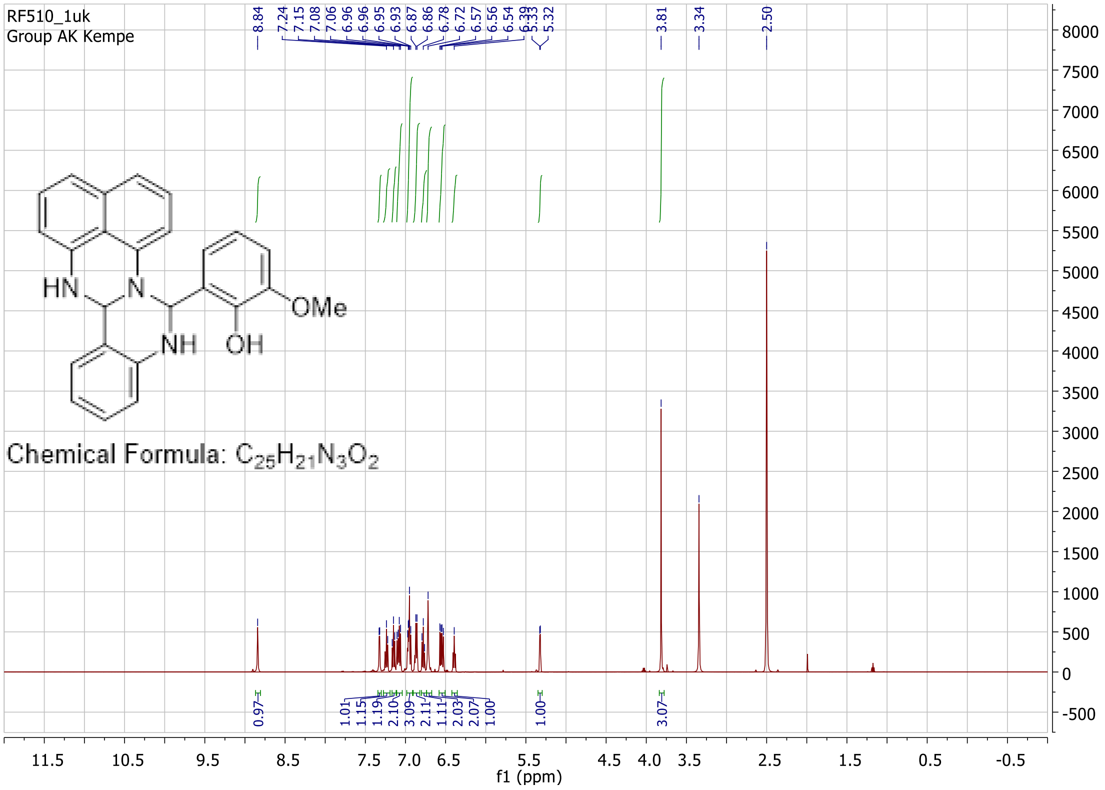

Supplementary Figure 154 ^{1}H NMR spectrum of compound **B2m**. (500 MHz, 293 K, DMSO-d_6).

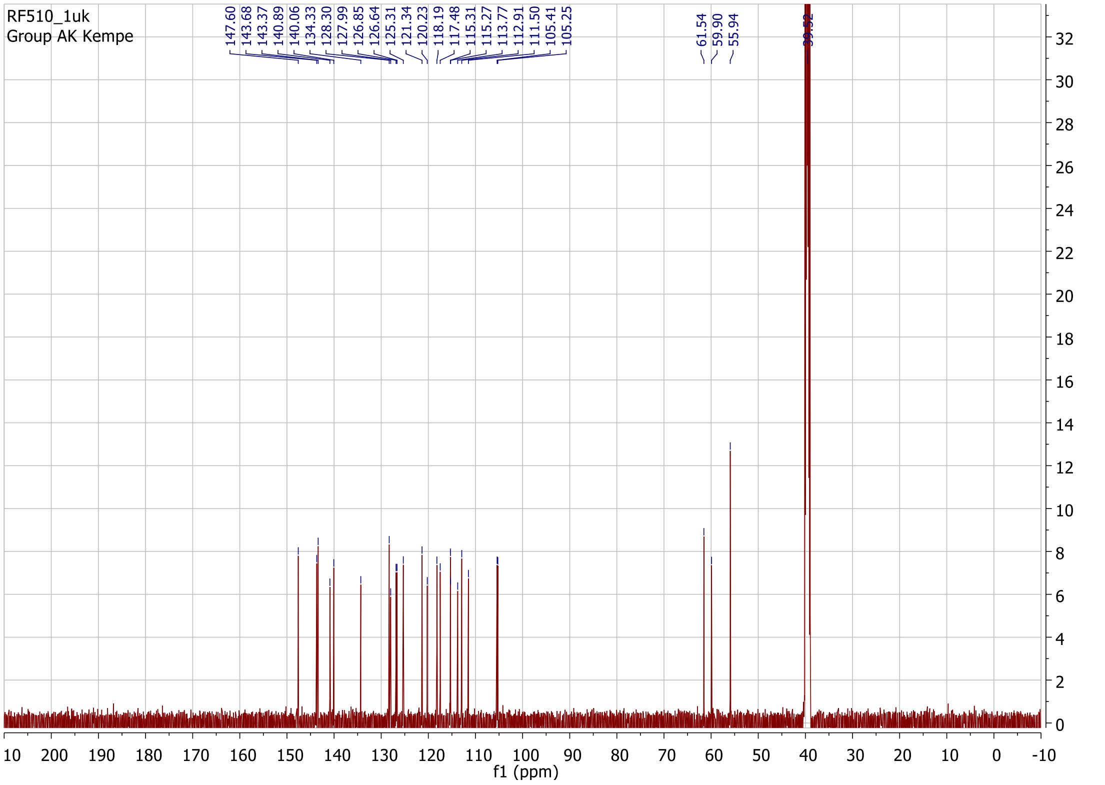

Supplementary Figure 155 ^{13}C NMR spectrum of compound **B2m**. (125 MHz, 293 K, DMSO-d_6).

NMR spectra of B2n

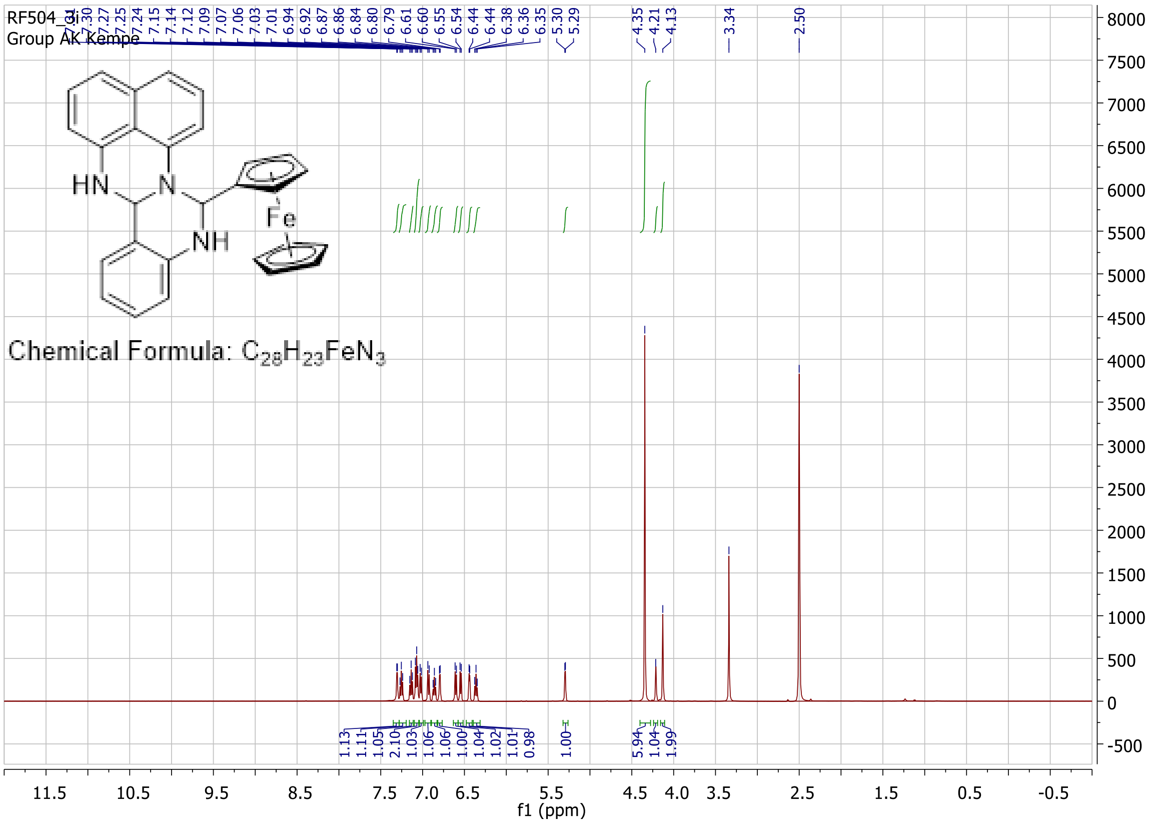

Supplementary Figure 156 ^{1}H NMR spectrum of compound **B2n**. (500 MHz, 293 K, DMSO-d_6).

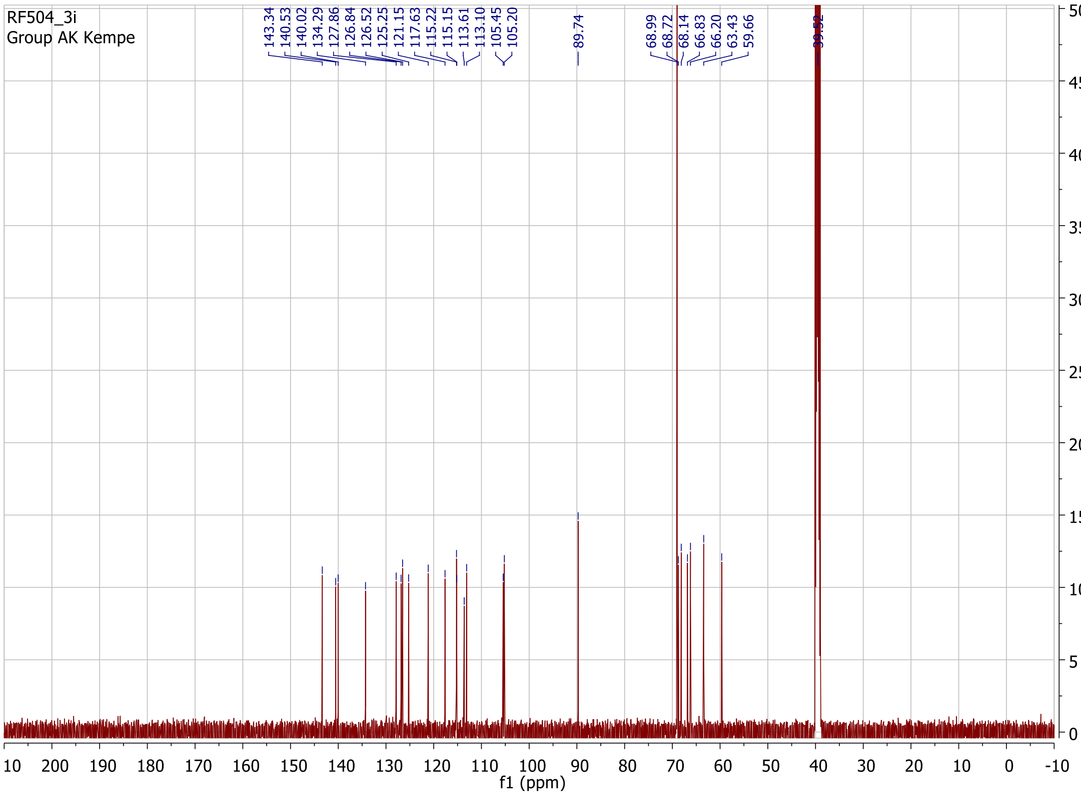

Supplementary Figure 157 ^{13}C NMR spectrum of compound **B2n**. (125 MHz, 293 K, DMSO-d_6).

NMR spectra of B2o

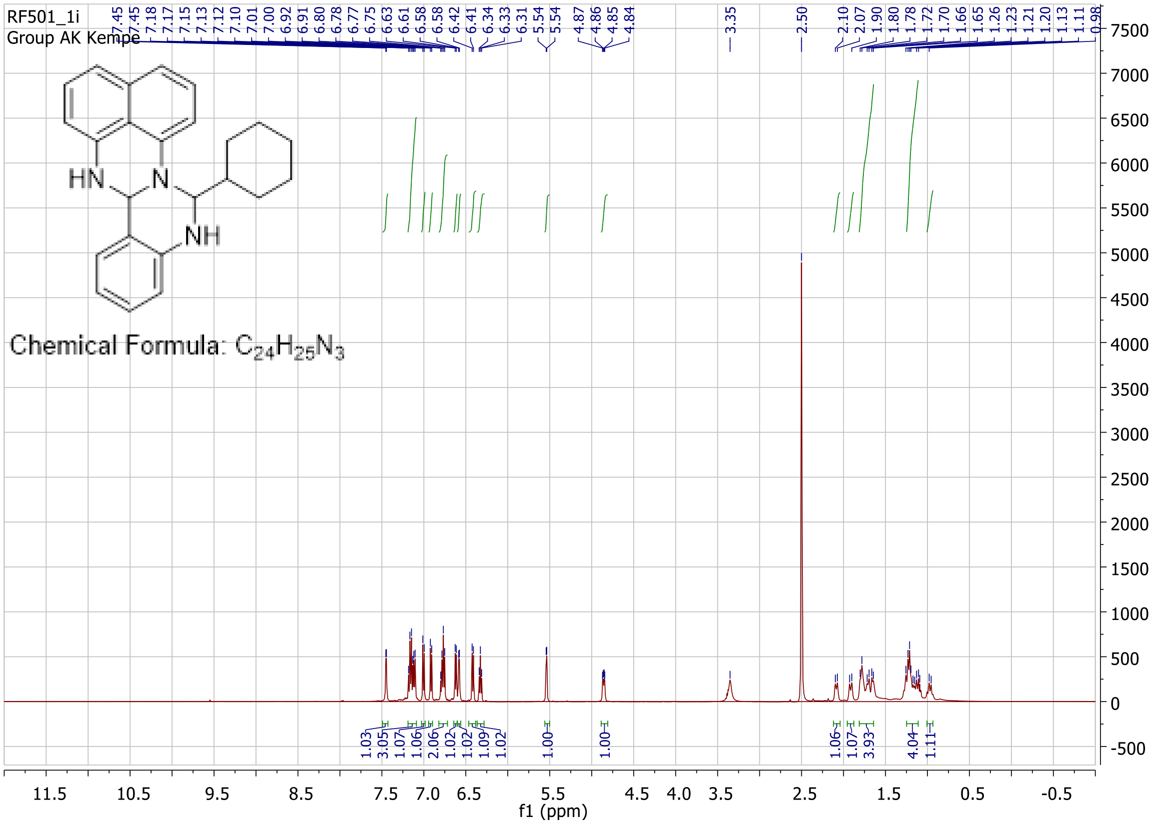

Supplementary Figure 158 ^{1}H NMR spectrum of compound **B2o**. (500 MHz, 293 K, DMSO-d$_6$).

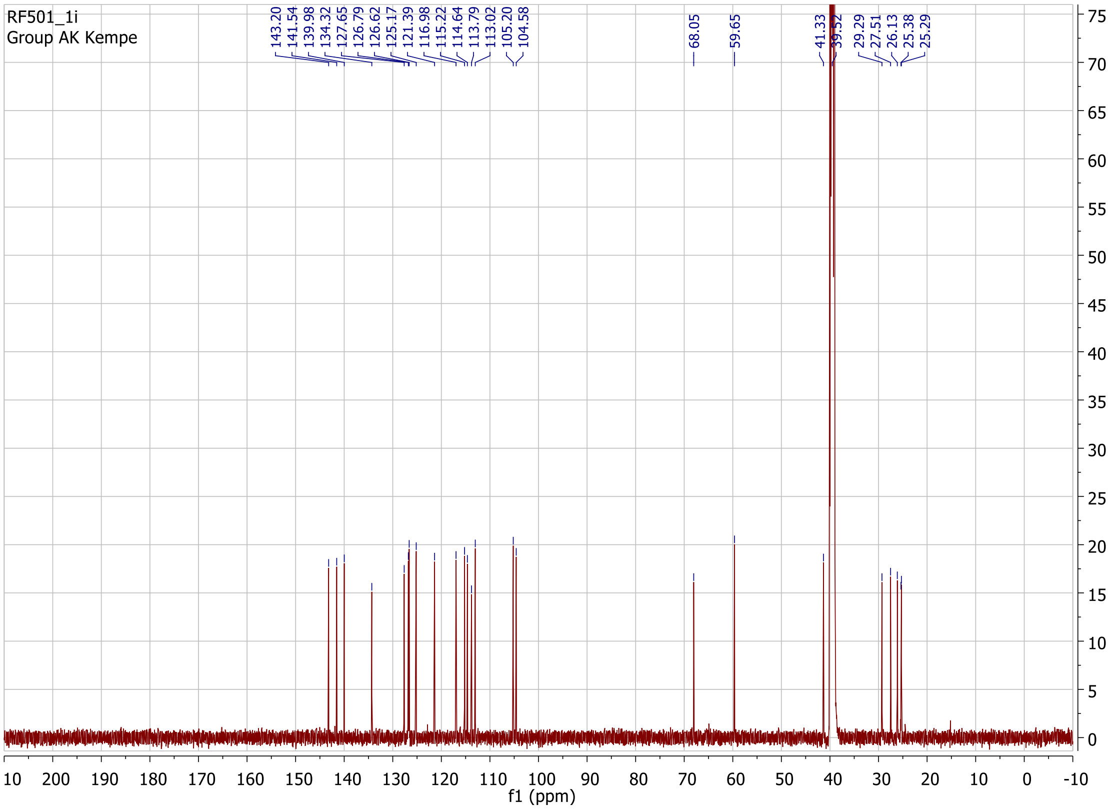

Supplementary Figure 159 ^{13}C NMR spectrum of compound **B2o**. (125 MHz, 293 K, DMSO-d$_6$).

NMR spectra of B2p

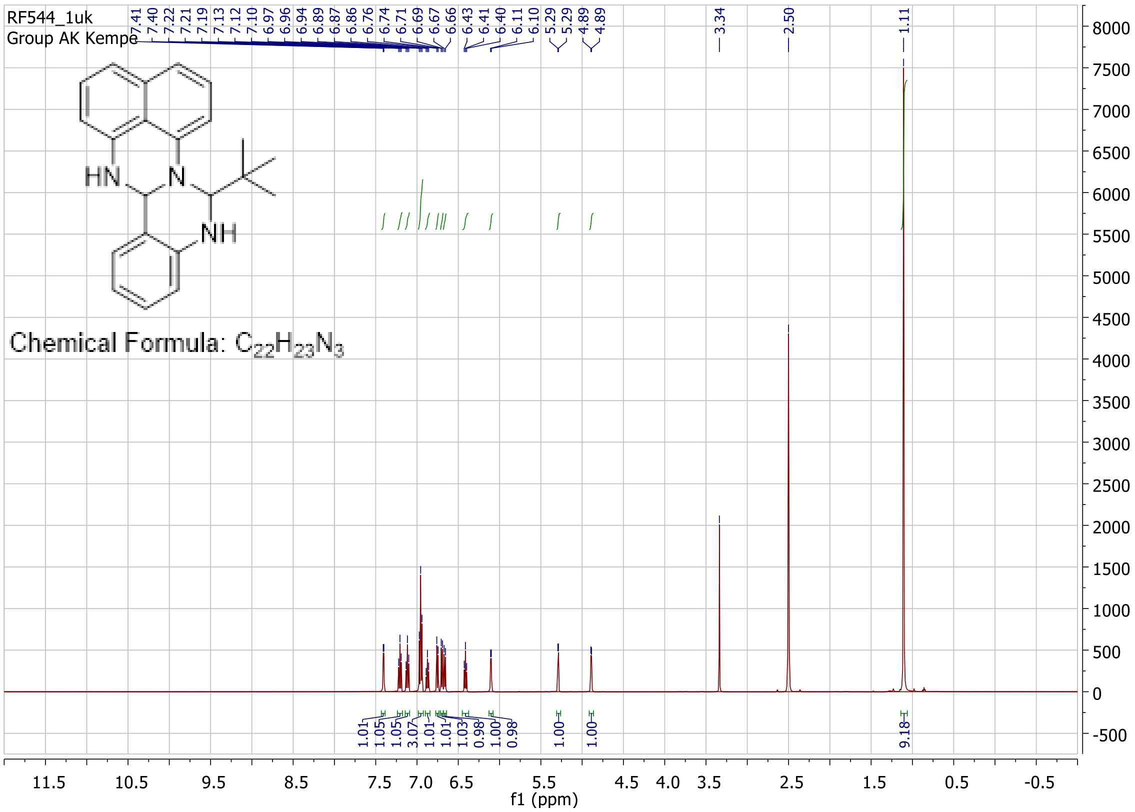

Supplementary Figure 160 ^{1}H NMR spectrum of compound **B2p**. (500 MHz, 293 K, DMSO-d_6).

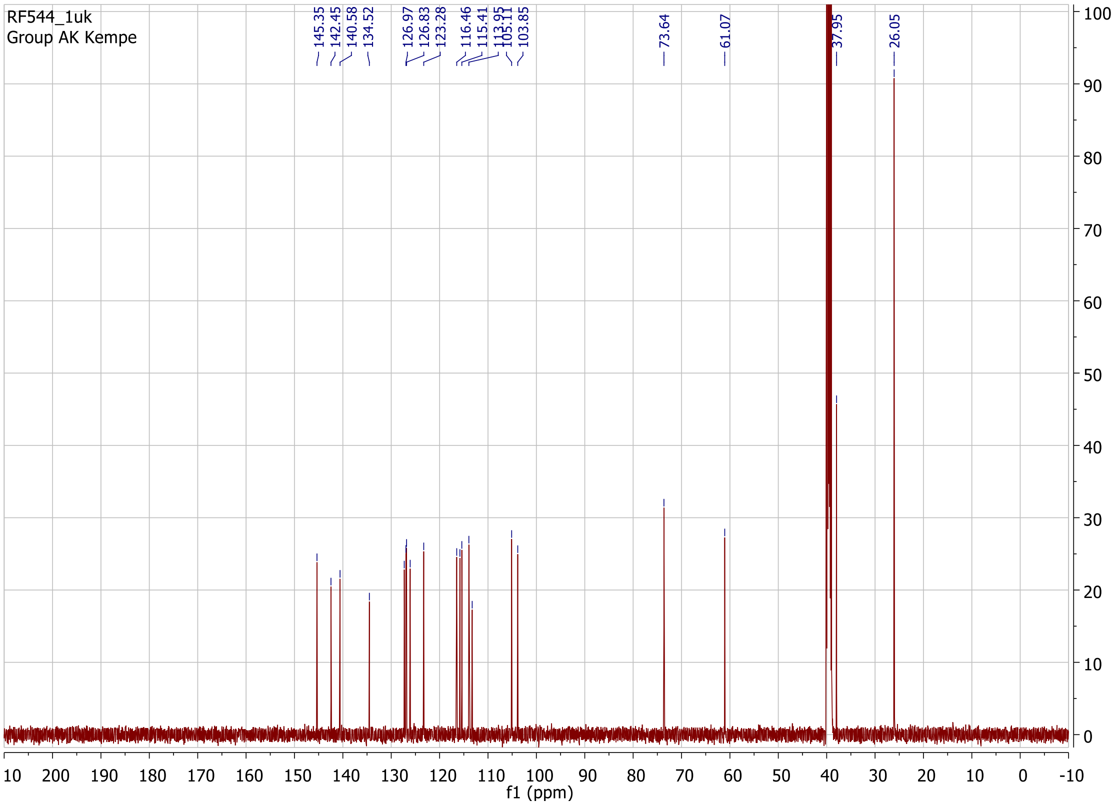

Supplementary Figure 161 ^{13}C NMR spectrum of compound **B2p**. (125 MHz, 293 K, DMSO-d_6).

NMR spectra of B3a

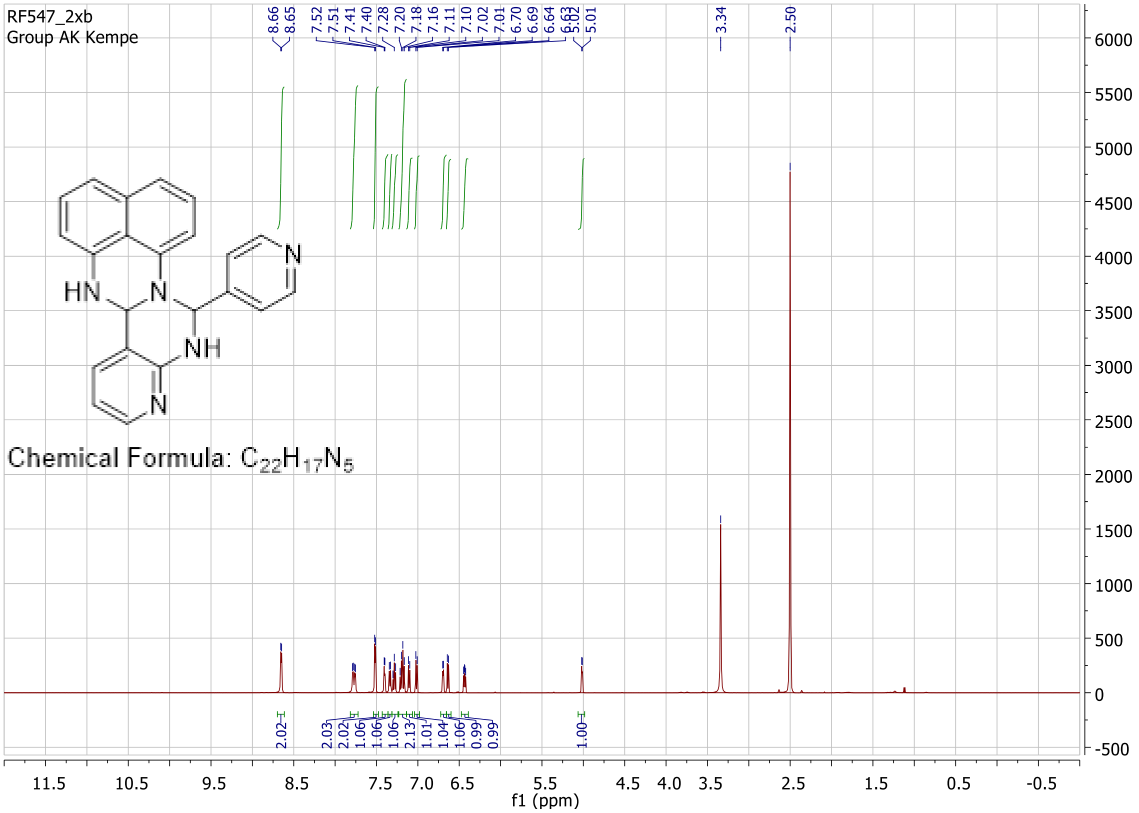

Supplementary Figure 162 ^{1}H NMR spectrum of compound **B3a**. (500 MHz, 293 K, DMSO-d_6).

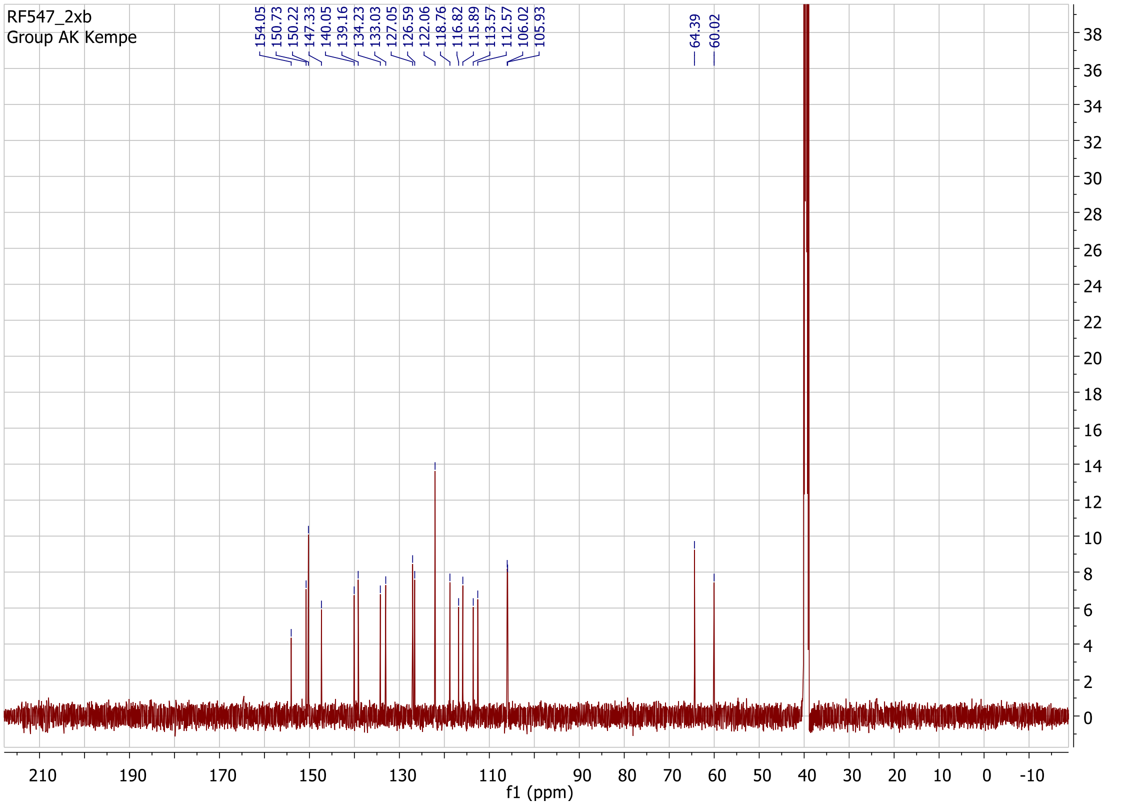

Supplementary Figure 163 ^{13}C NMR spectrum of compound **B3a**. (125 MHz, 293 K, DMSO-d_6).

NMR spectra of B3b

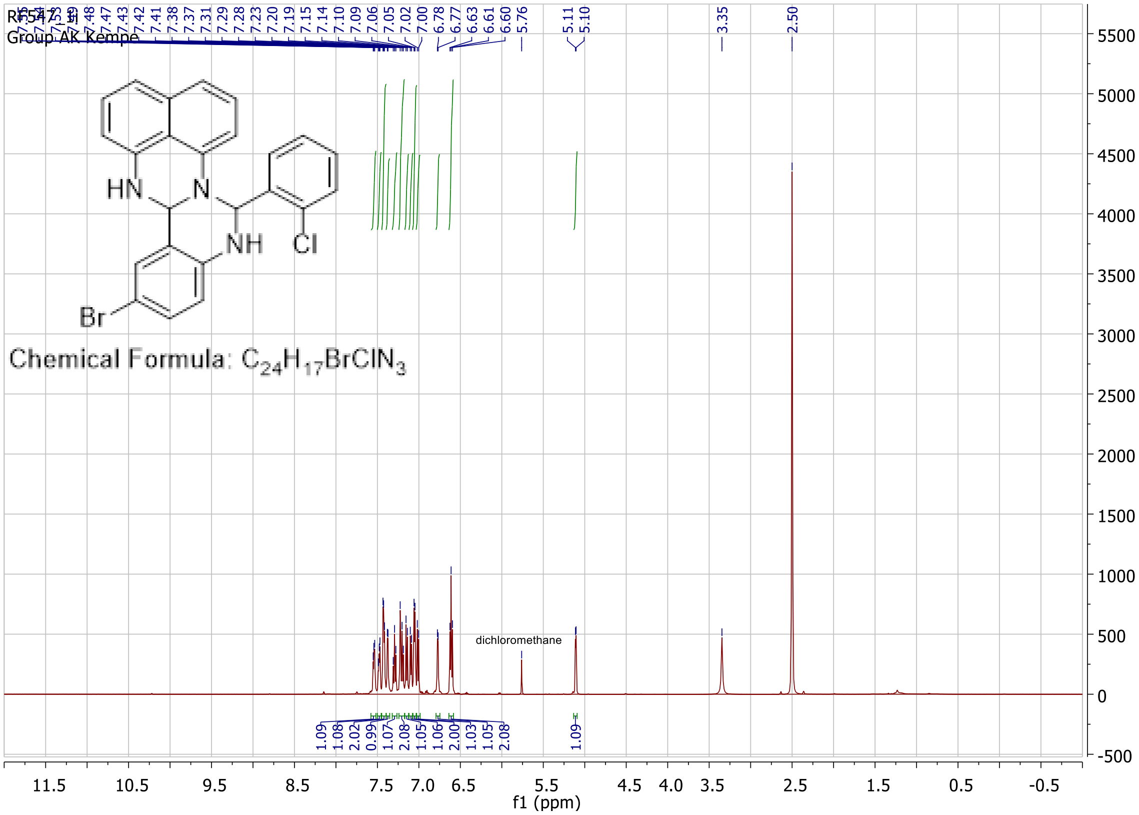

Supplementary Figure 164 ^{1}H NMR spectrum of compound **B3b**. (500 MHz, 293 K, DMSO-d_6).

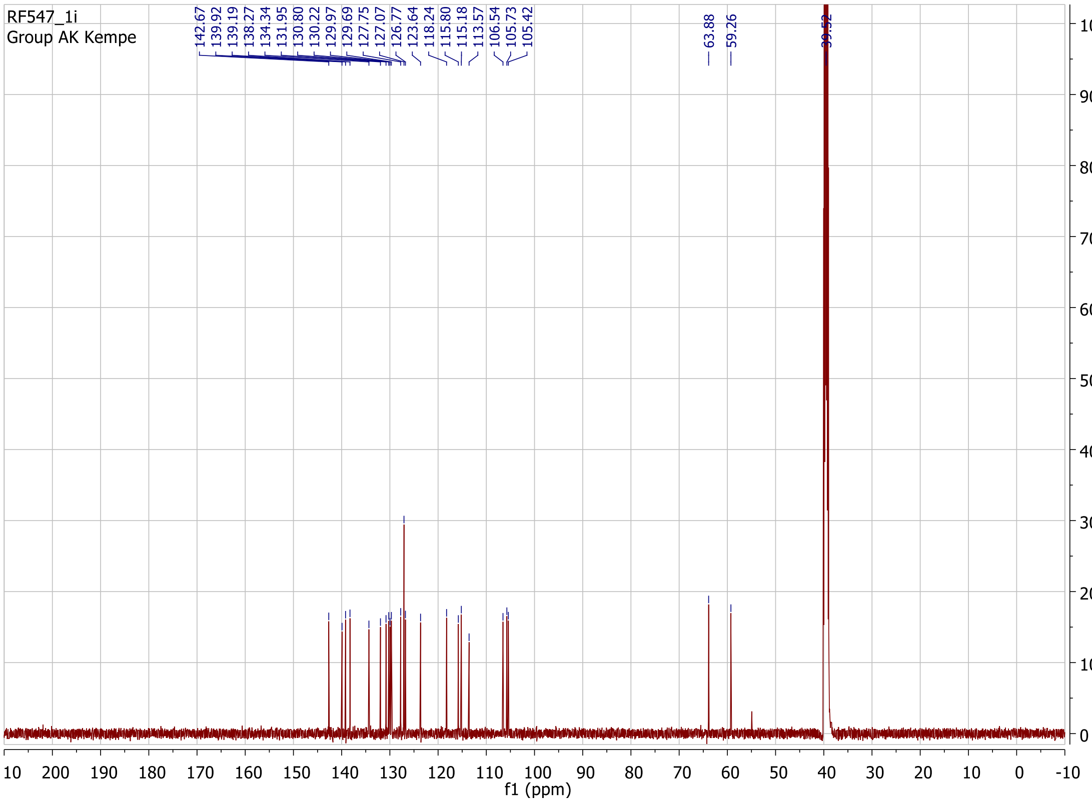

Supplementary Figure 165 ^{13}C NMR spectrum of compound **B3b**. (125 MHz, 293 K, DMSO-d_6).

NMR spectra of B3c

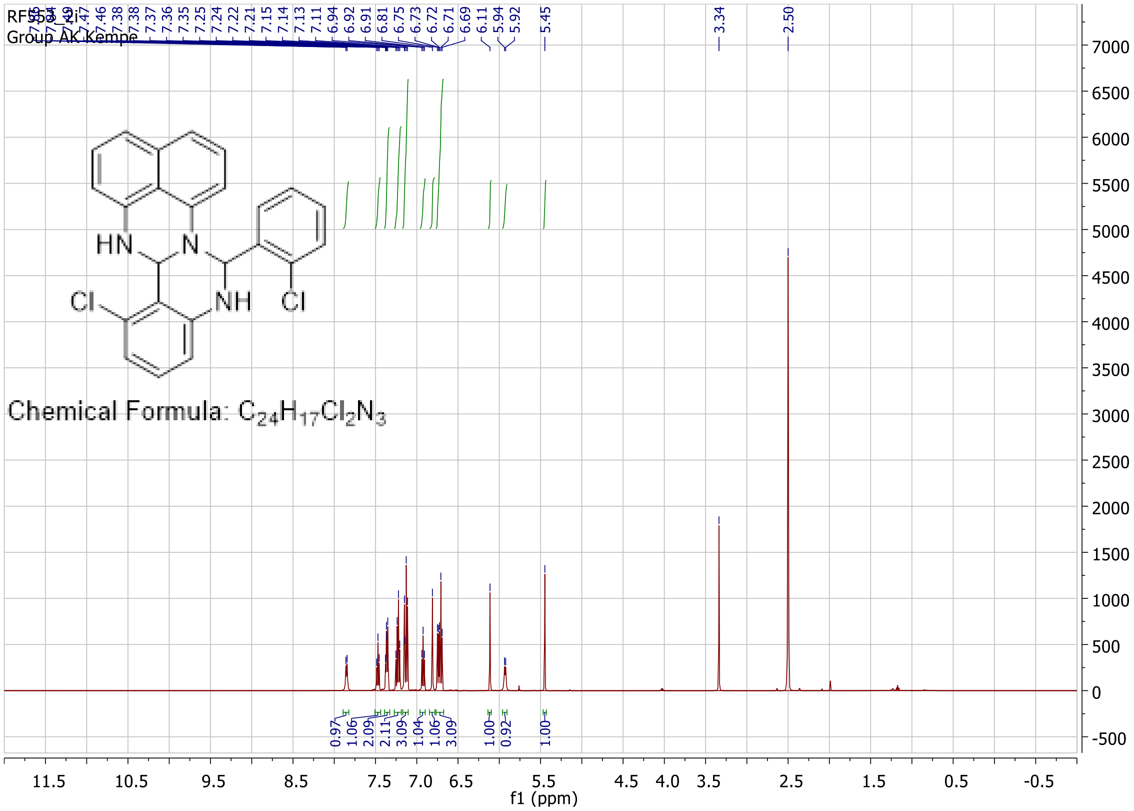

Supplementary Figure 166 ^{1}H NMR spectrum of compound **B3c**. (500 MHz, 293 K, DMSO-d_6).

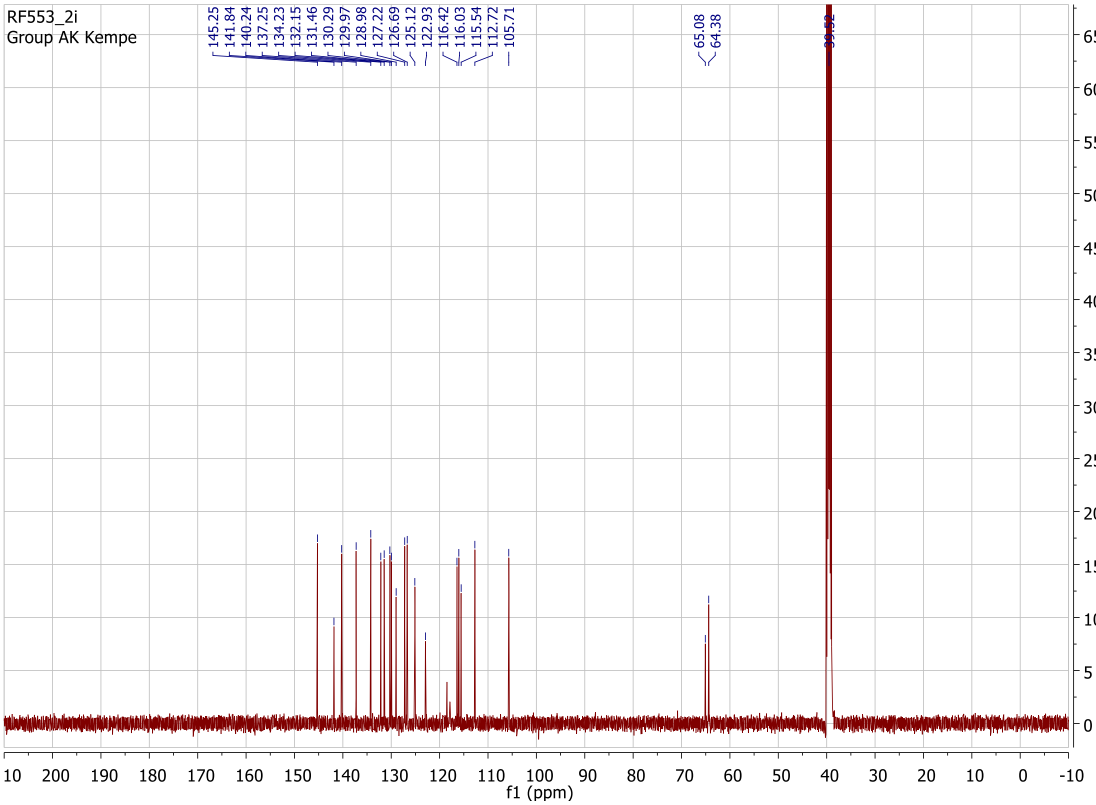

Supplementary Figure 167 ^{13}C NMR spectrum of compound **B3c**. (125 MHz, 293 K, DMSO-d_6).

NMR spectra of B3d

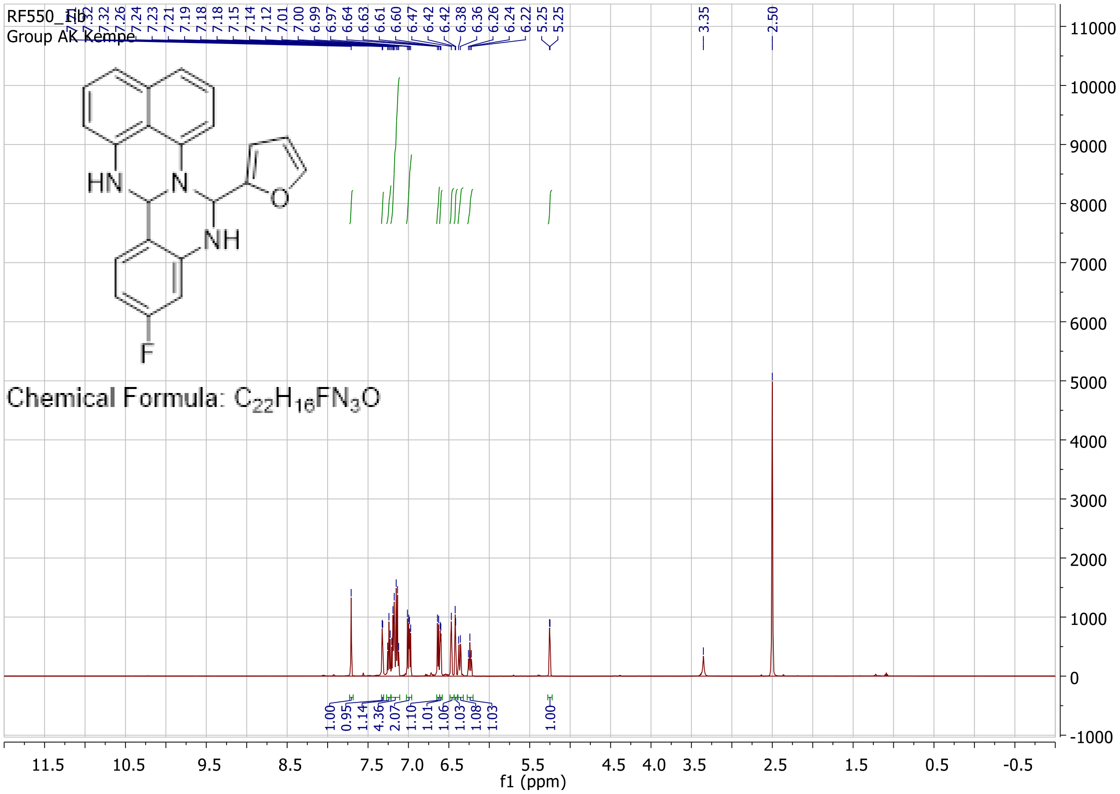

Supplementary Figure 168 ^{1}H NMR spectrum of compound **B3d**. (500 MHz, 293 K, DMSO-d_6).

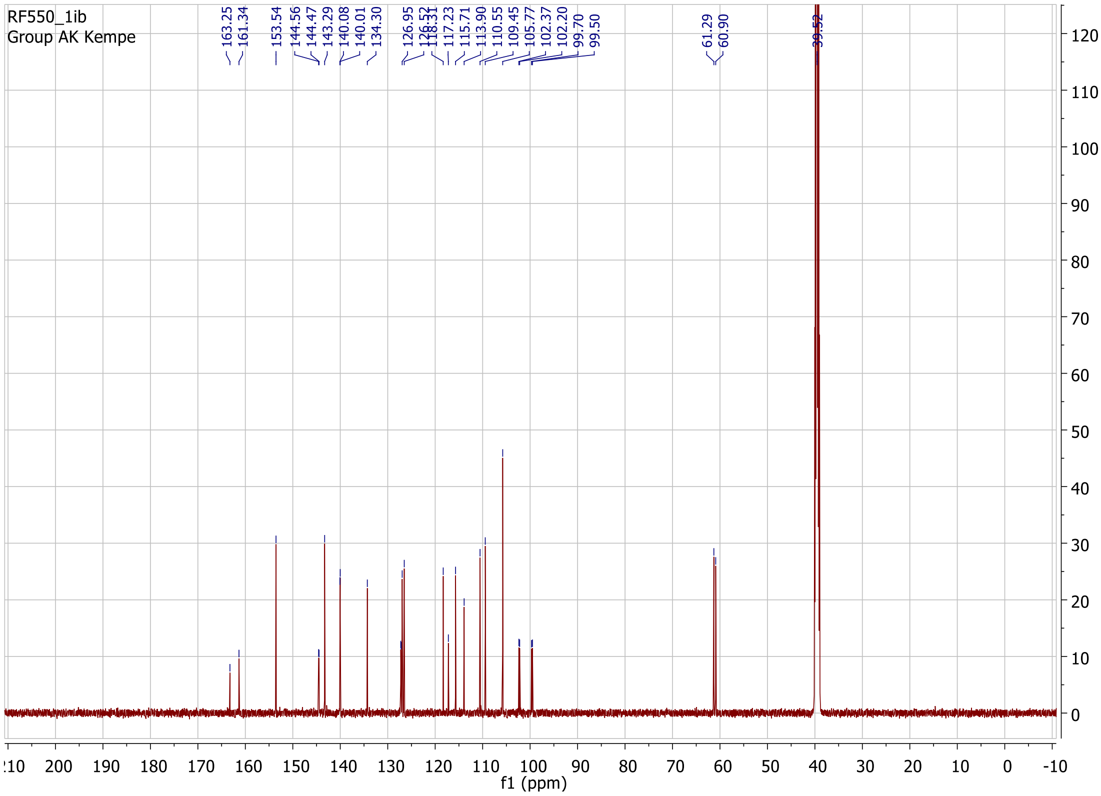

Supplementary Figure 169 ^{13}C NMR spectrum of compound **B3d**. (125 MHz, 293 K, DMSO-d_6).

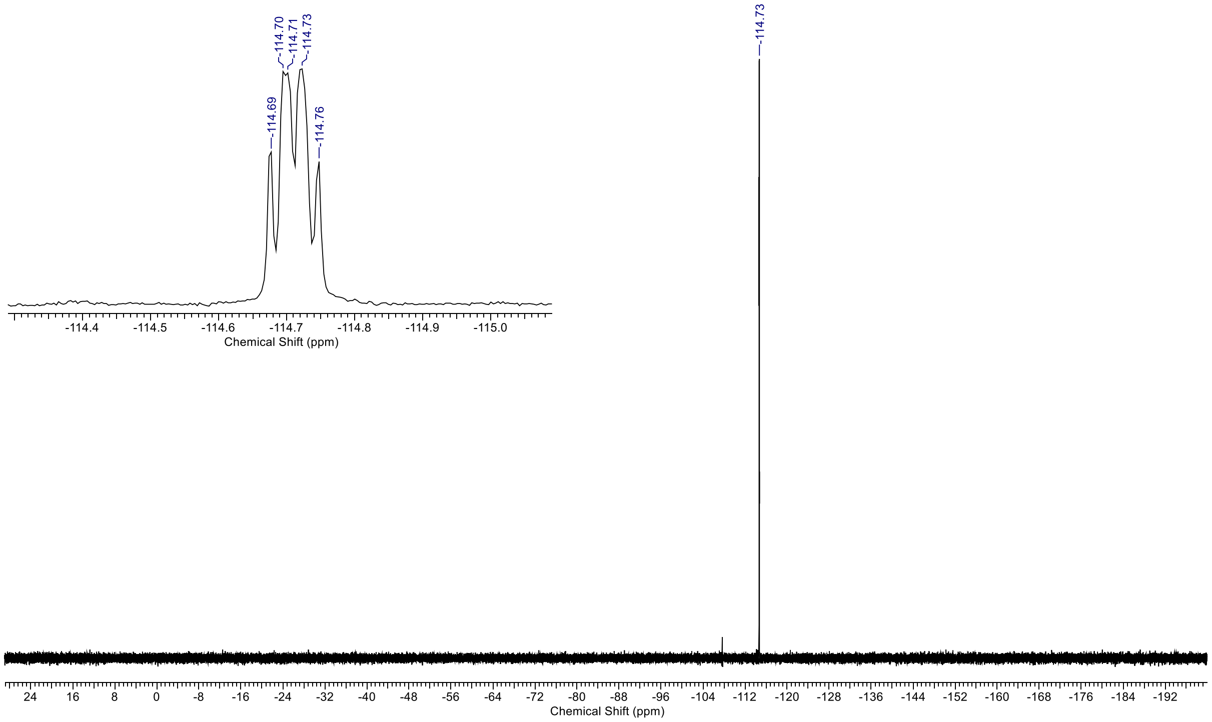

Supplementary Figure 170 ^{19}F NMR spectrum of compound **B3d**. (376 MHz, 293 K, DMSO-d_6).

NMR spectra of B3e

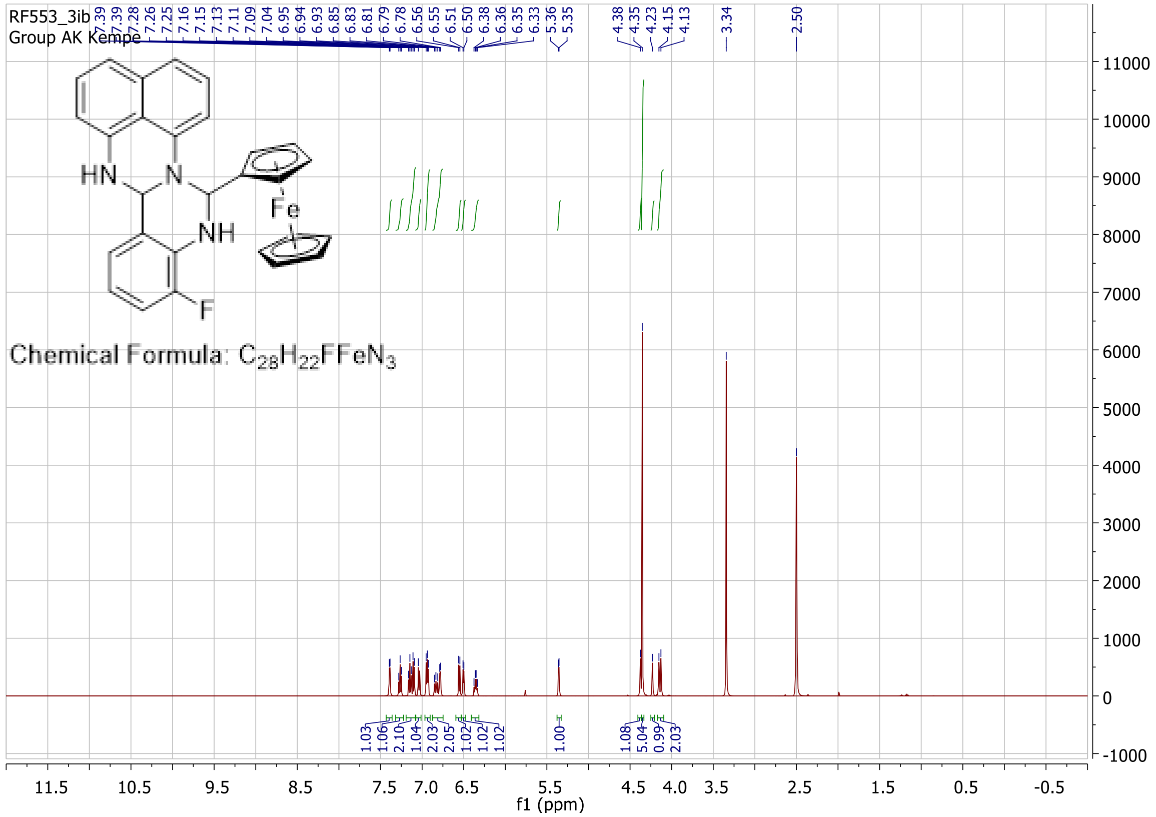

Supplementary Figure 171 ^{1}H NMR spectrum of compound **B3e**. (500 MHz, 293 K, DMSO-d_6).

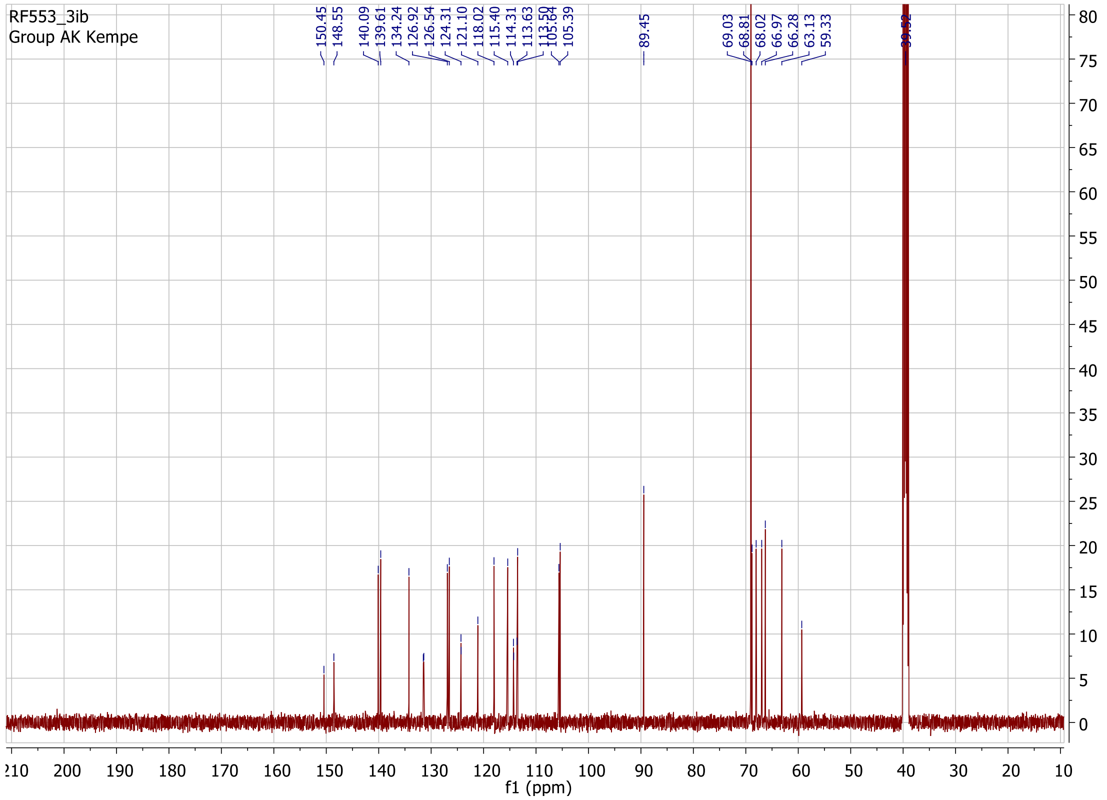

Supplementary Figure 172 ^{13}C NMR spectrum of compound **B3e**. (125 MHz, 293 K, DMSO-d_6).

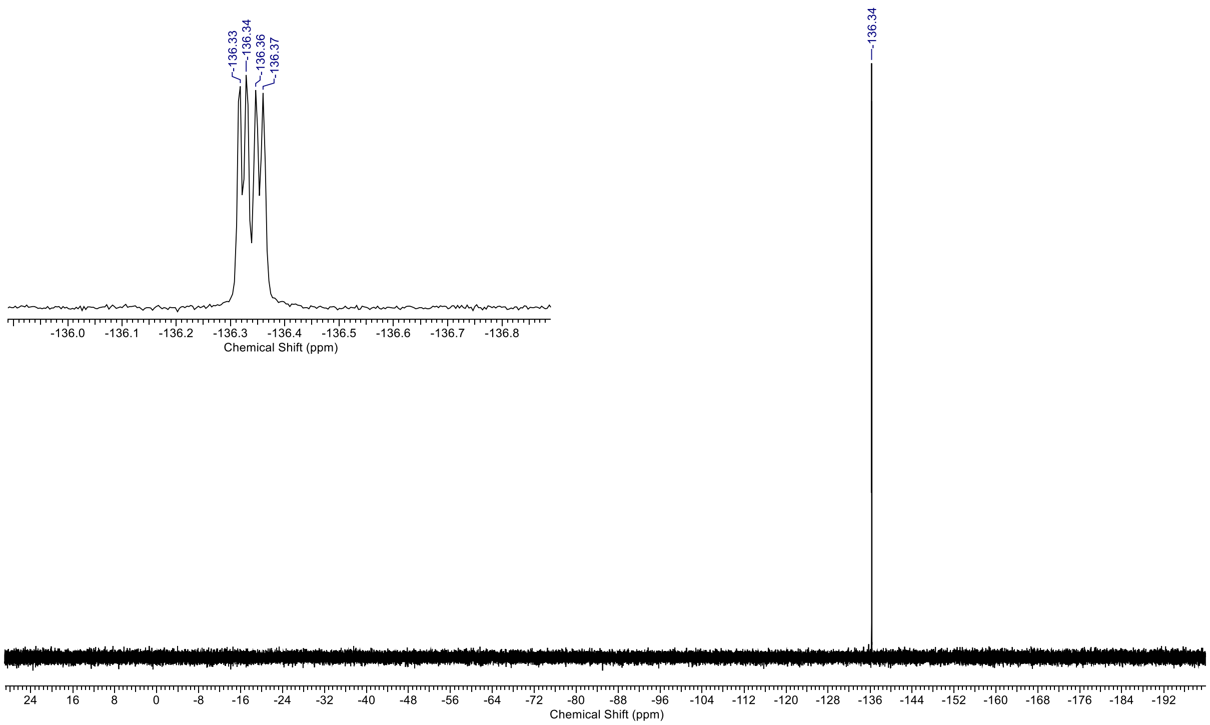

Supplementary Figure 173 ^{19}F NMR spectrum of compound **B3e**. (376 MHz, 293 K, DMSO-d_6).

NMR spectra of B4a

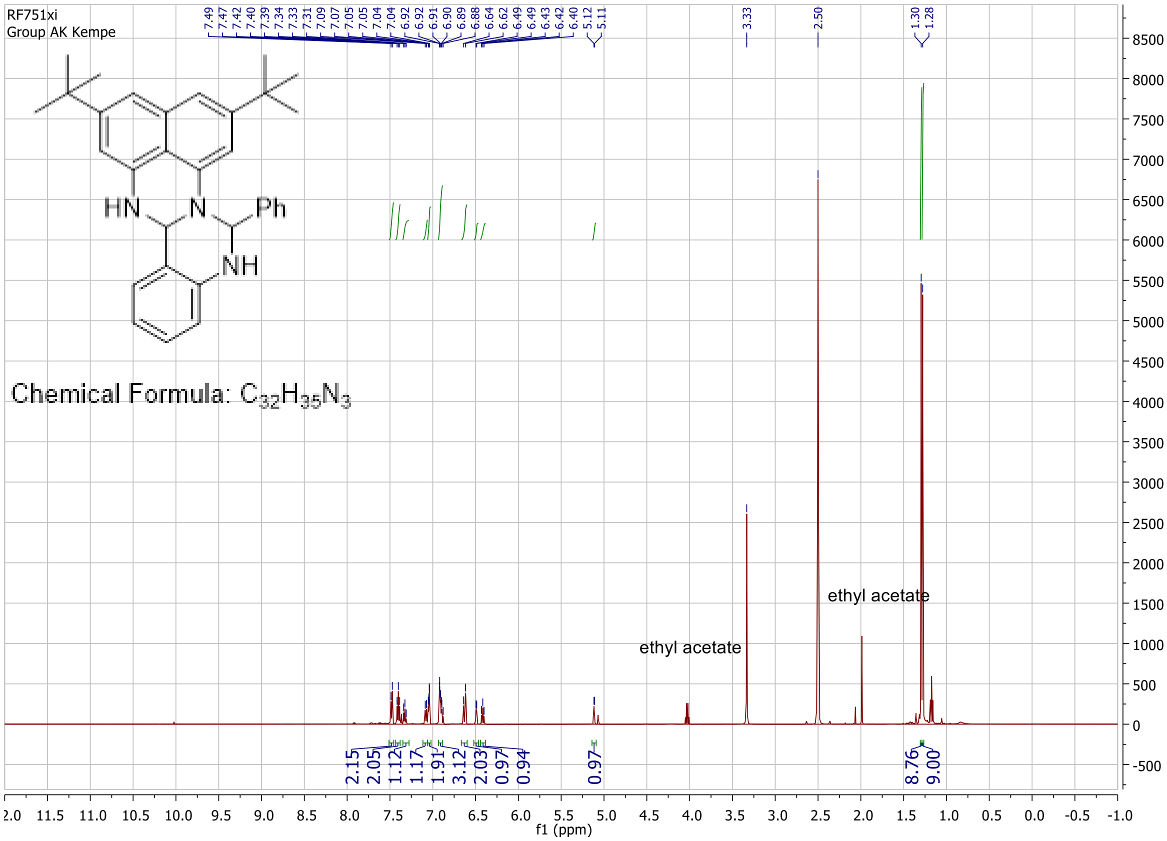

Supplementary Figure 174 ^{1}H NMR spectrum of compound **B4a**. (500 MHz, 293 K, DMSO-d_6).

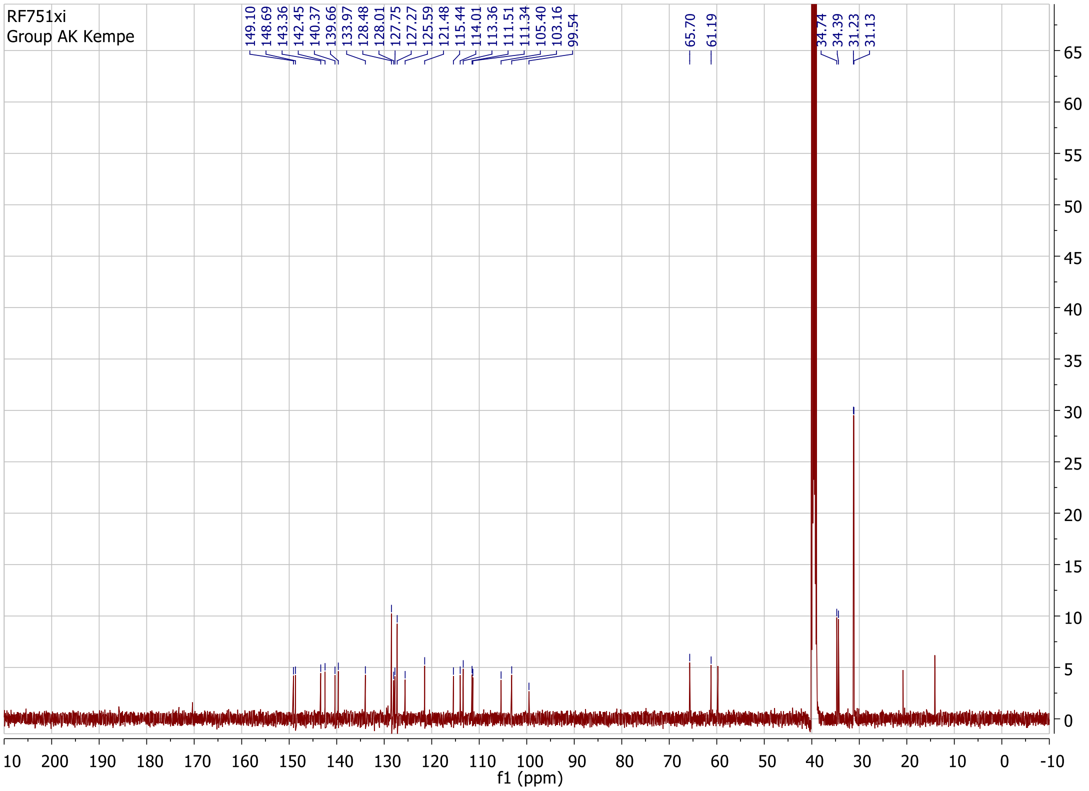

Supplementary Figure 175 ^{13}C NMR spectrum of compound **B4a**. (125 MHz, 293 K, DMSO-d_6).

NMR spectra of B4b

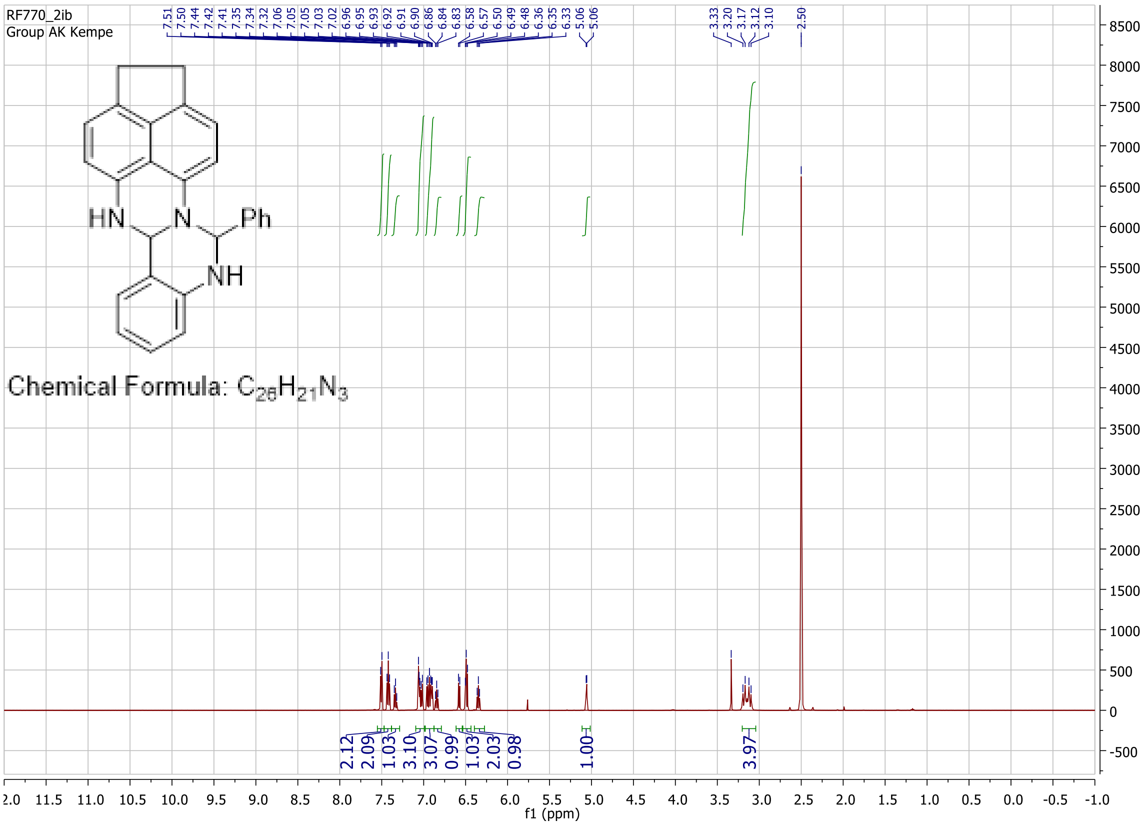

Supplementary Figure 176 ^{1}H NMR spectrum of compound **B4b**. (500 MHz, 293 K, DMSO-d_6).

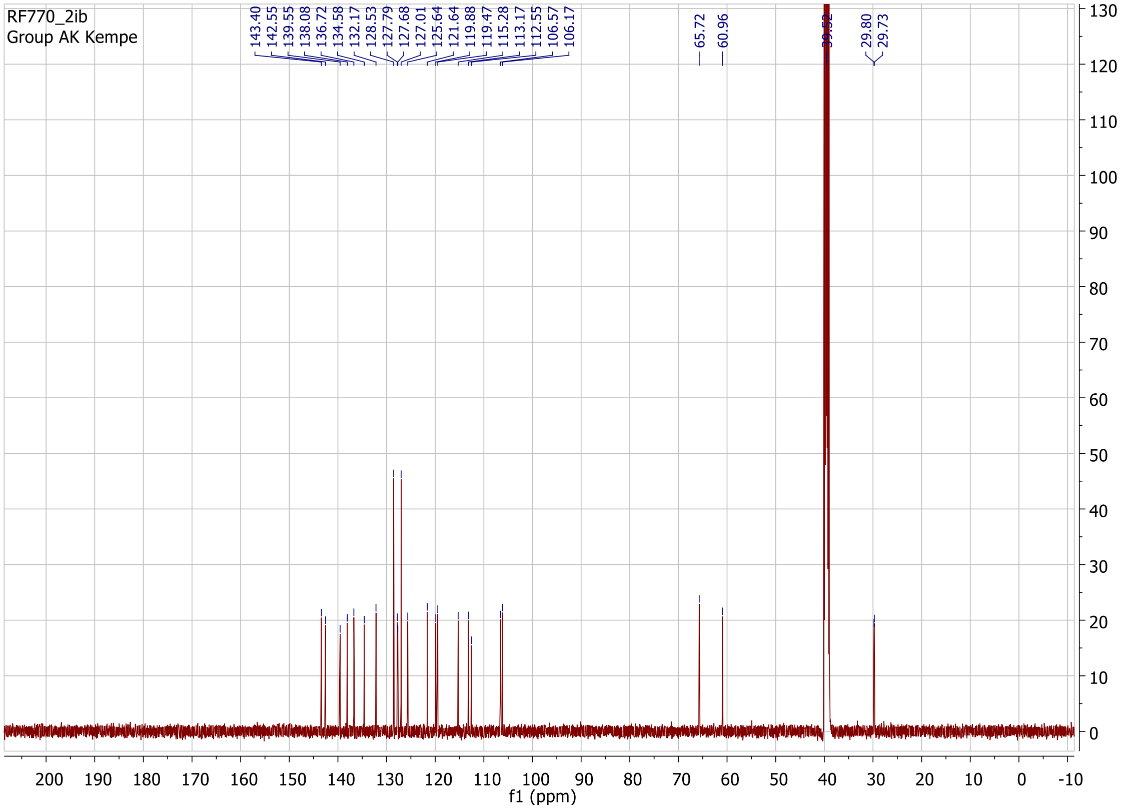

Supplementary Figure 177 ^{13}C NMR spectrum of compound **B4b**. (125 MHz, 293 K, DMSO-d_6).

NMR spectra of B4c

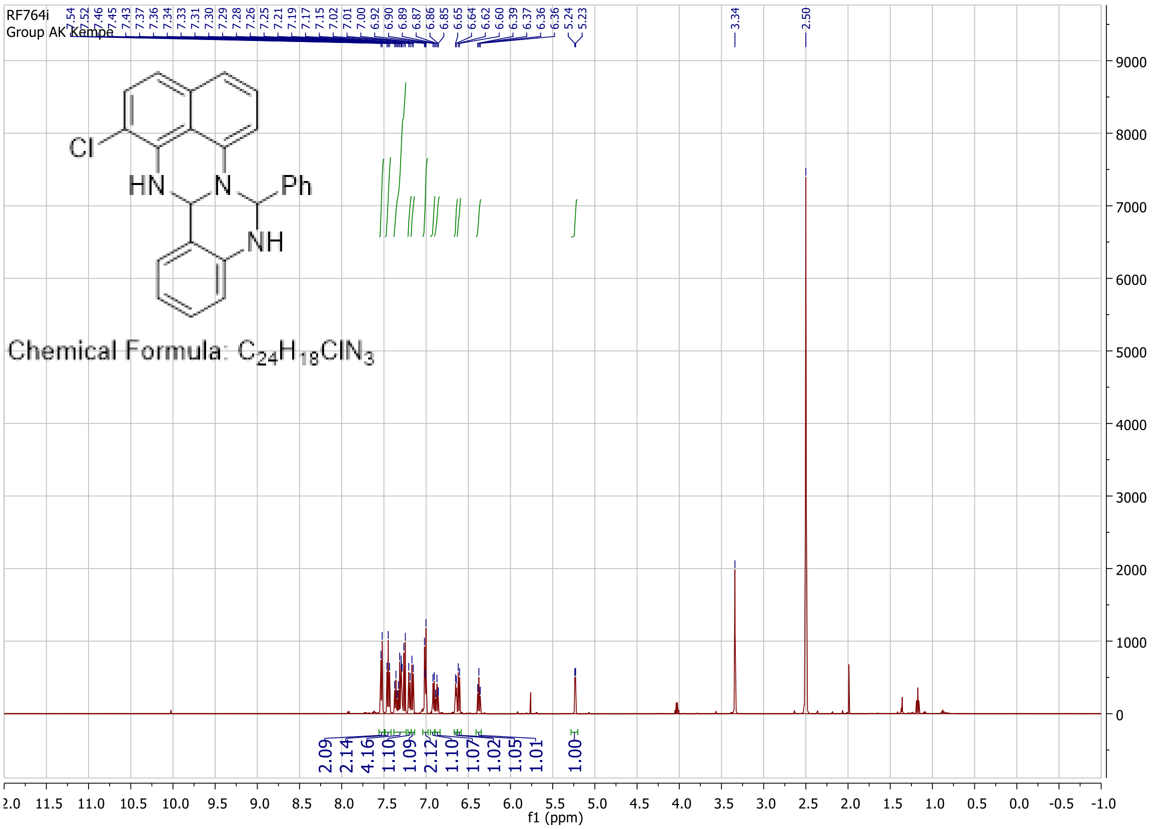

Supplementary Figure 178 ^{1}H NMR spectrum of compound **B4c**. (500 MHz, 293 K, DMSO-d_6).

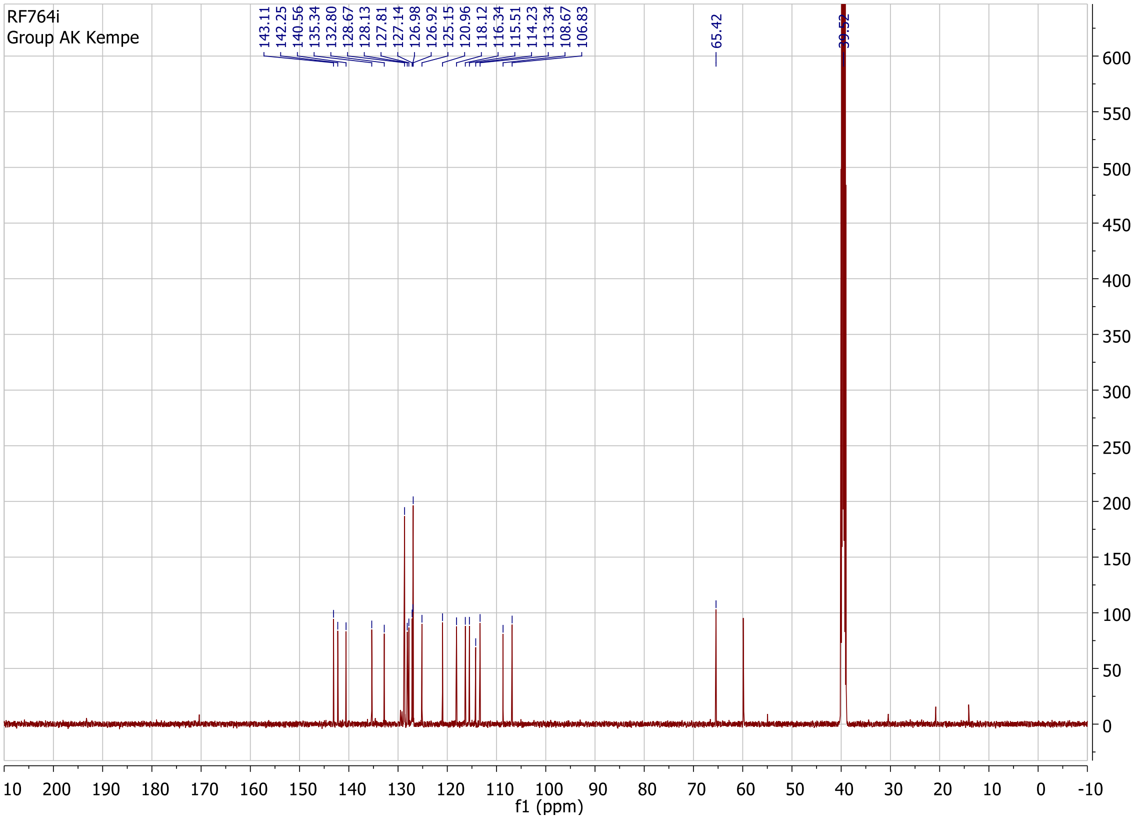

Supplementary Figure 179 ^{13}C NMR spectrum of compound **B4c**. (125 MHz, 293 K, DMSO-d_6).

NMR spectra of B5a

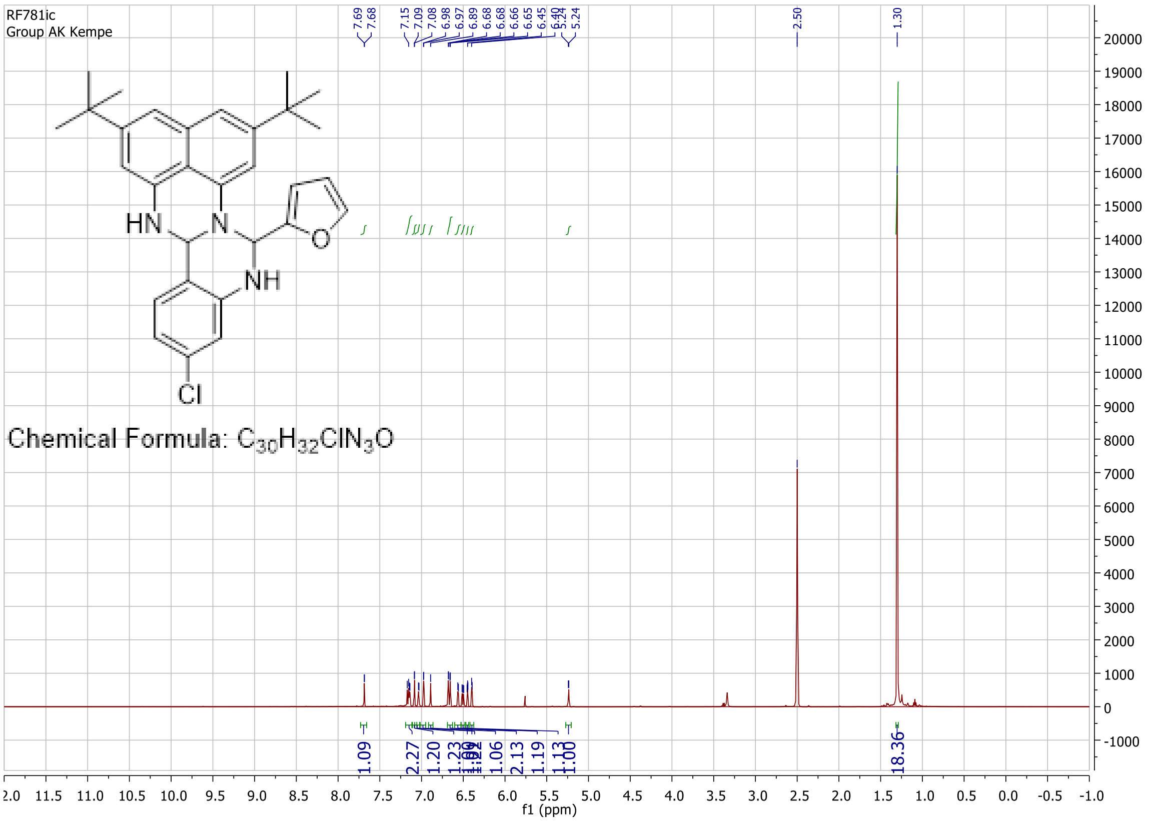

Supplementary Figure 180 ^{1}H NMR spectrum of compound **B5a**. (500 MHz, 293 K, DMSO-d_6).

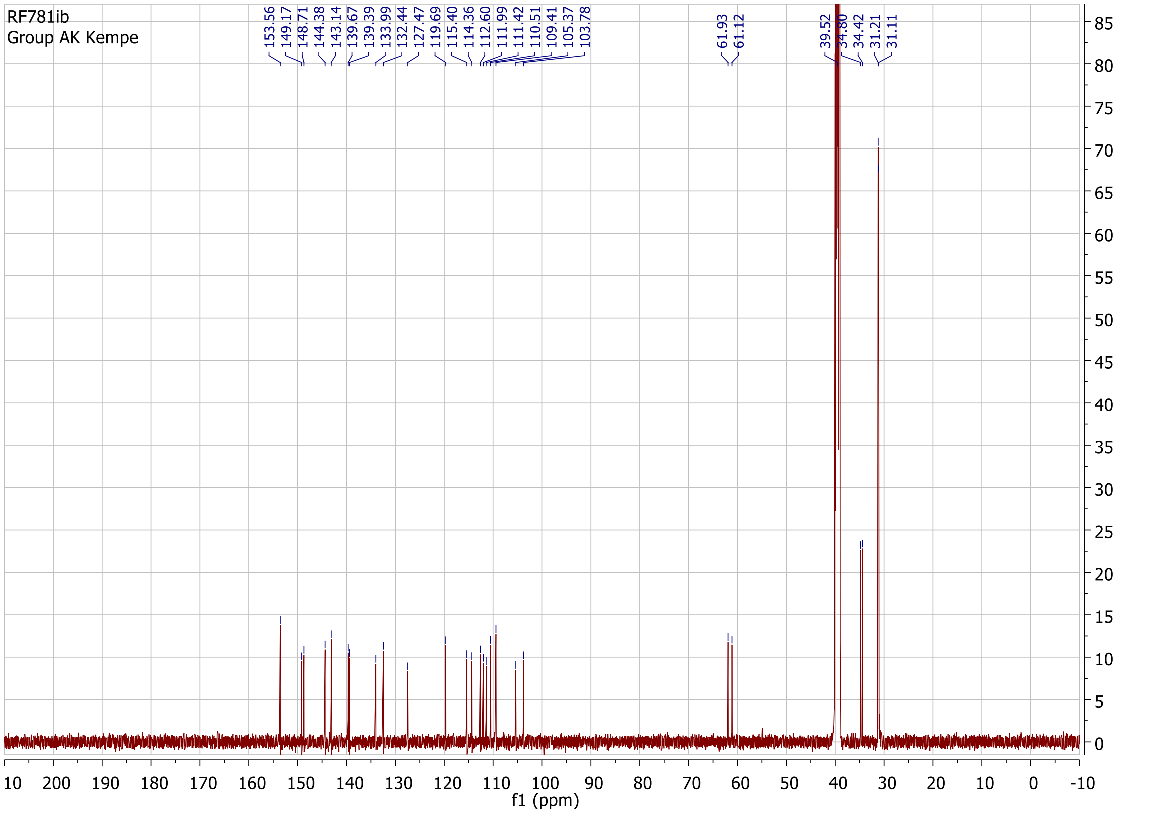

Supplementary Figure 181 ^{13}C NMR spectrum of compound **B5a**. (125 MHz, 293 K, DMSO-d_6).

NMR spectra of B5b

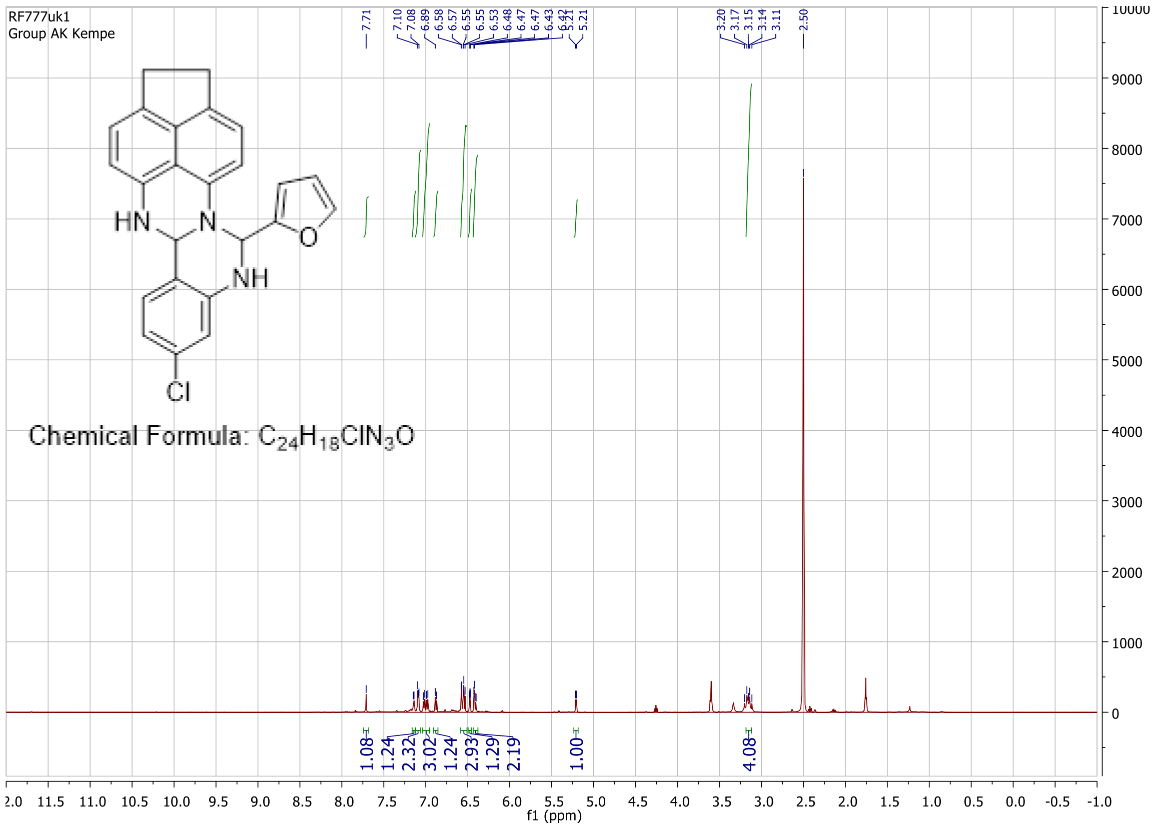

Supplementary Figure 182 ^{1}H NMR spectrum of compound **B5b**. (500 MHz, 293 K, DMSO-d$_6$).

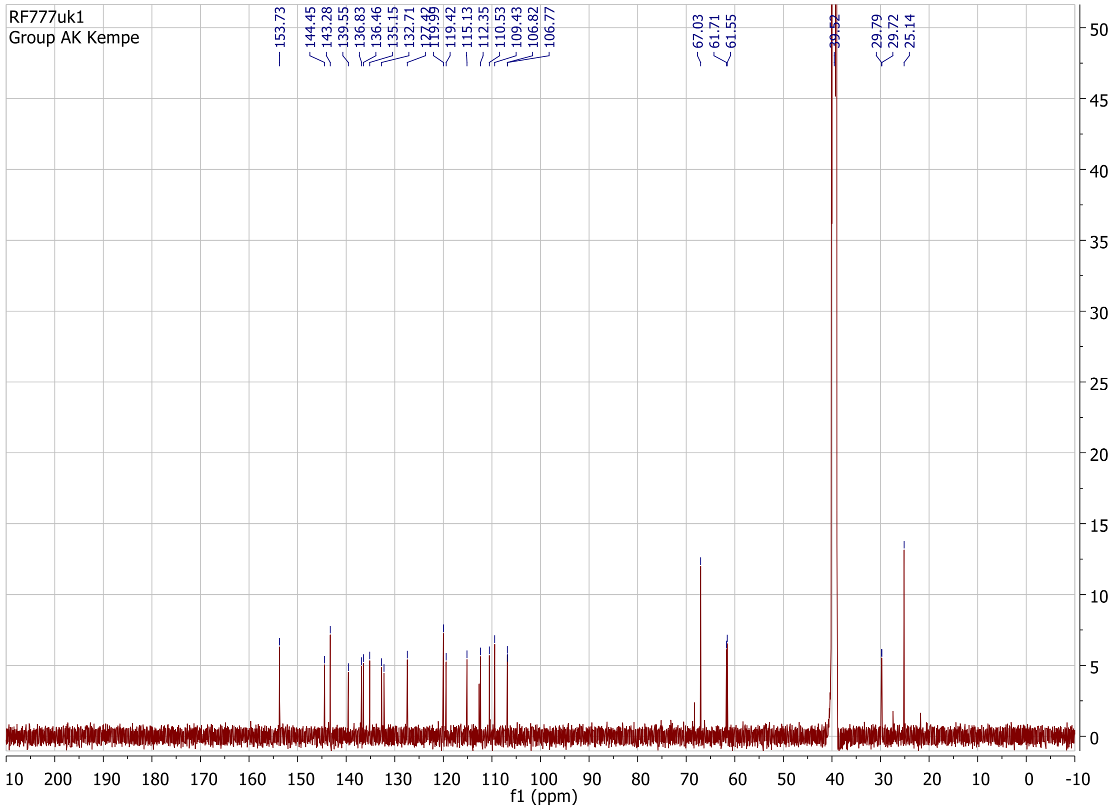

Supplementary Figure 183 ^{13}C NMR spectrum of compound **B5b**. (125 MHz, 293 K, DMSO-d$_6$).

NMR spectra of B5c

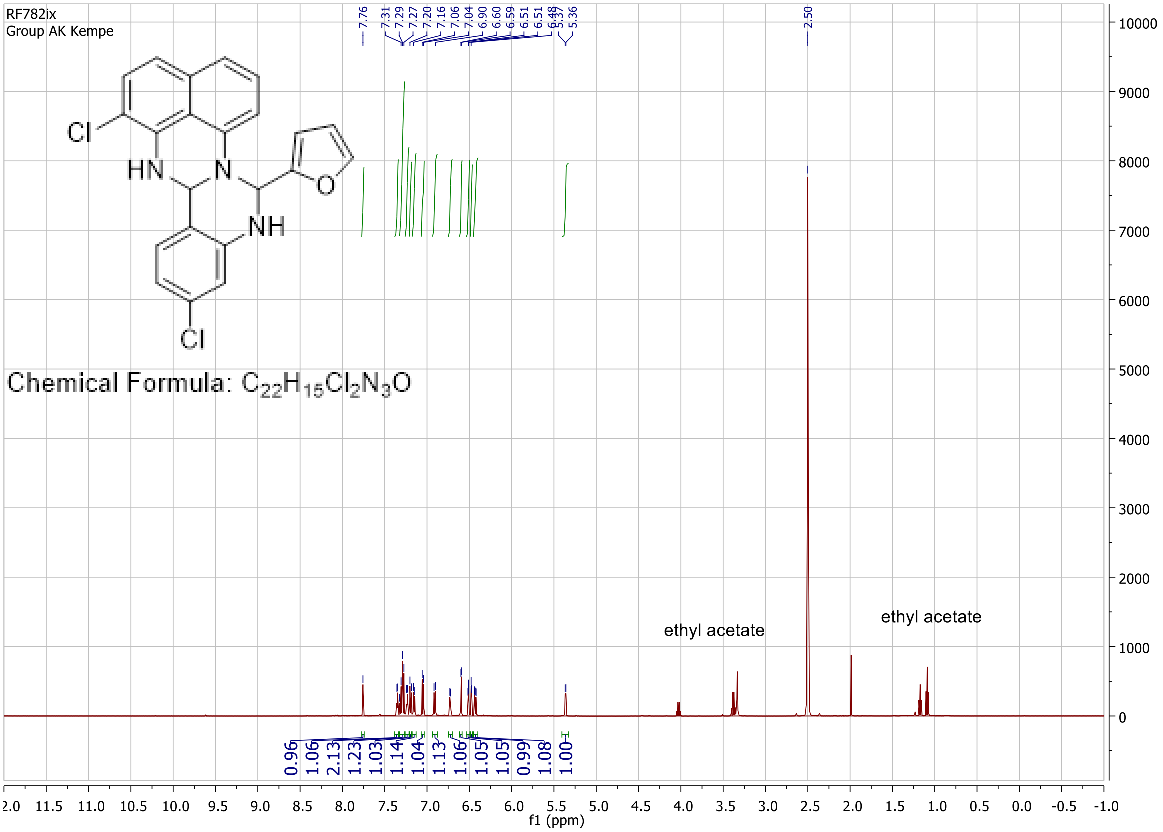

Supplementary Figure 184 ^{1}H NMR spectrum of compound **B5c**. (500 MHz, 293 K, DMSO-d_6).

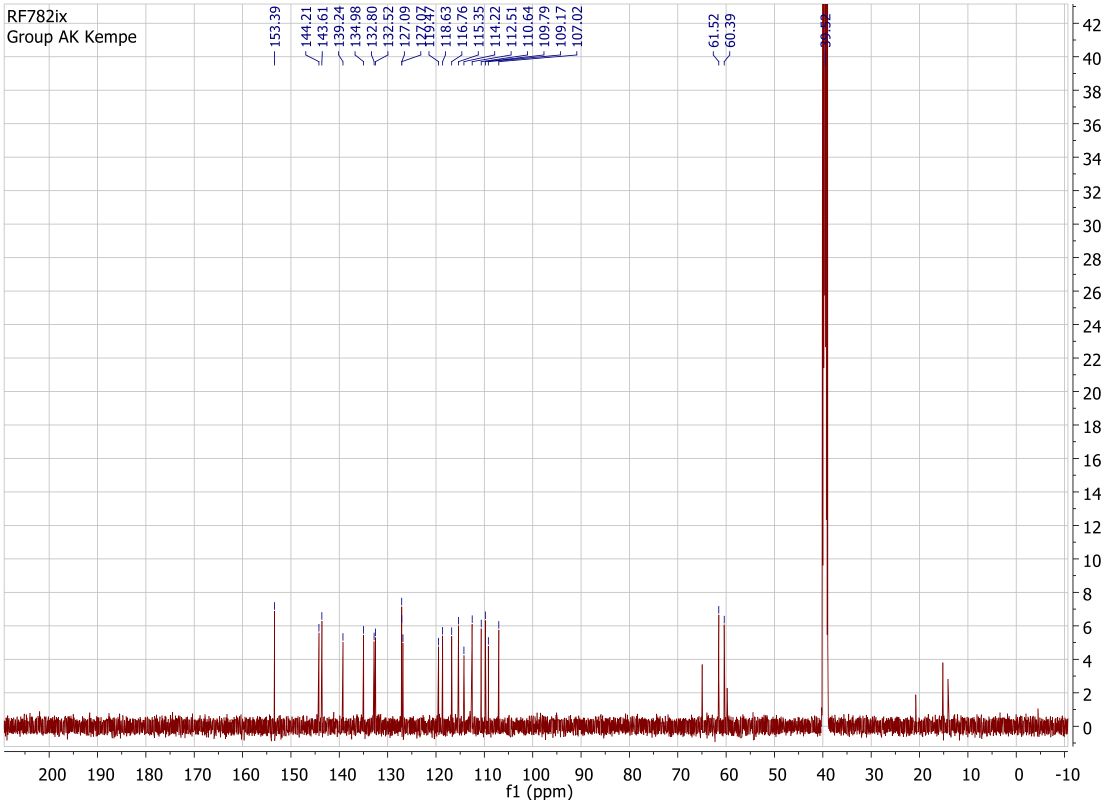

Supplementary Figure 185 ^{13}C NMR spectrum of compound **B5c**. (125 MHz, 293 K, DMSO-d_6).

NMR spectra of B6a

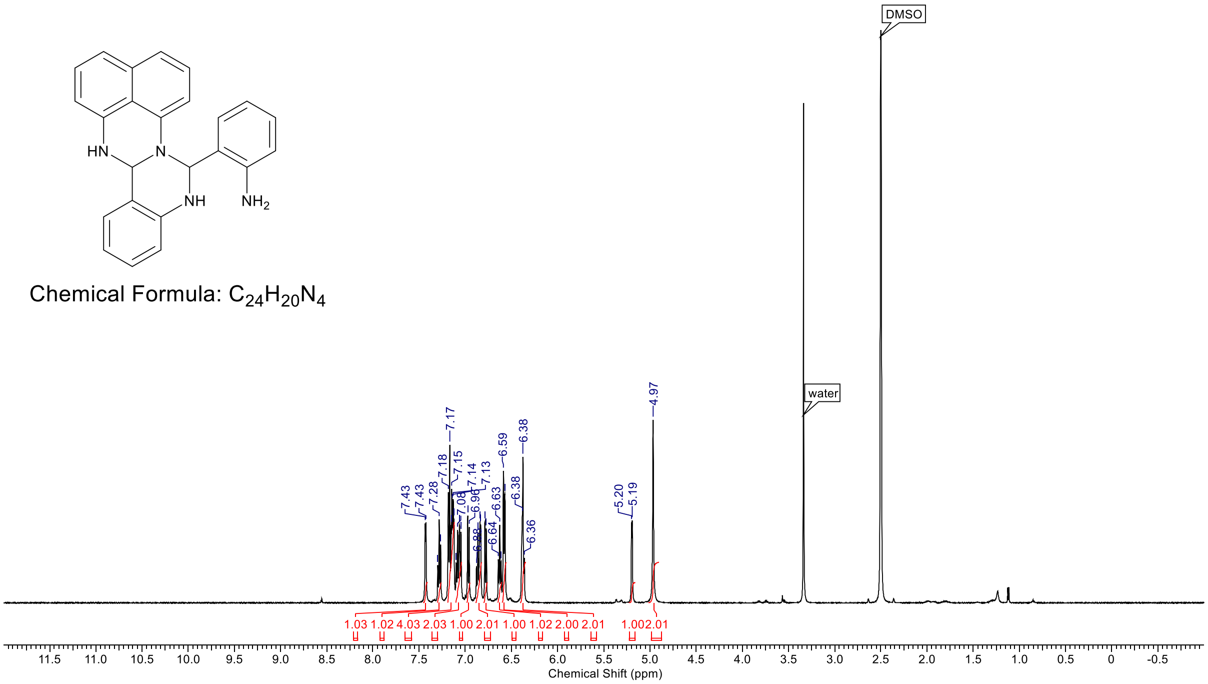

Supplementary Figure 186 ^{1}H NMR spectrum of compound **B6a**. (500 MHz, 293 K, DMSO-d_6).

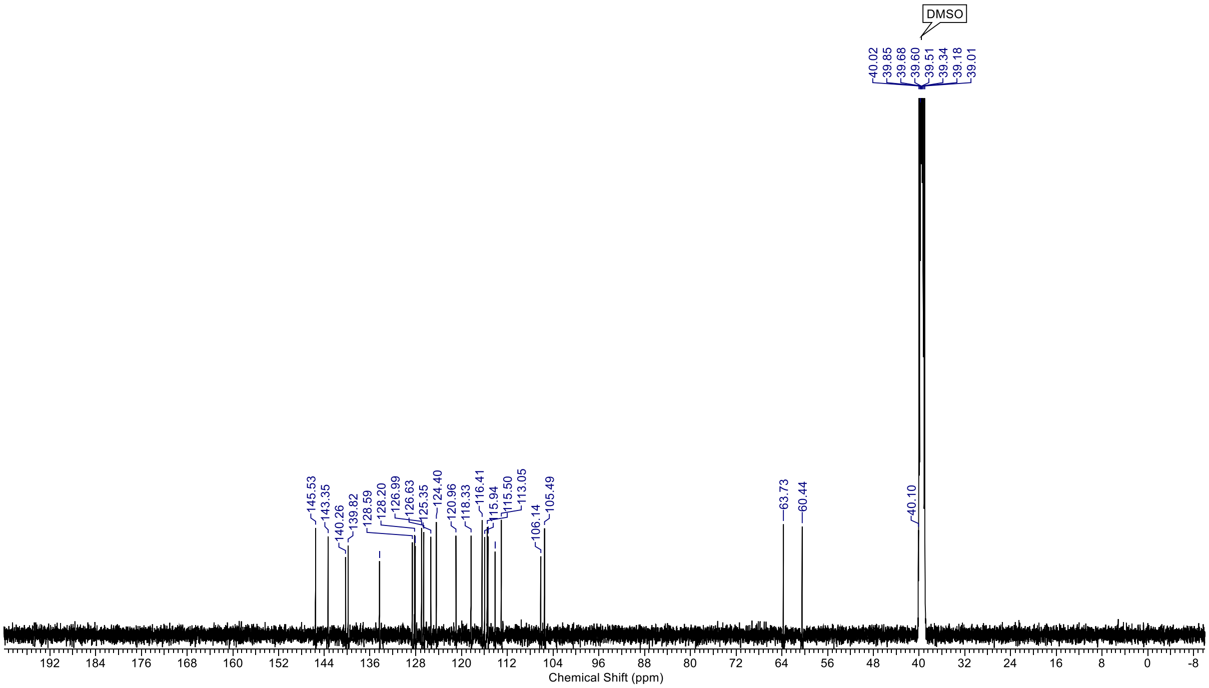

Supplementary Figure 187 ^{13}C NMR spectrum of compound **B6a**. (125 MHz, 293 K, DMSO-d_6).

NMR spectra of B6b

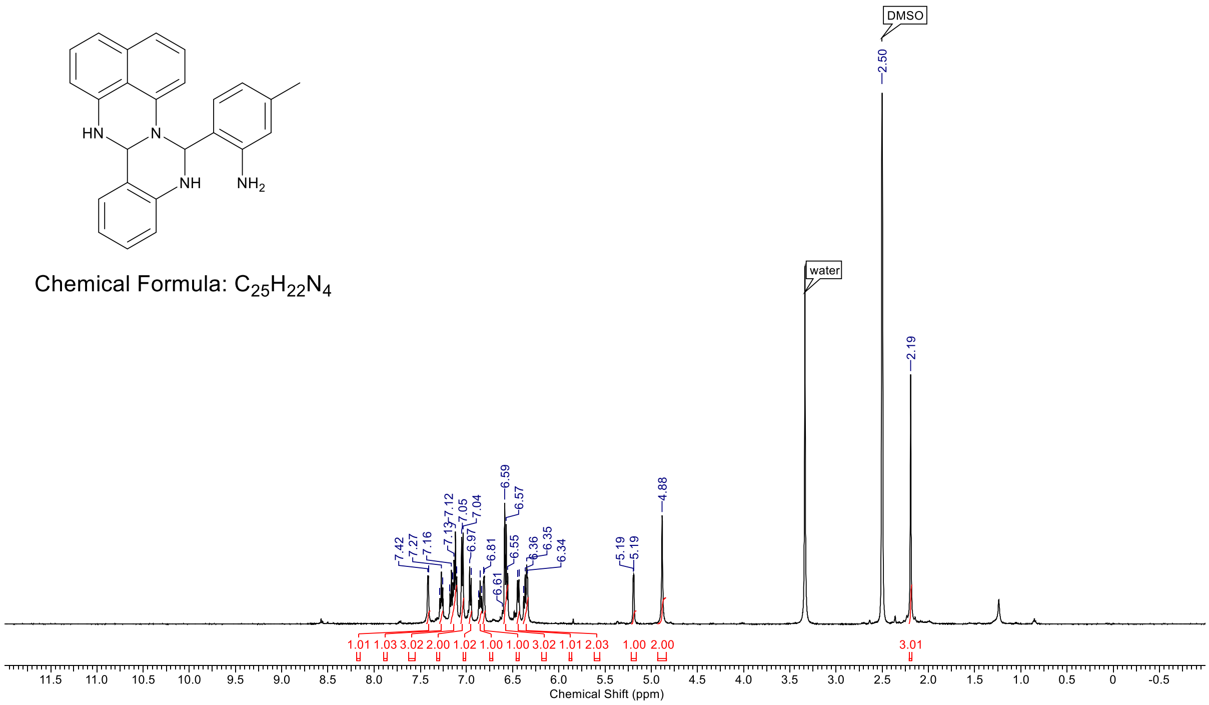

Supplementary Figure 188 ^{1}H NMR spectrum of compound **B6b**. (500 MHz, 293 K, DMSO-d_6).

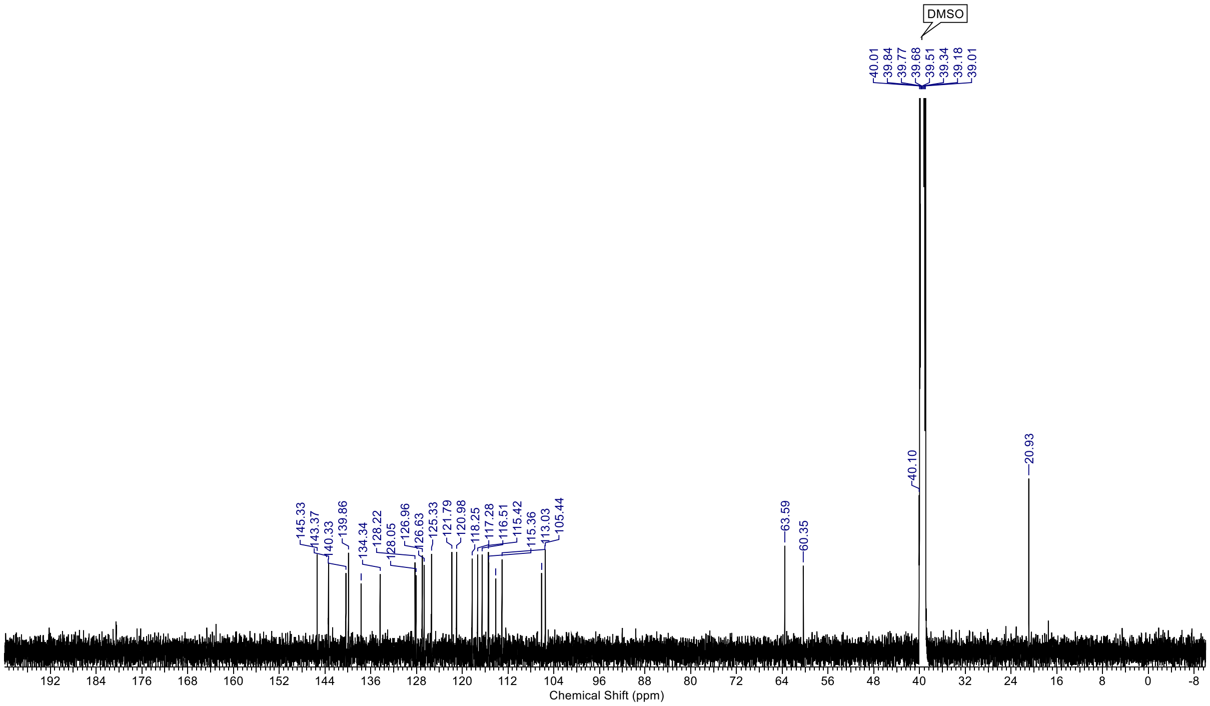

Supplementary Figure 189 ^{13}C NMR spectrum of compound **B6b**. (125 MHz, 293 K, DMSO-d_6).

NMR spectra of B6c

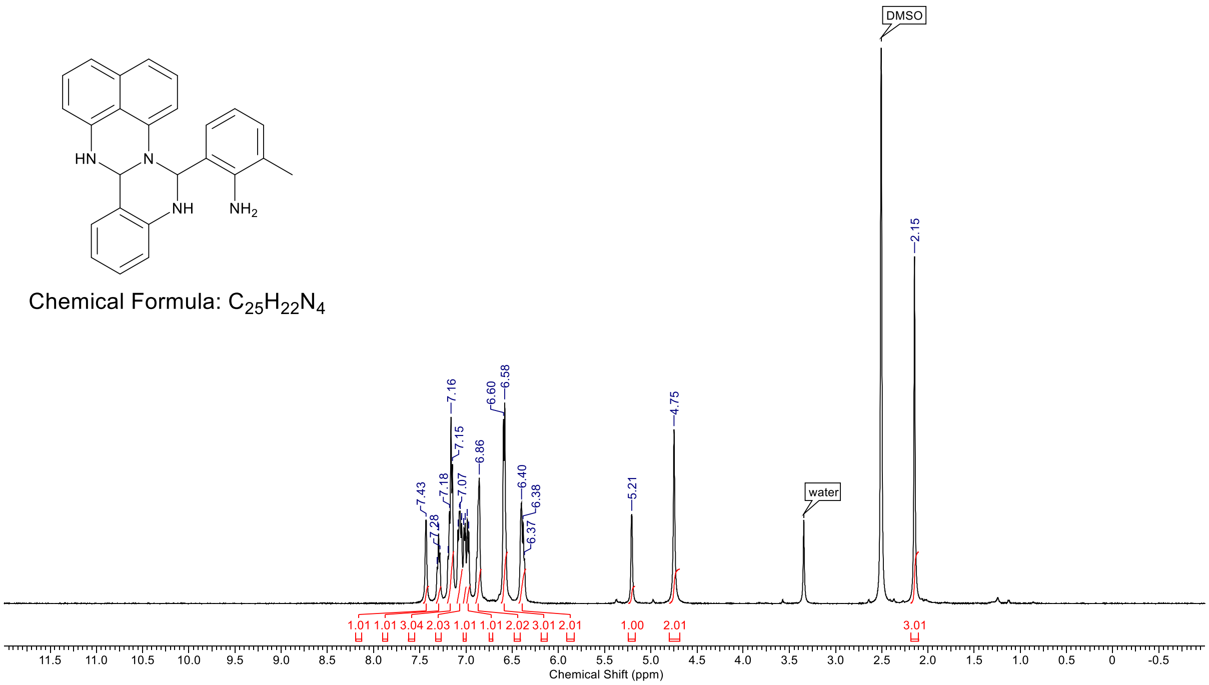

Supplementary Figure 190 ^{1}H NMR spectrum of compound **B6c**. (500 MHz, 293 K, DMSO-d_6).

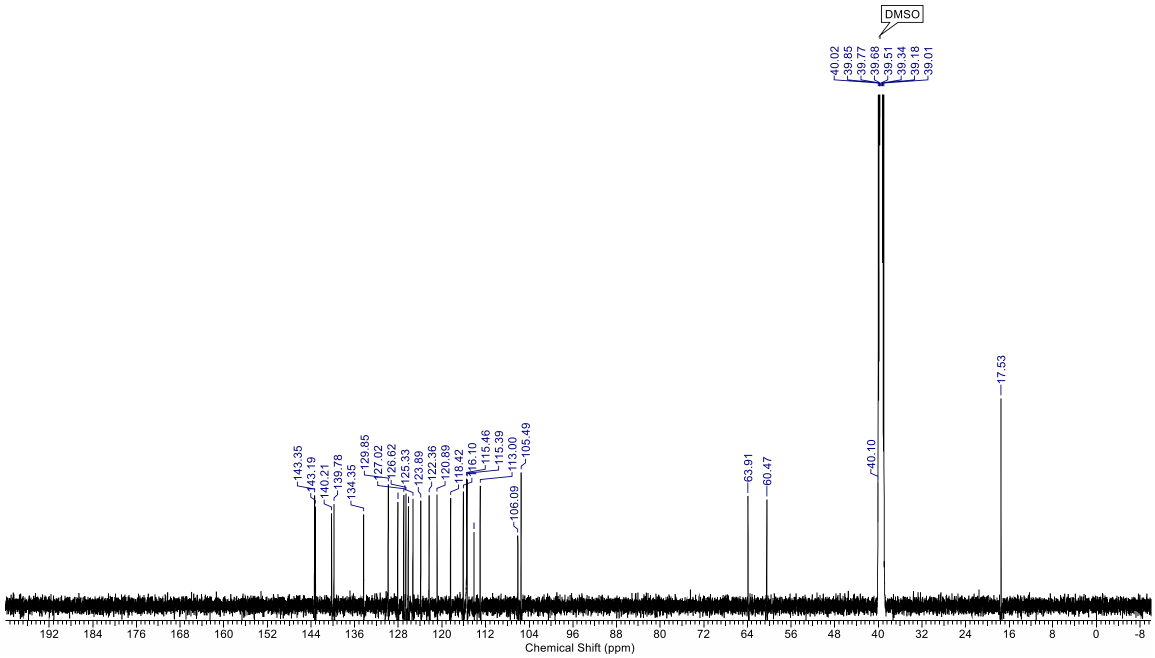

Supplementary Figure 191 ^{13}C NMR spectrum of compound **B6c**. (125 MHz, 293 K, DMSO-d_6).

NMR spectra of C1

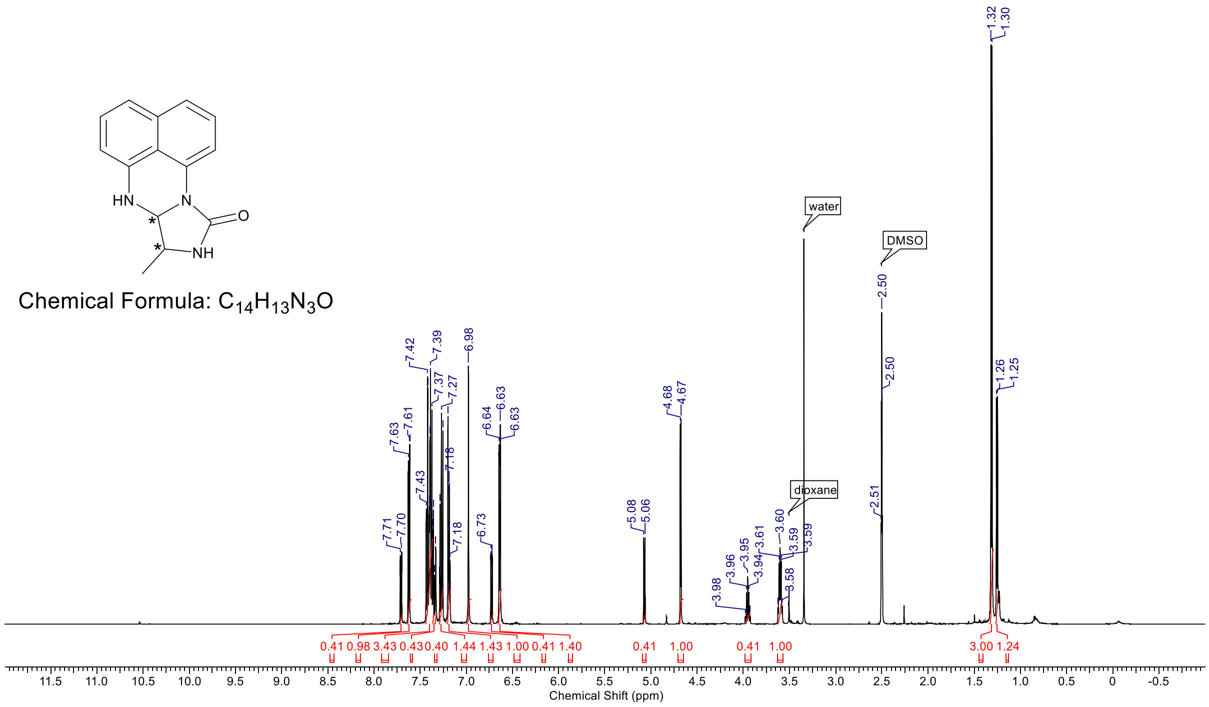

Supplementary Figure 192 ^{1}H NMR spectrum of compound **C1**. (500 MHz, 293 K, DMSO-d_6).

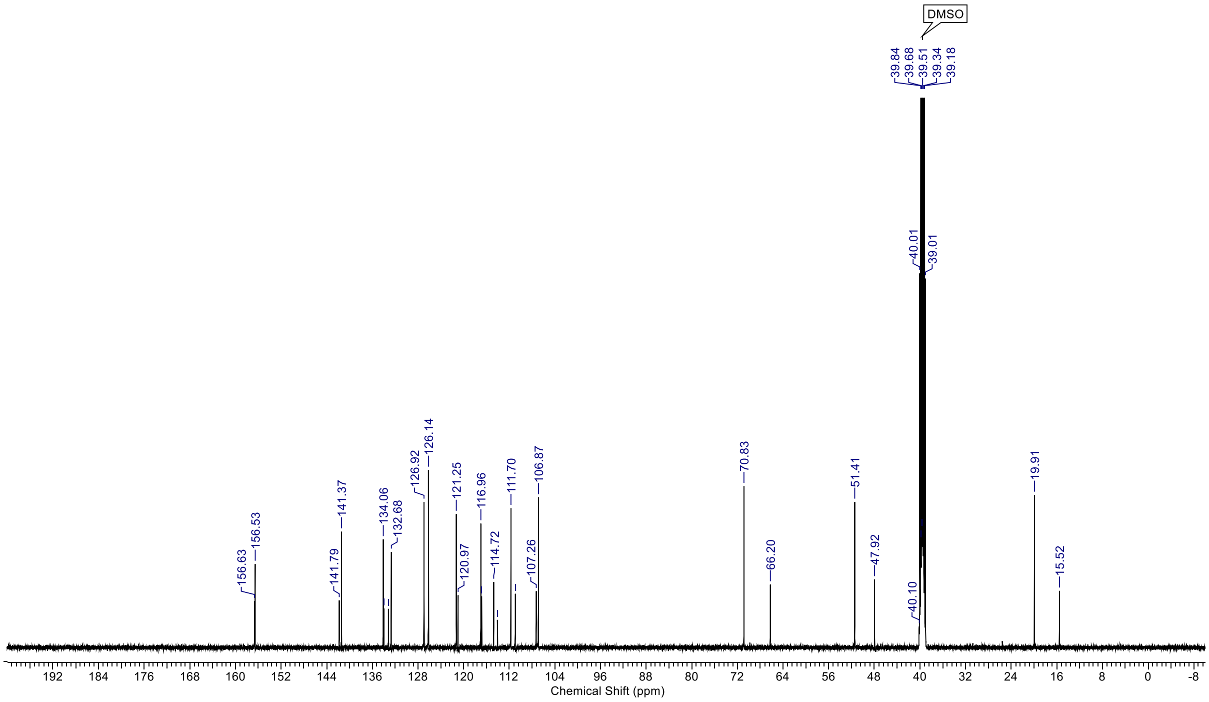

Supplementary Figure 193 ^{13}C NMR spectrum of compound **C1**. (125 MHz, 293 K, DMSO-d_6).

NMR spectra of C2

Main isomer of **C2**:

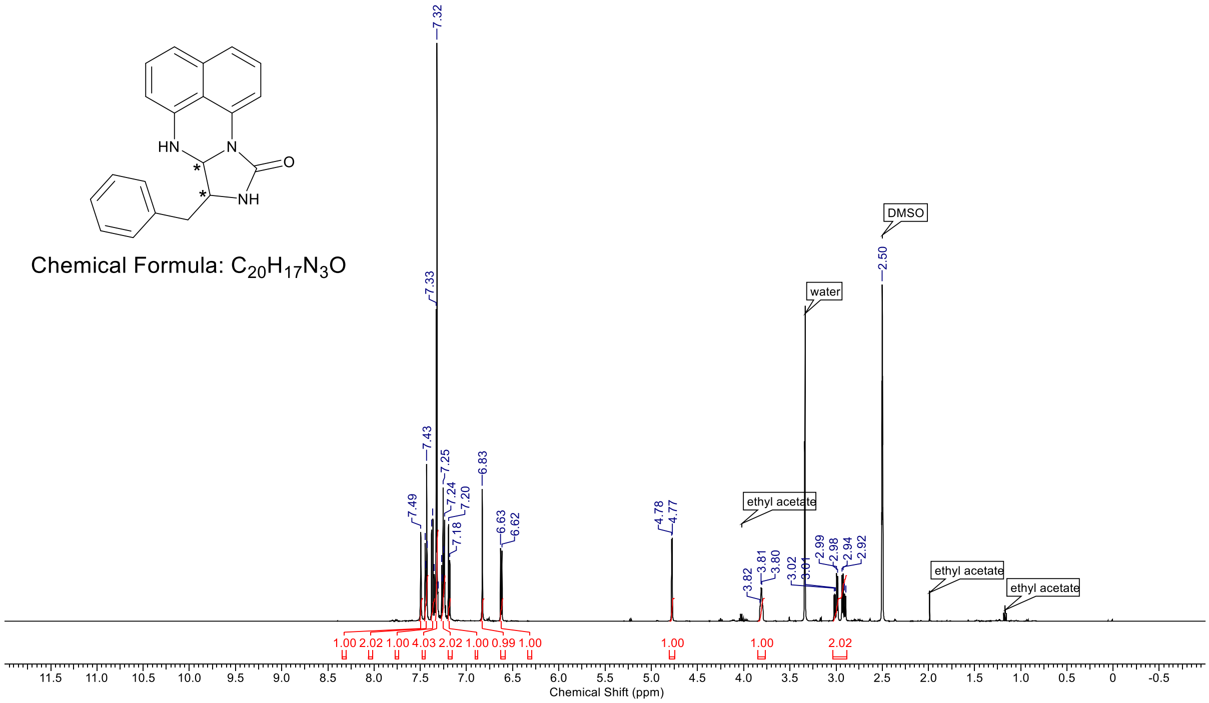

Supplementary Figure 194 ^{1}H NMR spectrum of the main isomer of compound **C2**. (500 MHz, 293 K, DMSO-d$_6$).

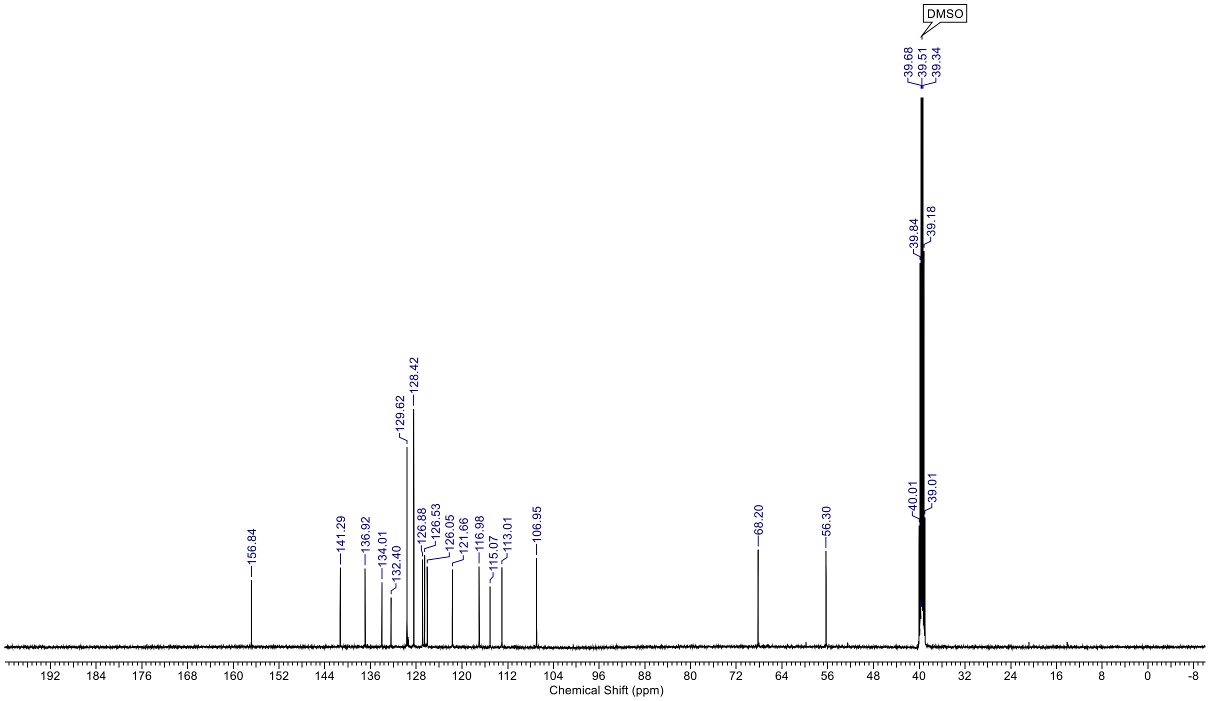

Supplementary Figure 195 ^{13}C NMR spectrum of the main isomer of compound **C2**. (125 MHz, 293 K, DMSO-d$_6$).

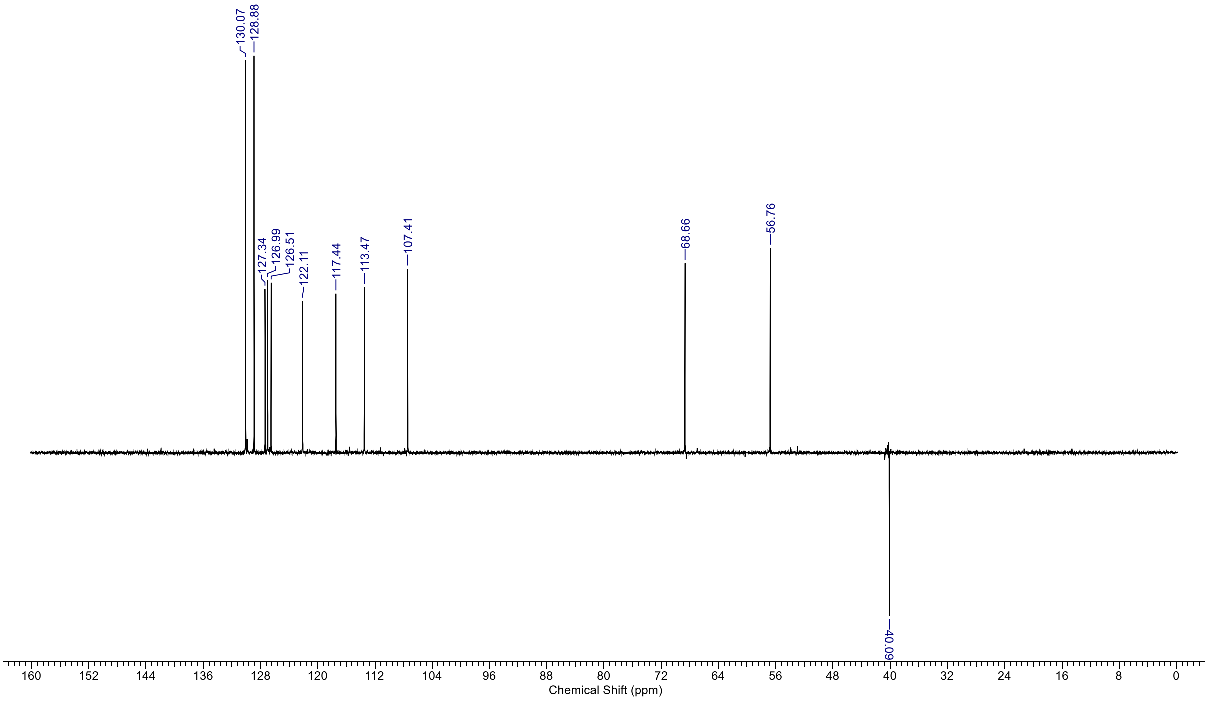

Supplementary Figure 196 DEPT 135 NMR spectrum of the main isomer of compound **C2**. (500 MHz, 293 K, DMSO-d_6).

Minor isomer of **C2**:

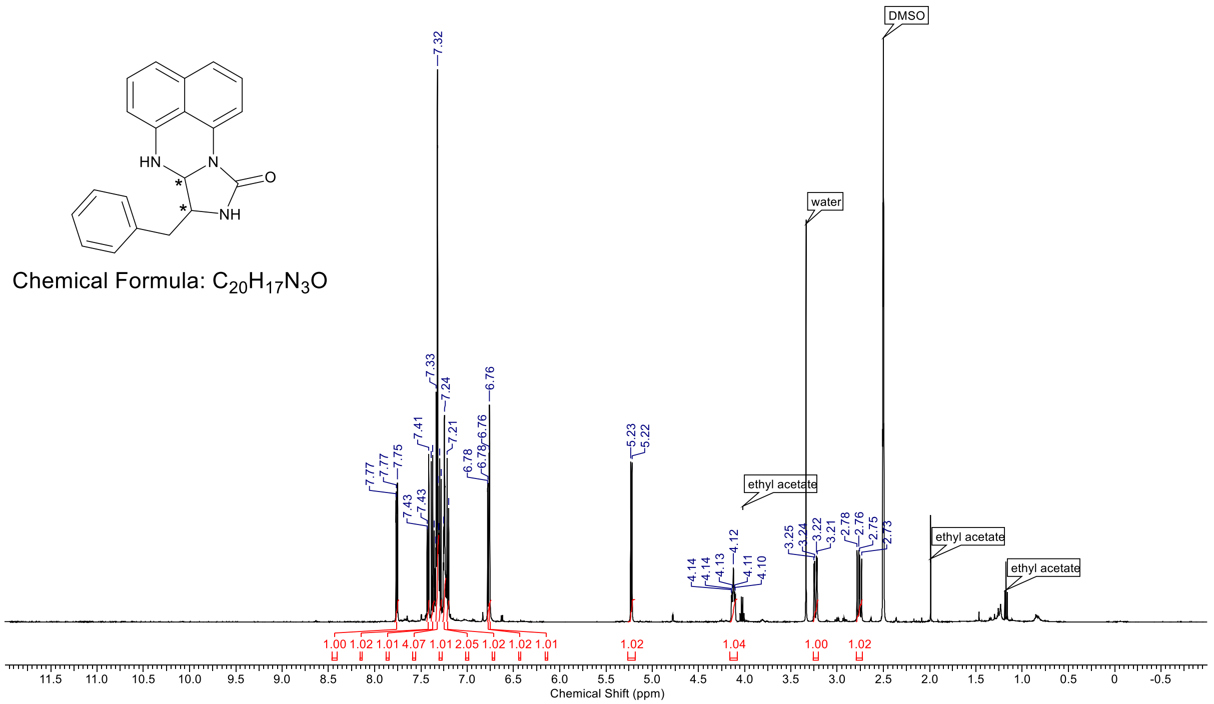

Supplementary Figure 197 ^{1}H NMR spectrum of the minor isomer of compound **C2**. (500 MHz, 293 K, DMSO-d_6).

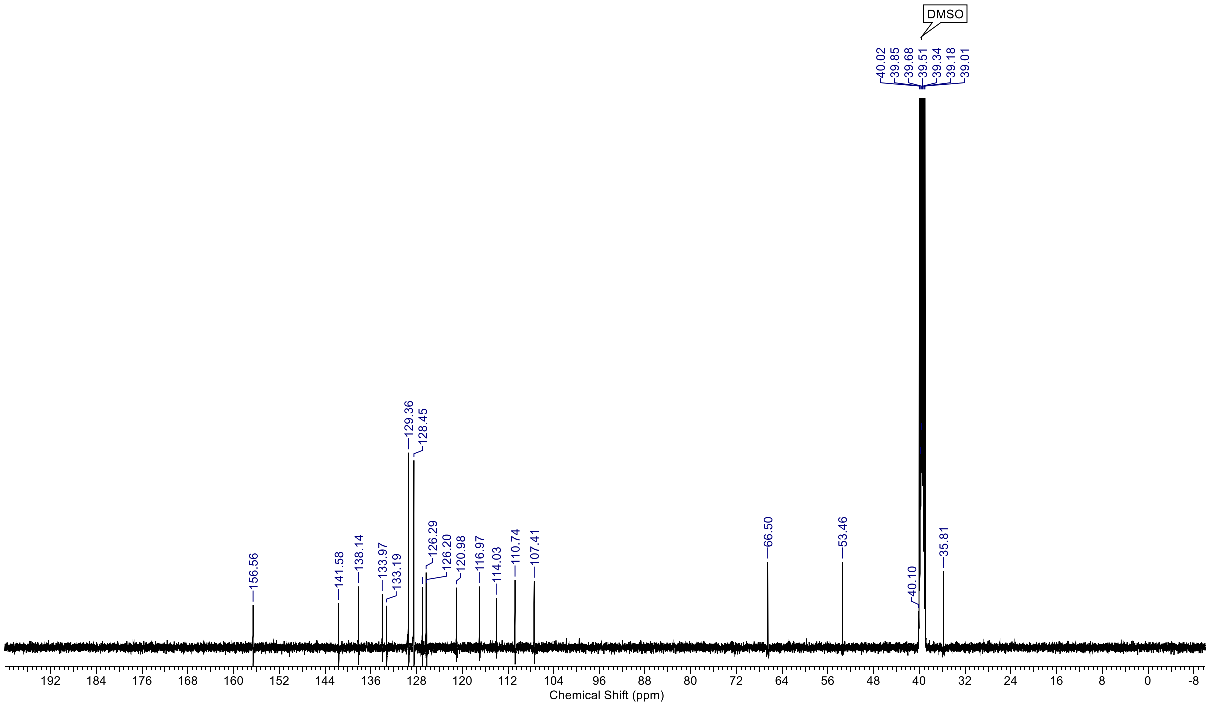

Supplementary Figure 198 ^{13}C NMR spectrum of the minor isomer of compound **C2**. (125 MHz, 293 K, DMSO-d_6).

NMR spectra of C3

Main isomer of **C3**:

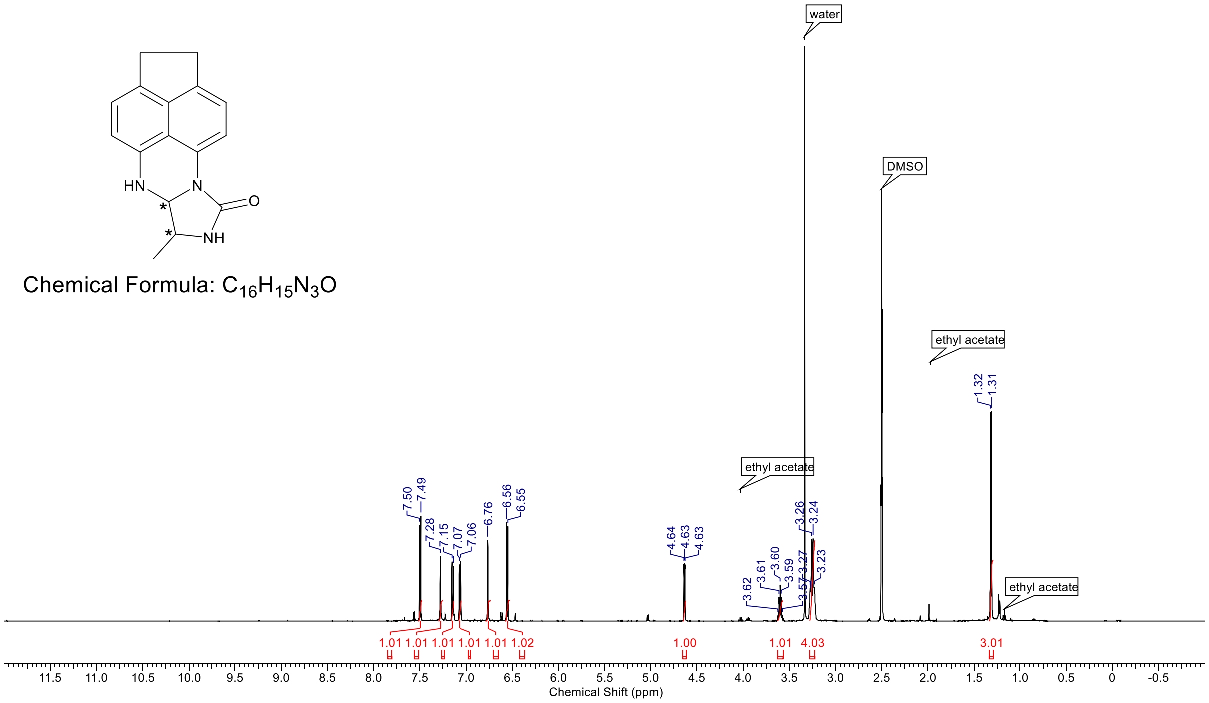

Supplementary Figure 199 ^{1}H NMR spectrum of the main isomer of compound **C3**. (500 MHz, 293 K, DMSO-d$_6$).

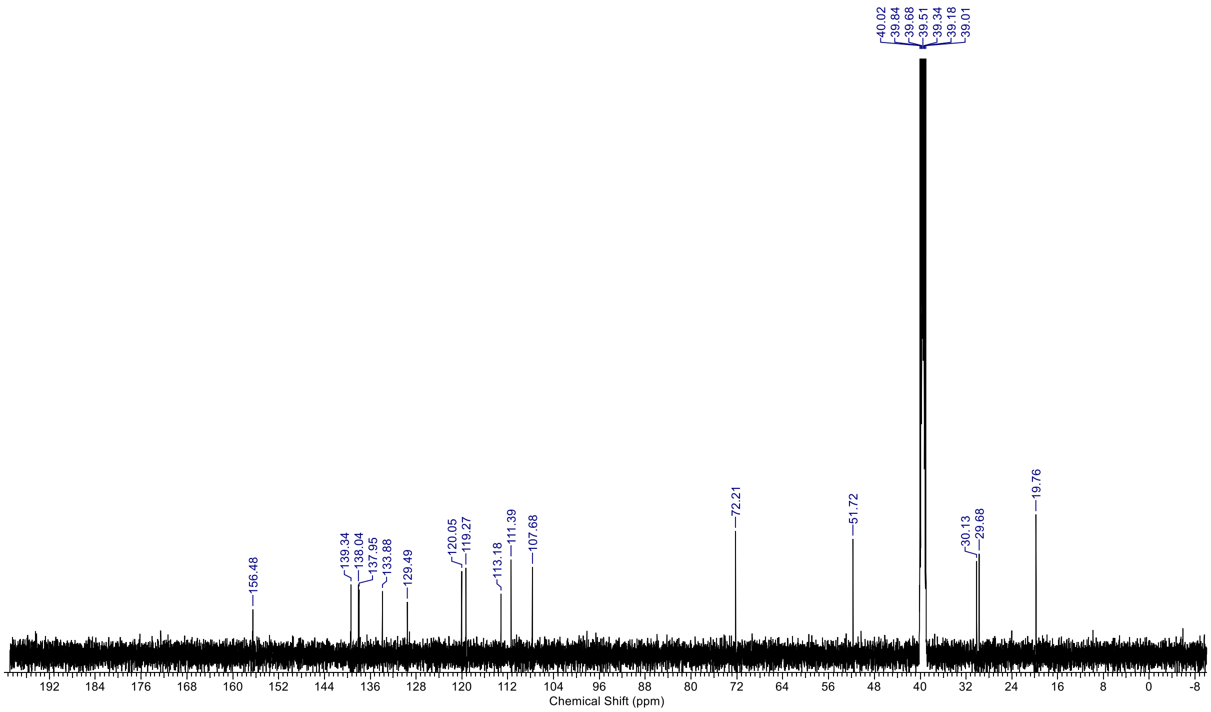

Supplementary Figure 200 ^{13}C NMR spectrum of the main isomer of compound **C3**. (125 MHz, 293 K, DMSO-d$_6$).

Minor isomer of **C3**:

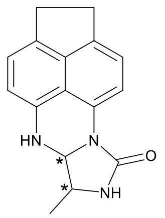

Chemical Formula: $C_{16}H_{15}N_3O$

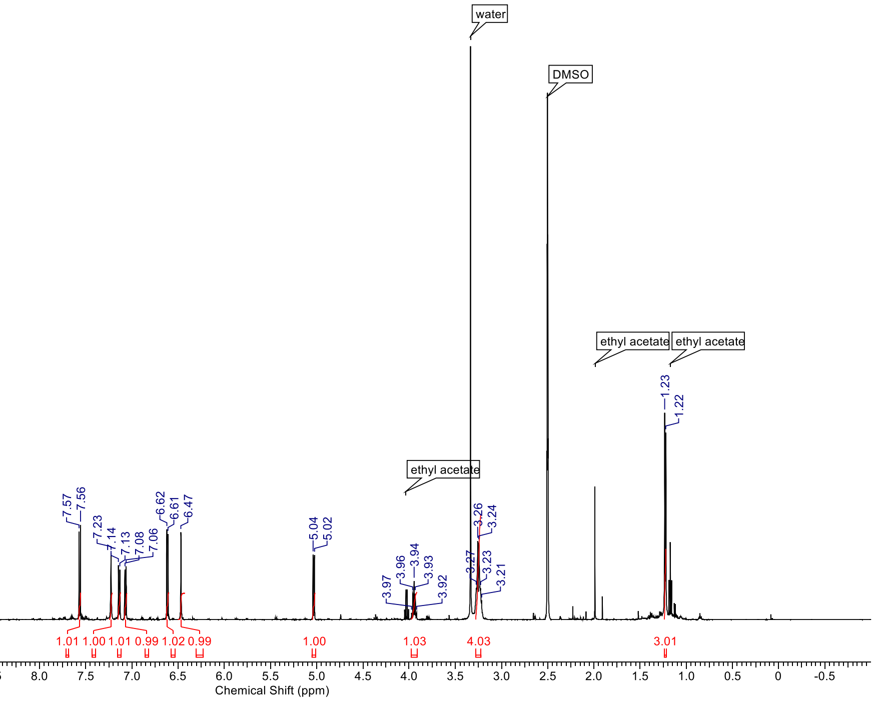

Supplementary Figure 201 ^{1}H NMR spectrum of the minor isomer of compound **C3**. (500 MHz, 293 K, DMSO-d$_6$).

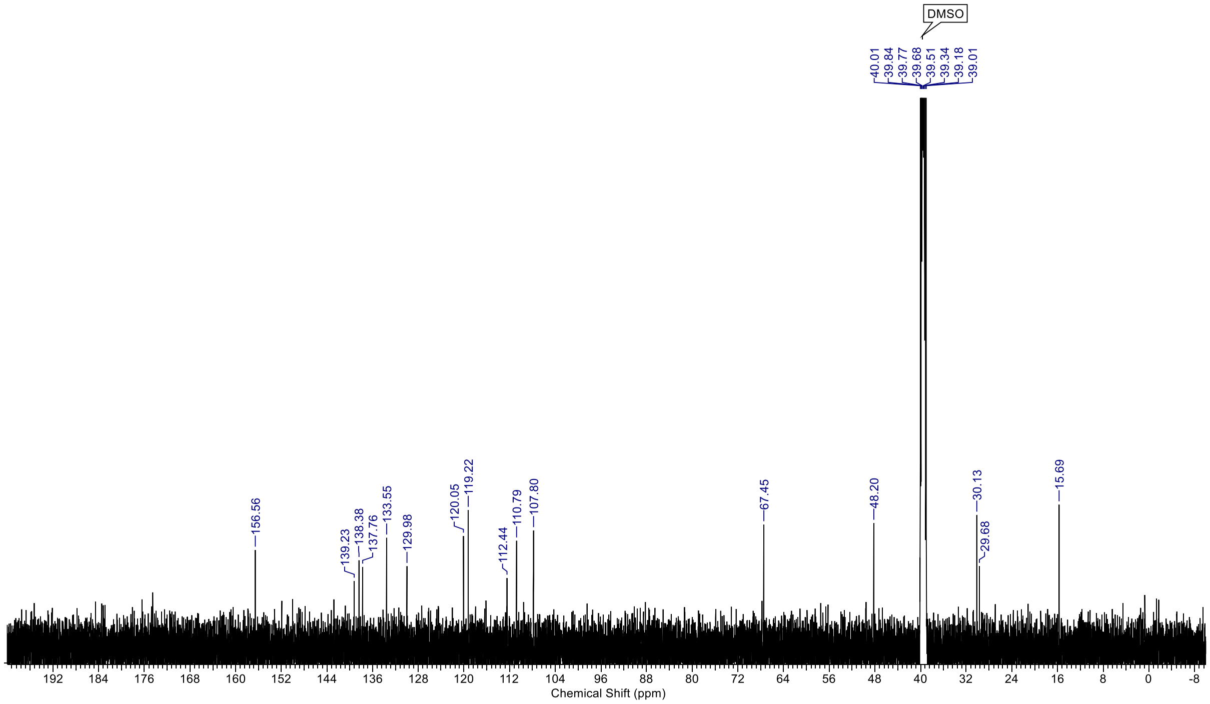

Supplementary Figure 202 ^{13}C NMR spectrum of the minor isomer of compound **C3**. (125 MHz, 293 K, DMSO-d$_6$).

15. LC-HRMS spectra

General conditions

Liquid chromatography-high resolution mass spectra (LC-HRMS) were obtained from a Thermo Fisher scientific Q-Exactive instrument with a hybrid quadrupole orbitrap analyser in ESI+ mode. For liquid chromatography a Luna Omega PS C18 (100x2.1 mm, 1.6 µm) column was used with a solvent gradient from 30:70 MeCN/water to 90:10 MeCN/water. The samples were dissolved in ethanol or DMSO.

Due to the air sensitivity of most substances and specimen preparation under air the formation of nitrosamines occurred after short period of time, which is visible in traces in some of the spectra.

LC-HRMS of A25

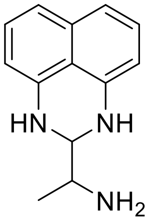

Chemical Formula: $C_{13}H_{15}N_3$

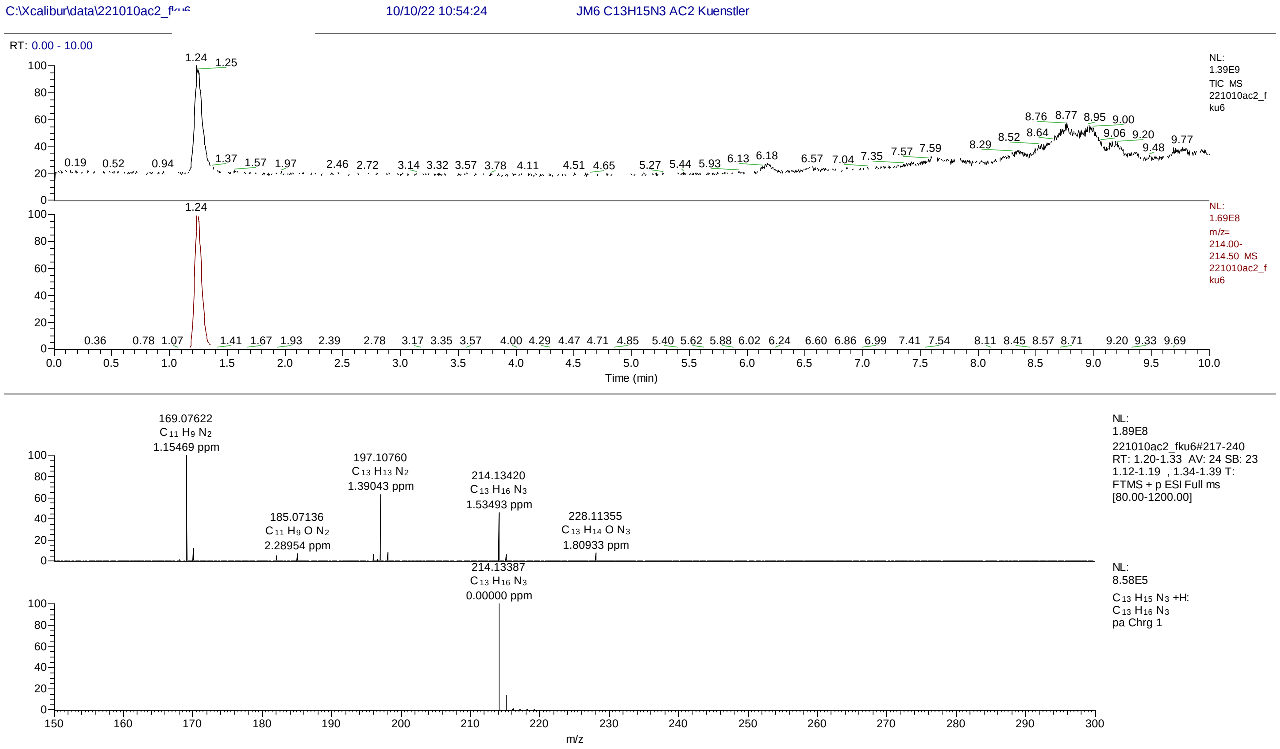

Supplementary Figure 203 LC-HRMS spectrum of compound **A25**.

LC-HRMS of A26

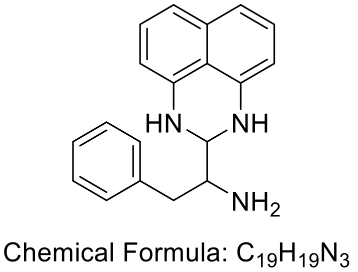

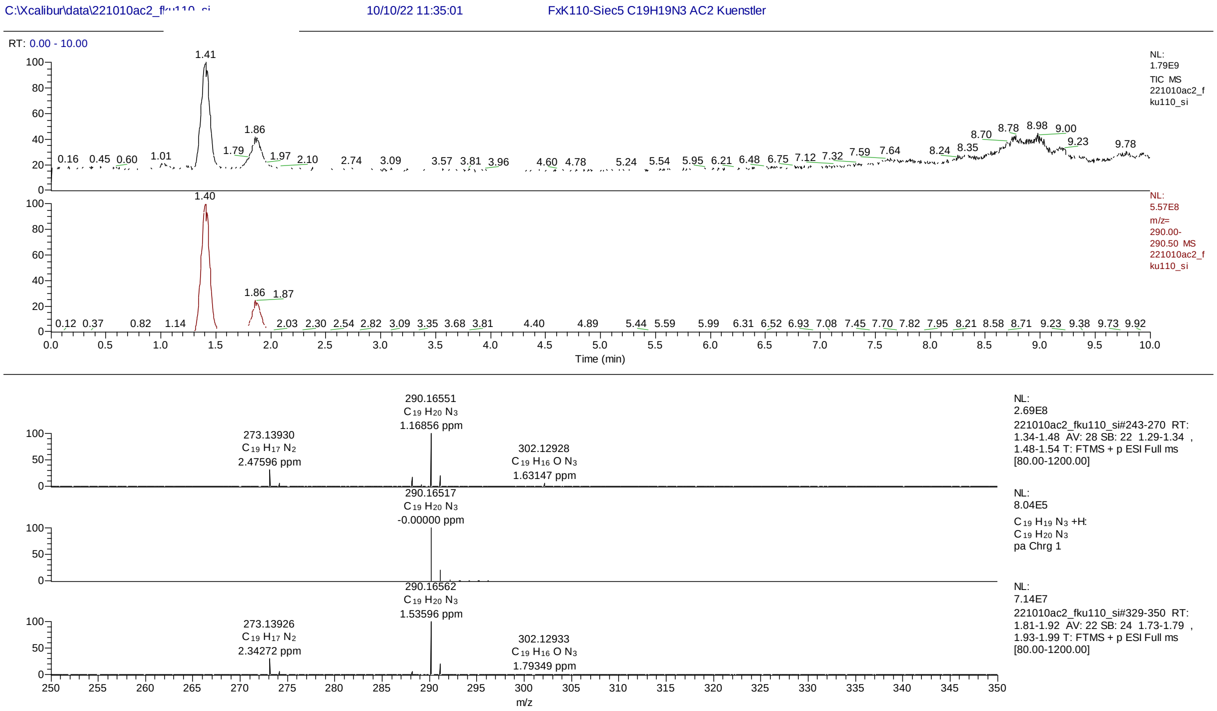

Supplementary Figure 204 LC-HRMS spectrum of compound **A26**.

LC-HRMS of A27

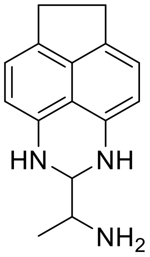

Chemical Formula: $C_{15}H_{17}N_3$

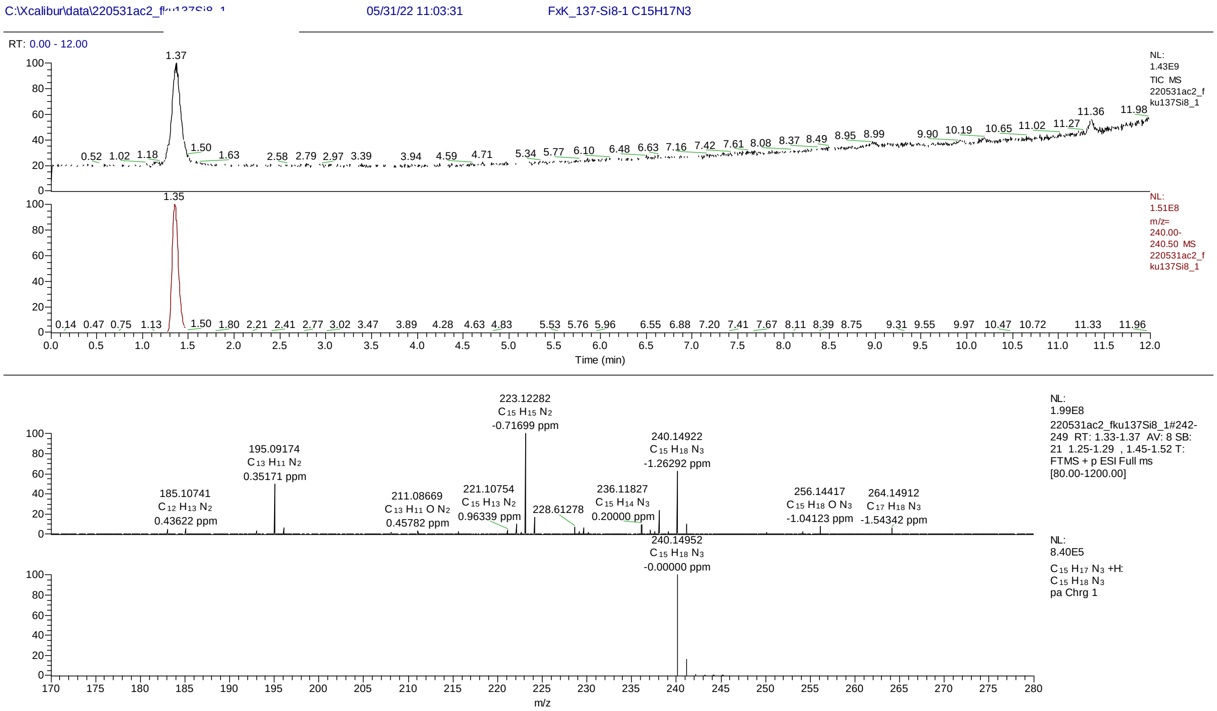

Supplementary Figure 205 LC-HRMS spectrum of compound **A27**.

LC-HRMS of B6a

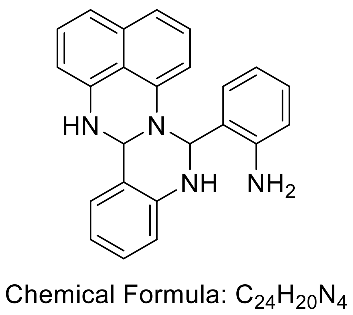

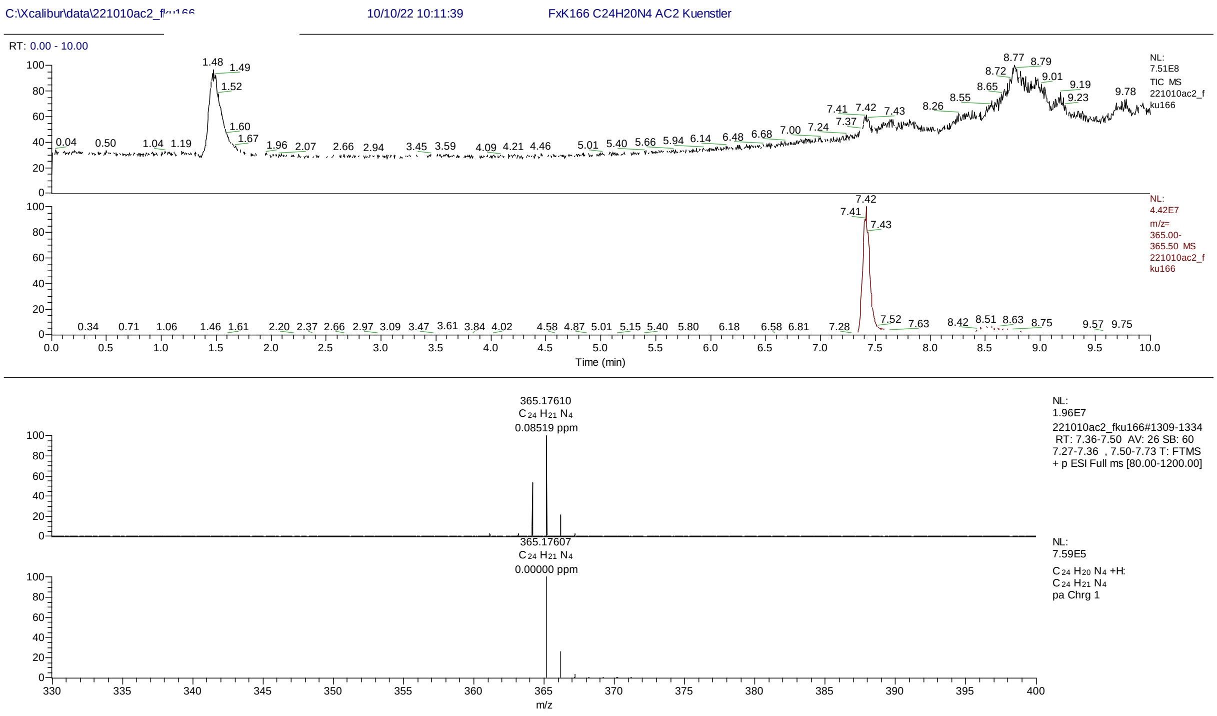

Supplementary Figure 206 LC-HRMS spectrum of compound **B6a**.

LC-HRMS of B6b

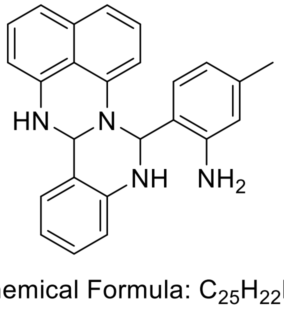

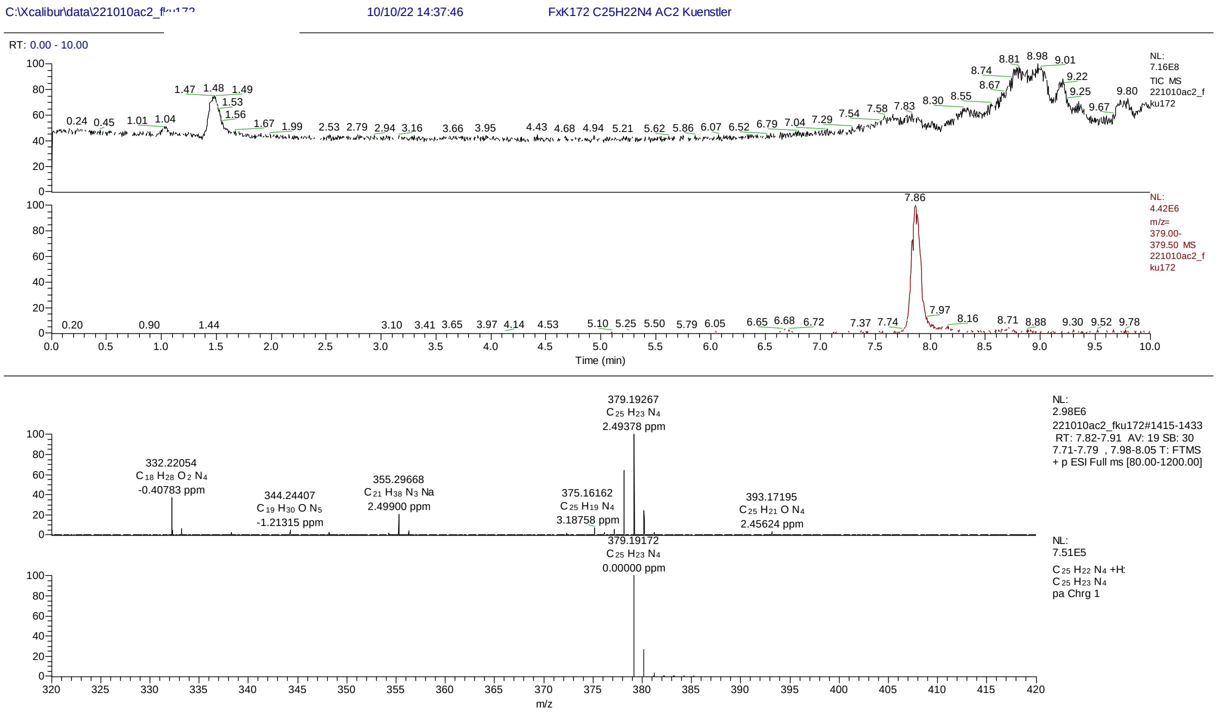

Supplementary Figure 207 LC-HRMS spectrum of compound **B6b**.

LC-HRMS of B6c

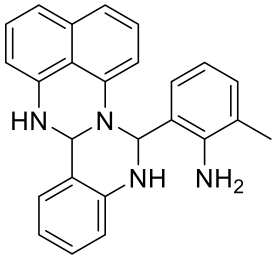

Chemical Formula: $C_{25}H_{22}N_4$

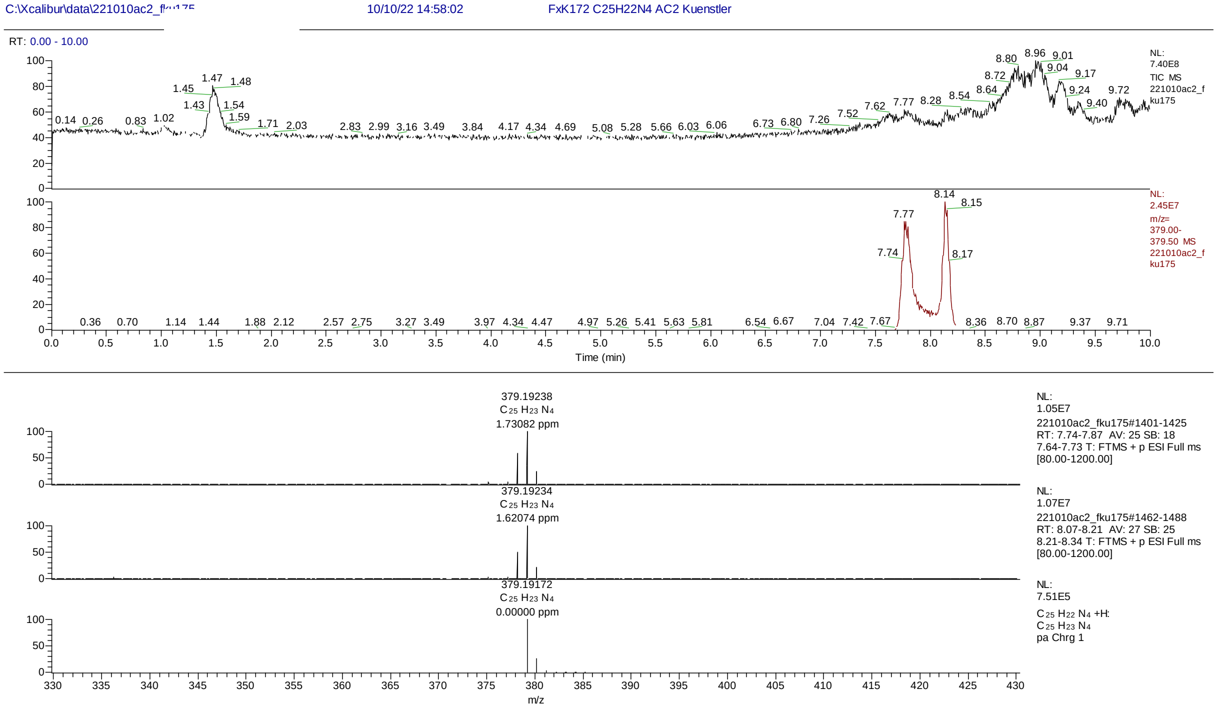

Supplementary Figure 208 LC-HRMS spectrum of compound **B6c**.

LC-HRMS of C1

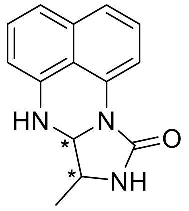

Chemical Formula: $C_{14}H_{13}N_3O$

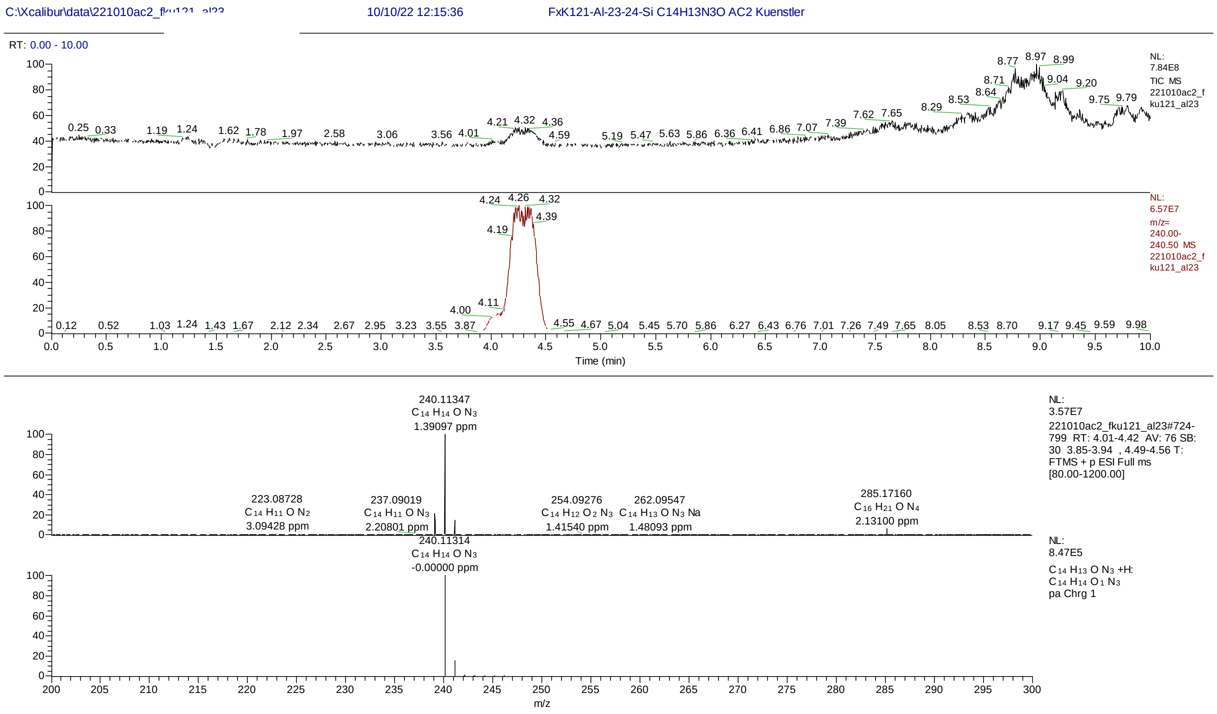

Supplementary Figure 209 LC-HRMS spectrum of compound **C1**.

LC-HRMS of C2

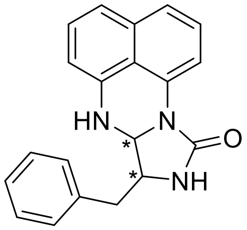

Chemical Formula: $C_{20}H_{17}N_3O$

main isomer of **C2**:

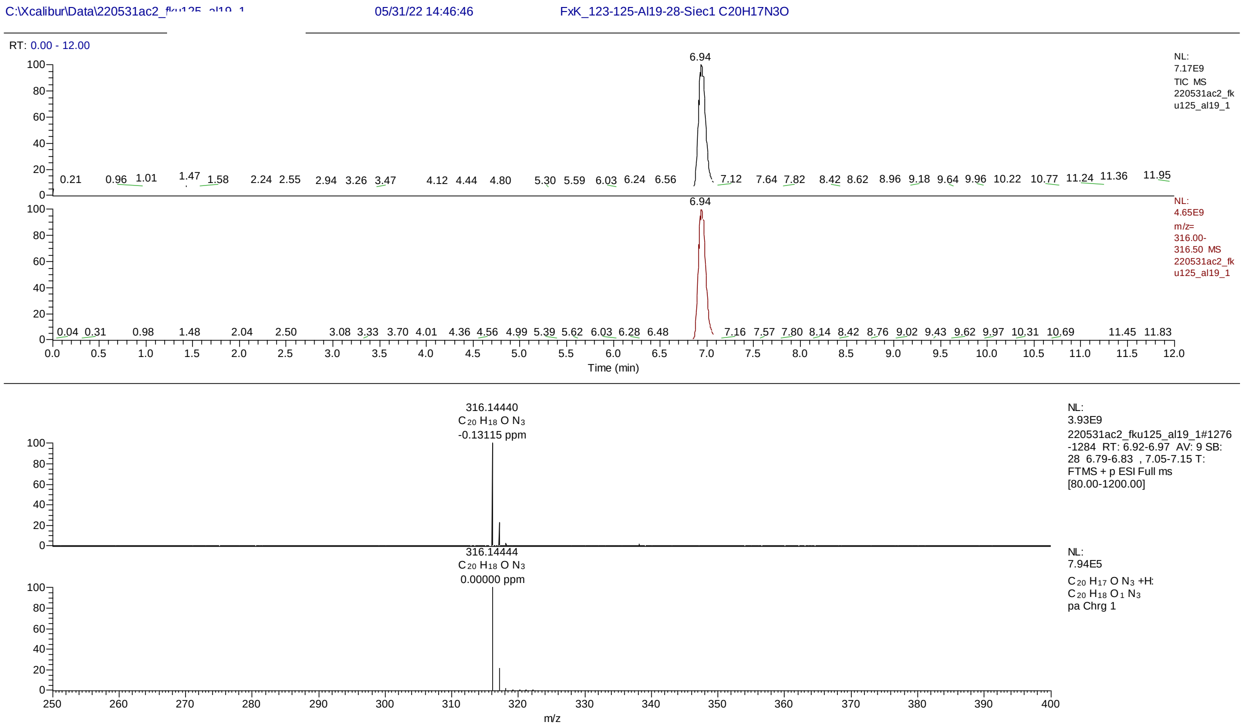

Supplementary Figure 210 LC-HRMS spectrum of the main isomer of compound **C2**.

minor isomer of **C2**:

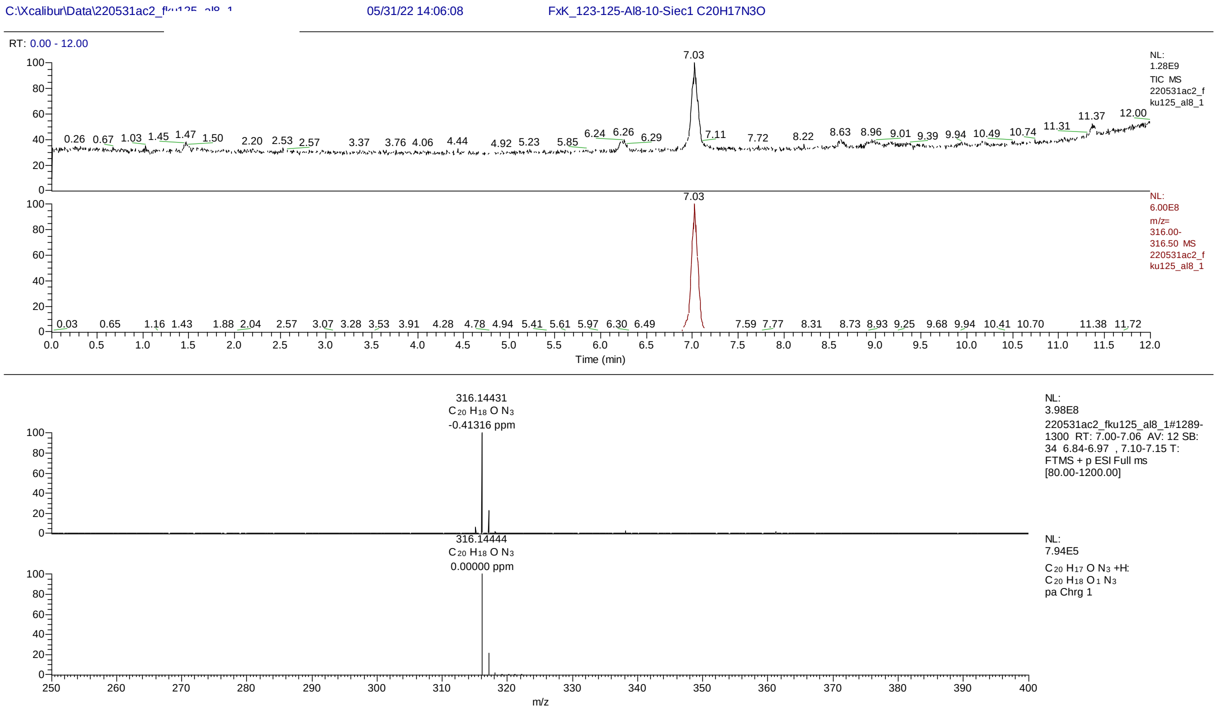

Supplementary Figure 211 LC-HRMS spectrum of the minor isomer of compound **C2**.

LC-HRMS of C3

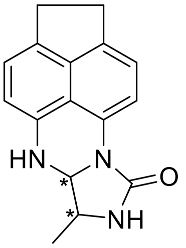

Chemical Formula: $C_{16}H_{15}N_3O$

main isomer of **C3**:

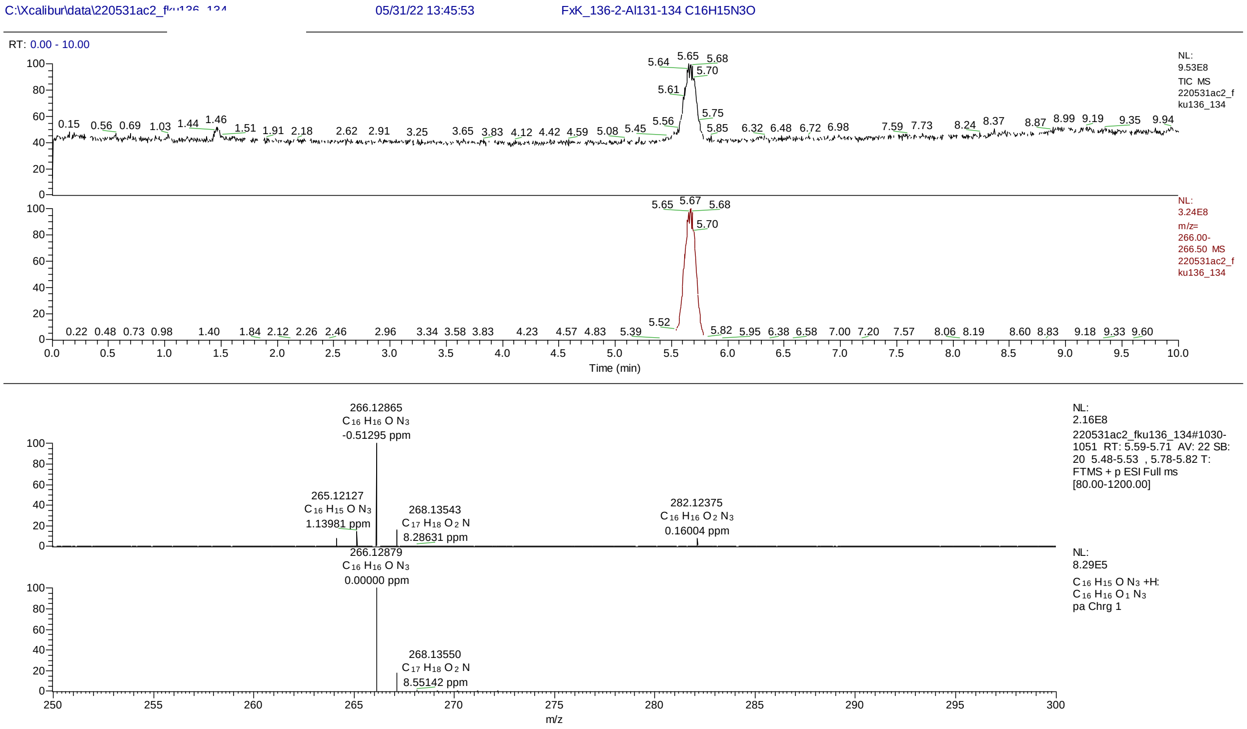

Supplementary Figure 212 LC-HRMS spectrum of the main isomer of compound **C3**.

minor isomer of **C3**:

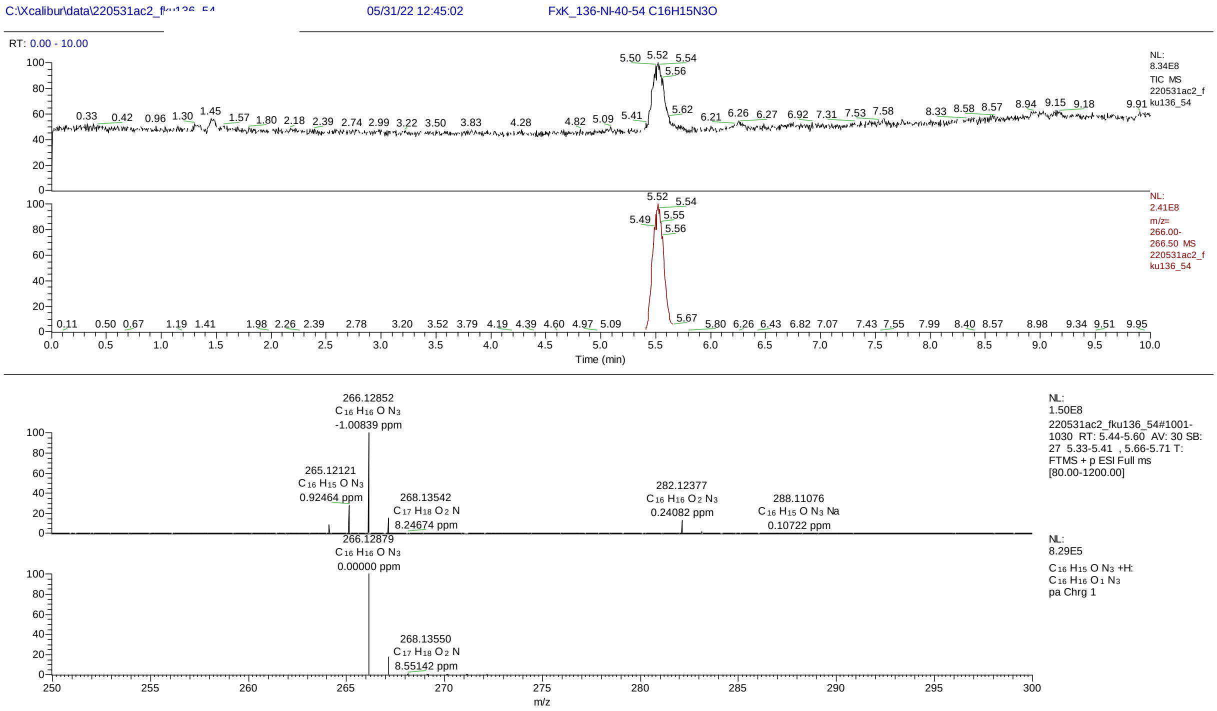

Supplementary Figure 213 LC-HRMS spectrum of the minor isomer of compound **C3**.

Blind sample of ethanol

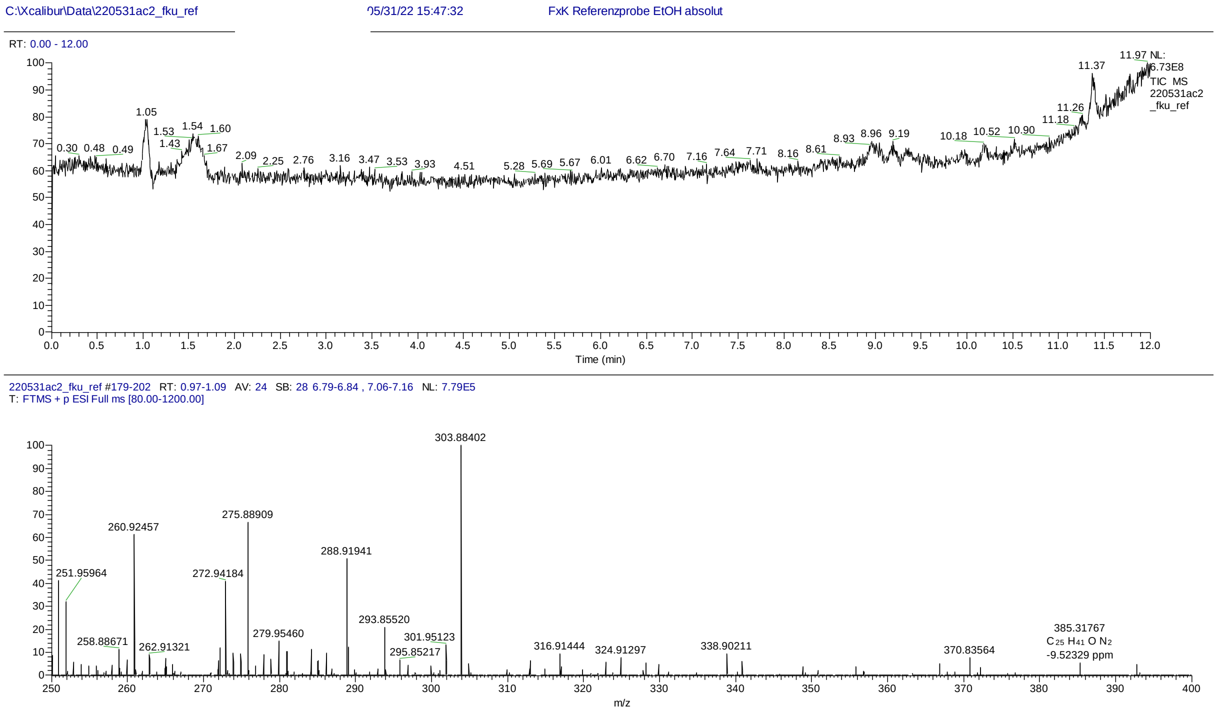

Supplementary Figure 214 LC-HRMS spectrum of the used solvent ethanol.

Blind sample of DMSO

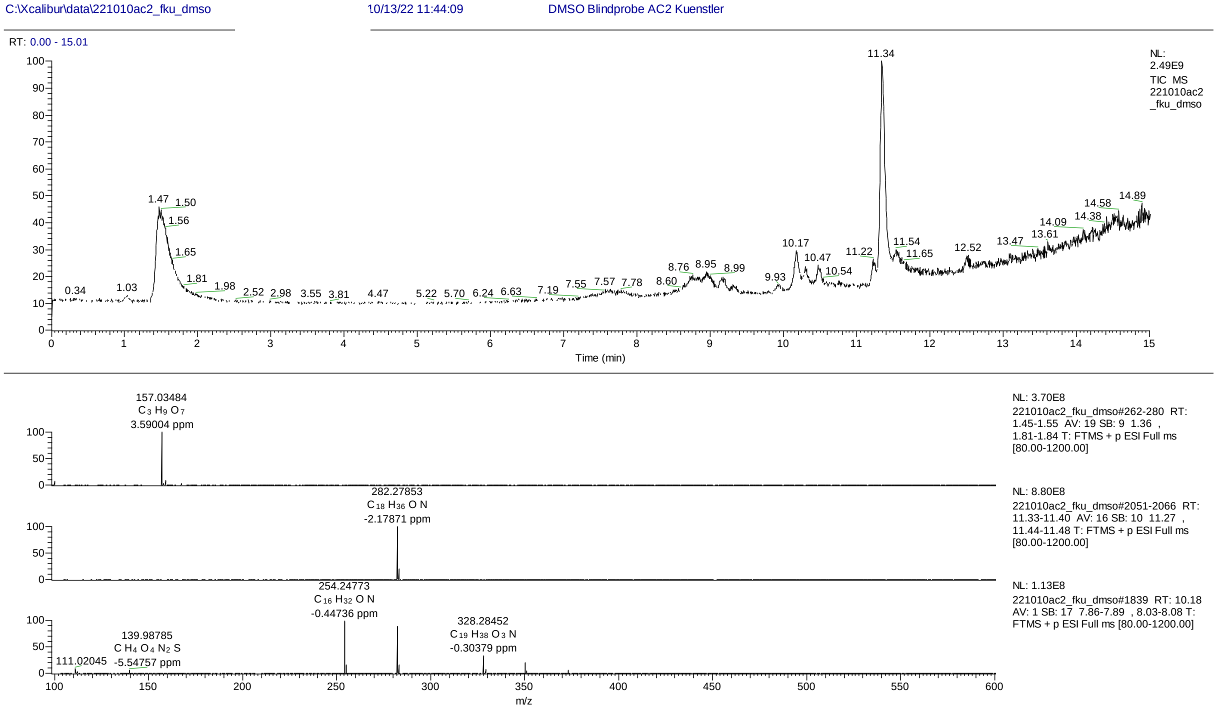

Supplementary Figure 215 LC-HRMS spectrum of the used solvent DMSO.

16. Crystallographic data

Supplementary Data 1: Crystallographic details of **A1** (CCDC number: 2084882).

checkCIF/PLATON report

Structure factors have been supplied for datablock(s) sv481_1_te_i41_2

THIS REPORT IS FOR GUIDANCE ONLY. IF USED AS PART OF A REVIEW PROCEDURE FOR PUBLICATION, IT SHOULD NOT REPLACE THE EXPERTISE OF AN EXPERIENCED CRYSTALLOGRAPHIC REFEREE.

No syntax errors found. CIF dictionary Interpreting this report

Datablock: sv481_1_te_i41_2

```
Bond precision:  C-C = 0.0051 A          Wavelength=0.71073

Cell:              a=22.690(3)      b=22.690(3)      c=10.320(2)
                   alpha=90         beta=90          gamma=90
Temperature:       133 K

                Calculated                Reported
Volume          5313.1(17)                5313.1(18)
Space group     I 41                      I 41
Hall group      I 4bw                     I 4bw
Moiety formula  C17 H15 N3                1.143(C17 H15 N3)
Sum formula     C17 H15 N3                C19.43 H17.14 N3.43
Mr              261.32                    298.65
Dx,g cm-3       1.307                     1.307
Z               16                        14
Mu (mm-1)       0.079                     0.079
F000            2208.0                    2208.0
F000'           2208.70
h,k,lmax        30,30,13                  27,30,13
Nref            6770[ 3568]               4150
Tmin,Tmax       0.998,1.000
Tmin'           0.996

Correction method= Not given

Data completeness= 1.16/0.61      Theta(max)= 28.529

R(reflections)= 0.0507( 3042)      wR2(reflections)= 0.1234( 4150)

S = 0.974                  Npar= 369
```

The following ALERTS were generated. Each ALERT has the format
test-name_ALERT_alert-type_alert-level.
Click on the hyperlinks for more details of the test.

Alert level B

```
PLAT417_ALERT_2_B Short Inter D-H..H-D       Hn1       ..Hn3B     .       1.94 Ang.
                                        -1/2+y,1-x,-1/4+z  =      4_464 Check
```

Alert level C

```
STRVA01_ALERT_2_C           Chirality of atom sites is inverted?
           From the CIF: _refine_ls_abs_structure_Flack     5.000
           From the CIF: _refine_ls_abs_structure_Flack_su     4.000
PLAT340_ALERT_3_C Low Bond Precision on  C-C Bonds ..............    0.00508 Ang.
PLAT420_ALERT_2_C D-H Bond Without Acceptor  N2        --Hn2       .     Please Check
PLAT420_ALERT_2_C D-H Bond Without Acceptor  N4        --Hn4       .     Please Check
PLAT420_ALERT_2_C D-H Bond Without Acceptor  N1        --Hn1       .     Please Check
PLAT420_ALERT_2_C D-H Bond Without Acceptor  N5        --Hn5       .     Please Check
PLAT420_ALERT_2_C D-H Bond Without Acceptor  N3        --Hn3B      .     Please Check
PLAT420_ALERT_2_C D-H Bond Without Acceptor  N3        --Hn3A      .     Please Check
PLAT420_ALERT_2_C D-H Bond Without Acceptor  N6        --Hn6B      .     Please Check
PLAT601_ALERT_2_C Unit Cell Contains Solvent Accessible VOIDS of .         32 Ang**3
PLAT907_ALERT_2_C Flack x > 0.5, Structure Needs to be Inverted? .       5.00 Check
PLAT911_ALERT_3_C Missing FCF Refl Between Thmin & STh/L=    0.600         21 Report
```

Alert level G

```
CELLZ01_ALERT_1_G Difference between formula and atom_site contents detected.
CELLZ01_ALERT_1_G ALERT: check formula stoichiometry or atom site occupancies.
           From the CIF: _cell_formula_units_Z    14
           From the CIF: _chemical_formula_sum  C19.43 H17.14 N3.43
           TEST: Compare cell contents of formula and atom_site data

           atom    Z*formula  cif sites diff
           C         272.02    272.00    0.02
           H         239.96    240.00   -0.04
           N          48.02     48.00    0.02
PLAT007_ALERT_5_G Number of Unrefined Donor-H Atoms .............          6 Report
PLAT032_ALERT_4_G Std. Uncertainty on Flack Parameter Value High .      4.000 Report
PLAT042_ALERT_1_G Calc. and Reported Moiety Formula Strings Differ     Please Check
PLAT045_ALERT_1_G Calculated and Reported Z Differ by a Factor ...       1.14 Check
PLAT180_ALERT_4_G Check Cell Rounding: # of Values Ending with 0 =          3 Note
PLAT720_ALERT_4_G Number of Unusual/Non-Standard Labels .........          8 Note
PLAT870_ALERT_4_G ALERTS Related to Twinning Effects Suppressed ..          ! Info
PLAT910_ALERT_3_G Missing # of FCF Reflection(s) Below Theta(Min).          1 Note
PLAT912_ALERT_4_G Missing # of FCF Reflections Above STh/L=  0.600        173 Note
PLAT916_ALERT_2_G Hooft y and Flack x Parameter Values Differ by .       5.60 Check
PLAT941_ALERT_3_G Average HKL Measurement Multiplicity ..........        3.5 Low
PLAT950_ALERT_5_G Calculated (ThMax) and CIF-Reported Hmax Differ          3 Units
```

---

0 **ALERT level A** = Most likely a serious problem - resolve or explain
1 **ALERT level B** = A potentially serious problem, consider carefully
12 **ALERT level C** = Check. Ensure it is not caused by an omission or oversight
14 **ALERT level G** = General information/check it is not something unexpected

4 ALERT type 1 CIF construction/syntax error, inconsistent or missing data
12 ALERT type 2 Indicator that the structure model may be wrong or deficient
4 ALERT type 3 Indicator that the structure quality may be low
5 ALERT type 4 Improvement, methodology, query or suggestion
2 ALERT type 5 Informative message, check

It is advisable to attempt to resolve as many as possible of the alerts in all categories. Often the minor alerts point to easily fixed oversights, errors and omissions in your CIF or refinement strategy, so attention to these fine details can be worthwhile. In order to resolve some of the more serious problems it may be necessary to carry out additional measurements or structure refinements. However, the purpose of your study may justify the reported deviations and the more serious of these should normally be commented upon in the discussion or experimental section of a paper or in the "special_details" fields of the CIF. checkCIF was carefully designed to identify outliers and unusual parameters, but every test has its limitations and alerts that are not important in a particular case may appear. Conversely, the absence of alerts does not guarantee there are no aspects of the results needing attention. It is up to the individual to critically assess their own results and, if necessary, seek expert advice.

Publication of your CIF in IUCr journals

A basic structural check has been run on your CIF. These basic checks will be run on all CIFs submitted for publication in IUCr journals (*Acta Crystallographica*, *Journal of Applied Crystallography*, *Journal of Synchrotron Radiation*); however, if you intend to submit to *Acta Crystallographica Section C* or *E* or *IUCrData*, you should make sure that full publication checks are run on the final version of your CIF prior to submission.

Publication of your CIF in other journals

Please refer to the *Notes for Authors* of the relevant journal for any special instructions relating to CIF submission.

PLATON version of 16/05/2021; check.def file version of 13/05/2021

Datablock sv481_1_te_i41_2 - ellipsoid plot

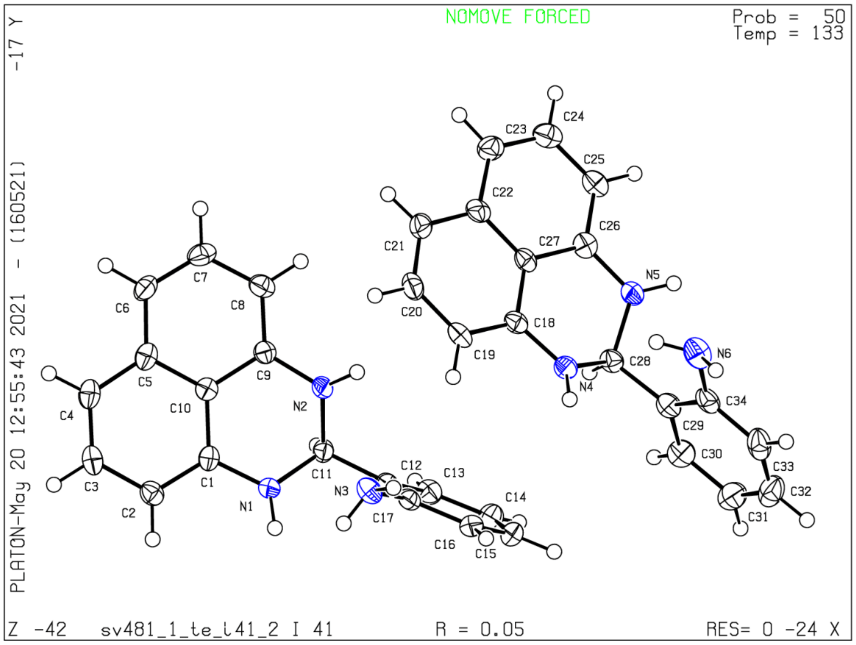

Supplementary Figure 216 Molecular structure of **A1**.

Supplementary Data 2: Crystallographic details of **B1a** (CCDC number: 2083140)

checkCIF/PLATON report

Structure factors have been supplied for datablock(s) sv499_1_m_p21c

THIS REPORT IS FOR GUIDANCE ONLY. IF USED AS PART OF A REVIEW PROCEDURE FOR PUBLICATION, IT SHOULD NOT REPLACE THE EXPERTISE OF AN EXPERIENCED CRYSTALLOGRAPHIC REFEREE.

No syntax errors found.   CIF dictionary   Interpreting this report

Datablock: sv499_1_m_p21c

```
Bond precision:  C-C = 0.0020 A          Wavelength=0.71073

Cell:            a=5.7600(12)     b=11.550(2)       c=25.410(5)
                 alpha=90         beta=90.70(3)     gamma=90
Temperature:     133 K

                Calculated                Reported
Volume          1690.4(6)                 1690.4(6)
Space group     P 21/c                    P 1 21/c 1
Hall group      -P 2ybc                   -P 2ybc
Moiety formula  C24 H19 N3                C24 H19 N3
Sum formula     C24 H19 N3                C24 H19 N3
Mr              349.42                    349.42
Dx,g cm-3       1.373                     1.373
Z               4                         4
Mu (mm-1)       0.082                     0.082
F000            736.0                     736.0
F000'           736.24
h,k,lmax        7,15,34                   7,15,33
Nref            4271                      4090
Tmin,Tmax       0.995,0.997
Tmin'           0.985

Correction method= Not given

Data completeness= 0.958          Theta(max)= 28.448

R(reflections)= 0.0433( 2915)     wR2(reflections)= 0.1155( 4090)

S = 1.039                 Npar= 320
```

The following ALERTS were generated. Each ALERT has the format
test-name_ALERT_alert-type_alert-level.
Click on the hyperlinks for more details of the test.

Alert level C

```
CRYSC01_ALERT_1_C The word below has not been recognised as a standard
            identifier.
            yellowish
PLAT222_ALERT_3_C NonSolvent Resd 1  H   Uiso(max)/Uiso(min) Range        7.0 Ratio
PLAT245_ALERT_2_C U(iso) H24       Smaller than U(eq) C24       by      0.015 Ang**2
PLAT410_ALERT_2_C Short Intra H...H Contact  H1        ..H8       .       1.96 Ang.
                                                       x,y,z  =      1_555 Check
PLAT420_ALERT_2_C D-H Bond Without Acceptor  N1        --Hn1      .     Please Check
PLAT420_ALERT_2_C D-H Bond Without Acceptor  N3        --H5       .     Please Check
PLAT906_ALERT_3_C Large K Value in the Analysis of Variance ......      2.149 Check
PLAT911_ALERT_3_C Missing FCF Refl Between Thmin & STh/L=    0.600          7 Report
```

Alert level G

```
PLAT180_ALERT_4_G Check Cell Rounding: # of Values Ending with 0 =          4 Note
PLAT720_ALERT_4_G Number of Unusual/Non-Standard Labels ..........          1 Note
PLAT793_ALERT_4_G Model has Chirality at C11         (Centro SPGR)          S Verify
PLAT793_ALERT_4_G Model has Chirality at C18         (Centro SPGR)          R Verify
PLAT910_ALERT_3_G Missing # of FCF Reflection(s) Below Theta(Min).          1 Note
PLAT912_ALERT_4_G Missing # of FCF Reflections Above STh/L=  0.600        171 Note
PLAT941_ALERT_3_G Average HKL Measurement Multiplicity ...........        3.2 Low
PLAT978_ALERT_2_G Number C-C Bonds with Positive Residual Density.         17 Info
PLAT992_ALERT_5_G Repd & Actual _reflns_number_gt Values Differ by          2 Check
```

---

0 **ALERT level A** = Most likely a serious problem - resolve or explain
0 **ALERT level B** = A potentially serious problem, consider carefully
8 **ALERT level C** = Check. Ensure it is not caused by an omission or oversight
9 **ALERT level G** = General information/check it is not something unexpected

1 ALERT type 1 CIF construction/syntax error, inconsistent or missing data
5 ALERT type 2 Indicator that the structure model may be wrong or deficient
5 ALERT type 3 Indicator that the structure quality may be low
5 ALERT type 4 Improvement, methodology, query or suggestion
1 ALERT type 5 Informative message, check

It is advisable to attempt to resolve as many as possible of the alerts in all categories. Often the minor alerts point to easily fixed oversights, errors and omissions in your CIF or refinement strategy, so attention to these fine details can be worthwhile. In order to resolve some of the more serious problems it may be necessary to carry out additional measurements or structure refinements. However, the purpose of your study may justify the reported deviations and the more serious of these should normally be commented upon in the discussion or experimental section of a paper or in the "special_details" fields of the CIF. checkCIF was carefully designed to identify outliers and unusual parameters, but every test has its limitations and alerts that are not important in a particular case may appear. Conversely, the absence of alerts does not guarantee there are no aspects of the results needing attention. It is up to the individual to critically assess their own results and, if necessary, seek expert advice.

Publication of your CIF in IUCr journals

A basic structural check has been run on your CIF. These basic checks will be run on all CIFs submitted for publication in IUCr journals (*Acta Crystallographica*, *Journal of Applied Crystallography*, *Journal of Synchrotron Radiation*); however, if you intend to submit to *Acta Crystallographica Section C* or *E* or *IUCrData*, you should make sure that full publication checks are run on the final version of your CIF prior to submission.

Publication of your CIF in other journals

Please refer to the *Notes for Authors* of the relevant journal for any special instructions relating to CIF submission.

PLATON version of 22/03/2021; check.def file version of 19/03/2021

Datablock sv499_1_m_p21c - ellipsoid plot

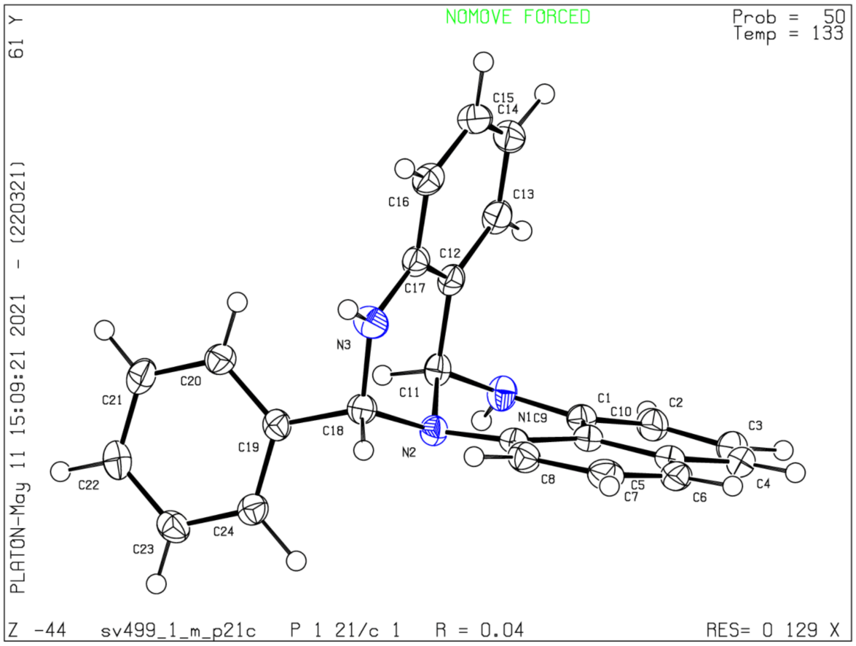

Supplementary Figure 217 Molecular structure of **B1a.**

Supplementary References

1. Dolomanov, O. V., Bourhis, L. J., Gildea, R. J., Howard, J. A. K. & Puschmann, H. OLEX2: A complete structure solution, refinement and analysis program. *J. Appl. Cryst.* **42**, 339–341 (2009).
2. Sheldrick, G. M. Crystal structure refinement with SHELXL. *Acta Crystallogr. Sect. C Struct. Chem.* **71**, 3–8 (2015).
3. MacRae, C. F. *et al.* Mercury 4.0: From visualization to analysis, design and prediction. *J. Appl. Cryst.* **53**, 226–235 (2020).
4. Kallmeier, F., Dudziec, B., Irrgang, T. & Kempe, R. Manganese-Catalyzed Sustainable Synthesis of Pyrroles from Alcohols and Amino Alcohols. *Angew. Chem. Int. Ed.* **56**, 7261–7265 (2017).
5. Mastalir, M., Glatz, M., Pittenauer, E., Allmaier, G. & Kirchner, K. Sustainable Synthesis of Quinolines and Pyrimidines Catalyzed by Manganese PNP Pincer Complexes. *J. Am. Chem. Soc.* **138**, 15543–15546 (2016).
6. Mastalir, M. *et al.* Divergent Coupling of Alcohols and Amines Catalyzed by Isoelectronic Hydride Mn(I) and Fe(II) PNP Pincer Complexes. *Chem. Eur. J.* **22**, 12316–12320 (2016).
7. Freitag, F., Irrgang, T. & Kempe, R. Cobalt-Catalyzed Alkylation of Secondary Alcohols with Primary Alcohols via Borrowing Hydrogen/Hydrogen Autotransfer. *Chem. Eur. J.* **23**, 12110–12113 (2017).
8. Rösler, S., Ertl, M., Irrgang, T. & Kempe, R. Cobalt-Catalyzed Alkylation of Aromatic Amines by Alcohols. *Angew. Chem. Int. Ed.* **54**, 15046–15050 (2015).
9. Mastalir, M. *et al.* Air Stable Iron(II) PNP Pincer Complexes as Efficient Catalysts for the Selective Alkylation of Amines with Alcohols. *Adv. Synth. Catal.* **358**, 3824–3831 (2016).
